# Deoxycyanation of Alkyl Alcohols Using Photoredox Catalysis

**DOI:** 10.1021/acs.orglett.6c00711

**Published:** 2026-04-29

**Authors:** Carla Hümpel, William L. Lyon, Ryan E. McNamee, David W. C. MacMillan, Jingjia Chen

**Affiliations:** Merck Center for Catalysis at Princeton University, Princeton, New Jersey 08544, United States

## Abstract

Cyano groups represent an important class of functional motifs in medicinal chemistry given their synthetic versatility and capacity to engage in essential interactions with biological targets. However, the synthesis of sterically hindered alkyl nitriles remains challenging, and, furthermore, traditional methods often rely on toxic cyanide sources. Herein, we report a photoredox-catalyzed, metal-free deoxycyanation of alkyl alcohols that allows rapid access to a wide array of 1°, 2°, and 3° cyanides using the easily handled, low-toxicity, tosyl cyanide reagent.

## Graphical abstract

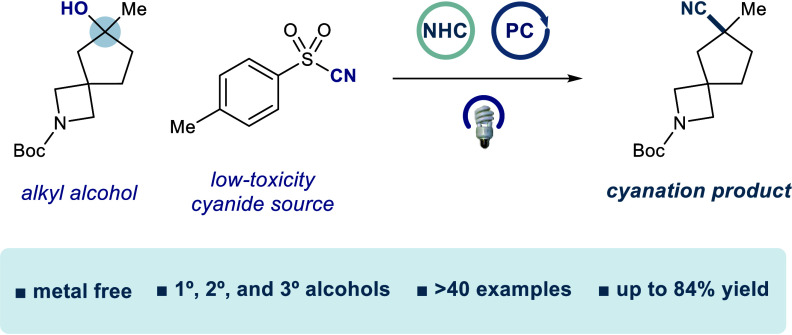

Cyanide-containing compounds exhibit unique chemical properties and are widely utilized across pharmaceuticals, materials science, and agrochemicals.^1,2^ In medicinal chemistry, nitriles are particularly valued for their ability to engage in critical hydrogen bonding or covalent interactions with biological targets.^3^ Combined with their metabolic stability, polarity, and linear geometry, these features make the cyano group a distinctive and highly sought-after motif in drug design (Figure 1A), with over 70 approved drugs incorporating this functionality.^4^ In addition to their biological relevance, nitriles serve as versatile synthetic handles, readily transforming into amines, amides, acids, and heteroaromatics.^5^ Despite these advantages, the development of general and mild methods for nitrile synthesis using less toxic reagents remains an important goal. While aryl nitriles are readily accessible, the synthesis of alkyl nitriles—particularly sterically hindered tertiary variants—remains challenging. Traditional approaches, such as transition metal-catalyzed cross-coupling, and cyanide addition to electrophiles,^3,6^ while effective for primary and secondary alkyl nitriles, are often limited in their ability to deliver sterically encumbered tertiary alkyl cyanides and typically require the use of highly toxic cyanide reagents.

**Figure 1:**
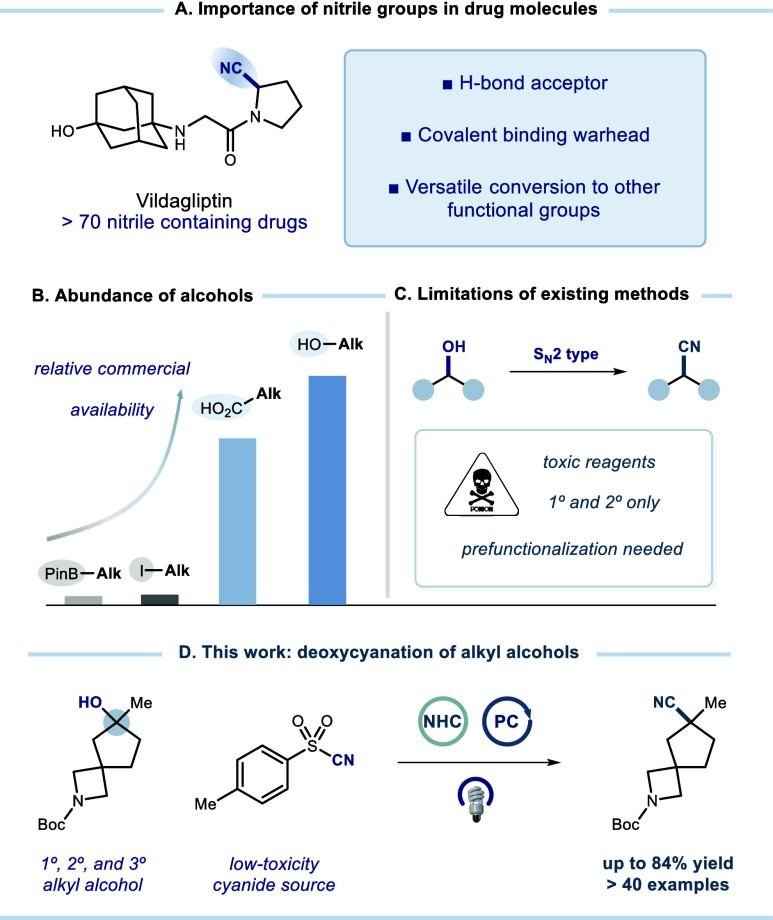
Deoxycyanation of alkyl alcohols.

Due to their versatility and high reactivity, radical intermediates offer a promising solution for the synthesis of sterically encumbered tertiary alkyl cyanides.^7^ Over the past two decades, photoredox catalysis has emerged as a powerful tool for generating radical intermediates and enabling challenging bond formations.^8^ Various photoredox-based approaches have been explored for the construction of C­(sp^3^)–CN bonds, including decarboxylation,^9,10^ hydrogen atom transfer (HAT),^11,12^ oxidation-deprotonation,^13,14^ radical substitution,^14^ ring opening,^15^ XAT,^16^ and olefination.^17,18^ However, many of these methods continue to rely on toxic cyanide reagents, suffer from limited substrate scope, or prefunctionalizations.

Alcohols, among the most abundant sources of functional C­(sp^3^) centers (Figure 1B),^19,20^ are highly attractive starting materials for building block synthesis and late-stage functionalization (Figure 1C). Recent work by the Kim group demonstrated the synthesis of secondary alkyl cyanides from SuFEx-activated alcohols via an S_N_2-type mechanism.^21^ Similarly, Han and co-workers successfully synthesized tertiary alkyl cyanides using α-N-phthalimido-oxy isobutyrate (NPIB)-activated alcohols under copper-mediated metallaphotoredox conditions.^22^ While we were preparing this manuscript, the Tortosa group also reported cyanation of alkyl scaffolds using decarboxylation, deoxygenation, and dehalogenation with a newly synthesized trityl isocyanide.^23^ These approaches, while inspiring, are limited by a narrow substrate scope or the use of toxic TMSCN reagents. Thus, we envisioned that a low-toxicity, general one-step method for the deoxycyanation of alkyl alcohols would be highly desirable.

Previously, the MacMillan group has introduced a benzoxazolium salt (″NHC″) as a convenient alcohol-activating reagent.^24^ Employing this strategy, alcohol–NHC adducts are formed *in situ* under mild conditions and readily undergo photoredox-mediated deoxygenation to generate reactive alkyl radicals. We envisioned that radical addition of this nucleophilic alkyl radical species into tosyl cyanide, a low-toxicity electrophilic partner, would allow for the efficient formation of cyanation products.^25^

The proposed mechanistic design is outlined in Figure 2. First, alkyl alcohol **1** reacts with benzoxazolium salt **2** to form NHC–alcohol adduct **3**. Blue light irradiation of photocatalyst (PC) 4-CzPN (**4**) generates a highly oxidizing excited species **5** ^26^ that can be quenched by adduct **3** via single-electron transfer (SET). Subsequent deprotonation of the now acidified methine C–H (p*K*_a_ ∼ 10)^24^ would provide α-amino radical **7**, which can then undergo facile β-scission^27^ of the alcohol C–O bond to afford the alkyl radical **8** and an inert byproduct. The nucleophilic alkyl radical would then add to the electrophilic tosyl cyanide **9** to afford the desired cyanation product **10**,^28–31^ while reduced photocatalyst **6** would be reoxidized to **4** with an external oxidant.

**Figure 2:**
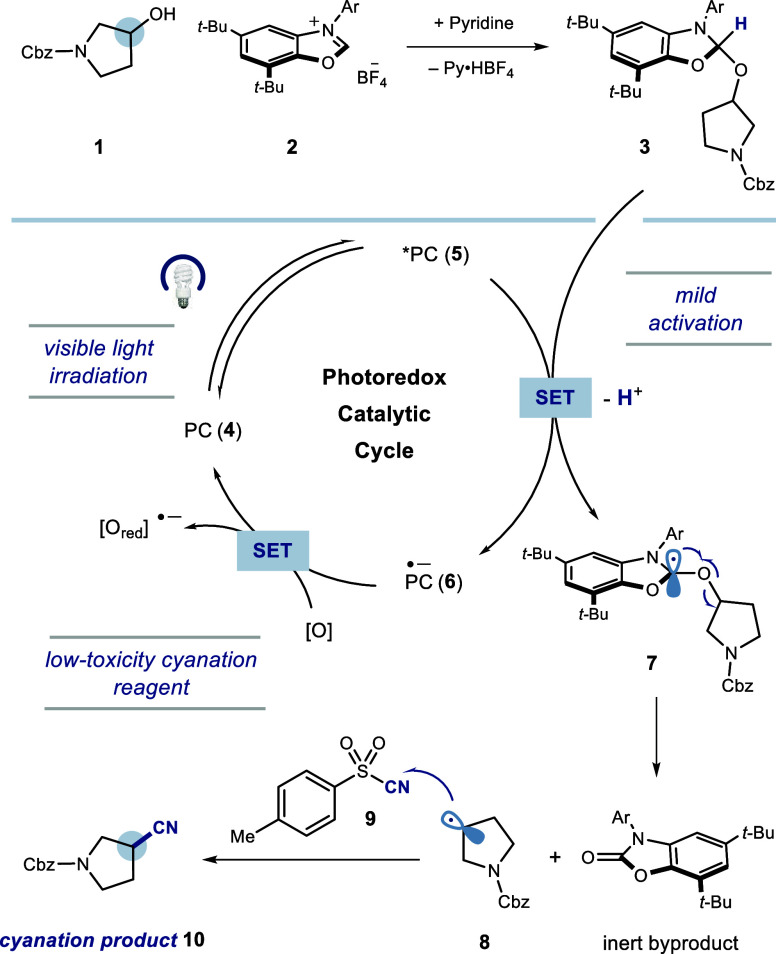
Proposed mechanism for deoxycyanation.

Extensive optimization revealed that mixing benzyl 3-hydroxypyrrolidine-1-carboxylate (**1**) with **NHC-J** (1.2 equiv) and pyridine (1.5 equiv) in methyl *tert*-butyl ether (MTBE) [0.1M] followed by filtration and subsequent irradiation with 450 nm light in the presence of tosyl cyanide **9** (1.5 equiv), 4-CzPN **4** (2 mol %), benzoyl peroxide (Bz_2_O_2_,1 equiv), and 2,4,6-trimethylpyridine (Me_3_Pyr) in a solvent mixture of MTBE/acetone/water (15:15:1) [0.017 M] afforded the desired cyanation product in 54% yield (Table S1, entry 1). Control experiments demonstrated that all components of the reaction are essential for efficient product formation. However, using air as additional or the only oxidant is both detrimental to the reaction (Table S1, entries 2 and 3). Filtration of the NHC condensate mixture or using water as a cosolvent proved beneficial (Table S1, entries 5–6).

With optimized conditions in hand, we proceeded to explore the scope of our method with respect to the alcohol (Table 1). We were delighted to find that a variety of unactivated secondary alcohols perform well in the reaction, affording the desired cyanation products in good yields (**10**–**19**, 50–71% yield). Given the prevalence of tertiary alcohols and their accessibility through simple nucleophilic addition into ketones and esters, we wondered whether our method could be used to access tertiary cyanides from their corresponding alcohols. Remarkably, a broad range of cyclic tertiary alcohols, including those embedded in four-, five-, six-, and seven-membered saturated heterocycles, were successfully converted to their corresponding nitriles in good yields (**20**–**25**, 51–67% yield). Additionally, linear alcohols underwent efficient deoxycyanation (**26**–**28**, 51–84%). Notably, our method proved uniquely effective for highly challenging substrates such as spirocyclic and strained tertiary alcohols, which have remained largely inaccessible by traditional means, delivering the desired products in synthetically useful to excellent yields (**29**–**34**, 57–75% yield). However, our method is more limited for primary alcohols (**35–39**, 30–39% yield), likely due to the relative instability and reduced nucleophilicity of the corresponding primary radical species.

**Table 1:**
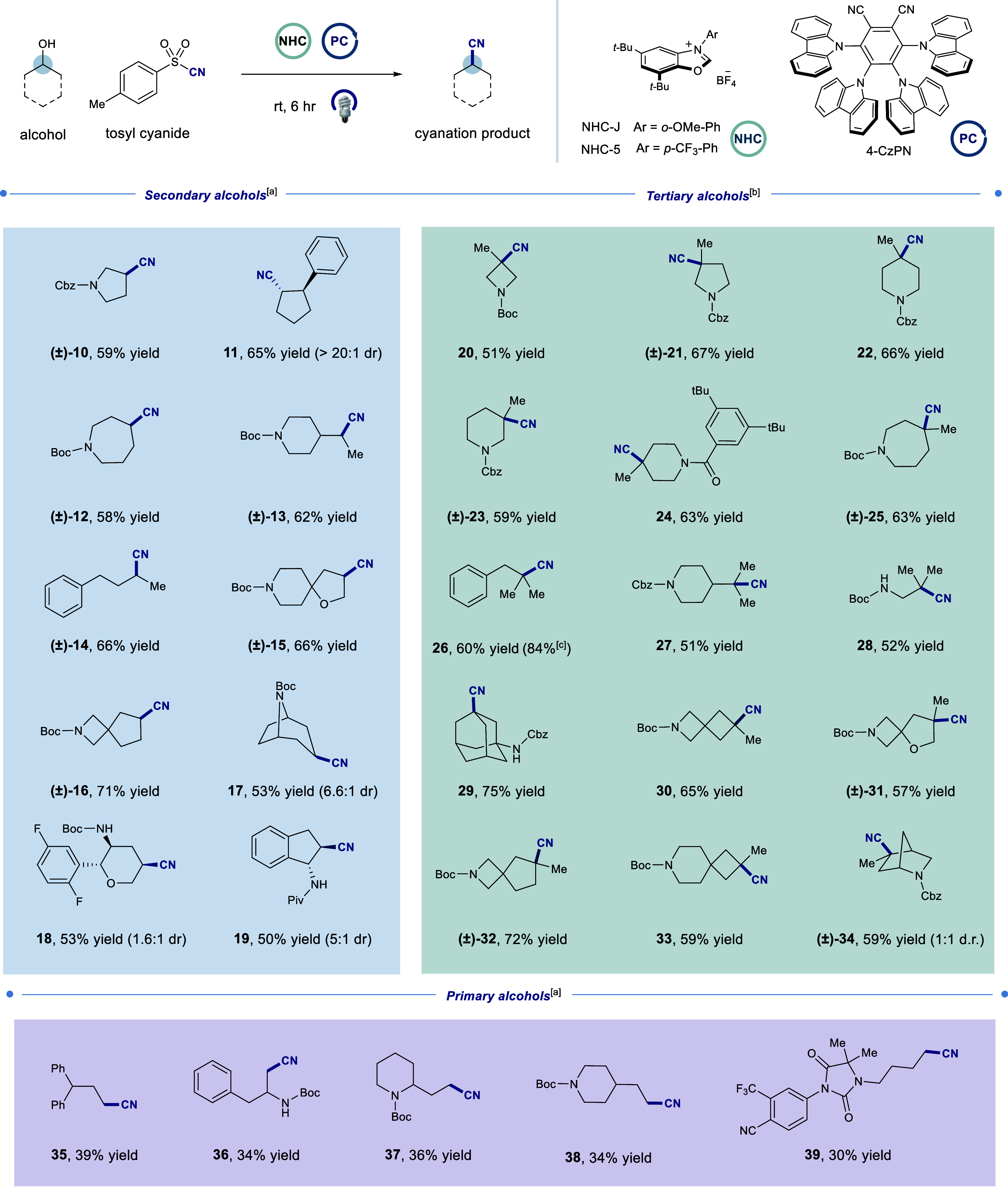
Scope of Alcohol Building Blocks^a^

a Standard conditions: Alcohol (0.5 mmol, 1 equiv), **NHC-J** (1.2 equiv), pyridine (1.5 equiv), MTBE (0.10 M), 45 min; Tosyl cyanide (1.5 equiv), 4-CzPN (2 mol %), Bz_2_O_2_ (1 equiv), Me_3_Pyr (3 equiv), 15:15:1 MTBE/Acetone/H_2_O (0.017 M), IPR 450 nm (100% intensity) for 6 h. Structural assignments were made with additional information from gCOSY, gHSQC, and gHMBC experiments. See Supporting Information for experimental details

b Reaction performed with NHC-5 and in solvent α,α,α-trifluorotoluene (TFT).

c Yield determined by ultraperformance liquid chromatography (UPLC) analysis using mesitylene as an internal standard.

d A range of alcohols can be used for cyanation. All yields are isolated unless otherwise specified, relative stereochemistry shown; absolute configuration not determined.

Next, we explored the applicability of our methodology to pharmaceutical molecules and biomolecules (Table 2). We were excited to observe that deoxycyano analogues of saxagliptin (**40**, 58% yield) and an ibuprofen derivative (**41**, 44% yield) could be successfully synthesized, demonstrating the applicability of our method to the late-stage modification of complex, medicinally relevant molecules. Pharmaceutical molecule derivatives with electron-rich heterocycles were also well tolerated (**42**–**44**, 48–57% yield), showcasing the mild nature of our reaction conditions. Furthermore, biomolecules such as an unnatural sugar (**45**, 41% yield), hydroxyproline (**46**, 50% yield), and nucleotides (**47**, 54% yield) performed well under our reaction conditions.

**Table 2:**
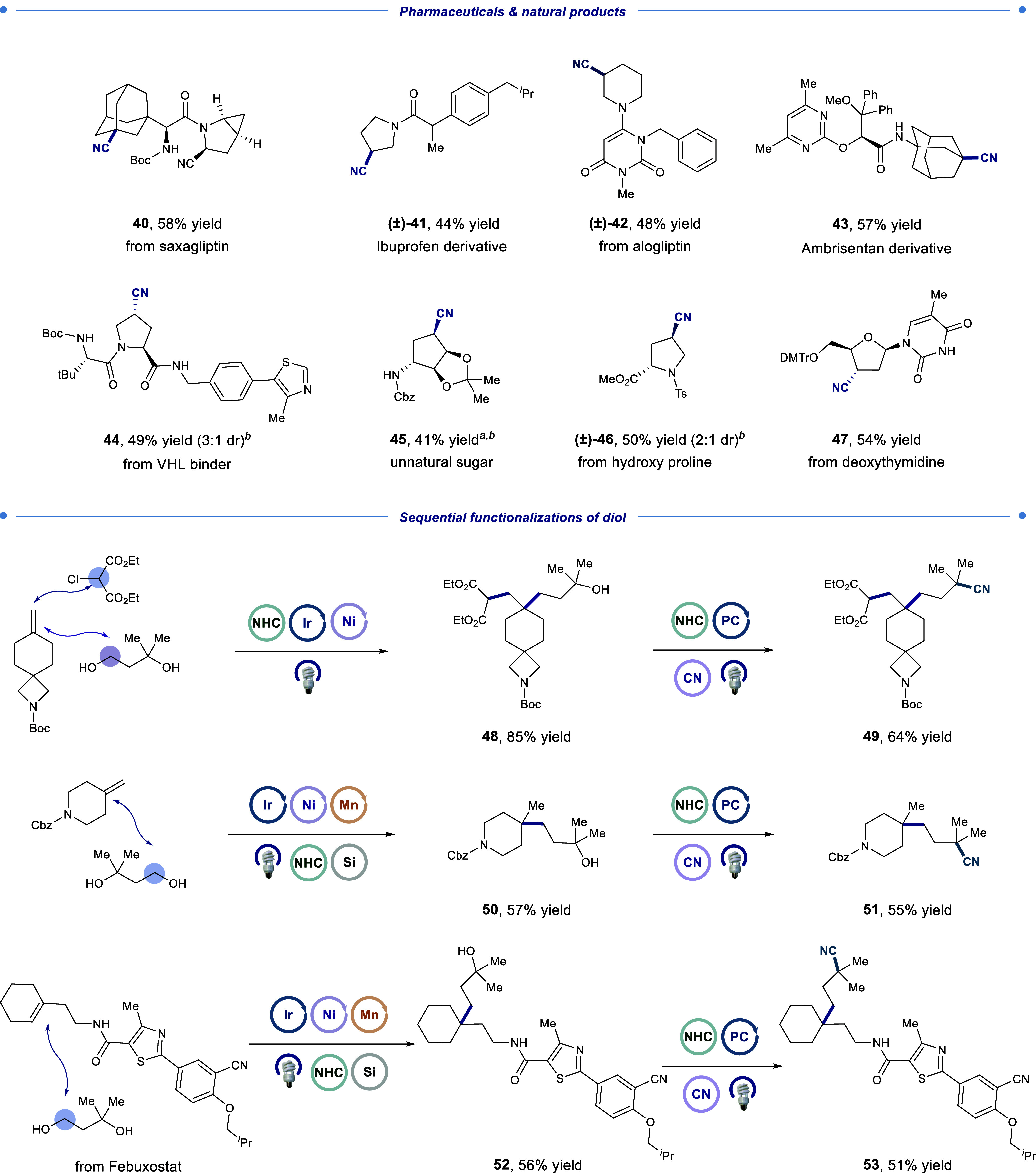
Complex Building Block Applications and Sequential Functionalization of Diols^a^

a Assay yield determined by ^1^H NMR analysis against 1,3,5-trimethoxybenzene as an internal standard.

b Relative geometry of major diastereomer shown.

c All yields are isolated unless otherwise noted. See the Supporting Information for detailed reaction conditions.

To further demonstrate the versatility of our reaction, we sought to construct complex, C­(sp^3^)-rich structures using diols in an iterative, modular fashion. Taking note of the high chemoselectivity of the NHC condensation process (1° > 2° ≫ 3°), we first subjected 3-methylbutane-1,3-diol to reactions with olefins via either alkene dialkylation^32^ (**48**) or MHAT-mediated olefin-alcohol cross-coupling^33^ (**50**, **52**), utilizing methods previously developed within our lab. As expected, olefin alkylation occurred exclusively at the primary alcohol, forming quaternary carbon centers on spirocycle, piperidine, and Febuxostat derivative substrates in good to excellent yields (56–85% yield). Each alkylated adduct possessed a tertiary alcohol that had remained untouched during the previous functionalization. These intermediates were then subjected to deoxycyanation to afford the desired tertiary alkyl nitriles (**49, 51, 53**, 51–64% yield).

In conclusion, we have developed a metal-free deoxycyanation of alcohols utilizing low-toxicity cyanide sources. This novel method demonstrates robust reactivity, excellent functional group tolerance, and the capacity to achieve late-stage functionalization. Given the importance of cyano groups in bioactive compounds, we expect that this reaction will find broad use across the synthetic community.

Note: A preprint was previously posted on ChemRxiv.^34^

## Supplementary Material



## Data Availability

The data underlying this study are available in the published article and its Supporting Information.

## Notes

Experimental details, expanded substrate table, compound characterization data, and spectra (PDF)

## References

[1] Shan Y., Zhang X., Liu G., Li J., Liu Y., Wang J., Chen D. (2024). Cyanation with Isocyanides: Recent
Advances and Perspectives. Chem. Commun..

[2] Neetha M., Afsina C. M. A., Aneeja T., Anilkumar G. (2020). Recent Advances
and Prospects in the Palladium-Catalyzed Cyanation of Aryl Halides. RSC Adv..

[3] Fleming F. F., Yao L., Ravikumar P. C., Funk L., Shook B. C. (2010). Nitrile-Containing
Pharmaceuticals: Efficacious Roles of the Nitrile Pharmacophore. J. Med. Chem..

[4] Bonatto V., Lameiro R. F., Rocho F. R., Lameira J., Leitão A., Montanari C. A. (2023). Nitriles:
An Attractive Approach to the Development
of Covalent Inhibitors. RSC Med. Chem..

[5] Silva L., Marra I., Amarante G. (2022). RECENT ADVANCES
IN CYANATION REACTIONS^†^. Quím.
Nova.

[6] Guo X., Price N. G., Zhu Q. (2024). Electrochemical
Cyanation of Alcohols
Enabled by an Iodide-Mediated Phosphine P­(V/III) Redox Couple. Org. Lett..

[7] Gaspar B., Carreira E. M. (2007). Mild Cobalt-Catalyzed
Hydrocyanation of Olefins with
Tosyl Cyanide. Angew. Chem., Int. Ed..

[8] Shaw M. H., Twilton J., MacMillan D. W. C. (2016). Photoredox Catalysis in Organic Chemistry. J. Org. Chem..

[9] Barton D. H. R., Jaszberenyi J. Cs., Theodorakis E. A. (1992). The Invention
of Radical Reactions. Part XXIII New Reactions: Nitrile and Thiocyanate
Transfer to Carbon Radicals from Sulfonyl Cyanides and Sulfonyl Isothiocyanates. Tetrahedron.

[10] Ramirez N. P., König B., Gonzalez-Gomez J. C. (2019). Decarboxylative Cyanation of Aliphatic
Carboxylic Acids via Visible-Light Flavin Photocatalysis. Org. Lett..

[11] Bao X., Wang Q., Zhu J. (2019). Dual Photoredox/Copper
Catalysis
for the Remote C­(Sp ^3^)–H Functionalization of Alcohols
and Alkyl Halides by *N* -Alkoxypyridinium Salts. Angew. Chem., Int. Ed..

[12] Kim K., Lee S., Hong S. H. (2021). Direct
C­(Sp ^3^)–H Cyanation Enabled
by a Highly Active Decatungstate Photocatalyst. Org. Lett..

[13] Patel R. I., Sharma S., Sharma A. (2021). Cyanation: A Photochemical
Approach
and Applications in Organic Synthesis. Org.
Chem. Front..

[14] Robb I., Murphy J. A. (2024). Direct, Selective α-Aryloxyalkyl Radical Cyanation
and Allylation of Aryl Alkyl Ethers. Org. Lett..

[15] Chen J., Wang P.-Z., Lu B., Liang D., Yu X.-Y., Xiao W.-J., Chen J.-R. (2019). Enantioselective Radical Ring-Opening
Cyanation of Oxime Esters by Dual Photoredox and Copper Catalysis. Org. Lett..

[16] Zhao H., Cuomo V. D., Rossi-Ashton J. A., Procter D. J. (2024). Aryl Sulfonium Salt
Electron Donor-Acceptor Complexes for Halogen Atom Transfer: Isocyanides
as Tunable Coupling Partners. Chem..

[17] Guo Q., Wang M., Peng Q., Huo Y., Liu Q., Wang R., Xu Z. (2019). Dual-Functional Chiral Cu-Catalyst-Induced
Photoredox Asymmetric Cyanofluoroalkylation of Alkenes. ACS Catal..

[18] Berger M., Ma D., Baumgartner Y., Wong T. H.-F., Melchiorre P. (2023). Stereoselective
Conjugate Cyanation of Enals by Combining Photoredox and Organocatalysis. Nat. Catal..

[19] Henkel T., Brunne R. M., Müller H., Reichel F. (1999). Statistical Investigation
into the Structural Complementarity of Natural Products and Synthetic
Compounds. Angew. Chem., Int. Ed..

[20] Ertl P., Schuhmann T. (2019). A Systematic
Cheminformatics Analysis of Functional
Groups Occurring in Natural Products. J. Nat.
Prod..

[21] Odoh A. S., Keeler C., Kim B. (2024). SuFEx-Enabled Direct
Deoxy-Diversification
of Alcohols. Org. Lett..

[22] Lee S., Kang G., Han S. (2024). Development
of an Easy-To-Handle
Redox Active Group for Alcohols: Catalytic Transformation of Tertiary
Alcohols to Nitriles. Org. Lett..

[23] Quirós I., Martín M., Pérez-Sánchez C., Rigotti T., Tortosa M. (2024). Trityl Isocyanide as a General Reagent
for Visible Light Mediated Photoredox-Catalyzed Cyanations. Chem. Sci..

[24] Dong Z., MacMillan D. W. C. (2021). Metallaphotoredox-Enabled Deoxygenative Arylation of
Alcohols. Nature.

[25] Liu Y., Li R., Yu B. (2024). Photo-Induced
Radical Transformations of Tosyl Cyanide. Org.
Biomol. Chem..

[26] Bryden M. A., Zysman-Colman E. (2021). Organic Thermally Activated Delayed Fluorescence (TADF)
Compounds Used in Photocatalysis. Chem. Soc.
Rev..

[27] Sakai H. A., MacMillan D. W. C. (2022). Nontraditional Fragment Couplings of Alcohols and Carboxylic
Acids: C­(*Sp*
^3^)–C­(*Sp*
^3^) Cross-Coupling via Radical Sorting. J. Am. Chem. Soc..

[28] Kamijo S., Hoshikawa T., Inoue M. (2011). Photochemically Induced Radical Transformation
of C­(Sp^3^)–H Bonds to C­(Sp^3^)–CN
Bonds. Org. Lett..

[29] Zhang H., Zhou Y., Tian P., Jiang C. (2019). Copper-Catalyzed Amide
Radical-Directed Cyanation of Unactivated C_sp_
^3^ – H Bonds. Org. Lett..

[30] Kong X., Wang Y., Chen Y., Chen X., Lin L., Cao Z.-Y. (2022). Cyanation and Cyanomethylation of Trimethylammonium
Salts *via* Electrochemical Cleavage of C–N
Bonds. Org. Chem. Front..

[31] Chen Y., Chen Q., Zhang S., Feng K., Xu Y.-Q., Chen X., Cao Z.-Y., Kong X. (2024). Electrochemically Driven
Denitrative Cyanation of Nitroarenes. Org. Lett..

[32] Wang J. Z., Lyon W. L., MacMillan D. W. C. (2024). Alkene Dialkylation by Triple Radical
Sorting. Nature.

[33] Cai Q., McWhinnie I. M., Dow N. W., Chan A. Y., MacMillan D. W. C. (2024). Engaging
Alkenes in Metallaphotoredox: A Triple Catalytic, Radical Sorting
Approach to Olefin-Alcohol Cross-Coupling. J.
Am. Chem. Soc..

[34] Hümpel C., Chen J., Lyon W. L., McNamee R. E., MacMillan D. W. C. (2024). Deoxycyanation
of Alkyl Alcohols. ChemRxiv.

