# Supplementary material for: Deoxycyanation of Alkyl Alcohols Using Photoredox Catalysis
Source: Org Lett. 2026 Apr 29;28(19):6096–101. doi: 10.1021/acs.orglett.6c00711 (PMC13185116; doi:10.1021/acs.orglett.6c00711)

*Supplementary Information*

# Deoxycyanation of Alkyl Alcohols Using Photoredox Catalysis

Carla Hümpel[†], William L. Lyon, Ryan McNamee, David W. C. MacMillan, Jingjia Chen*[†]

*Merck Center for Catalysis at Princeton University, Princeton, NJ 08540, USA*

[†]*These authors contributed equally to this work.*

[*]*Corresponding author. Email: chenjingjia98@gmail.com*

**Table of Contents**


**1) General information** **S3**

**2) Standard Procedures Used for Reaction Optimization** **S5**

**3) Reaction optimization** **S6**

**4) Extended Scope and Limitations** **S11**

**5) General procedures for Substrate Synthesis** **S12**

**6) Experimental and Characterization Data for Substrates** **S14**

**7) General Procedures for Deoxycyanation of Alcohols** **S20**

**8) Experimental and Characterization Data for Deoxycyanation Products** **S22**

**9) Reaction Scale-Up** **S67**

**10) References** **S68**

**11) Spectral Data for Isolated Products** **S69**

## 1) General Information

Commercial reagents were used without further purification unless otherwise indicated. All photocatalysts investigated are commercially available but are also available *via* synthetic procedures from the literature. Alcohol activation reagents, **NHC-1**, **NHC-J** and NHC-$CF_3$ (also termed **NHC-5**) were prepared according to literature procedures.[1] All organic reaction solvents were purified according to the method of Grubbs.[2] Unless indicated otherwise, filtrations of heterogeneous mixtures were performed using ChemRus 20 mL or 60 mL disposable filters. Photochemical reactions were typically irradiated using a 450 nm LED module housed within the Penn PhD Integrated Photoreactor (a standardized reactor available for purchase through Sigma Aldrich).[3] Organic solutions were concentrated under reduced pressure using a Büchi rotary evaporator with a water bath. Chromatographic purification of products was accomplished on an automated Combi-flash NextGen 300$^+$ system or an automate Biotage Isolera Four system using Silicycle SiliaSep™ and RediSep® Silver cartridges for normal-phase purifications (20 to 40 microns). Reverse phase chromatography was performed on preparative High Performance Liquid Chromatography (prepHPLC) with a Teledyne ISCO ACCQPrep® HP150 system, using a Waters XBridge BEH C18 OBD Prep Column (30 mm x 150 mm, 130 Å, 5 µm) with 0.1% ammonium hydroxide buffered water and acetonitrile solutions. Thin-layer chromatography (TLC) was performed on Analtech Uniplate 0.25 mm or Supelco 0.20 mm silica gel F-254 plates. Visualization of the developed chromatography was performed by fluorescence quenching or *via* the use of $KMnO_4$ or iodine stains.

$^1$H NMR spectra were recorded at 400 MHz or 500 MHz, using a BRUKER NanoBay Avance III HD 400 or BRUKER Avance III NMR spectrometer, respectively. $^{13}$C NMR spectra were recorded at 101 MHz or 126 MHz on a BRUKER NanoBay Avance III HD 400 or BRUKER Avance III NMR spectrometer, respectively. Chemical shifts of $^1$H NMR and $^{13}$C NMR spectra (measured at 298 K) are given in ppm by using residual solvent signals as references ($CDCl_3$: 7.26 ppm and 77.16 ppm, respectively; DMSO-*d6*: 2.50 ppm and 39.52 ppm, respectively; MeOD: 3.31 ppm and 49.00 ppm, respectively; MeCN-*d3*: 1.94 ppm and 118.26 ppm, respectively; $D_2O$: 4.79 ppm).[4] $^{19}$F NMR spectra were recorded on a BRUKER NanoBay Avance III HD 300 or BRUKER NanoBay Avance III HD 300, and are reported unreferenced. Coupling constants (J) are reported in Hertz (Hz). Standard abbreviations indicating multiplicity were used as follows: s (singlet), d

(doublet), t (triplet), q (quartet), p (pentet), s (sextet), h (septet), m (multiplet), b (broad). Apparent multiplets arising from overlapping signals are marked as virtual multiplets (*virt*). Data for $^{13}C$ NMR are reported in terms of chemical shifts.

Ultra-Performance liquid chromatography (UPLC) analysis was performed on an Agilent 1200 or Agilent 1290 Infinity II LC system. Infrared (IR) spectroscopy was performed on a Thermo Nicolet 6700 FTIR spectrometer with diamond Smart Orbit ATR accessory, and spectra are reported in wavenumbers (cm–1). High resolution mass spectra (HRMS) were obtained from the Princeton University Mass Spectral Facility on Agilent 6220 ESI-TOF LC/MS or Agilent 7200 GC-QTOF systems.

## 2) Standard Procedures Used for Reaction Optimization

Alcohol activation: An oven-dried 8-mL vial equipped with a stir bar was charged with NHC-J (1.2 equiv.) and alcohol (0.05 mmol, 1 equiv.). The vial was evacuated and backfilled with $N_2$ for three times before methyl *tert*-butyl ether (MTBE, 0.5 mL) was added via syringe. The heterogeneous solution was stirred for 5 minutes, and then pyridine (1.5 equiv.) was added dropwise. The vial was sealed with parafilm and allowed to stir for 45 minutes.

Reaction vial: An oven-dried 8-mL vial equipped with a stir bar was charged with 4-CzPN (2 mol%), tosyl cyanide (1.5 equiv.), benzoyl peroxide (1 equiv.), acetone (1.5 mL), MTBE (1 mL), $H_2O$ (0.1 mL), and stirred until the solution became homogeneous. 2,4,6-trimethylpyridine (3 equiv.) was then added.

Upon completion of the NHC condensation, the Alcohol activation solution was filtered into the Reaction vial under $N_2$ via syringe, and the Reaction vial was sparged with $N_2$ for 2 minutes in ice bath. The Reaction vial was then sealed with parafilm and subjected to IPR irradiation using 450 nm LED modules at 100% light intensity with maximum fan speed and 1000 rpm stir rate for 2 hours. The reaction vial was then opened to air and mesitylene was added as an internal standard for UPLC analysis.

*Note 1: Reaction purging must be in ice bath and should not be sparged for too long to prevent solvent evaporation.*

*Note 2: NHC condensation was typically prepared in >0.2 mmol scale to ensure reproducibility.*

*Note 3: The collidine base was typically added last, right before filtration of the NHC condensate.*

Safety Considerations**:** Tosyl cyanide is a toxic reagent and should be handled in a well-ventilated fume hood with appropriate personal protective equipment. Although it is less volatile than traditional cyanide sources, caution should be exercised to avoid exposure. Benzoyl peroxide is a strong oxidant and may present fire or explosion hazards under certain conditions; it should be handled in small quantities and kept away from heat and reducing agents. All photochemical reactions were conducted using appropriate shielding and ventilation to minimize exposure to light and heat. Standard laboratory safety protocols were followed for all reactions.

## 3) Reaction Optimization

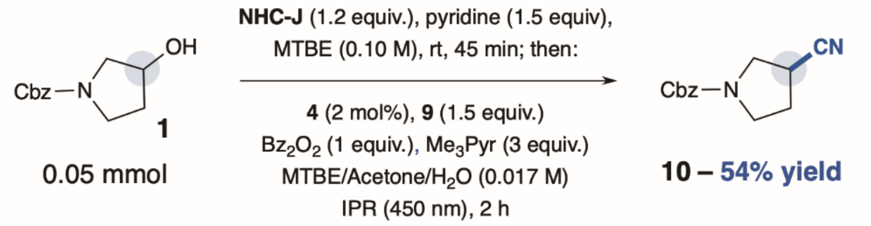


| entry | deviation | yield[b] |
|---|---|---|
| 1 | none | 54% |
| 2 | no purging | 15% |
| 3 | $O_2$ instead of $Bz_2O_2$ | 25% |
| 4 | no oxidant | 8% |
| 5 | no filtration | 41% |
| 6 | no water | 40% |
| 7 | no collidine base | 10% |
| 8 | no photocatalyst | 8% |

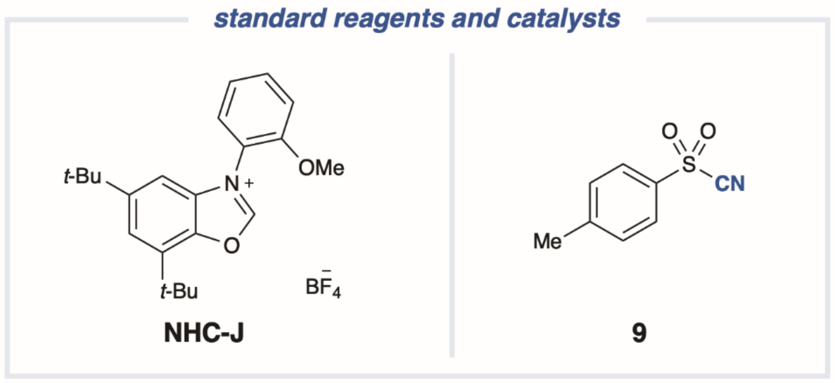


**Table S1.** Optimized Conditions and Control Reactions

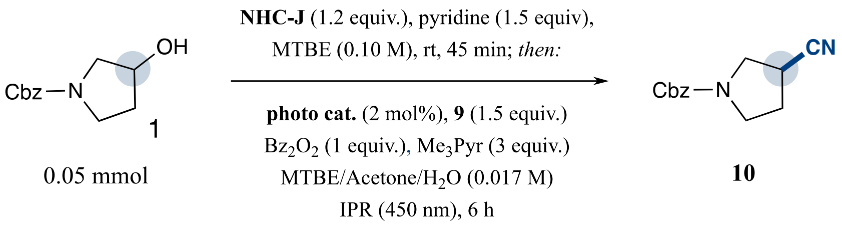


| entry | photo catalyst | yield |
|---|---|---|
| **1** | **4-CzPN** | **57%** |
| **2** | **4-CzIPN** | **60%** |
| **3** | **Ir[dF(CF$_3$)ppy]$_3$(dtbbpy)PF$_6$** | **59%** |
| 4 | Ir[F(Me)ppy]$_2$(dtbbpy)PF$_6$ | 52% |
| 5 | Ir(ppy)$_2$(dtbbpy)PF$_6$ | 51% |
| 6 | Ir(ppy)$_3$ | 43% |

**Table S2.** Optimization of photocatlysts for deoxycyanation.

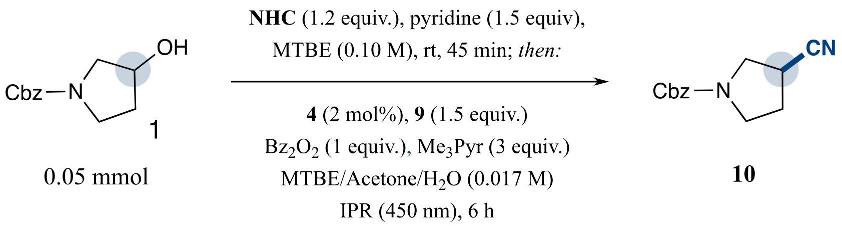


| entry | NHC | yield |
|---|---|---|
| **1** | **NHC-J** | **57%** |
| 2 | NHC-1 | 52% |
| 3 | NHC-*p*-OMe | 44% |
| 4 | NHC-5 | 10% |

**Table S3.** Optimization of NHC salts for deoxycyanation.

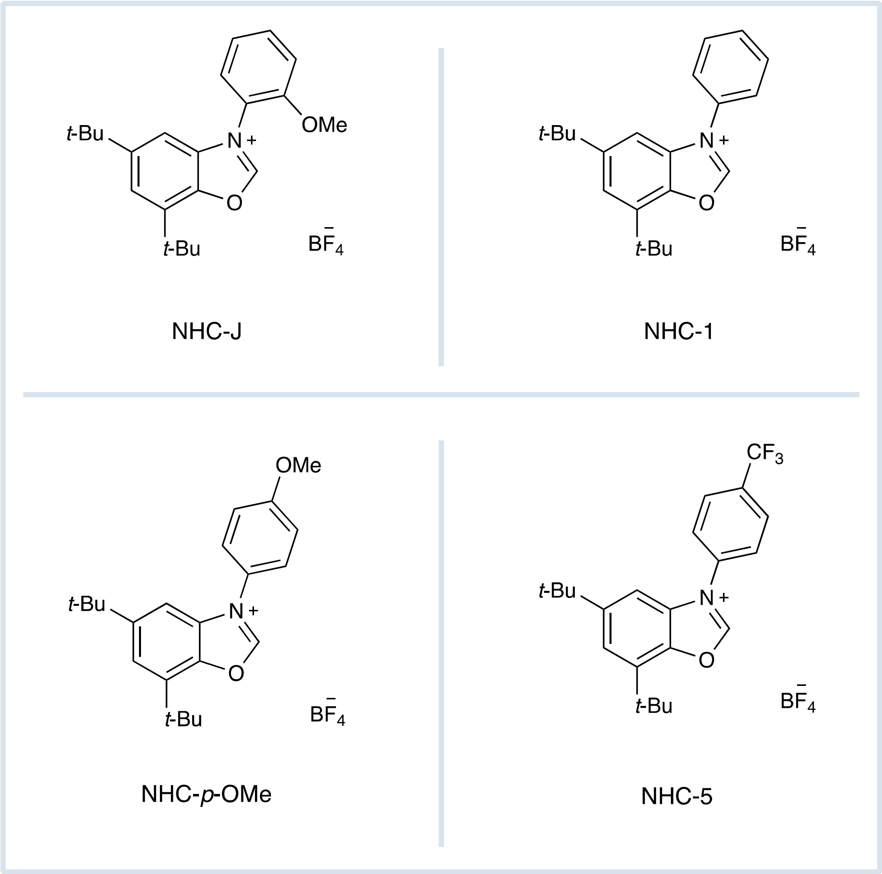


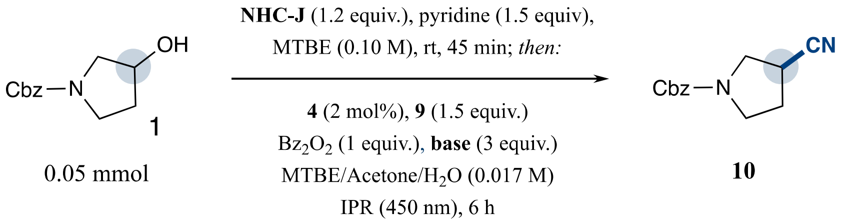


| entry | base | yield |
|---|---|---|
| **1** | **$Me_3Pyr$** | **54%** |
| **2** | **lutidine** | **55%** |
| 3 | $Cs_2CO_3$ | 47% |
| 4 | $K_2CO_3$ | 48% |
| 5 | CsOAc | 52% |
| 6 | quinuclidine | 0% |
| 7 | BTMG | 0% |

**Table S4.** Optimization of base for deoxycyanation.

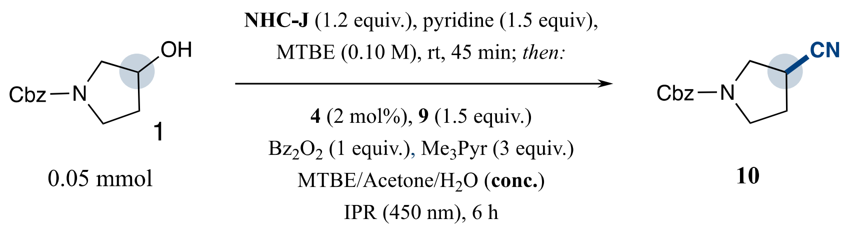


| entry | base | yield |
|---|---|---|
| 1 | 0.1 M | 25% |
| 2 | 0.05 M | 46% |
| 3 | 0.025 M | 38% |
| **4** | **0.017 M** | **59%** |

**Table S5.** Optimization of reaction concentration for deoxycyanation.

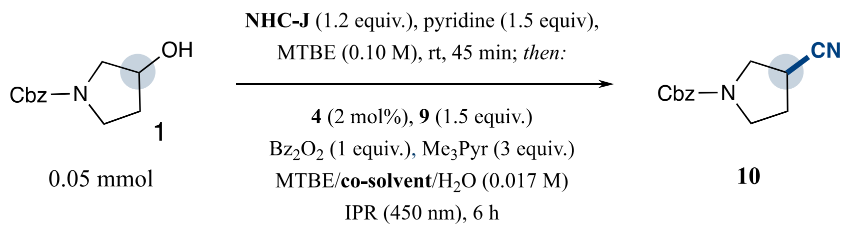


| entry | co-solvent | yield |
|---|---|---|
| **1** | **acetone** | **54%** |
| 2 | MTBE | 51% |
| 3 | DMSO | 28% |
| 4 | DMF | 47% |
| 5 | DMA | 0% |
| 6 | DCM | 5% |
| 7 | EtOAc | 0% |
| 8 | MeCN | 0% |
| 9 | dioxane | 49% |
| 10 | THF | 35% |
| **11** | **DME** | **56%** |
| 12 | DMC | 46% |

**Table S6.** Optimization of reaction solvent for deoxycyanation.

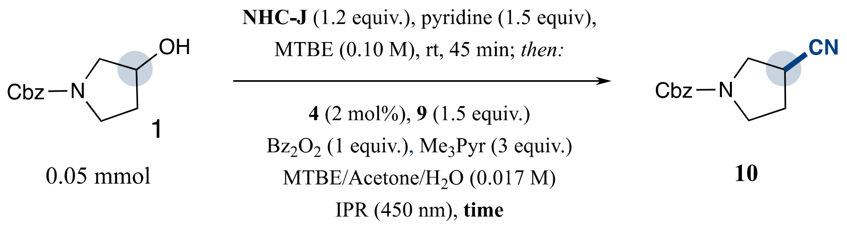


| entry | time | yield |
|---|---|---|
| 1 | 5 min | 38% |
| 2 | 10 min | 50% |
| 3 | 20 min | 54% |
| 4 | 30 min | 54% |
| 5 | 1 h | 55% |
| 6 | 2 h | 56% |
| 7 | 4 h | 53% |
| 8 | 6 h | 58% |

**Table S7.** Time study for deoxycyanation.

# 4) Extended Scope and Limitations

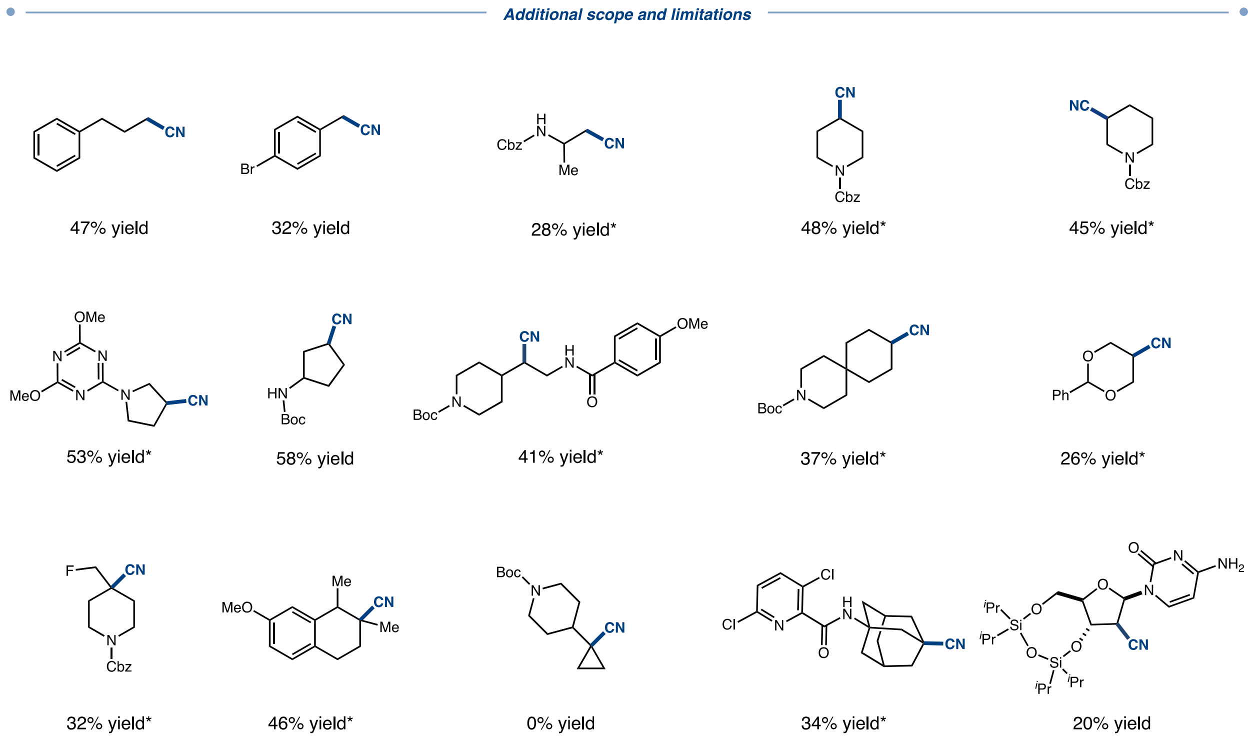


**Figure S1.** Extended substrate scope and limitations. Preliminary assay yields determined by UPLC-MS analysis at 195 nm UV absorption based on based on starting material extinction coefficient vs. mesitylene as an internal standard. (*Isolated yields are given)

**Substrate limitations:** (1) Benzylic alcohols generally perform poorly in the reaction. (2) Alcohols with nearby electron-withdrawing groups such as trifluoromethyl group often show poor reactivity, likely a result of either sluggish NHC condensation yields due to attenuated nucleophilicity of the alcohols or the electrophilic nature of the corresponding deoxygenated radicals. (3) Alcohols that are very sterically encumbered and have poor nucleophilicity are not tolerated.

## 5) General Procedures for Substrate Synthesis

### General procedure S1 – Grignard addition

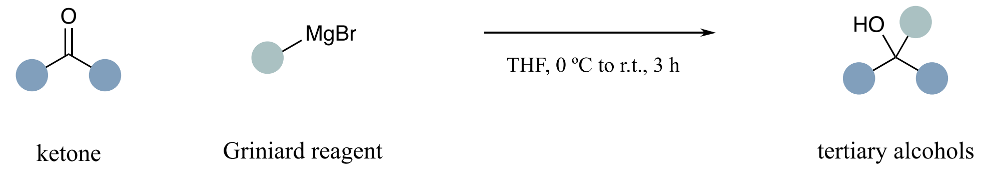


An oven-dried 100 mL Schlenk flask equipped with a stir bar was sealed with a septum, vacuumed and backfilled with $N_2$ for three times. The Grignard reagent (2.0 equiv.) and THF solvent (5 mL) were added to the flask via syringe. The solution was cooled down to 0 °C and a solution of the ketone (1.0 equiv.) in THF (4 mL) was added dropwise at 0 °C while stirring. The reaction mixture was then allowed to warm up to room temperature and stirred for 3 hours. The reaction was then quenched by pouring the mixture into 100 mL sat. $NH_4Cl$-solution. The aqueous layer was extracted with EtOAc (3×100 mL). The combined organic layers were dried over anhydrous sodium sulfate, filtered, and concentrated. The crude was purified by column chromatography.

### General procedure S2 – Boc-protection

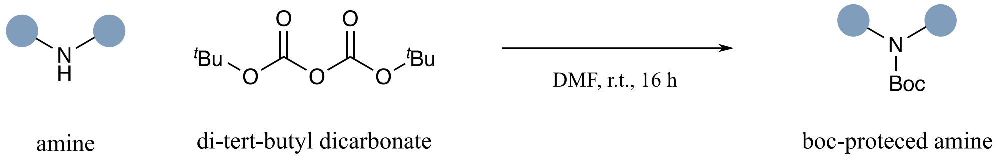


To an oven-dried 250 mL round bottom flask charged with a stir bar, the amine (1.0 equiv.) and di-tert-butyl dicarbonate (1.2 equiv.) was dissolved in DMF (10 mL). The reaction mixture was stirred at room temperature for overnight. The crude mixture was then diluted with EtOAc (100 mL) and the organic layers washed with 5% LiCl solution, $H_2O$, and brine. The combined organic layers were dried over anhydrous sodium sulfate, filtered, and concentrated. The resulting solid/oil was then either purified by recrystallisation or column chromatography.

### General procedure S3 – Amide coupling

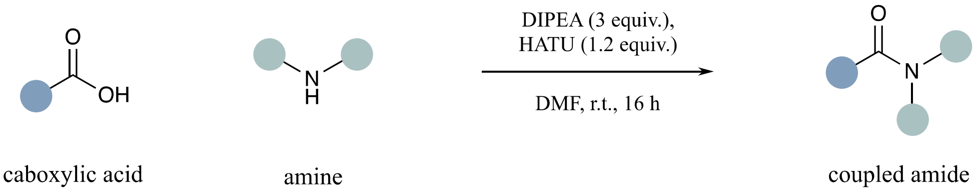


To an oven-dried 250 mL round bottom flask charged with a stir bar, the carboxylic acid (1.0 equiv.), amine (1.2 equiv.), and HATU (1.2 equiv.) was dissolved in DMF (20 mL), and then DIPEA (3 equiv.) was added into the reaction flask. The reaction mixture was stirred at room temperature for overnight. The crude mixture was then diluted with EtOAc (100 mL), washed with 5% LiCl solution, $H_2O$, and brine. The combined organic layers were dried over anhydrous sodium sulfate, filtered, and concentrated. The resulting solid was then purified by column chromatography.

## 6) Experimental and Characterization Data for Substrates

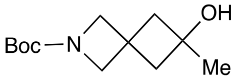


***Tert*-butyl 6-hydroxy-6-methyl-2-azaspiro[3.3]heptane-2-carboxylate (S1)**

The title compound was synthesized according to **General procedure S1**. *Tert*-butyl 6-oxo-2-azaspiro[3.3]heptane-2-carboxylate (1.5 g, 7.10 mmol, 1.0 equiv.) and methylmagnesium bromide (3.0 M solution in $Et_2O$, 4.47 mL, 2.0 equiv., 14.2 mmol) were used to generate the desired product. The crude reaction mixture was purified by automated flash chromatography (40 g high performance silica column, 0–100% EtOAc in hexane) to yield the title compound (1.33 g, 5.87 mmol, 82.7%) as a white solid.

**$^1$H NMR (400 MHz, $CDCl_3$)** δ 3.89 (d, $J$ = 11.4 Hz, 4H), 2.25 (s, 4H), 1.89 (s, 1H), 1.42 (s, 9H), 1.30 (s, 3H).

**$^{13}$C NMR (126 MHz, $CDCl_3$)** δ 156.3, 79.5, 68.8, 48.4, 28.7, 28.5, 28.4, 28.1.

**IR (film)** $\nu_{max}$ 3400, 2964, 2928, 2873, 1653, 1532, 1475, 1424, 1392, 1364, 1315, 1273, 1253, 1210, 1192, 1149, 1131, 1075, 963, 943, 933, 897, 856, 765, 635, 586, 532, 473, 446.

**HRMS (ESI-TOF)** *m/z* calcd. for $C_{12}H_{21}NO_3Na^+$ ($[M+Na]^+$) 250.1414, found 250.1414.

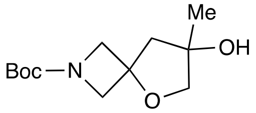


***(±)-Tert*-butyl 7-hydroxy-7-methyl-5-oxa-2-azaspiro[3.4]octane-2-carboxylate (S2)**

The title compound was synthesized according to **General procedure S1**. *Tert*-butyl 7-oxo-5-oxa-2-azaspiro[3.4]octane-2-carboxylate (1.0 g, 4.4 mmol, 1.0 equiv.) and methylmagnesium bromide (3.0 M solution in $Et_2O$, 2.93 mL, 2.0 equiv., 8.80 mmol) were used to generate the desired product. The crude reaction mixture was purified by automated flash chromatography (40 g high performance silica column, 0-100% EtOAc in hexane) to yield the title compound (747 mg, 3.07 mmol, 69.8%) as a slightly yellow oil.

**$^1$H NMR (500 MHz, CDCl$_3$)** δ 4.07 (d, *J* = 9.3 Hz, 1H), 4.03 (d, *J* = 1.5 Hz, 2H), 3.91 (d, *J* = 9.3 Hz, 1H), 3.80 (d, *J* = 9.2 Hz, 1H), 3.68 (d, *J* = 9.2 Hz, 1H), 2.30 (d, *J* = 13.3 Hz, 1H), 2.06 (d, *J* = 13.3 Hz, 1H), 1.82 (s, 1H), 1.42 (s, 9H), 1.40 (s, 3H).

**$^{13}$C NMR (126 MHz, CDCl$_3$)** δ 156.5, 80.2, 79.7, 79.0, 78.9, 50.3, 28.5, 24.7.

**IR (film)** $\nu_{max}$ 3382, 2976, 2939, 2872, 1682, 1522, 1496, 1392, 1368, 1326, 1303, 1288, 1238, 1176, 1158, 1112, 1073, 1053, 998, 973, 949, 932, 883, 867, 829, 811, 794, 769, 738, 725, 637, 613, 583, 531, 485, 459, 441.

**HRMS (ESI-TOF)** *m/z* calcd. for $C_{12}H_{21}NO_4Na^+$ ($[M+Na]^+$) 266.1363, found 266.1362.

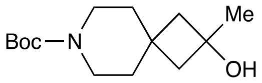


***Tert*-butyl 2-hydroxy-2-methyl-7-azaspiro[3.5]nonane-7-carboxylate (S3)**

The title compound was synthesized according to **General procedure S1**. *Tert*-butyl 2-oxo-7-azaspiro[3.5]nonane-7-carboxylate (2.0 g, 8.36 mmol, 1.0 equiv.) and methylmagnesium bromide (3.0 M solution in $Et_2O$, 5.57 mL, 2.0 equiv., 16.71 mmol) were used to generate the desired product. The crude reaction mixture was purified by automated flash chromatography (40 g silica column, 0-30% EtOAc in hexane) to yield the title compound (1.66 g, 6.50 mmol, 77.8%) as a white solid.

Spectral data are consistent with those reported in literature: Aguilar Troyano, F. J.; Ballaschk, F.; Jaschinski, M.; Özkaya, Y.; Gómez-Suárez, A. *Chem. Eur. J.,* **2019**, *25*, 14054 – 14058.

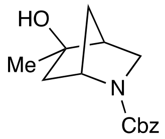


**Benzyl 5-hydroxy-5-methyl-2-azabicyclo[2.2.1]heptane-2-carboxylate (S4)**

The title compound was synthesized according to **General procedure S1**. Benzyl 5-oxo-2-azabicyclo[2.2.1]heptane-2-carboxylate (1.0 g, 4.08 mmol, 1.0 equiv.), methylmagnesium bromide (3.0 M solution in $Et_2O$, 2.72 mL, 2.0 equiv., 8.15 mmol) were used to generate the

desired product. The crude reaction mixture was purified by automated flash chromatography (40 g high performance silica column, 0-100% EtOAc in hexane) to yield the title compound (820 mg, 43.14 mmol, 77.0%) as a white solid.

**$^1$H NMR (500 MHz, CDCl$_3$)** δ 7.39 – 7.27 (m, 5H), 5.13 (d, *J* = 13.1 Hz, 2H), 4.23 (d, *J* = 22.3 Hz, 1H), 3.85 (dd, *J* = 9.8, 1.6 Hz, 1H), 3.18 (dd, *J* = 9.9, 3.3 Hz, 1H), 2.35 – 2.30 (m, 1H), 1.77 – 1.62 (m, 5H), 1.40 (s, 3H).

**$^{13}$C NMR (126 MHz, CDCl$_3$)** δ 154.8, 137.2, 128.6, 127.9, 75.5, 66.6, 57.6, 49.1, 48.7, 47.8, 47.3, 46.2, 38.2, 37.8, 30.3.

**IR (film)** $\nu_{max}$ 3410, 2967, 2884, 1672, 1498, 1419, 1358, 1329, 1299, 1262, 1208, 1171, 1095, 1069, 1027, 1000, 945, 916, 884, 859, 812, 793, 733, 696, 591, 548, 451.

**HRMS (ESI-TOF)** *m/z* calcd. for $C_{15}H_{19}NO_3Na^+$ ($[M+Na]^+$) 284.1257, found 284.1258.

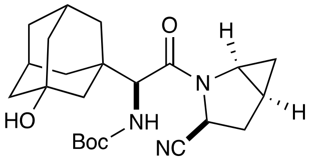


**_Tert_-butyl ((_S_)-2-((1_S_,3_S_,5_S_)-3-cyano-2-azabicyclo[3.1.0]hexan-2-yl)-1-((1_r_,3_R_,5_R_,7_S_)-3-hydroxyadamantan-1-yl)-2-oxoethyl)carbamate (S5)**

The title compound was synthesized according to **General procedure S2**. *tert*-butyl ((*S*)-2-((1*S*,3*S*,5*S*)-3-cyano-2-azabicyclo[3.1.0]hexan-2-yl)-1-((1*r*,3*R*,5*R*,7*S*)-3-hydroxyadamantan-1-yl)-2-oxoethyl)carbamate (0.8 g, 2.54 mmol, 1.0 equiv.), di-tert-butyl dicarbonate (664.3 mg, 1.2 equiv., 3.04 mmol) were used to generate the desired product. The crude reaction mixture was purified by automated flash chromatography (40 g high performance silica column, 0-100% EtOAc in hexane) to yield the title compound (1.03 g, 2.48 mmol, 97.7%) as a white solid.

**$^1$H NMR (500 MHz, CDCl$_3$)** δ 5.29 (d, *J* = 9.9 Hz, 1H), 5.04 (dd, *J* = 10.6, 2.3 Hz, 1H), 4.46 (d, *J* = 9.9 Hz, 1H), 3.91 – 3.71 (m, 1H), 2.57 (ddd, *J* = 13.6, 10.6, 5.8 Hz, 1H), 2.37 (dd, *J* = 13.7, 2.4 Hz, 1H), 2.25 (p, *J* = 3.1 Hz, 2H), 1.94 – 1.85 (m, 1H), 1.79 (dt, *J* = 11.5, 2.5 Hz, 1H), 1.76 – 1.58 (m, 9H), 1.53 – 1.44 (m, 3H), 1.43 (s, 9H), 1.14 – 0.99 (m, 2H).

**$^{13}$C NMR (126 MHz, CDCl$_3$)** δ 170.1, 156.0, 119.4, 80.1, 68.7, 58.8, 46.5, 45.2, 44.6, 44.4, 41.3, 38.2, 37.7, 37.2, 35.3, 30.6, 30.3, 28.5, 18.0, 13.7.

**IR (film)** $\nu_{max}$ 3376, 2904, 2851, 2176, 1703, 1693, 1676, 1650, 1625, 1522, 1501, 1453, 1427, 1366, 1315, 1246, 1160, 1128, 530, 478.

**HRMS (ESI-TOF)** *m/z* calcd. for $C_{23}H_{34}N_3O_4^+$ ($[M+H]^+$) 416.2544, found 416.2547.

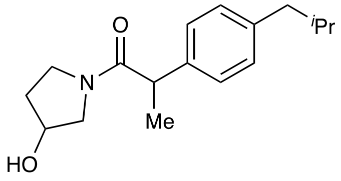


**1-(3-hydroxypyrrolidin-1-yl)-2-(4-isobutylphenyl)propan-1-one** (**S6**)

The title compound was synthesized according to **General procedure S3**. (S)-3-hydroxypyrrolidine hydrochloride (588.4 mg, 1.2 equiv., 4.80 mmol,), 2-(4-isobutylphenyl)propanoic acid (825.1 mg, 1.0 equiv., 4.0 mmol), HATU (1.83 g, 1.2 quiv., 4.80 mmol), DIPEA (2.09 ml, 3 equiv., 12.0 mmol) were used to generate the desired product. The crude reaction mixture was purified by automated flash chromatography (40 g high performance silica column, 0-100% EtOAc in hexane) to yield the title compound (890 mg, 3.24 mmol, 80.9%) as a white solid.

**$^1$H NMR (500 MHz, CDCl$_3$)** δ 7.26 – 7.12 (m, 2H), 7.09 (dd, *J* = 8.1, 2.9 Hz, 2H), 4.51 – 4.33 (m, 1H), 3.84 – 3.41 (m, 4H), 3.42 – 3.15 (m, 1H), 2.82 (s, 1H), 2.45 (ddd, *J* = 7.3, 3.5, 1.8 Hz, 2H), 2.07 – 1.75 (m, 3H), 1.44 (ddd, *J* = 9.1, 6.2, 4.0 Hz, 3H), 0.90 (dd, *J* = 6.7, 3.4 Hz, 6H). **(summary of rotamers and diastereomers)**

**$^{13}$C NMR (126 MHz, CDCl$_3$)** δ 173.2, 173.04, 172.97, 140.38, 140.36, 140.3, 138.8, 138.7, 138.61, 138.56, 129.64, 129.62, 129.61, 129.59, 127.4, 127.31, 127.30, 71.28, 71.27, 71.2, 69.51, 69.49, 69.41, 69.39, 54.74, 54.68, 54.5, 45.2, 44.9, 44.63, 44.59, 44.50, 44.46, 44.2, 44.1, 44.0, 38.8, 34.4, 34.3, 33.0, 32.8, 30.30, 30.28, 22.54, 22.52, 20.4, 20.3, 20.24, 20.16. **(Summary of rotamers and diastereomers.)**

**IR (film)** $\nu_{max}$ 3303, 2949, 2866, 1604, 1440, 1341, 1240, 1104, 985, 871, 848, 706, 683, 549.

**HRMS (ESI-TOF)** *m/z* calcd. for $C_{17}H_{26}NO_2^+$ ($[M+H]^+$) 276.1958, found 276.1959.

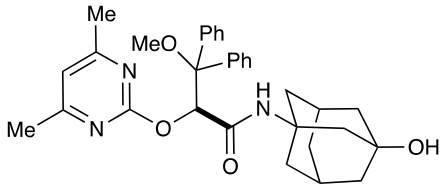


**(*S*)-2-((4,6-dimethylpyrimidin-2-yl)oxy)-*N*-((1*r*,3*R*,5*R*,7*S*)-3-hydroxyadamantan-1-yl)-3-methoxy-3,3-diphenylpropanamide (S7)**

The title compound was synthesized according to **General procedure S3**. 3-aminoadamantan-1-ol (250.9 mg, 1.5 equiv., 1.50 mmol,), Ambrisentan (278.4 mg, 1.0 equiv., 1.0 mmol), HATU (570.3 mg, 1.5 quiv., 1.5 mmol), DIPEA (0.87 ml, 5 equiv., 5.0 mmol) were used to generate the desired product. The crude reaction mixture was purified by automated flash chromatography (40 g high performance silica column, 0-100% EtOAc in hexane) to yield the title compound (324 mg, 0.61 mmol, 61.4%) as a light yellow powder.

**$^1$H NMR (500 MHz, CDCl$_3$)** δ 7.65 – 7.56 (m, 2H), 7.39 – 7.31 (m, 4H), 7.31 – 7.26 (m, 2H), 7.26 – 7.20 (m, 2H), 6.72 (s, 1H), 6.27 (s, 1H), 5.11 (s, 1H), 3.24 (s, 3H), 2.42 (s, 6H), 1.79 – 1.32 (m, 14H).

**$^{13}$C NMR (126 MHz, CDCl$_3$)** δ 169.6, 166.8, 163.7, 141.2, 140.9, 129.2, 129.1, 128.0, 127.8, 127.7, 127.6, 115.2, 84.2, 78.0, 69.2, 53.7, 52.7, 48.6, 44.1, 39.8, 39.7, 34.9, 30.6, 24.0.

**IR (film)** $\nu_{max}$ 2909, 2851, 1678, 1594, 1456, 1445, 1395, 1361, 1096, 1076, 1033, 749, 699, 552, 534.

**HRMS (ESI-TOF)** *m/z* calcd. for $C_{32}H_{38}N_3O_4^+$ ($[M+H]^+$) 528.2857, found 528.2859.

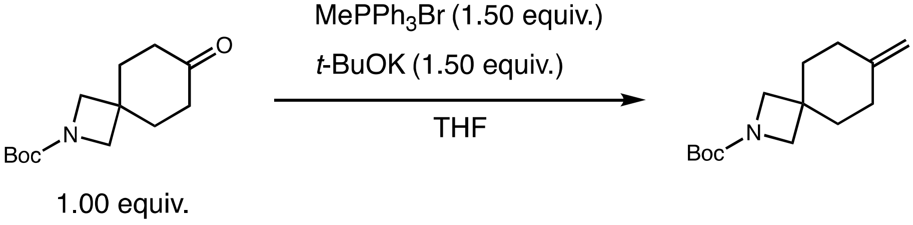

***Tert*-butyl 7-methylene-2-azaspiro[3.5]nonane-2-carboxylate (S8)**

To a flame-dried 250 mL round-bottom flask was added $MePPh_3Br$ (2.24 g, 6.27 mmol, 1.50 equiv.), and THF (20 mL). The solution was cooled to 0 ºC and potassium *tert*-butoxide (703 mg, 6.27 mmol, 1.50 equiv.) was added portionwise. The reaction was then stirred for 1 hour at room temperature, under nitrogen atmosphere. Next, the reaction mixture was cooled to 0 ºC and a solution of *tert*-butyl 7-oxo-2-azaspiro[3.5]nonane-2-carboxylate (1.00 g, 4.18 mmol, 1.00 equiv.) in 20 mL THF was added dropwise. The reaction was stirred for 16 hours at room temperature and then quenched by addition of saturated aqueous $NH_4Cl$ solution (50 mL). The quenched reaction mixture was transferred to a separatory funnel, and the organic layer was collected. The aqueous layer was extracted with DCM (2 × 50 mL), and all organic layers were combined, dried with $Na_2SO_4$, filtered, concentrated by rotary evaporation, and purified by automated flash chromatography (40 g silica column, 0-10% EtOAc/hexanes gradient), followed by automated flash chromatography (50 g silica column, 0-2% EtOAc/DCM gradient) to afford the title compound as a white solid (727.2 mg, 3.06 mmol, 73.3% yield).

**$^1$H NMR (500 MHz, $CDCl_3$)** δ 4.64 (s, 2H), 3.62 (d, $J$ = 1.1 Hz, 4H), 2.14 – 2.07 (m, 4H), 1.74 – 1.70 (m, 4H), 1.44 (d, $J$ = 1.1 Hz, 9H).

**$^{13}$C NMR (126 MHz, $CDCl_3$)** δ 156.7, 147.4, 108.1, 79.4, 59.1, 37.2, 34.9, 31.4, 28.6.

**IR (film)** $\nu_{max}$ 3075, 3001, 2975, 2928, 2873, 2847, 1694, 1645, 1544, 1476, 1441, 1436, 1403, 1364, 1349, 1314, 1253, 1167, 1139, 1104, 1079, 1001, 962, 928, 877, 861, 811, 770, 757, 683, 647, 570, 502, 463, 420 $cm^{-1}$.

**HRMS (ESI-TOF)** *m/z* calcd. for $C_9H_{16}N+$ ($[M+H-Boc]^+$), 138.1277 found 138.1277.

## 7) General Procedures for Deoxycyanation of Alcohols

### General procedure A – 1°/2° alcohols

<u>Alcohol Activation:</u> To an oven-dried 40 mL vial equipped with a stir bar, NHC-J (255.2 mg, 0.6 mmol, 1.2 equiv.) and the alcohol (0.5 mmol, 1.0 equiv., if solid) were added. The vial was

vacuumed and backfilled with $N_2$ for three times before alcohol (if liquid) and MTBE (5 mL) was added. The reaction mixture was stirred for 5 minutes under nitrogen, and then pyridine (60.4 μL, 0.75 mmol, 1.5 equiv.) was added dropwise via syringe. The vial was then sealed with parafilm and allowed to stir for 45 minutes. Another 10 mL of MTBE was added to rinse out the condensation vial.

Reaction Vial: To an oven-dried 40 mL vial was equipped with a stir bar, 4-CzPN (7.9 mg, 1 μmol, 2 mol%), tosyl cyanide (135.9 mg, 0.75 mmol, 1.5 equiv.), and benzoyl peroxide (121.1 mg, 0.5 mmol, 1 equiv.), 15 mL acetone, and 1 mL of $H_2O$ were added, and the resulting mixture was sonicated for a few minutes until all solids were dissolved. Then 2,4,6-trimethylpyridine (198.4 μL, 1.5 mmol, 3 equiv.) was added.

Upon completion of the NHC condensation, the solution in the Alcohol Activation vial was then syringe filtered into the Reaction Vial under $N_2$ atmosphere. The reaction mixture was sparged with nitrogen for 5 minutes in ice bath, sealed with parafilm, and irradiated with 450 nm LED modules at 100% light intensity with maximum fan speed and 1000 rpm stir rate in a PennPhD Integrated Photoreactor for 6 hours. After this time, mesitylene (69.6 μL, 1 equiv.) was added as an internal standard, and an aliquot was removed for UPLC-MS analysis.

The reaction was concentrated under reduced pressure and the crude mixture was subjected to automated column chromatography followed by preparative HPLC (if necessary) to afford the pure cyanation product. Structural assignments were made with additional information from gCOSY, gHSQC, and gHMBC experiments.

*Note:* A wash using $NaHCO_3$ could help remove the benzoic acid byproduct in the reaction and avoid streaking on the column during chromatography.

**General procedure B – 3° alcohols**

Alcohol Activation: To an oven-dried 40 mL vial equipped with a stir bar, NHC-5 (277.9 mg, 0.6 mmol, 1.2 equiv.) and the alcohol (0.5 mmol, 1.0 equiv.) were added. The vial was vacuumed and

backfilled with $N_2$ for three times before dry $\alpha$, $\alpha$, $\alpha$-trifluorotoluene (5 mL) was added. The reaction mixture was stirred for 5 minutes under nitrogen, and then pyridine (60.4 µL, 0.75 mmol, 1.5 equiv.) was added dropwise via syringe at –25 ºC. The vial was then sealed with parafilm and allowed to stir for 2 hours while slowly warming up to room temperature. Another 10 mL of $\alpha$, $\alpha$, $\alpha$-trifluorotoluene was added to rinse out the condensation vial.

Reaction Vial: To an oven-dried 40 mL vial was equipped with a stir bar, 4-CzPN (7.9 mg, 1 µmol, 2 mol%), tosyl cyanide (135.9 mg, 0.75 mmol, 1.5 equiv.), and benzoyl peroxide (121.1 mg, 0.5 mmol, 1 equiv.), 15 mL acetone, and 1 mL of $H_2O$ were added, and the resulting mixture was sonicated for a few minutes until all solids were dissolved. Then 2,4,6-trimethylpyridine (198.4 µL, 1.5 mmol, 3 equiv.) was added.

Upon completion of the NHC condensation, the solution in the Alcohol Activation vial was then syringe filtered into the Reaction Vial under $N_2$ atmosphere. The reaction mixture was sparged with nitrogen for 5 minutes in ice bath, sealed with parafilm, and irradiated with 450 nm LED modules at 100% light intensity with maximum fan speed and 1000 rpm stir rate in a PennPhD Integrated Photoreactor for 6 hours. After this time, mesitylene (69.6 µL, 1 equiv.) was added as an internal standard, and an aliquot was removed for UPLC-MS analysis.

The reaction was concentrated under reduced pressure and the crude mixture was subjected to automated column chromatography followed by preparative HPLC (if necessary) to afford the pure cyanation product. Structural assignments were made with additional information from gCOSY, gHSQC, and gHMBC experiments.

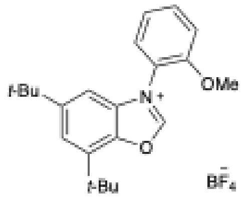


NHC-J

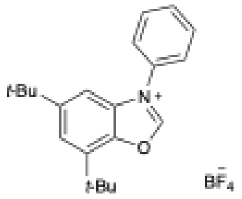


NHC-1

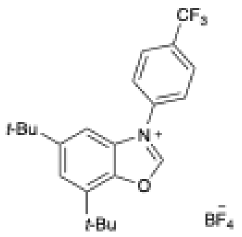


NHC-5

## 8) Experimental and Characterization Data for Deoxycyanation Products

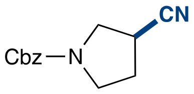

**(±)-Benzyl-3-cyanopyrrolidine-1-carboxylate (10)**

The title compound was prepared according to **General procedure A**. 4-CzPN (7.9 mg, 0.01 mmol, 2 mol%), tosyl cyanide (135.9 mg, 0.75 mmol, 1.5 equiv.), benzoyl peroxide (121.1 mg, 0.5 mmol, 1.0 equiv.), 2,4,6-trimethylpyridine (198 µL, 1.5 mmol, 3.0 equiv.), acetone (15 mL) and $H_2O$ (1 mL) were added directly to the reaction vial. From activated alcohol solution: benzyl 3-hydroxypyrrolidine-1-carboxylate (110.6 mg, 0.5 mmol, 1.0 equiv.), NHC-J (255.2 mg, 0.6 mmol, 1.2 equiv.), MTBE (15 mL) and pyridine (60 µL, 0.75 mmol, 1.5 equiv.). The residue was purified by automated flash chromatography (40 g high performance silica column, 0-100% EtOAc in hexane). The product containing fractions were then combined and after a second purification via preparative HPLC (XBridge BEH C18 OBD column, 20-100% MeCN in water (0.1% $NH_4OH$ modifier), the title compound (69.1 mg, 0.30 mmol, 59.1%) was obtained as a slightly yellow oil.

**$^{1}$H** and **$^{13}$C NMR** spectroscopic data is consistent with that of reported in the literature: Winkler, M.; Meischler, D.; Klempier, N. *Adv. Synth. Catal.* **2007**, *349*, 1475-1480.

**$^{1}$H NMR (500 MHz, $CDCl_3$)** $\delta$ 7.40 – 7.30 (m, 5H), 5.14 (d, $J$ = 3.3 Hz, 2H), 3.80 – 3.46 (m, 4H), 3.11 (h, $J$ = 6.5 Hz, 1H), 2.25 (dtt, $J$ = 19.9, 13.1, 6.0 Hz, 2H).

**$^{13}$C NMR (126 MHz, $CDCl_3$)** δ 154.5, 154.3, 136.5, 136.4, 128.7, 128.33, 128.31, 128.20, 128.16, 119.9, 119.8, 67.37, 67.35, 49.3, 48.8, 45.0, 44.6, 30.3, 29.4, 28.5, 27.8 **(Summary of rotamers.)**

**IR (film)** $\nu_{max}$ 2956, 2866, 2244, 1695, 1586, 1537, 1447, 1413, 1359, 1340, 1246, 1212, 1167, 1111, 1029, 985, 913, 881, 825, 766, 768, 696, 606, 569, 542, 508, 456.

**HRMS (ESI-TOF)** *m/z* calcd. for $C_{13}H_{14}N_2O_2Na^+$ ($[M+Na]^+$) 253.0948, found 253.0949.

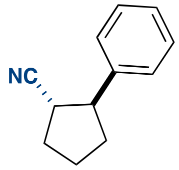


**2-phenylcyclopentane-1-carbonitrile (11)**

The title compound was prepared according to **General procedure A**. 4-CzPN (7.9 mg, 0.01 mmol, 2 mol%), tosyl cyanide (135.9 mg, 0.75 mmol, 1.5 equiv.), benzoyl peroxide (121.1 mg, 0.5 mmol, 1.0 equiv.), 2,4,6-trimethylpyridine (198 µL, 1.5 mmol, 3.0 equiv.), acetone (15 mL) and $H_2O$ (1 mL) were added directly to the reaction vial. From activated alcohol solution: (1*S*)-(2*R*)-phenylcyclopentan-1-ol (81.1 mg, 0.5 mmol, 1.0 equiv.), NHC-J (255.2 mg, 0.6 mmol, 1.2 equiv.), MTBE (15 mL) and pyridine (60 µL, 0.75 mmol, 1.5 equiv.). The residue was purified by automated flash chromatography (40 g high performance silica column, 0-100% EtOAc in hexane). The product containing fractions were then combined and after a second purification via preparative HPLC (XBridge BEH C18 OBD column, 20-100% MeCN in water (0.1% $NH_4OH$ modifier), the title compound (55.2 mg, 0.32 mmol, 64.5%) was obtained as a slightly yellow liquid as a single diastereomer.

The spectral data corresponds to the *trans* diastereomer: Dzienchciejewski, W. J.; Weber, R.; Sowada, O.; Boysen, M. M. K. *Org. Lett.* **2015**, *17*, 4132–4135.

**$^1$H NMR (500 MHz, $CDCl_3$)** $\delta$ 7.32 (dd, $J$ = 8.2, 6.9 Hz, 2H), 7.28 – 7.21 (m, 3H), 3.26 (td, $J$ = 10.0, 7.6 Hz, 1H), 2.70 (q, $J$ = 8.9 Hz, 1H), 2.31 – 2.13 (m, 2H), 2.05 (dtd, $J$ = 13.1, 8.6, 7.0 Hz, 1H), 1.95 – 1.73 (m, 3H).

**$^{13}$C NMR (126 MHz, $CDCl_3$)** $\delta$ 141.3, 129.0, 127.4, 127.1, 122.6, 51.2, 36.8, 33.8, 31.2, 24.7.

**IR (film)** $\nu_{max}$ 3029, 2959, 2875, 2237, 1602, 1495, 1452, 1152, 1030, 753, 698, 558, 520, 494.

**HRMS (GC/Q-TOF)** *m/z* calcd. for $C_{12}H_{13}N^+$ ($[M]^+$) 171.1043, found 171.1044.

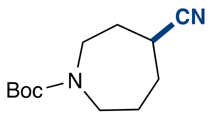


**(±)-*Tert*-butyl-4-cyanoazepane-1-carboxylate (12)**

The title compound was prepared according to a modified **General procedure A**. 4-CzPN (7.9 mg, 0.01 mmol, 2 mol%), tosyl cyanide (135.9 mg, 0.75 mmol, 1.5 equiv.), benzoyl peroxide (121.1 mg, 0.5 mmol, 1.0 equiv.), 2,4,6-trimethylpyridine (198 µL, 1.5 mmol, 3.0 equiv.), acetone (15 mL) and $H_2O$ (1 mL) were added directly to the reaction vial. From activated alcohol solution: tert-butyl 4-hydroxyazepane-1-carboxylate (107.6 mg, 0.5 mmol, 1.0 equiv.), NHC-1 (237.1 mg,

0.6 mmol, 1.2 equiv.), MTBE (15 mL) and pyridine (60 µL, 0.75 mmol, 1.5 equiv.). The residue was purified by automated flash chromatography (40 g high performance silica column, 0-100% EtOAc in hexane). The product containing fractions were then combined and after a second purification via preparative HPLC (XBridge BEH C18 OBD column, 20-100% MeCN in water (0.1% $NH_4OH$ modifier), the title compound (65.3 mg, 0.29 mmol, 58.2%) was obtained as a slightly yellow oil.

Spectroscopic data is consistent with that of reported in the literature: Zhao, H.; Cuomo, V. D.; Rossi-Ashton, J. A.; Procter, D. J. *Chem* **2024**, *10*, 1240-1251.

**$^1$H NMR (500 MHz, $CDCl_3$)** δ 3.67 – 3.24 (m, 4H), 2.83 (tq, *J* = 7.2, 3.1 Hz, 1H), 2.09 – 1.72 (m, 6H), 1.46 (d, *J* = 3.0 Hz, 9H).

**$^{13}$C NMR (126 MHz, $CDCl_3$)** δ 155.3, 121.8, 79.8, 46.2, 45.3, 43.9, 43.7, 32.0, 31.8, 29.7, 29.5, 29.4, 29.2, 28.5, 25.6, 25.5.

**IR (film)** $\nu_{max}$ 2931, 2239, 1684, 1478, 1449, 1412, 1365, 1335, 1311, 1275, 1249, 1161, 1120, 1078, 982, 895, 871, 771, 646, 540. 460.

**HRMS (ESI-TOF)** *m/z* calcd. for $C_{12}H_{20}N_2O_2Na^+$ ($[M+Na]^+$) 247.1417, found 247.1419.

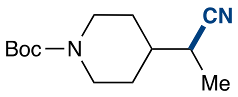


**(±)-*Tert*-butyl 4-(1-cyanoethyl)piperidine-1-carboxylate (13)**

The title compound was prepared according to a modified **General procedure A**. 4-CzPN (7.9 mg, 0.01 mmol, 2 mol%), tosyl cyanide (135.9 mg, 0.75 mmol, 1.5 equiv.), benzoyl peroxide (121.1 mg, 0.5 mmol, 1.0 equiv.), 2,4,6-trimethylpyridine (198 µL, 1.5 mmol, 3.0 equiv.), acetone (15 mL) and $H_2O$ (1 mL) were added directly to the reaction vial. From activated alcohol solution: tert-butyl 4-(1-hydroxyethyl)piperidine-1-carboxylate (114.7 mg, 0.5 mmol, 1.0 equiv.), NHC-1 (237.1 mg, 0.6 mmol, 1.2 equiv.), MTBE (15 mL) and pyridine (60 µL, 0.75 mmol, 1.5 equiv.). The residue was purified by automated flash chromatography (40 g high performance silica column, 0-100% EtOAc in hexane). The product containing fractions were then combined and after a second purification via preparative HPLC (XBridge BEH C18 OBD column, 20-100%

MeCN in water (0.1% $NH_4OH$ modifier), the title compound (74.4 mg, 0.31 mmol, 62.4 %) was obtained as a white solid.

**$^1$H NMR (400 MHz, $CDCl_3$)** δ 4.19 (s, 2H), 2.66 (dd, *J* = 18.5, 6.7 Hz, 2H), 2.52 (p, *J* = 7.0 Hz, 1H), 1.85 (dp, *J* = 13.0, 2.7 Hz, 1H), 1.71 (dt, *J* = 12.9, 2.8 Hz, 1H), 1.62 – 1.54 (m, 1H), 1.46 (s, 9H), 1.32 (d, *J* = 7.2 Hz, 5H).

**$^{13}$C NMR (126 MHz, $CDCl_3$)** δ 154.8, 121.7, 79.8, 42.6, 39.4, 31.3, 29.8, 28.6, 15.4.

**IR (film)** $\nu_{max}$ 2980, 2942, 2862, 2236, 1670, 1469, 1450, 1424, 1391, 1380, 1365, 1332, 1283, 1233, 1164, 1142, 1131, 1102, 1083, 1027, 1009, 992, 938, 867, 817, 764, 475, 637, 544, 468.

**HRMS (ESI-TOF)** *m/z* calcd. for $C_{13}H_{22}N_2O_2Na^+$ ($[M+Na]^+$) 261.1574, found 261.1572.

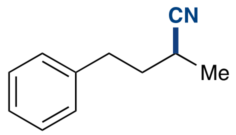


**(±)-2-methyl-4-phenyl-butanenitrile (14)**

The title compound was prepared according to a modified **General procedure A**. 4-CzPN (7.9 mg, 0.01 mmol, 2 mol%), tosyl cyanide (135.9 mg, 0.75 mmol, 1.5 equiv.), benzoyl peroxide (121.1 mg, 0.5 mmol, 1.0 equiv.), 2,4,6-trimethylpyridine (198 µL, 1.5 mmol, 3.0 equiv.), acetone (15 mL) and $H_2O$ (1 mL) were added directly to the reaction vial. From activated alcohol solution: 4-phenylbutan-2-ol (75.1 mg, 0.5 mmol, 1.0 equiv.), NHC-1 (237.1 mg, 0.6 mmol, 1.2 equiv.), MTBE (15 mL) and pyridine (60 µL, 0.75 mmol, 1.5 equiv.). The residue was purified by automated flash chromatography (40 g high performance silica column, 0-100% EtOAc in hexane). The product containing fractions were then combined and after a second purification via preparative HPLC (XBridge BEH C18 OBD column, 20-100% MeCN in water (0.1% $NH_4OH$ modifier), the title compound (52.9 mg, 0.33 mmol, 66.5 %) was obtained as a colorless liquid.

**$^1$H** and **$^{13}$C NMR** spectroscopic data is consistent with that of reported in the literature: Gasper, B.; Carreira, E. M. *Angew. Chem. Int. Ed.* **2007**, *46*, 4519–4522.

**$^{1}H$ NMR (500 MHz, $CDCl_3$)** δ 7.31 (t, $J$ = 7.5 Hz, 2H), 7.25 – 7.18 (m, 3H), 2.88 (ddd, $J$ = 14.2, 9.1, 5.4 Hz, 1H), 2.75 (ddd, $J$ = 13.9, 8.8, 7.5 Hz, 1H), 2.62 – 2.53 (m, 1H), 1.97 (dtt, $J$ = 14.4, 9.2, 4.4 Hz, 1H), 1.90 – 1.79 (m, 1H), 1.34 (d, $J$ = 7.1 Hz, 3H).

**$^{13}C$ NMR (126 MHz, $CDCl_3$)** δ 140.3, 128.8, 128.6, 126.5, 122.9, 35.9, 33.3, 25.0, 18.1.

**IR (film)** $\nu_{max}$ 3028, 2981, 2931, 2862, 2239, 1604, 1497, 1454, 1382, 1346, 1180, 1123, 1050, 1030, 918, 752, 698, 599, 558, 501.

**HRMS (GC/Q-TOF)** *m/z* calcd. for $C_{11}H_{13}N^+$ ($[M]^+$) 159.1043, found 159.1042.

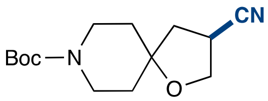


**(±)-*Tert*-butyl-3-cyano-1-oxa-8-azaspiro[4.5]decane-8-carboxylate (15)**

The title compound was prepared according to **General procedure A**. 4-CzPN (7.9 mg, 0.01 mmol, 2 mol%), tosyl cyanide (135.9 mg, 0.75 mmol, 1.5 equiv.), benzoyl peroxide (121.1 mg, 0.5 mmol, 1.0 equiv.), 2,4,6-trimethylpyridine (198 µL, 1.5 mmol, 3.0 equiv.), acetone (15 mL) and $H_2O$ (1 mL) were added directly to the reaction vial. From activated alcohol solution: tert-butyl 3-hydroxy-1-oxa-8-azaspiro[4.5]decane-8-carboxylate (128.7 mg, 0.5 mmol, 1.0 equiv.), NHC-J (255.2 mg, 0.6 mmol, 1.2 equiv.), MTBE (15 mL) and pyridine (60 µL, 0.75 mmol, 1.5 equiv.). The residue was purified by automated flash chromatography (40 g high performance silica column, 0-100% EtOAc in hexane). The product containing fractions were then combined and after a second purification via preparative HPLC (XBridge BEH C18 OBD column, 20-100% MeCN in water (0.1% $NH_4OH$ modifier)), the title compound (88.1 mg, 0.33 mmol, 66.2%) was obtained as a colorless liquid.

**$^{1}H$ NMR (400 MHz, $CDCl_3$)** δ 4.18 – 3.98 (m, 2H), 3.68 (s, 2H), 3.32 – 3.08 (m, 3H), 2.19 – 2.01 (m, 2H), 1.84 – 1.74 (m, 1H), 1.70 – 1.51 (m, 3H), 1.45 (s, 9H).

**$^{13}C$ NMR (126 MHz, $CDCl_3$)** δ 154.7, 120.5, 81.2, 79.7, 74.0, 68.8, 41.5, 40.8, 35.9, 35.9, 28.5, 28.4.

**IR (film)** $\nu_{max}$ 2974, 2942, 2871, 2242, 1682, 1469, 1418, 1365, 1283, 1241, 1171, 1145, 1109, 1068, 1047, 995, 966, 910, 862, 824, 768, 639, 575, 540, 488.

**HRMS (ESI-TOF)** *m/z* calcd. for $C_{14}H_{22}N_2O_3Na$ ($[M+Na]^+$) 289.1523, found 289.1522.

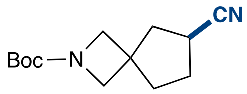


**(±)-*Tert*-butyl 6-cyano-2-azaspiro[3.4]octane-2-carboxylate (16)**

The title compound was prepared according to **General procedure A**. 4-CzPN (7.9 mg, 0.01 mmol, 2 mol%), tosyl cyanide (135.9 mg, 0.75 mmol, 1.5 equiv.), benzoyl peroxide (121.1 mg, 0.5 mmol, 1.0 equiv.), 2,4,6-trimethylpyridine (198 µL, 1.5 mmol, 3.0 equiv.), acetone (15 mL) and $H_2O$ (1 mL) were added directly to the reaction vial. From activated alcohol solution: tert-butyl 6-hydroxy-2-azaspiro[3.4]octane-2-carboxylate (113.7 mg, 0.5 mmol, 1.0 equiv.), NHC-J (255.2 mg, 0.6 mmol, 1.2 equiv.), MTBE (15 mL) and pyridine (60 µL, 0.75 mmol, 1.5 equiv.). The residue was purified by automated flash chromatography (40 g high performance silica column, 0-100% EtOAc in hexane). The product containing fractions were then combined and after a second purification via preparative HPLC (XBridge BEH C18 OBD column, 20-100% MeCN in water (0.1% $NH_4OH$ modifier)), the title compound (83.5 mg, 0.35 mmol, 70.7%) was obtained as a slightly yellow oil.

**$^1$H NMR (400 MHz, $CDCl_3$)** δ 3.98 – 3.84 (m, 2H), 3.78 (s, 2H), 2.85 (tt, *J* = 8.5, 6.7 Hz, 1H), 2.31 – 1.86 (m, 6H), 1.46 (s, 9H).

**$^{13}$C NMR (126 MHz, $CDCl_3$)** δ 156.3, 122.7, 79.8, 60.3, 42.2, 41.0, 37.1, 30.0, 29.5, 28.5, 26.8.

**IR (film)** $\nu_{max}$ 2934, 2873, 2238, 1693, 1479, 1450, 1390, 1365, 1326, 1252, 1146, 1094, 931, 860, 772, 594, 463.

**HRMS (ESI-TOF)** *m/z* calcd. for $C_{13}H_{20}N_2O_2Na$ ($[M+Na]^+$) 259.1417, found 259.1418.

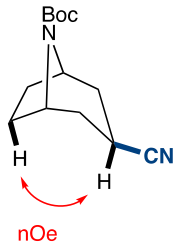


**_Tert_-butyl-3-cyano-8-azabicyclo[3.2.1]octane-8-carboxylate (17)**

Prepared following a modified **General procedure A**. 4-CzPN (7.9 mg, 0.01 mmol, 2 mol%), tosyl cyanide (135.9 mg, 0.75 mmol, 1.5 equiv.), benzoyl peroxide (121.1 mg, 0.5 mmol, 1.0 equiv.), 2,4,6-trimethylpyridine (198 µL, 1.5 mmol, 3.0 equiv.), acetone (15 mL) and $H_2O$ (1 mL) were added directly to the reaction vial. From activated alcohol solution: tert-butyl (1R,3S,5S)-3-hydroxy-8-azabicyclo[3.2.1]octane-8-carboxylate (227.3 mg, 0.5 mmol, 1.0 equiv.), NHC-J (276.47 mg, 0.65 mmol, 1.3 equiv.), MTBE (15 mL) and pyridine (60 µL, 0.75 mmol, 1.5 equiv.). The residue was purified by automated flash chromatography (40 g high performance silica column, 30-100% ethyl acetate/hexane gradient). The product containing fractions were then combined and after a second purification via preparative HPLC (XBridge BEH C18 OBD column, 30-45% MeCN in water (0.1% $NH_4OH$ modifier)), the title compound (63.1 mg, 0.27 mmol, 53%, 6.6:1 d.r.) was obtained as a white solid.

**$^1$H** and **$^{13}$C NMR** spectroscopic data is consistent with that of reported in the literature: Caiger, L., Zhao, H., Constantin, T., Douglas, J. J., and Leonori, D. *ACS Catal*. **2023**, *13*, 4985-4991.

**$^1$H NMR (500 MHz, $CDCl_3$)** δ 4.37 – 4.08 (m, 2H), 2.97 (dq, *J* = 12.3, 6.2 Hz, 1H), 2.27 – 1.92 (m, 4H), 1.85 (ddd, *J* = 13.5, 5.5, 2.6 Hz, 2H), 1.68 – 1.55 (m, 2H), 1.47 (d, *J* = 9.8 Hz, 9H).

**Quantitative $^{13}$C NMR (126 MHz, $CDCl_3$)** δ 153.1, [124.4,] 121.7, 80.0, [79.8,] 52.8, 52.1, 34.3, 33.6, 28.5, 28.0, 27.3, 20.8, [19.5]. **(minor diastereomer in brackets)**

**IR (film)** $\nu_{max}$ 2976, 2955, 2926, 2236, 1703, 1659, 1650, 1577, 1621, 1503, 1481, 1452, 1369, 1263, 1173, 1108.

**HRMS (ESI-TOF)** *m/z* calcd. for $C_9H_{13}N_2O_2^+$ ($[M\text{-isobutene}+H]^+$) 181.0972, found 181.0974.

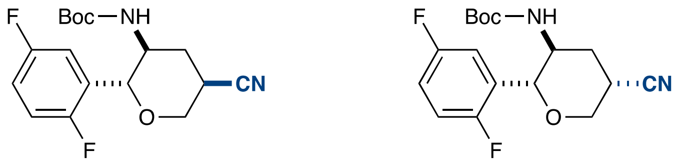


major diastereomer minor diastereomer

**Tert-butyl-5-cyano-2-(2,5-difluorophenyl)tetrahydro-2H-pyran-3-yl)carbamate (18)**

The title compound was prepared according to **General procedure A**. 4-CzPN (7.9 mg, 0.01 mmol, 2 mol%), tosyl cyanide (135.9 mg, 0.75 mmol, 1.5 equiv.), benzoyl peroxide (121.1 mg, 0.5 mmol, 1.0 equiv.), 2,4,6-trimethylpyridine (198 µL, 1.5 mmol, 3.0 equiv.), acetone (15 mL) and $H_2O$ (1 mL) were added directly to the reaction vial. From activated alcohol solution: tert-butyl N-[rac-(2R,3S)-2-(2,5-difluorophenyl)-5-hydroxy-tetrahydropyran-3-yl]carbamate (164.7 mg, 0.5 mmol, 1.0 equiv.), NHC-J (255.2 mg, 0.6 mmol, 1.2 equiv.), MTBE (15 mL) and pyridine (60 µ L, 0.75 mmol, 1.5 equiv.). The residue was purified by automated flash chromatography (40 g high performance silica column, 0-100% EtOAc in hexane). The product containing fractions were then combined and after a second purification via preparative HPLC (XBridge BEH C18 OBD column, 10-100% MeCN in water (0.1% $NH_4OH$ modifier)), the title compound (89.8 mg, 0.27 mmol, 53.1%, 1.6:1 d.r.) was obtained as a white solid. The diastereomers were separated via chiral HPLC (Enantiocel A6 (2 x 25 cm) column, 40% ethanol/$CO_2$ (100 bar), 65 mL/min, 220 nm).

**Major diastereomer**:

**$^1$H NMR (500 MHz, $CDCl_3$)** δ 7.18 – 7.11 (m, 1H), 7.03 – 6.92 (m, 2H), 4.49 – 4.39 (m, 2H), 4.30 (ddd, $J$ = 11.4, 4.6, 2.1 Hz, 1H), 3.63 (t, $J$ = 11.3 Hz, 2H), 3.02 (ddt, $J$ = 12.6, 11.3, 4.2 Hz, 1H), 2.62 (dtd, $J$ = 13.0, 4.0, 2.1 Hz, 1H), 1.86 (q, $J$ = 12.5 Hz, 1H), 1.26 (d, $J$ = 7.9 Hz, 9H).

**$^{13}$C NMR (126 MHz, $CDCl_3$)** δ 160.0, 158.1, 157.2, 155.3, 154.6, 117.9, 116.6 (dd, $J$ = 24.3, 8.8 Hz), 116.3 (dd, $J$ = 26.3, 8.2 Hz), 115.0 (dd, $J$ = 25.1, 4.1 Hz), 76.6, 68.5, 50.7, 34.4, 29.9, 28.2, 27.8.

**$^{19}$F NMR (471 MHz, $CDCl_3$)** δ -116.8 – -118.6 (m), -122.9 – -124.4 (m).

**IR (film)** $\nu_{max}$ 3381, 2987, 2937, 2856, 2245, 1686, 1600, 1524, 1498, 1478, 1464, 1428, 1394, 1371, 1323, 1300, 1275, 1257, 1234, 1166, 1151, 1122, 1100, 1090, 1049, 1029, 979, 950, 899, 884, 860, 818, 781, 765, 746, 737, 714, 613, 588, 524, 478, 482, 463, 450.

**HRMS (ESI-TOF)** *m/z* calcd. for $C_{17}H_{20}F_2O_3N_2Na$ ($[M+Na]^+$) 361.1334, found 361.1338.

**Minor diastereomer**:

**$^1$H NMR (500 MHz, $CDCl_3$)** δ 7.31 (t, *J* = 7.3 Hz, 1H), 6.98 (t, *J* = 6.2 Hz, 2H), 4.77 – 4.33 (m, 2H), 4.27 (dt, *J* = 11.8, 1.8 Hz, 1H), 3.83 (d, *J* = 10.5 Hz, 1H), 3.75 – 3.55 (m, 1H), 3.06 (dq, *J* = 4.2, 2.1 Hz, 1H), 2.48 (d, *J* = 13.5 Hz, 1H), 2.14 (d, *J* = 34.9 Hz, 1H), 1.27 (d, *J* = 20.7 Hz, 9H).

**$^{13}$C NMR (126 MHz, $CDCl_3$)** δ 160.2, 158.2, 157.2, 157.1, 155.23, 155.21, 154.5, 119.8, 116.5 (dd, *J* = 24.3, 8.7 Hz), 116.2 (d, *J* = 27.8 Hz), 115.2 (d, *J* = 24.8 Hz), 76.3, 68.3, 49.9, 32.4, 29.9, 28.6, 28.2.

**$^{19}$F NMR (471 MHz, $CDCl_3$)** δ -117.2 – -118.3 (m), -123.5 – -124.9 (m).

**IR (film)** $\nu_{max}$ 3382, 2980, 2940, 2872, 2243, 1685, 1600, 1522, 1497, 1459, 1427, 1392, 1368, 1326, 1303, 1274, 1238, 1176, 1112, 1094, 1077, 1053, 1028, 974, 949, 883, 868, 828, 811, 795, 775, 738, 725, 616, 593, 486, 475, 460, 440.

**HRMS (ESI-TOF)** *m/z* calcd. for $C_{17}H_{20}F_2O_3N_2Na$ ($[M+Na]^+$) 361.1334, found 361.1335.

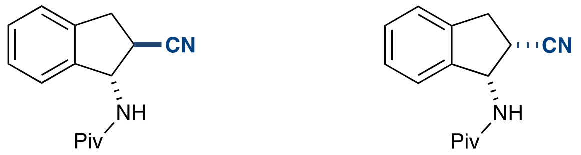


**major diastereomer** **minor diastereomer**

**_N_-(2-cyanoindan-1-yl)-2,2-dimethyl-propanamide (19)**

The title compound was prepared according to **General procedure A**. 4-CzPN (7.9 mg, 0.01 mmol, 2 mol%), tosyl cyanide (135.9 mg, 0.75 mmol, 1.5 equiv.), benzoyl peroxide (121.1 mg, 0.5 mmol, 1.0 equiv.), 2,4,6-trimethylpyridine (198 μL, 1.5 mmol, 3.0 equiv.), acetone (15 mL) and $H_2O$ (1 mL) were added directly to the reaction vial. From activated alcohol solution: *N*-

((1$R$,2$R$)-2-hydroxy-2,3-dihydro-1$H$-inden-1-yl)pivalamide (116.7 mg, 0.5 mmol, 1.0 equiv.), NHC-J (255.2 mg, 0.6 mmol, 1.2 equiv.), MTBE (15 mL) and pyridine (60 µL, 0.75 mmol, 1.5 equiv.). The residue was purified by automated flash chromatography (40 g high performance silica column, 0-100% EtOAc in hexane). The product containing fractions were then combined and after a second purification via preparative HPLC (XBridge BEH C18 OBD column, 10-100% MeCN in water (0.1% $NH_4OH$ modifier)), the title compound (60.9 mg, 0.25 mmol, 50.3%) was obtained as a white solid as a mixture of diastereomers (5:1 dr).

**Major diastereomer**

**$^1$H NMR (500 MHz, $CDCl_3$)** δ 7.37 – 7.09 (m, 4H), 6.08 (d, $J$ = 8.0 Hz, 1H), 5.62 (t, $J$ = 7.7 Hz, 1H), 3.47 – 3.35 (m, 1H), 3.24 – 3.11 (m, 2H), 1.25 (s, 9H).

**Minor diastereomer**

**$^1$H NMR (500 MHz, $CDCl_3$)** δ 7.37 – 7.09 (m, 4H), 6.19 (d, $J$ = 8.7 Hz, 1H), 5.66 (t, $J$ = 7.9 Hz, 1H), 3.76 (td, $J$ = 7.3, 4.0 Hz, 1H), 3.30 (t, $J$ = 5.1 Hz, 2H), 1.29 (s, 9H).

**$^{13}$C NMR (126 MHz, $CDCl_3$)** δ 179.2, 178.8, 140.2, 139.4, 129.3, 129.2, 128.2, 128.2, 125.4, 125.1, 123.9, 123.9, 120.8, 119.9, 58.8, 55.1, 39.0, 36.5, 36.1, 35.2, 35.2, 29.8, 27.7. **(summary of diastereomers)**

**IR (film)** $\nu_{max}$ 3405, 3311, 2958, 2918, 2849, 2244, 1656, 1638, 1510, 1479, 1459, 1395, 1365, 1327, 1301, 1279, 1227, 1192, 1154, 1072, 1024, 992, 938, 863, 806, 796, 750, 522, 691, 670, 639, 596, 448, 526, 476, 439.

**HRMS (ESI-TOF)** $m/z$ calcd. for $C_{15}H_{19}N_2O$ ($[M+H]^+$) 243.1492, found 243.1493.

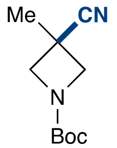


**$Tert$-butyl 3-cyano-3-methylazetidine-1-carboxylate (20)**

The title compound was prepared according to **General procedure B**. 4-CzPN (7.9 mg, 0.01 mmol, 2 mol%), tosyl cyanide (135.9 mg, 0.75 mmol, 1.5 equiv.), benzoyl peroxide (121.1 mg, 0.5 mmol, 1.0 equiv.), 2,4,6-trimethylpyridine (198 µL, 1.5 mmol, 3.0 equiv.), acetone (15

mL) and $H_2O$ (1 mL) were added directly to the reaction vial. From activated alcohol solution: tert-butyl 3-hydroxy-3-methyl-azetidine-1-carboxylate (93.6 mg, 0.5 mmol, 1.0 equiv.), NHC-5 (277.9 mg, 0.6 mmol, 1.2 equiv.), TFT (15 mL) and pyridine (60 µL, 0.75 mmol, 1.5 equiv.). The residue was purified by automated flash chromatography (40 g high performance silica column, 0-100% EtOAc in hexane). The product containing fractions were then combined and after a second purification via preparative HPLC (XBridge BEH C18 OBD column, 20-100% MeCN in water (0.1% $NH_4OH$ modifier)), the title compound (49.8 mg, 0.25 mmol, 50.7%) was obtained as a slightly yellow oil.

**$^1$H NMR (500 MHz, $CDCl_3$)** δ 4.29 (d, *J* = 8.5 Hz, 2H), 3.80 (d, *J* = 8.5 Hz, 2H), 1.67 (s, 3H), 1.44 (d, *J* = 1.0 Hz, 9H).

**$^{13}$C NMR (126 MHz, $CDCl_3$)** δ 155.8, 122.2, 80.8, 59.3, 28.4, 26.1, 23.7.

**IR (film)** $\nu_{max}$ 2977, 2890, 2242, 1700, 1478, 1453, 1387, 1366, 1307, 1255, 1205, 1166, 1108, 916, 858, 771, 758, 641, 587, 526, 465.

**HRMS (ESI-TOF)** *m/z* calcd. for $C_6H_9N_2O_2^+$ ($[M\text{-isobutene}+H]^+$) 141.0659, found 141.0660.

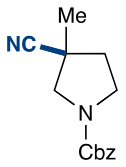


**(±)-Benzyl-3-cyano-3-methylpyrrolidine-1-carboxylate (21)**

The title compound was prepared according to **General procedure B**. 4-CzPN (7.9 mg, 0.01 mmol, 2 mol%), tosyl cyanide (135.9 mg, 0.75 mmol, 1.5 equiv.), benzoyl peroxide (121.1 mg, 0.5 mmol, 1.0 equiv.), 2,4,6-trimethylpyridine (198 µL, 1.5 mmol, 3.0 equiv.), acetone (15 mL) and $H_2O$ (1 mL) were added directly to the reaction vial. From activated alcohol solution: benzyl 3-hydroxy-3-methyl-pyrrolidine-1-carboxylate (117.6 mg, 0.5 mmol, 1.0 equiv.), NHC-5 (277.9 mg, 0.6 mmol, 1.2 equiv.), TFT (15 mL) and pyridine (60 µL, 0.75 mmol, 1.5 equiv.). The residue was purified by automated flash chromatography (40 g high performance silica column, 0-100% EtOAc in hexane). The product containing fractions were then combined and after a second purification via preparative HPLC (XBridge BEH C18 OBD column, 20-100% MeCN in water

(0.1% $NH_4OH$ modifier)), the title compound (82.3 mg, 0.34 mmol, 67.4%) was obtained as a slightly yellow oil.

**$^{1}$H NMR (400 MHz, $CDCl_3$)** δ 7.42 – 7.28 (m, 5H), 5.14 (d, $J$ = 3.4 Hz, 2H), 3.89 (dd, $J$ = 17.7, 11.1 Hz, 1H), 3.71 – 3.54 (m, 2H), 3.32 (t, $J$ = 10.6 Hz, 1H), 2.37 (qd, $J$ = 6.9, 3.3 Hz, 1H), 2.02 – 1.87 (m, 1H), 1.50 (d, $J$ = 6.2 Hz, 3H).

**$^{13}$C NMR (126 MHz, $CDCl_3$)** δ 154.4, 154.3, 136.4, 128.6, 128.2, 128.2, 128.1, 128.0, 122.8, 67.3, 67.2, 56.3, 56.0, 44.8, 44.4, 37.8, 37.4, 36.9, 36.6, 22.4, 22.3. **(summary of rotamers)**

**IR (film)** $\nu_{max}$ 2954, 2883, 2240, 1698, 1498, 1448, 1359, 1343, 1282, 1212, 1161, 1150, 1131, 1095, 1030, 1003, 988, 915, 879, 820, 767, 741, 696, 629, 599, 551, 496, 459.

**HRMS (ESI-TOF)** *m/z* calcd. for $C_{14}H_{17}N_2O_2$ ($[M+H]^+$) 245.1285, found 245.1288.

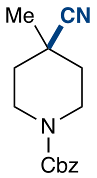


**Benzyl 4-cyano-4-methylpiperidine-1-carboxylate (22)**

The title compound was prepared according to **General procedure B**. 4-CzPN (7.9 mg, 0.01 mmol, 2 mol%), tosyl cyanide (135.9 mg, 0.75 mmol, 1.5 equiv.), benzoyl peroxide (121.1 mg, 0.5 mmol, 1.0 equiv.), 2,4,6-trimethylpyridine (198 µL, 1.5 mmol, 3.0 equiv.), acetone (15 mL) and $H_2O$ (1 mL) were added directly to the reaction vial. From activated alcohol solution: benzyl 4-hydroxy-4-methylpiperidine-1-carboxylate (124.7 mg, 0.5 mmol, 1.0 equiv.), NHC-5 (277.9 mg, 0.6 mmol, 1.2 equiv.), TFT (15 mL) and pyridine (60 µL, 0.75 mmol, 1.5 equiv.). The residue was purified by automated flash chromatography (40 g high performance silica column, 0-100% EtOAc in hexane). The product containing fractions were then combined and after a second purification via preparative HPLC (XBridge BEH C18 OBD column, 20-100% MeCN in water (0.1% $NH_4OH$ modifier), the title compound (84.7 mg, 0.33 mmol, 65.6%) was obtained as a slightly yellow oil.

**$^{1}$H NMR (400 MHz, $CDCl_3$)** δ 7.41 – 7.29 (m, 5H), 5.13 (s, 2H), 4.19 (s, 2H), 3.12 (s, 2H), 1.91 (d, $J$ = 13.5 Hz, 2H), 1.46 (dd, $J$ = 13.2, 4.3 Hz, 2H), 1.40 (s, 3H).

**$^{13}$C NMR (126 MHz, CDCl$_3$)** δ 155.0, 136.5, 128.6, 128.2, 128.0, 122.9, 67.4, 41.3, 36.2, 33.2, 26.7.

**IR (film)** $\nu_{max}$ 3033, 2933, 2869, 2234, 1704, 1498, 1430, 1366, 1351, 1281, 1248, 1227, 1172, 1118, 1092, 1014, 976, 905, 765, 699, 612.

**HRMS (ESI-TOF)** *m/z* calcd. for $C_{15}H_{19}N_2O_2^+$ ([M+H]$^+$) 259.1441, found 259.1442.

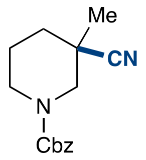


**(±)-Benzyl-3-cyano-3-methylpiperidine-1-carboxylate (23)**

The title compound was prepared according to **General procedure B**. 4-CzPN (7.9 mg, 0.01 mmol, 2 mol%), tosyl cyanide (135.9 mg, 0.75 mmol, 1.5 equiv.), benzoyl peroxide (121.1 mg, 0.5 mmol, 1.0 equiv.), 2,4,6-trimethylpyridine (198 µL, 1.5 mmol, 3.0 equiv.), acetone (15 mL) and $H_2O$ (1 mL) were added directly to the reaction vial. From activated alcohol solution: benzyl 3-hydroxy-3-methyl-piperidine-1-carboxylate (124.7 mg, 0.5 mmol, 1.0 equiv.), NHC-5 (277.9 mg, 0.6 mmol, 1.2 equiv.), TFT (15 mL) and pyridine (60 µL, 0.75 mmol, 1.5 equiv.). The residue was purified by automated flash chromatography (40 g high performance silica column, 0-100% EtOAc in hexane). The product containing fractions were then combined and after a second purification via preparative HPLC (XBridge BEH C18 OBD column, 20-100% MeCN in water (0.1% $NH_4OH$ modifier)), the title compound (76.0 mg, 0.29 mmol, 58.8%) was obtained as a slightly yellow oil.

**$^1$H NMR (500 MHz, CDCl$_3$)** δ 7.35 (dd, *J* = 22.7, 4.9 Hz, 5H), 5.14 (dd, *J* = 20.8, 13.6 Hz, 2H), 4.17 (ddd, *J* = 79.0, 51.6, 13.2 Hz, 2H), 2.83 (tt, *J* = 22.5, 11.1 Hz, 2H), 2.08 (dd, *J* = 13.9, 4.4 Hz, 1H), 1.84 (t, *J* = 11.8 Hz, 1H), 1.70 (t, *J* = 9.3 Hz, 1H), 1.46 (td, *J* = 12.8, 3.9 Hz, 1H), 1.40 – 1.30 (m, 3H).

**$^{13}$C NMR (126 MHz, CDCl$_3$)** δ 155.0, 136.6, 128.7, 128.3, 128.2, 122.7, 67.7, 52.5, 52.3, 43.8, 35.8, 34.0, 23.4, 22.4.

**IR (film)** $\nu_{max}$ 2937, 2860, 2235, 1696, 1498, 1425, 1364, 1349, 1304, 1261, 1245, 1226, 1214, 1169, 1113, 1092, 1041, 1029, 1003, 978, 914, 869, 797, 763, 735, 697, 607, 558, 462.

**HRMS (ESI-TOF)** *m/z* calcd. for $C_{15}H_{19}N_2O_2^+$ ($[M+H]^+$) 259.1441, found 259.1442.

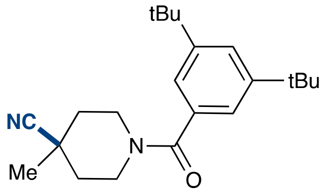


**1-(3,5-di-*tert*-butylbenzoyl)-4-methylpiperidine-4-carbonitrile (24)**

The title compound was prepared according to **General procedure B**. 4-CzPN (7.9 mg, 0.01 mmol, 2 mol%), tosyl cyanide (135.9 mg, 0.75 mmol, 1.5 equiv.), benzoyl peroxide (121.1 mg, 0.5 mmol, 1.0 equiv.), 2,4,6-trimethylpyridine (198 μL, 1.5 mmol, 3.0 equiv.), acetone (15 mL) and $H_2O$ (1 mL) were added directly to the reaction vial. From activated alcohol solution: (3,5-di-*tert*-butylphenyl)(4-hydroxy-4-methylpiperidin-1-yl)methanone (165.8 mg, 0.5 mmol, 1.0 equiv.), NHC-5 (277.9 mg, 0.6 mmol, 1.2 equiv.), TFT (15 mL) and pyridine (60 μL, 0.75 mmol, 1.5 equiv.). The residue was purified by automated flash chromatography (40 g high performance silica column, 0-100% EtOAc in hexane). The product containing fractions were then combined and after a second purification via prep HPLC (C18AQ 20×150 redisep, 10-100% acetonitrile in water (0.1 mol% formic acid modifier)), the title compound (108 mg, 0.32 mmol, 63.4 %) was obtained as a white solid.

**$^1$H NMR (400 MHz, MeOD)** δ 7.57 (t, *J* = 1.8 Hz, 1H), 7.27 (d, *J* = 1.9 Hz, 2H), 4.64 (s, 1H), 3.76 (s, 1H), 3.46 – 3.05 (m, 2H), 1.97 (d, *J* = 55.1 Hz, 2H), 1.74 – 1.52 (m, 2H), 1.44 (s, 3H), 1.35 (s, 18H).

**$^{13}$C NMR (126 MHz, MeOD)** δ 173.4, 152.7, 136.0, 125.2, 124.2, 122.2, 46.5, 40.8, 37.4, 36.7, 35.9, 34.8, 31.7, 26.5.

**IR (film)** $\nu_{max}$ 2952, 2920, 2867, 2233, 1627, 1594, 1474, 1446, 1418, 1373, 1360, 1309, 1274, 1249, 1202, 1136, 1112, 1025, 994, 982, 940, 908, 888, 830, 765, 749, 658, 637, 608, 541, 459.

**HRMS (ESI-TOF)** *m/z* calcd. for $C_{22}H_{32}N_2O^+$ ($[M+H]^+$) 341.2588, found 341.2588.

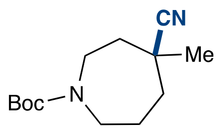

**(±)-*Tert*-butyl-4-cyano-4-methylazepane-1-carboxylate (25)**

The title compound was prepared according to **General procedure B**. 4-CzPN (7.9 mg, 0.01 mmol, 2 mol%), tosyl cyanide (135.9 mg, 0.75 mmol, 1.5 equiv.), benzoyl peroxide (121.1 mg, 0.5 mmol, 1.0 equiv.), 2,4,6-trimethylpyridine (198 µL, 1.5 mmol, 3.0 equiv.), acetone (15 mL) and $H_2O$ (1 mL) were added directly to the reaction vial. From activated alcohol solution: tert-butyl 4-hydroxy-4-methyl-azepane-1-carboxylate (114.7 mg, 0.5 mmol, 1.0 equiv.), NHC-5 (277.9 mg, 0.6 mmol, 1.2 equiv.), TFT (15 mL) and pyridine (60 µL, 0.75 mmol, 1.5 equiv.). The residue was purified by automated flash chromatography (40 g high performance silica column, 0-100% EtOAc in hexane). The product containing fractions were then combined and after a second purification via preparative HPLC (XBridge BEH C18 OBD column, 20-100% MeCN in water (0.1% $NH_4OH$ modifier)), the title compound (75.2 mg, 0.32 mmol, 63.1%) was obtained as a slightly yellow oil.

**$^1$H NMR (400 MHz, $CDCl_3$)** δ 3.79 (ddt, *J* = 76.6, 15.1, 4.0 Hz, 1H), 3.59 (tt, *J* = 14.5, 7.9 Hz, 1H), 3.40 – 3.08 (m, 2H), 2.06 – 1.87 (m, 4H), 1.72 – 1.49 (m, 2H), 1.46 (d, *J* = 1.4 Hz, 9H), 1.40 (d, *J* = 1.3 Hz, 3H).

**$^{13}$C NMR (126 MHz, $CDCl_3$)** δ 155.6, 155.5, 123.9, 46.1, 45.0, 42.8, 42.7, 40.7, 40.6, 37.4, 37.3, 37.1, 37.0, 28.6, 28.5, 28.4, 24.1, 23.9. **(summary of rotamers)**

**IR (film)** $\nu_{max}$ 2974, 2933, 2233, 1686, 1454, 1411, 1364, 1328, 1295, 1273, 1250, 1224, 1160, 1112, 1080, 1044, 970, 872, 857, 771, 611, 545, 461.

**HRMS (ESI-TOF)** *m/z* calcd. for $C_{13}H_{22}N_2O_2Na$ ($[M+Na]^+$) 261.1574, found 261.1577.

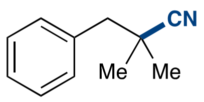

**2,2-dimethyl-3-phenylpropanenitrile (26)**

The title compound was prepared according to **General procedure B**. 4-CzPN (7.9 mg, 0.01 mmol, 2 mol%), tosyl cyanide (135.9 mg, 0.75 mmol, 1.5 equiv.), benzoyl peroxide (121.1 mg, 0.5 mmol, 1.0 equiv.), 2,4,6-trimethylpyridine (198 µL, 1.5 mmol, 3.0 equiv.), acetone (15 mL) and $H_2O$ (1 mL) were added directly to the reaction vial. From activated alcohol solution: 2-methyl-1-phenyl-propan-2-ol (75.1 mg, 0.5 mmol, 1.0 equiv.), NHC-5 (277.9 mg, 0.6 mmol, 1.2 equiv.), TFT (15 mL) and pyridine (60 µL, 0.75 mmol, 1.5 equiv.). The residue was purified by automated flash chromatography (40 g high performance silica column, 0-100% EtOAc in hexane). The product containing fractions were then combined and after a second purification via preparative HPLC (XBridge BEH C18 OBD column, 20-100% MeCN in water (0.1% $NH_4OH$ modifier)), the title compound (47.5 mg, 0.30 mmol, 59.7%) was obtained as a slightly yellow solid.

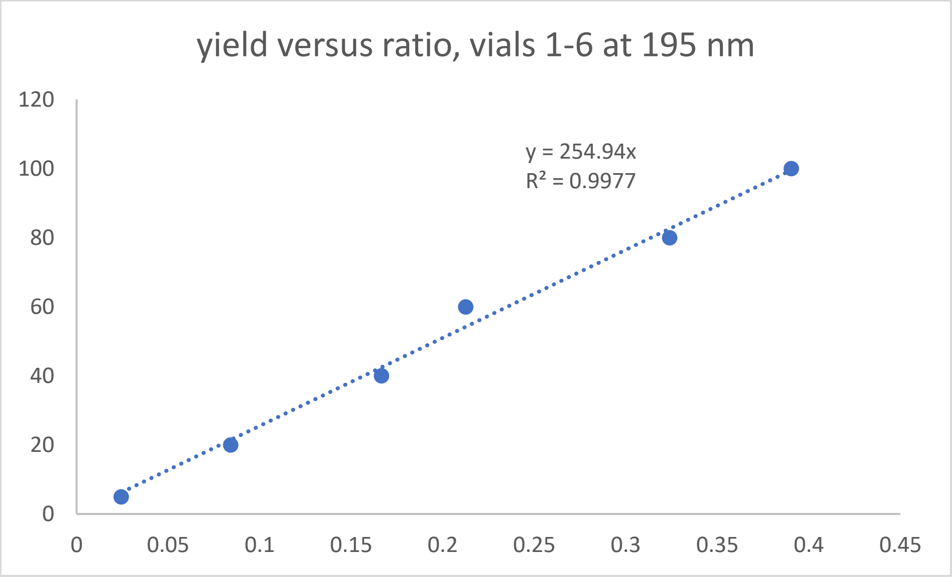


Calibration curve used for determining the assay yield using UPLC analysis and mesitylene as an internal standard. Analytical yield was determined to be 84%.

**$^1$H NMR (500 MHz, $CDCl_3$)** δ 7.40 – 7.27 (m, 5H), 2.84 (s, 2H), 1.38 (d, $J$ = 1.2 Hz, 6H).

**$^{13}$C NMR** (126 MHz, $CDCl_3$) δ 135.8, 130.3, 128.5, 127.5, 124.9, 46.8, 33.6, 26.6.

**IR (film)** $\nu_{max}$ 2975, 2931, 2855, 2230, 1722, 1602, 1494, 1467, 1451, 1389, 1369, 1262, 1192, 1126, 1071, 1032, 1023, 975, 925, 906, 859, 817, 760, 704, 683, 623, 577, 557, 478.

**HRMS (GC/Q-TOF)** *m/z* calcd. for $C_{11}H_{13}N^+$ ($[M]^+$) 159.1043, found 159.1047.

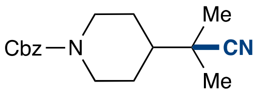

**Benzyl 4-(2-cyanopropan-2-yl)piperidine-1-carboxylate (27)**

The title compound was prepared according to **General procedure B**. 4-CzPN (7.9 mg, 0.01 mmol, 2 mol%), tosyl cyanide (135.9 mg, 0.75 mmol, 1.5 equiv.), benzoyl peroxide (121.1 mg, 0.5 mmol, 1.0 equiv.), 2,4,6-trimethylpyridine (198 µL, 1.5 mmol, 3.0 equiv.), acetone (15 mL) and $H_2O$ (1 mL) were added directly to the reaction vial. From activated alcohol solution: benzyl 4-(1-hydroxy-1-methyl-ethyl)piperidine-1-carboxylate (138.7 mg, 0.5 mmol, 1.0 equiv.), NHC-5 (277.9 mg, 0.6 mmol, 1.2 equiv.), TFT (15 mL) and pyridine (60 µL, 0.75 mmol, 1.5 equiv.). The residue was purified by automated flash chromatography (40 g high performance silica column, 0-100% EtOAc in hexane). The product containing fractions were then combined and after a second purification via preparative HPLC (XBridge BEH C18 OBD column, 20-100% MeCN in water (0.1% $NH_4OH$ modifier)), the title compound (73.4 mg, 0.26 mmol, 51.3%) was obtained as a slightly yellow oil.

**$^1$H NMR (500 MHz, CDCl$_3$)** δ 7.40 – 7.28 (m, 5H), 5.18 – 5.07 (m, 2H), 4.32 (d, *J* = 29.4 Hz, 2H), 2.73 (s, 2H), 1.82 (d, *J* = 12.6 Hz, 2H), 1.55 – 1.45 (m, 1H), 1.38 (td, *J* = 12.5, 4.2 Hz, 2H), 1.33 (s, 6H).

**$^{13}$C NMR (126 MHz, CDCl$_3$)** δ 155.2, 136.6, 128.6, 128.2, 128.1, 124.3, 67.3, 44.4, 44.0, 35.9, 27.3, 24.3.

**IR (film)** $\nu_{max}$ 2948, 2858, 2232, 1692, 1497, 1470, 1428, 1393, 1364, 1309, 1279, 1236, 1180, 1155, 1115, 1071, 1029, 955, 911, 864, 787, 763, 735, 697, 604, 589, 555, 457.

**HRMS (ESI-TOF)** *m/z* calcd. for $C_{17}H_{22}N_2O_2Na^+$ ([M+Na]$^+$) 309.1574, found 309.1573.

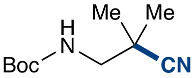

***Tert*-butyl (2-cyano-2-methylpropyl)carbamate (28)**

Prepared following **General procedure B**. 4-CzPN (7.9 mg, 0.01 mmol, 2 mol%), tosyl cyanide (135.9 mg, 0.75 mmol, 1.5 equiv.), benzoyl peroxide (121.1 mg, 0.5 mmol, 1.0 equiv.),

2,4,6-trimethylpyridine (198 μL, 1.5 mmol, 3.0 equiv.), acetone (15 mL) and $H_2O$ (1 mL) were added directly to the reaction vial. From activated alcohol solution: tert-butyl (2-hydroxy-2-methylpropyl)carbamate (94.6 mg, 0.5 mmol, 1.0 equiv.), NHC-5 (277.9 mg, 0.6 mmol, 1.2 equiv.), TFT (15 mL) and pyridine (60 μL, 0.75 mmol, 1.5 equiv.). The residue was purified by automated flash chromatography (40 g high performance silica column, 0-30% ethyl acetate/hexane gradient). The product containing fractions were then combined and after a second purification via preparative HPLC (XBridge BEH C18 OBD column, 25-40% MeCN in water (0.1% $NH_4OH$ modifier)), the title compound (52 mg, 0.26 mmol, 52%) was obtained as a white solid.

**$^1$H NMR (500 MHz, $CDCl_3$)** δ 4.95 (t, *J* = 7.0 Hz, 1H), 3.25 (d, *J* = 6.9 Hz, 2H), 1.43 (s, 9H), 1.32 (s, 6H).

**$^{13}$C NMR (126 MHz, $CDCl_3$)** δ 156.0, 124.1, 80.1, 48.9, 34.8, 28.4, 24.0.

**IR (film)** $\nu_{max}$ 3369, 2973, 2931, 2237, 1693, 1524, 1366, 1315, 1270, 1251, 1173, 975, 888, 637, 617.

**HRMS (ESI-TOF)** *m*/*z* calcd. for $C_{10}H_{18}N_2O_2Na^+$ ($[M+Na]^+$) 221.1260, found 221.1264.

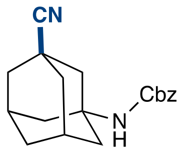


**Benzyl ((1*s*,3*r*,5*R*,7*S*)-3-cyanoadamantan-1-yl)carbamate (29)**

The title compound was prepared according to **General procedure B**. 4-CzPN (7.9 mg, 0.01 mmol, 2 mol%), tosyl cyanide (135.9 mg, 0.75 mmol, 1.5 equiv.), benzoyl peroxide (121.1 mg, 0.5 mmol, 1.0 equiv.), 2,4,6-trimethylpyridine (198 μL, 1.5 mmol, 3.0 equiv.), acetone (15 mL) and $H_2O$ (1 mL) were added directly to the reaction vial. From activated alcohol solution: benzyl N-(3-hydroxy-1-adamantyl)carbamate (150.6 mg, 0.5 mmol, 1.0 equiv.), NHC-5 (277.9 mg, 0.6 mmol, 1.2 equiv.), TFT (15 mL) and pyridine (60 μL, 0.75 mmol, 1.5 equiv.). The residue was purified by automated flash chromatography (40 g high performance silica column, 0-100% EtOAc in hexane). The product containing fractions were then combined and after a second purification via preparative HPLC (XBridge BEH C18 OBD column, 20-100% MeCN in water

(0.1% NH4OH modifier)), the title compound (116.5 mg, 0.38 mmol, 75.0%) was obtained as a colorless oil.

**$^{1}$H NMR (500 MHz, $CDCl_3$)** δ 7.43 – 7.31 (m, 5H), 5.07 (s, 2H), 4.67 (s, 1H), 2.32 (s, 2H), 2.25 (p, *J* = 3.1 Hz, 2H), 2.09 – 1.96 (m, 6H), 1.83 (dd, *J* = 12.1, 2.9 Hz, 2H), 1.69 (t, *J* = 3.3 Hz, 2H).

**$^{13}$C NMR (126 MHz, $CDCl_3$)** δ 154.2, 136.5, 128.6, 128.2, 128.1, 124.0, 66.3, 49.8, 43.0, 40.3, 38.8, 34.6, 31.8, 28.4.

**IR (film)** $\nu_{max}$ 3340, 3033, 2913, 2858, 2233, 1698, 1517, 1454, 1362, 11353, 1342, 1318, 1296, 1272, 1238, 1213, 1173, 1149, 1114, 1074, 1052, 1024, 1003, 939, 836, 777, 735, 696, 588, 493, 455.

**HRMS (ESI-TOF)** *m/z* calcd. for $C_{19}H_{23}N_2O_2^+$ ($[M+H]^+$) 311.1754, found 311.1754.

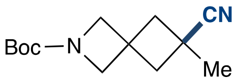


***Tert*-butyl 6-hydroxy-6-methyl-2-azaspiro[3.3]heptane-2-carboxylate (30)**

The title compound was prepared according to **General procedure B**. 4-CzPN (7.9 mg, 0.01 mmol, 2 mol%), tosyl cyanide (135.9 mg, 0.75 mmol, 1.5 equiv.), benzoyl peroxide (121.1 mg, 0.5 mmol, 1.0 equiv.), 2,4,6-trimethylpyridine (198 µL, 1.5 mmol, 3.0 equiv.), acetone (15 mL) and $H_2O$ (1 mL) were added directly to the reaction vial. From activated alcohol solution: tert-butyl 6-hydroxy-6-methyl-2-azaspiro[3.3]heptane-2-carboxylate (113.7 mg, 0.5 mmol, 1.0 equiv.), NHC-5 (277.9 mg, 0.6 mmol, 1.2 equiv.), TFT (15 mL) and pyridine (60 µ L, 0.75 mmol, 1.5 equiv.). The residue was purified by automated flash chromatography (40 g high performance silica column, 0-100% EtOAc in hexane). The product containing fractions were then combined and after a second purification via preparative HPLC (XBridge BEH C18 OBD column, 10-100% MeCN in water (0.1% NH4OH modifier)), the title compound (76.5 mg, 0.32 mmol, 64.8%) was obtained as a white solid.

**$^{1}$H NMR (500 MHz, $CDCl_3$)** δ 4.01 (s, 2H), 3.92 (s, 2H), 2.77 – 2.71 (m, 2H), 2.29 – 2.22 (m, 2H), 1.46 (s, 3H), 1.42 (s, 9H).

**$^{13}$C NMR (126 MHz, $CDCl_3$)** δ 156.1, 124.7, 79.8, 61.5, 44.4, 33.4, 28.5, 26.6, 25.1.

**IR (film)** $\nu_{max}$ 2972, 2926, 2873, 2231, 1694, 1453, 1402, 1364, 1315, 1273, 1255, 1221, 1203, 1162, 1134, 1086, 932, 859, 770, 758, 584, 467, 460.

**HRMS (ESI-TOF)** *m/z* calcd. for $C_{13}H_{20}N_2O_2Na$ ($[M+Na]^+$) 259.1417, found 259.1418.

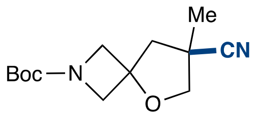


**(±)-*Tert*-butyl-7-cyano-7-methyl-5-oxa-2-azaspiro[3.4]octane-2-carboxylate (31)**

The title compound was prepared according to **General procedure B**. 4-CzPN (7.9 mg, 0.01 mmol, 2 mol%), tosyl cyanide (135.9 mg, 0.75 mmol, 1.5 equiv.), benzoyl peroxide (121.1 mg, 0.5 mmol, 1.0 equiv.), 2,4,6-trimethylpyridine (198 µL, 1.5 mmol, 3.0 equiv.), acetone (15 mL) and $H_2O$ (1 mL) were added directly to the reaction vial. From activated alcohol solution: tert-butyl 7-hydroxy-7-methyl-5-oxa-2-azaspiro[3.4]octane-2-carboxylate (121.7 mg, 0.5 mmol, 1.0 equiv.), NHC-5 (277.9 mg, 0.6 mmol, 1.2 equiv.), TFT (15 mL) and pyridine (60 µL, 0.75 mmol, 1.5 equiv.). The residue was purified by automated flash chromatography (40 g high performance silica column, 0-100% EtOAc in hexane). The product containing fractions were then combined and after a second purification via preparative HPLC (XBridge BEH C18 OBD column, 20-100% MeCN in water (0.1% NH4OH modifier)), the title compound (72.7 mg, 0.29 mmol, 57.6%) was obtained as a slightly yellow solid.

**$^1$H NMR (500 MHz, CDCl$_3$)** δ 4.16 (d, *J* = 8.9 Hz, 1H), 4.09 (dd, *J* = 9.5, 3.5 Hz, 2H), 4.03 (d, *J* = 9.5 Hz, 1H), 3.93 (d, *J* = 9.4 Hz, 1H), 3.68 (dd, *J* = 8.8, 1.4 Hz, 1H), 2.70 (d, *J* = 13.3 Hz, 1H), 2.16 (dd, *J* = 13.4, 1.1 Hz, 1H), 1.49 – 1.46 (m, 3H), 1.45 – 1.41 (m, 9H).

**$^{13}$C NMR (126 MHz, CDCl$_3$)** δ 156.3, 122.8, 80.1, 79.1, 62.9, 62.6, 48.3, 38.0, 28.5, 22.4.

**IR (film)** $\nu_{max}$ 2977, 2935, 2883, 2238, 1690, 1450, 1386, 1364, 1328, 1243, 1205, 1177, 1143, 1118, 1072, 1015, 832, 858, 768, 637, 595, 568, 532, 488, 450.

**HRMS (ESI-TOF)** *m/z* calcd. for $C_{13}H_{20}N_2O_3Na$ ($[M+Na]^+$) 275.1366, found 275.1366.

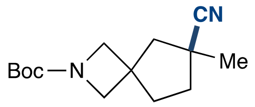

**(±)-*Tert*-butyl 6-cyano-6-methyl-2-azaspiro[3.4]octane-2-carboxylate (32)**

The title compound was prepared according to **General procedure B**. 4-CzPN (7.9 mg, 0.01 mmol, 2 mol%), tosyl cyanide (135.9 mg, 0.75 mmol, 1.5 equiv.), benzoyl peroxide (121.1 mg, 0.5 mmol, 1.0 equiv.), 2,4,6-trimethylpyridine (198 µL, 1.5 mmol, 3.0 equiv.), acetone (15 mL) and $H_2O$ (1 mL) were added directly to the reaction vial. From activated alcohol solution: tert-butyl 6-hydroxy-6-methyl-2-azaspiro[3.4]octane-2-carboxylate (120.7 mg, 0.5 mmol, 1.0 equiv.), NHC-5 (277.9 mg, 0.6 mmol, 1.2 equiv.), TFT (15 mL) and pyridine (60 µL, 0.75 mmol, 1.5 equiv.). The residue was purified by automated flash chromatography (40 g high performance silica column, 0-100% EtOAc in hexane). The product containing fractions were then combined and after a second purification via preparative HPLC (XBridge BEH C18 OBD column, 20-100% MeCN in water (0.1% NH4OH modifier), the title compound (89.5 mg, 0.36 mmol, 71.5%) was obtained as a white solid.

**$^1$H NMR (400 MHz, $CDCl_3$)** δ 3.97 (d, *J* = 8.6 Hz, 1H), 3.85 (d, *J* = 8.6 Hz, 1H), 3.82 (s, 2H), 2.45 (d, *J* = 13.7 Hz, 1H), 2.23 (ddd, *J* = 13.2, 7.8, 5.6 Hz, 1H), 2.14 (dt, *J* = 13.6, 7.8 Hz, 1H), 2.03 (ddd, *J* = 13.6, 8.2, 5.5 Hz, 1H), 1.86 (d, *J* = 13.7 Hz, 1H), 1.69 (dt, *J* = 13.1, 7.9 Hz, 1H), 1.43 (s, 9H), 1.42 (s, 3H).

**$^{13}$C NMR (126 MHz, $CDCl_3$)** δ 156.3, 125.5, 79.8, 50.9, 41.0, 39.0, 37.5, 37.4, 28.5, 25.7.

**IR (film)** $\nu_{max}$ 3002, 2924, 2876, 2228, 1683, 1477, 1446, 1411, 1362, 1299, 1243, 1174, 1139, 1121, 930, 862, 770, 703, 591, 567, 486, 464, 443.

**HRMS (ESI-TOF)** *m/z* calcd. for $C_{14}H_{22}N_2O_2Na$ ($[M+Na]^+$) 273.1573, found 273.1574.

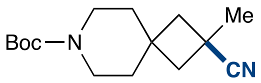


***Tert*-butyl 2-cyano-2-methyl-7-azaspiro[3.5]nonane-7-carboxylate (33)**

Prepared following **General procedure B**. 4-CzPN (7.9 mg, 0.01 mmol, 2 mol%), tosyl cyanide (135.9 mg, 0.75 mmol, 1.5 equiv.), benzoyl peroxide (121.1 mg, 0.5 mmol, 1.0 equiv.), 2,4,6-trimethylpyridine (198 µL, 1.5 mmol, 3.0 equiv.), acetone (15 mL) and $H_2O$ (1 mL) were added directly to the reaction vial. From activated alcohol solution: tert-butyl 2-hydroxy-2-methyl-7-azaspiro[3.5]nonane-7-carboxylate (227.3 mg, 0.5 mmol, 1.0 equiv.), NHC-5 (277.9 mg, 0.6 mmol, 1.2 equiv.), TFT (15 mL) and pyridine (60 µL, 0.75 mmol, 1.5 equiv.). The residue was

purified by automated flash chromatography (40 g high performance silica column, 0-100% ethyl acetate/hexane gradient). The product containing fractions were then combined and after a second purification via preparative HPLC (XBridge BEH C18 OBD column, 40-55% MeCN in water (0.1% NH4OH modifier)), the title compound (78.4 mg, 0.30 mmol, 59%) was obtained as a white solid.

**$^1$H NMR (500 MHz, $CDCl_3$)** δ 3.38 – 3.31 (m, 2H), 3.26 (dd, *J* = 6.7, 4.7 Hz, 2H), 2.47 – 2.36 (m, 2H), 1.96 – 1.84 (m, 2H), 1.72 (t, *J* = 5.7 Hz, 2H), 1.51 (s, 5H), 1.42 (s, 9H).

**$^{13}$C NMR (126 MHz, $CDCl_3$)** δ 154.9, 126.3, 79.6, 43.6, 40.2, 39.0, 37.4, 32.7, 28.5, 27.3, 24.9.

**IR (film)** $\nu_{max}$ 2972, 2971, 2845, 2228, 1674, 1455, 1426, 1361, 1256, 1245, 1181, 1151, 1132, 1085, 975, 764.

**HRMS (ESI-TOF)** *m/z* calcd. for $C_{11}H_{17}N_2O_2^+$ ($[M\text{-isobutene}+H]^+$) 209.1285, found 209.1286.

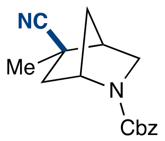


**Benzyl 5-cyano-5-methyl-2-azabicyclo[2.2.1]heptane-2-carboxylate (34)**

The title compound was prepared according to **General procedure B**. 4-CzPN (7.9 mg, 0.01 mmol, 2 mol%), tosyl cyanide (135.9 mg, 0.75 mmol, 1.5 equiv.), benzoyl peroxide (121.1 mg, 0.5 mmol, 1.0 equiv.), 2,4,6-trimethylpyridine (198 µL, 1.5 mmol, 3.0 equiv.), acetone (15 mL) and $H_2O$ (1 mL) were added directly to the reaction vial. From activated alcohol solution: benzyl 5-hydroxy-5-methyl-2-azabicyclo[2.2.1]heptane-2-carboxylate (130.7 mg, 0.5 mmol, 1.0 equiv.), NHC-5 (277.9 mg, 0.6 mmol, 1.2 equiv.), TFT (15 mL) and pyridine (60 µL, 0.75 mmol, 1.5 equiv.). The residue was purified by automated flash chromatography (40 g high performance silica column, 0-100% EtOAc in hexane). The product containing fractions were then combined and after a second purification via preparative HPLC (XBridge BEH C18 OBD column, 20-100% MeCN in water (0.1% NH4OH modifier)), the title compound (79.1 mg, 0.29 mmol, 58.5%, 1:1 d.r.) was obtained as a slightly yellow oil.

**$^1$H NMR (400 MHz, $CDCl_3$)** δ 7.34 (d, *J* = 5.5 Hz, 5H), 5.12 (d, *J* = 7.0 Hz, 2H), 4.34 (d, *J* = 29.1 Hz, 1H), 3.50 – 3.24 (m, 2H), 2.76 (s, 1H), 2.27 (ddd, *J* = 13.3, 10.3, 2.9 Hz, 1H), 2.06 (d, *J* = 10.9

Hz, 1H), 1.92 (t, $J$ = 9.9 Hz, 1H), 1.78 – 1.56 (m, 1H), 1.49 – 1.44 (m, 3H). **(summary of diastereomers)**

**$^{13}$C NMR (126 MHz, CDCl$_3$)** δ 154.5, 154.2, 136.73, 136.66, 128.7, 128.3, 128.2, 128.1, 128.0, 125.8, 125.7, 67.1, 67.0, 57.3, 57.2, 47.4, 47.0, 46.6, 46.5, 45.8, 45.3, 39.1, 38.6, 36.1, 36.0, 22.12, 22.05. **(Summary of diastereomers**.)

**IR (film)** $\nu_{max}$ 2978, 2861, 2232, 1694, 1497, 1445, 1412, 1358, 1329, 1301, 1291, 1263, 1211, 1181, 1142, 1094, 1029, 1008, 977, 950, 929, 909, 888, 868, 815, 735, 697, 597, 549, 459.

**HRMS (ESI-TOF)** *m/z* calcd. for $C_{16}H_{18}N_2O_2Na^+$ ($[M+Na]^+$) 293.1261, found 293.1262.

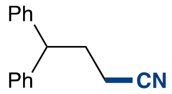


**4,4-diphenylbutanenitrile (35)**

The title compound was prepared according to **General procedure A**. 4-CzPN (7.9 mg, 0.01 mmol, 2 mol%), tosyl cyanide (135.9 mg, 0.75 mmol, 1.5 equiv.), benzoyl peroxide (121.1 mg, 0.5 mmol, 1.0 equiv.), 2,4,6-trimethylpyridine (198 µL, 1.5 mmol, 3.0 equiv.), acetone (15 mL) and $H_2O$ (1 mL) were added directly to the reaction vial. From activated alcohol solution: 3,3-diphenylpropan-1-ol (106.4 mg, 0.5 mmol, 1.0 equiv.), NHC-J (255.2 mg, 0.6 mmol, 1.2 equiv.), MTBE (15 mL) and pyridine (60 µL, 0.75 mmol, 1.5 equiv.). The residue was purified by automated flash chromatography (40 g high performance silica column, 0-100% EtOAc in hexane). The product containing fractions were then combined and after a second purification via preparative HPLC (XBridge BEH C18 OBD column, 20-100% MeCN in water (0.1% $NH_4OH$ modifier) the title compound (43.1 mg, 0.19 mmol, 39.0%) was obtained as a slightly yellow oil.

**$^1$H NMR** is consistent with known literature compound spectra: Elz, S.; Kramer, K.; Pertz, H. H.; Detert, H.; ter Laak, A. M.; Kühne, R.; Schunack, W. *J. Med. Chem*. **2000**, *43*, 1071–1084.

**$^1$H NMR (400 MHz, CDCl$_3$)** δ 7.34 – 7.28 (m, 4H), 7.26 – 7.19 (m, 6H), 4.06 (t, $J$ = 8.0 Hz, 1H), 2.47 – 2.35 (m, 2H), 2.28 (td, $J$ = 7.1, 1.0 Hz, 2H).

**$^{13}$C NMR (126 MHz, CDCl$_3$)** δ 142.9, 129.0 127.8, 127.0, 119.5, 50.0, 31.2, 16.0.

**IR (film)** $\nu_{max}$ 3060, 3026, 2933, 2244, 1598, 1493, 1450, 1422, 1156, 1087, 1031, 911, 842, 770, 740, 733, 696, 618, 632, 600, 5981, 533, 506, 474.

**HRMS (GC/Q-TOF)** *m/z* calcd. for $C_{16}H_{15}N^+$ ($[M]^+$) 221.1199, found 221.1197.

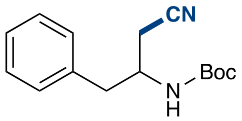


***Tert*-butyl N-(1-benzyl-2-cyano-ethyl)carbamate (36)**

The title compound was prepared according to **General procedure A**. 4-CzPN (7.9 mg, 0.01 mmol, 2 mol%), tosyl cyanide (135.9 mg, 0.75 mmol, 1.5 equiv.), benzoyl peroxide (121.1 mg, 0.5 mmol, 1.0 equiv.), 2,4,6-trimethylpyridine (198 µL, 1.5 mmol, 3.0 equiv.), acetone (15 mL) and $H_2O$ (1 mL) were added directly to the reaction vial. From activated alcohol solution: tert-butyl N-(1-benzyl-2-hydroxy-ethyl)carbamate (125.6 mg, 0.5 mmol, 1.0 equiv.), NHC-J (255.2 mg, 0.6 mmol, 1.2 equiv.), MTBE (15 mL) and pyridine (60 µL, 0.75 mmol, 1.5 equiv.). The residue was purified by automated flash chromatography (40 g high performance silica column, 0-100% EtOAc in hexane). The product containing fractions were then combined and after a second purification via preparative HPLC (XBridge BEH C18 OBD column, 20-100% MeCN in water (0.1% $NH_4OH$ modifier), the title compound (44.0 mg, 0.17 mmol, 33.8%) was obtained as a slightly yellow oil.

**$^1$H NMR (500 MHz, $CDCl_3$)** δ 7.41 – 7.23 (m, 5H), 4.81 (d, *J* = 8.1 Hz, 1H), 4.18 – 4.07 (m, 1H), 3.05 (dd, *J* = 13.9, 6.5 Hz, 1H), 2.91 (dd, *J* = 13.7, 8.3 Hz, 1H), 2.75 (dd, *J* = 16.9, 5.3 Hz, 1H), 2.46 (dd, *J* = 16.8, 4.4 Hz, 1H), 1.47 (s, 9H).

**$^{13}$C NMR (126 MHz, $CDCl_3$)** δ 155.0, 136.3, 129.2, 129.1, 127.4, 117.5, 80.4, 48.6, 39.5, 28.5, 28.4, 22.6.

**IR (film)** $\nu_{max}$ 3343, 2975, 2927, 2854, 2243, 1689, 1604, 1526, 1498, 1456, 1439, 1417, 1392, 1367, 1355, 1331, 1272, 1252, 1226, 1160, 1084, 1057, 1043, 1023, 922, 893, 851, 809, 776, 763, 748, 705, 631, 608, 516, 491, 466, 447.

**HRMS (ESI-TOF)** *m/z* calcd. for $C_{15}H_{21}N_2O_2$ ($[M+Na]^+$) 261.1598, found 261.1599.

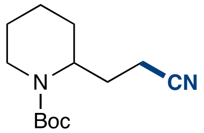

**Tert-butyl 2-(2-cyanoethyl)piperidine-1-carboxylate (37)**

The title compound was prepared according to **General procedure A**. 4-CzPN (7.9 mg, 0.01 mmol, 2 mol%), tosyl cyanide (135.9 mg, 0.75 mmol, 1.5 equiv.), benzoyl peroxide (121.1 mg, 0.5 mmol, 1.0 equiv.), 2,4,6-trimethylpyridine (198 µL, 1.5 mmol, 3.0 equiv.), acetone (15 mL) and $H_2O$ (1 mL) were added directly to the reaction vial. From activated alcohol solution: tert-butyl 2-(2-hydroxyethyl)piperidine-1-carboxylate (114.7 mg, 0.5 mmol, 1.0 equiv.), NHC-J (255.2 mg, 0.6 mmol, 1.2 equiv.), MTBE (15 mL) and pyridine (60 µL, 0.75 mmol, 1.5 equiv.). The residue was purified by automated flash chromatography (40 g high performance silica column, 0-100% EtOAc in hexane). The product containing fractions were then combined and after a second purification via preparative HPLC (XBridge BEH C18 OBD column, 20-100% MeCN in water (0.1% $NH_4OH$ modifier), the title compound (42.6 mg, 0.18 mmol, 35.8%) was obtained as a slightly yellow oil.

The spectroscopic data is consistent with that of reported in the literature: Abadie, B.; Jardel, D.; Pozzi, G.; Toullec, R.; Vincent, J.-M. *Chem. Eur. J.* **2019**, *25*, 15120.

**$^1$H NMR (400 MHz, $CDCl_3$)** $\delta$ 4.33 (dt, $J$ = 9.3, 4.7 Hz, 1H), 4.03 (d, $J$ = 13.8 Hz, 1H), 2.71 (t, $J$ = 12.9 Hz, 1H), 2.30 (t, $J$ = 7.0 Hz, 2H), 2.23 – 2.08 (m, 1H), 1.76 – 1.49 (m, 7H), 1.47 (s, 9H).

**$^{13}$C NMR (126 MHz, $CDCl_3$)** $\delta$ 155.2, 119.8, 80.1, 49.7, 28.9, 28.6, 26.3, 25.5, 19.2, 14.5.

**IR (film)** $\nu_{max}$ 2933, 2864, 2245, 1680, 1475, 1447, 1412, 1391, 1364, 1339, 1273, 1243, 1159, 1136, 1086, 1051, 1034, 995, 960, 903, 865, 815, 766, 661, 590, 530, 501, 458, 440.

**HRMS (ESI-TOF)** *m/z* calcd. for $C_{13}H_{22}N_2O_2Na^+$ ($[M+Na]^+$) 261.1574, found 261.1574.

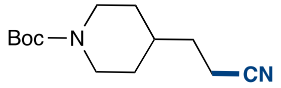

**Tert-butyl 4-(2-cyanoethyl)piperidine-1-carboxylate (38)**

The title compound was prepared according to **General procedure A**. 4-CzPN (7.9 mg, 0.01 mmol, 2 mol%), tosyl cyanide (135.9 mg, 0.75 mmol, 1.5 equiv.), benzoyl peroxide (121.1 mg, 0.5 mmol, 1.0 equiv.), 2,4,6-trimethylpyridine (198 µL, 1.5 mmol, 3.0 equiv.), acetone (15 mL) and $H_2O$ (1 mL) were added directly to the reaction vial. From activated alcohol solution: tert-butyl 4-(2-hydroxyethyl)piperidine-1-carboxylate (114.7 mg, 0.5 mmol, 1.0 equiv.), NHC-J (255.2 mg, 0.6 mmol, 1.2 equiv.), MTBE (15 mL) and pyridine (60 µL, 0.75 mmol, 1.5 equiv.). The residue was purified by automated flash chromatography (40 g high performance silica column, 0-100% EtOAc in hexane). The product containing fractions were then combined and after a second purification via preparative HPLC (XBridge BEH C18 OBD column, 35-50% MeCN in water (0.1% $NH_4OH$ modifier), the title compound (40.4 mg, 0.17 mmol, 34%) was obtained as a slightly yellow oil.

**$^1$H** and **$^{13}$C NMR** spectroscopic data is consistent with that of reported in the literature: Yamawaki, M.; Hashimoto, R.; Kawabata, Y.; Ichihashi, M.; Nachi, Y.; Inari, R.; Sakamoto, C.; Morita, T.; Yoshimi, Y. *Eur. J. Org. Chem*. **2022**, e202201225.

**$^1$H NMR (500 MHz, $CDCl_3$)** δ 4.27 – 3.94 (m, 2H), 2.69 (t, *J* = 13.4 Hz, 2H), 2.38 (t, *J* = 7.1 Hz, 2H), 1.67 (dt, *J* = 13.1, 2.5 Hz, 2H), 1.63 – 1.49 (m, 3H), 1.44 (s, 9H), 1.10 (tdd, *J* = 12.8, 11.0, 4.4 Hz, 2H).

**$^{13}$C NMR (126 MHz, $CDCl_3$)** δ 154.9, 119.7, 79.6, 43.7, 35.1, 31.8, 31.5, 28.5, 14.6.

**IR (film)** $\nu_{max}$ 2938, 2855, 2252, 1686, 1422, 1367, 1278, 1247, 1166, 1128, 1037, 967, 918.

**HRMS (ESI-TOF)** *m/z* calcd. for $C_9H_{15}N_2O_2$ ([M–isobutene+H]$^+$) 183.1128, found 183.1125.

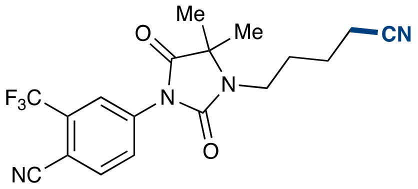


**4-(3-(4-cyanobutyl)-4,4-dimethyl-2,5-dioxoimidazolidin-1-yl)-2-(trifluoromethyl)benzonitrile (39)**

The title compound was prepared according to **General procedure A**. 4-CzPN (7.9 mg, 0.01 mmol, 2 mol%), tosyl cyanide (135.9 mg, 0.75 mmol, 1.5 equiv.), benzoyl peroxide (121.1 mg, 0.5 mmol, 1.0 equiv.), 2,4,6-trimethylpyridine (198 µL, 1.5 mmol, 3.0 equiv.), acetone (15 mL) and $H_2O$ (1 mL) were added directly to the reaction vial. From activated alcohol solution: 4-[3-(4-hydroxybutyl)-4,4-dimethyl-2,5-dioxo-imidazolidin-1-yl]-2-(trifluoromethyl)benzonitrile (184.7 mg, 0.5 mmol, 1.0 equiv.), NHC-J (255.2 mg, 0.6 mmol, 1.2 equiv.), MTBE (15 mL) and pyridine (60 µ L, 0.75 mmol, 1.5 equiv.). The residue was purified by automated flash chromatography (40 g high performance silica column, 0-100% EtOAc in hexane). The product containing fractions were then combined and after a second purification via preparative HPLC (XBridge BEH C18 OBD column, 40-55% MeCN in water (0.1% $NH_4OH$ modifier), the title compound (56.0 mg, 0.19 mmol, 29.6%) was obtained as a slightly yellow oil.

**$^{1}$H NMR (500 MHz, $CDCl_3$)** δ 8.14 (d, *J* = 2.0 Hz, 1H), 8.00 (dd, *J* = 8.4, 2.1 Hz, 1H), 7.92 (d, *J* = 8.5 Hz, 1H), 3.47 – 3.37 (m, 2H), 2.46 (t, *J* = 6.9 Hz, 2H), 1.95 – 1.83 (m, 2H), 1.83 – 1.72 (m, 2H), 1.55 (s, 6H).

**$^{13}$C NMR (126 MHz, $CDCl_3$)** δ 174.5, 153.2, 136.5, 135.4, 133.8 (q, *J* = 33.3 Hz), 128.0, 123.1 (q, *J* = 4.9 Hz), 121.0, 119.2, 115.1, 108.5, 108.5, 108.5, 108.5, 62.1, 39.4, 28.8, 23.7, 22.9, 17.0.

**$^{19}$F NMR (471 MHz, $CDCl_3$)** δ –62.0.

**IR (film)** $\nu_{max}$ 2927, 2868, 1688, 1643, 1510, 1367, 1433, 1340, 1278, 1247, 1170, 1128, 1009, 594, 556.

**HRMS (ESI-TOF)** *m/z* calcd. for $C_{18}H_{18}F_3N_4O_2^+$ ($[M+H]^+$) 379.1376, found 379.1381.

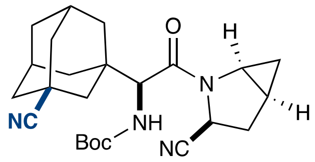


**Tert-butyl ((S)-2-((1S,3S,5S)-3-cyano-2-azabicyclo[3.1.0]hexan-2-yl)-1-((1r,3R,5R,7S)-3-cyanoadamantan-1-yl)-2-oxoethyl)carbamate (40)**

The title compound was prepared according to **General procedure B**. 4-CzPN (7.9 mg, 0.01 mmol, 2 mol%), tosyl cyanide (135.9 mg, 0.75 mmol, 1.5 equiv.), benzoyl peroxide (121.1 mg, 0.5 mmol, 1.0 equiv.), 2,4,6-trimethylpyridine (198 µL, 1.5 mmol, 3.0 equiv.), acetone (15 mL) and $H_2O$ (1 mL) were added directly to the reaction vial. From activated alcohol solution: *tert*-butyl ((*S*)-2-((1*S*,3*S*,5*S*)-3-cyano-2-azabicyclo[3.1.0]hexan-2-yl)-1-((1*r*,3*R*,5*R*,7*S*)-3-hydroxyadamantan-1-yl)-2-oxoethyl)carbamate (207.76 mg, 0.5 mmol, 1.0 equiv.), NHC-5 (277.9 mg, 0.6 mmol, 1.2 equiv.), TFT (15 mL) and pyridine (60 µL, 0.75 mmol, 1.5 equiv.). The residue was purified by automated flash chromatography (40 g high performance silica column, 0-100% EtOAc in hexane). The product containing fractions were then combined and after a second purification via preparative HPLC (XBridge BEH C18 OBD column, 35-55% MeCN in water (0.1% NH4OH modifier)), the title compound (123.7 mg, 0.29 mmol, 58%) was obtained as a white solid.

**$^1$H NMR (500 MHz, CDCl3)** δ 5.26 (d, *J* = 9.9 Hz, 1H), 5.02 (dd, *J* = 10.6, 2.3 Hz, 1H), 4.42 (d, *J* = 9.9 Hz, 1H), 3.80 (dt, *J* = 9.1, 4.2 Hz, 1H), 2.57 (ddd, *J* = 13.6, 10.6, 5.8 Hz, 1H), 2.37 (dd, *J* = 13.7, 2.4 Hz, 1H), 2.19 – 2.07 (m, 3H), 2.04 – 1.93 (m, 4H), 1.89 (dt, *J* = 7.9, 6.1 Hz, 1H), 1.82 (tt, *J* = 11.0, 2.0 Hz, 2H), 1.78 – 1.72 (m, 1H), 1.66 (tq, *J* = 3.4, 1.5 Hz, 2H), 1.60 – 1.53 (m, 2H), 1.42 (s, 9H), 1.10 – 1.01 (m, 2H).

**$^{13}$C NMR (126 MHz, $CDCl_3$)** δ 169.5, 155.9, 124.5, 119.2, 80.4, 58.7, 45.3, 40.7, 39.3, 39.3, 38.1, 37.7, 37.6, 36.5, 35.0, 30.8, 30.5, 28.4, 27.3, 27.2, 18.0, 13.7.

**IR (film)** $\nu_{max}$ 3324, 2928, 2858, 2233, 1700, 1642, 1498, 1446, 1424, 1244, 1160, 1046, 1021, 914,728, 684, 647.

**HRMS (ESI-TOF)** *m/z* calcd. for $C_{24}H_{33}N_4O_3^+$ ([M+H]$^+$) 425.2547, found 425.2545.

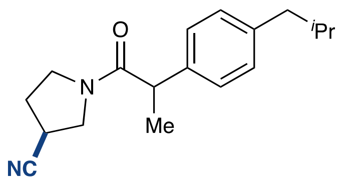

**(±)-1-(2-(4-isobutylphenyl)propanoyl)pyrrolidine-3-carbonitrile (41)**

The title compound was prepared according to **General procedure A**. 4-CzPN (7.9 mg, 0.01 mmol, 2 mol%), tosyl cyanide (135.9 mg, 0.75 mmol, 1.5 equiv.), benzoyl peroxide (121.1 mg, 0.5 mmol, 1.0 equiv.), 2,4,6-trimethylpyridine (198.4 µL, 1.5 mmol, 3.0 equiv.), acetone (15 mL) and $H_2O$ (1 mL) were added directly to the reaction vial. From activated alcohol solution: 1-(3-hydroxypyrrolidin-1-yl)-2-(4-isobutylphenyl)propan-1-one (137.7 mg, 0.5 mmol, 1.0 equiv.), NHC-J (276.4 mg, 0.65 mmol, 1.3 equiv.), MTBE (15 mL) and pyridine (60.4 µL, 0.75 mmol, 1.5 equiv.). The residue was purified by automated flash chromatography (40 g high performance silica column, 0-100% EtOAc in hexane). The product containing fractions were then combined and after a second purification via preparative HPLC (XBridge BEH C18 OBD column, 30-45% MeCN in water (0.1% NH4OH modifier)), the title compound (62.9 mg, 0.22 mmol, 44.2%) was obtained as an off-white solid (diastereomers unassigned).

**$^{1}$H NMR (500 MHz, $CDCl_3$)** δ 7.15 (dd, $J$ = 8.1, 4.0 Hz, 2H), 7.09 (td, $J$ = 8.2, 4.9 Hz, 2H), 4.01 – 3.22 (m, 5H), 3.14 – 2.91 (m, 1H), 2.44 (ddd, $J$ = 7.4, 4.8, 3.1 Hz, 2H), 2.34 – 2.01 (m, 2H), 1.84 (dqt, $J$ = 13.4, 8.6, 3.1 Hz, 1H), 1.44 (ddd, $J$ = 6.8, 4.1, 2.5 Hz, 3H), 0.89 (dd, $J$ = 6.7, 2.4 Hz, 6H). **(summary of diastereomers)**

**$^{13}$C NMR (126 MHz, $CDCl_3$)** δ 172.83, 172.80, 172.5, 172.4, 140.8, 140.74, 140.70, 140.65, 138.3, 138.2, 138.1, 137.9, 129.93, 129.89, 129.85, 129.8, 127.3, 127.23, 127.17, 120.0, 119.6, 119.1, 49.2, 49.08, 49.05, 45.14, 45.08, 45.04, 44.97, 44.9, 44.82, 44.81, 44.7, 30.6, 30.3, 29.8, 28.9, 28.8, 28.64, 28.62, 27.1, 26.9, 22.53, 22.52, 20.5, 20.4, 20.31, 20.25. **(Summary of diastereomers.)**

**IR (film)** $\nu_{max}$ 2956, 2869, 2250, 1641, 1427, 1366. 1168, 1063, 851, 593.

**HRMS (ESI-TOF)** *m/z* calcd. for $C_{18}H_{25}N_2O^+$ ($[M+H]^+$) 285.1961, found 285.1963.

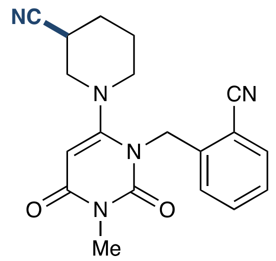

**(±)-1-(3-(2-cyanobenzyl)-1-methyl-2,6-dioxo-1,2,3,6-tetrahydropyridin-4-yl)piperidine-3-carbonitrile (42)**

Prepared following **General procedure A with** slight modification. 4-CzPN (7.9 mg, 0.01 mmol, 2 mol%), tosyl cyanide (135.9 mg, 0.75 mmol, 1.5 equiv.), benzoyl peroxide (121.1 mg, 0.5 mmol, 1.0 equiv.), 2,4,6-trimethylpyridine (198 µL, 1.5 mmol, 3.0 equiv.), acetone (15 mL) and $H_2O$ (1 mL) were added directly to the reaction vial. From activated alcohol solution: 2-((4-(3-hydroxypiperidin-1-yl)-1-methyl-2,6-dioxo-1,2,3,6-tetrahydropyridin-3-yl)methyl)benzonitrile (170.19 mg, 0.5 mmol, 1.0 equiv.), NHC-J (255.2 mg, 0.6 mmol, 1.2 equiv.), ***CpOMe*** (15 mL) and pyridine (60 µL, 0.75 mmol, 1.5 equiv.). The residue was purified by automated flash chromatography (40 g high performance silica column, 0-5% MeOH/DCM gradient). The product containing fractions were then combined and after a second purification via preparative HPLC (XBridge BEH C18 OBD column, 10-35% acetonitrile (0.1 mol% NH4OH) /$H_2O$ (0.1 mol% $NH_4OH$)), the title compound (84.2 mg, 0.24 mmol, 48%) was obtained as a yellow solid.

**$^1$H NMR (500 MHz, $CDCl_3$)** δ 7.67 (dd, *J* = 7.8, 1.4 Hz, 1H), 7.56 (td, *J* = 7.8, 1.4 Hz, 1H), 7.42 – 7.33 (m, 1H), 7.17 (d, *J* = 7.9 Hz, 1H), 5.40 (s, 1H), 5.30 (q, *J* = 15.9 Hz, 2H), 3.29 (s, 3H), 3.05 (s, 1H), 2.97 – 2.72 (m, 3H), 1.92 (s, 3H), 1.72 (q, *J* = 8.0 Hz, 1H).

**$^{13}$C NMR (126 MHz, $CDCl_3$)** δ 162.9, 159.1, 152.4, 140.3, 133.4, 133.3, 128.3, 127.2, 119.5, 117.3, 111.1, 91.6, 52.8, 52.4, 46.2, 28.1, 27.5, 27.1, 22.8.

**IR (film)** $\nu_{max}$ 2953, 2924, 2852, 2222, 1700, 1645, 1434, 1378, 1224, 764, 726, 517.

**HRMS (ESI-TOF)** *m/z* calcd. for $C_{20}H_{21}N_4O_2^+$ ($[M+H]^+$) 350.1612, found 350.1611.

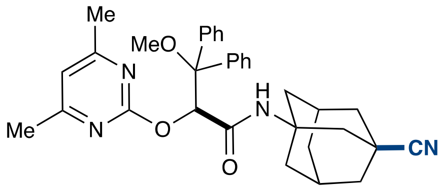


**($S$)-$N$-((1$s$,3$S$,5$R$,7$S$)-3-cyanoadamantan-1-yl)-2-((4,6-dimethylpyrimidin-2-yl)oxy)-3-methoxy-3,3-diphenylpropanamide (43)**

The title compound was prepared according to a modified **General procedure B** on a 0.4 mmol scale. 4-CzPN (6.3 mg, 8 µmol, 2 mol%), tosyl cyanide (108.73 mg, 0.6 mmol, 1.5 equiv.), benzoyl peroxide (96.89 mg, 0.4 mmol, 1.0 equiv.), 2,4,6-trimethylpyridine (158.7 µL, 1.45 mmol, 3.0 equiv.), acetone (12 mL) and $H_2O$ (0.8 mL) were added directly to the reaction vial. From activated alcohol solution: ($S$)-2-((4,6-dimethylpyrimidin-2-yl)oxy)-$N$-((1$r$,3$R$,5$R$,7$S$)-3-hydroxyadamantan-1-yl)-3-methoxy-3,3-diphenylpropanamide (211.06 mg, 0.4 mmol, 1.0 equiv.), NHC-5 (240.9 mg, 0.52 mmol, 1.3 equiv.), TFT (12 mL) and pyridine (48.3 µL, 0.6 mmol, 1.5 equiv.). The residue was purified by automated flash chromatography (40 g high performance silica column, 0-100% EtOAc in hexane). The product containing fractions were then combined and after a second purification via preparative HPLC (XBridge BEH C18 OBD column, 65-70% MeCN in water (0.1% NH4OH modifier)), the title compound (121.5 mg, 0.23 mmol, 56.6%) was obtained as a white solid.

**$^1$H NMR (500 MHz, $CDCl_3$)** δ 7.64 – 7.55 (m, 2H), 7.41 – 7.30 (m, 5H), 7.30 – 7.21 (m, 3H), 6.73 (s, 1H), 6.27 (d, $J$ = 1.6 Hz, 1H), 5.09 (s, 1H), 3.23 (d, $J$ = 2.2 Hz, 3H), 2.42 (d, $J$ = 2.8 Hz, 6H), 2.10 – 1.97 (m, 3H), 1.91 (dd, $J$ = 12.3, 2.3 Hz, 1H), 1.88 – 1.79 (m, 4H), 1.70 (d, $J$ = 12.2 Hz, 2H), 1.58 – 1.49 (m, 2H), 1.45 (dd, $J$ = 12.5, 2.9 Hz, 1H), 1.35 (dd, $J$ = 12.3, 2.9 Hz, 1H).

**$^{13}$C NMR (126 MHz, $CDCl_3$)** δ 169.6, 167.1, 163.7, 141.0, 140.8, 129.2, 129.1, 128.0, 127.8, 127.7, 127.6, 124.1, 115.3, 84.1, 77.9, 52.7, 50.1, 42.2, 39.7, 39.6, 38.9, 34.7, 31.6, 28.3, 24.0.

**IR (film)** $\nu_{max}$ 3368, 2975, 2931, 2238, 1693, 1524, 1447, 1366, 1314, 1270, 1251, 1174, 1099, 1050, 974, 888, 697, 639, 617.

**HRMS (ESI-TOF)** $m/z$ calcd. for $C_{33}H_{37}N_4O_3^+$ ($[M+H]^+$) 537.2860, found 537.2862.

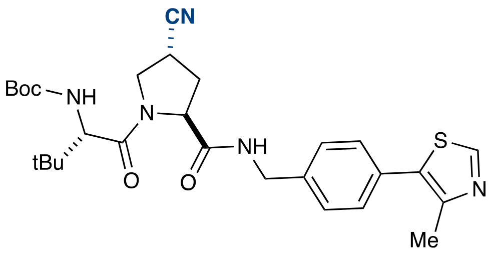

**Tert-butyl N-[rac-(1S)-2,2-dimethyl-1-[rac-(2S,4R)-4-cyano-2-[[4-(4-methylthiazol-5-yl)phenyl]methylcarbamoyl]pyrrolidine-1-carbonyl]propyl]carbamate (44)**

The title compound was prepared according to **General procedure A**. 4-CzPN (7.9 mg, 0.01 mmol, 2 mol%), tosyl cyanide (135.9 mg, 0.75 mmol, 1.5 equiv.), benzoyl peroxide (121.1 mg, 0.5 mmol, 1.0 equiv.), 2,4,6-trimethylpyridine (198 µL, 1.5 mmol, 3.0 equiv.), acetone (15 mL) and $H_2O$ (1 mL) were added directly to the reaction vial. From activated alcohol solution: tert-butyl N-[rac-(1S)-2,2-dimethyl-1-[rac-(2S,4R)-4-hydroxy-2-[[4-(4-methylthiazol-5-yl)phenyl]methylcarbamoyl]pyrrolidine-1-carbonyl]propyl]carbamate (265.3 mg, 0.5 mmol, 1.0 equiv.), NHC-J (255.2 mg, 0.6 mmol, 1.2 equiv.), MTBE (15 mL) and pyridine (60 µL, 0.75 mmol, 1.5 equiv.). The residue was purified by automated flash chromatography (40 g high performance silica column, 0-100% EtOAc in hexane). The product containing fractions were then combined and after a second purification via preparative HPLC (XBridge BEH C18 OBD column, 10-100% MeCN in water (0.1% NH4OH modifier)), the title compound (131.5 mg, 0.24 mmol, 48.7%, 3:1 d.r.) was obtained as a white solid. Diastereomers were separated using chiral HPLC (Enantiocel C8 (2 x 25 cm) column, 20% methanol (0.1% diethyl amine)/$CO_2$ (100 bar), 70 mL/min, 220 nm).

**$^1$H NMR (400 MHz, $CDCl_3$)** δ 8.69 (s, 1H), 7.42 – 7.27 (m, 5H), 5.07 (d, *J* = 9.8 Hz, 1H), 4.79 (dd, *J* = 8.0, 2.1 Hz, 1H), 4.69 – 4.47 (m, 1H), 4.45 – 4.21 (m, 1H), 4.15 (d, *J* = 9.4 Hz, 1H), 4.05 (t, *J* = 9.7 Hz, 1H), 3.95 (dd, *J* = 9.9, 7.9 Hz, 1H), 3.76 – 3.45 (m, 1H), 2.83 (ddd, *J* = 12.8, 6.8, 2.4 Hz, 1H), 2.52 (s, 3H), 2.28 – 2.10 (m, 1H), 1.42 (d, *J* = 7.3 Hz, 10H), 0.89 (d, *J* = 6.7 Hz, 9H). **(summary of diastereomers)**

**$^{13}$C NMR (126 MHz, $CDCl_3$)** δ 172.7, 169.4, 155.8, 150.5, 148.7, 137.7, 131.5, 129.8, 129.8, 129.7, 128.5, 128.4, 128.2, 118.5, 80.3, 59.1, 58.7, 58.7, 51.1, 50.2, 43.7, 43.4, 35.2, 31.1, 30.6, 29.4, 28.5, 28.4, 28.3, 28.1, 26.7, 26.4, 26.3, 16.2. **(summary of diastereomers)**

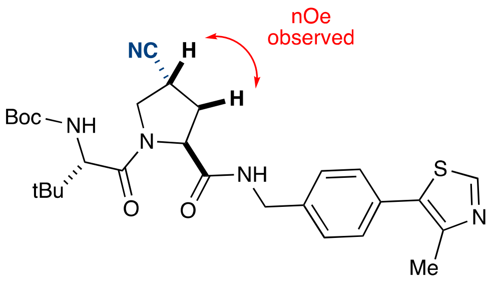


**Major:**

**$^1$H NMR (500 MHz, CDCl$_3$)** δ 8.67 (s, 1H), 7.39 – 7.29 (m, 5H), 5.09 (d, *J* = 9.5 Hz, 1H), 4.78 (dd, *J* = 8.0, 2.2 Hz, 1H), 4.54 (dd, *J* = 14.8, 6.8 Hz, 1H), 4.28 (dd, *J* = 14.8, 5.1 Hz, 1H), 4.15 (d, *J* = 9.5 Hz, 1H), 4.10 – 4.01 (m, 1H), 3.95 (t, *J* = 8.9 Hz, 1H), 3.56 (q, *J* = 8.5 Hz, 1H), 2.85 – 2.77 (m, 1H), 2.51 (s, 3H), 2.25 – 2.18 (m, 1H), 1.42 (s, 9H), 0.87 (s, 9H).

**$^{13}$C NMR (126 MHz, CDCl$_3$)** δ $^{13}$C NMR (126 MHz, CDCl$_3$) 172.60, 169.44, 155.75, 150.49, 150.45, 148.69, 137.65, 131.55, 131.41, 131.22, 129.76, 129.65, 128.36, 128.17, 118.46, 80.30, 58.69, 58.63, 50.19, 43.40, 35.22, 31.12, 31.06, 28.44, 28.37, 28.23, 26.29, 16.23, 16.19.

**IR (film)** $\nu_{max}$ 3311, 2959, 2248, 1638, 1500, 1417, 1391, 1365, 1325, 1237, 1163, 1057, 1007, 966, 935, 892, 829, 802, 697, 658, 613, 565, 550, 521, 486, 442.

**HRMS (ESI-TOF)** *m/z* calcd. for $C_{28}H_{38}SO_4N_5$ ($[M+H]^+$) 540.2639, found 540.2639.

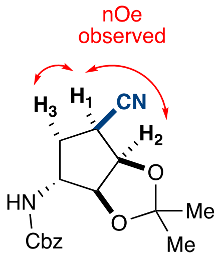


**Benzyl N-[rac-(3aS,4R,6aR)-6-cyano-2,2-dimethyl-4,5,6,6a-tetrahydro-3aH-cyclopenta[d][1,3]dioxol-4-yl]carbamate (45)**

The title compound was prepared according to **General procedure A**. 4-CzPN (7.9 mg, 0.01 mmol, 2 mol%), tosyl cyanide (135.9 mg, 0.75 mmol, 1.5 equiv.), benzoyl peroxide (121.1 mg, 0.5 mmol, 1.0 equiv.), 2,4,6-trimethylpyridine (198 µL, 1.5 mmol, 3.0 equiv.), acetone (15 mL) and $H_2O$ (1 mL) were added directly to the reaction vial. From activated alcohol solution:

5,7-ditert-butyl-3-(2-methoxyphenyl)-1,3-benzoxazol-3-ium; tetrafluoroborate (153.7 mg, 0.5 mmol, 1.0 equiv.), NHC-J (255.2 mg, 0.6 mmol, 1.2 equiv.), MTBE (15 mL) and pyridine (60 µL, 0.75 mmol, 1.5 equiv.).

The yield of the desired product (41% yield) was obtained via $^{1}$H NMR analysis (500 MHz, $CDCl_3$) using 1,3,5-trimethoxybenzene as an internal standard. NMR assay for product was assigned via diagnostic signals at δ 3.01 (d, $J$ = 10.2 Hz, 1H).

Spectral data used for assay assignment are consistent with the isolated pure product.

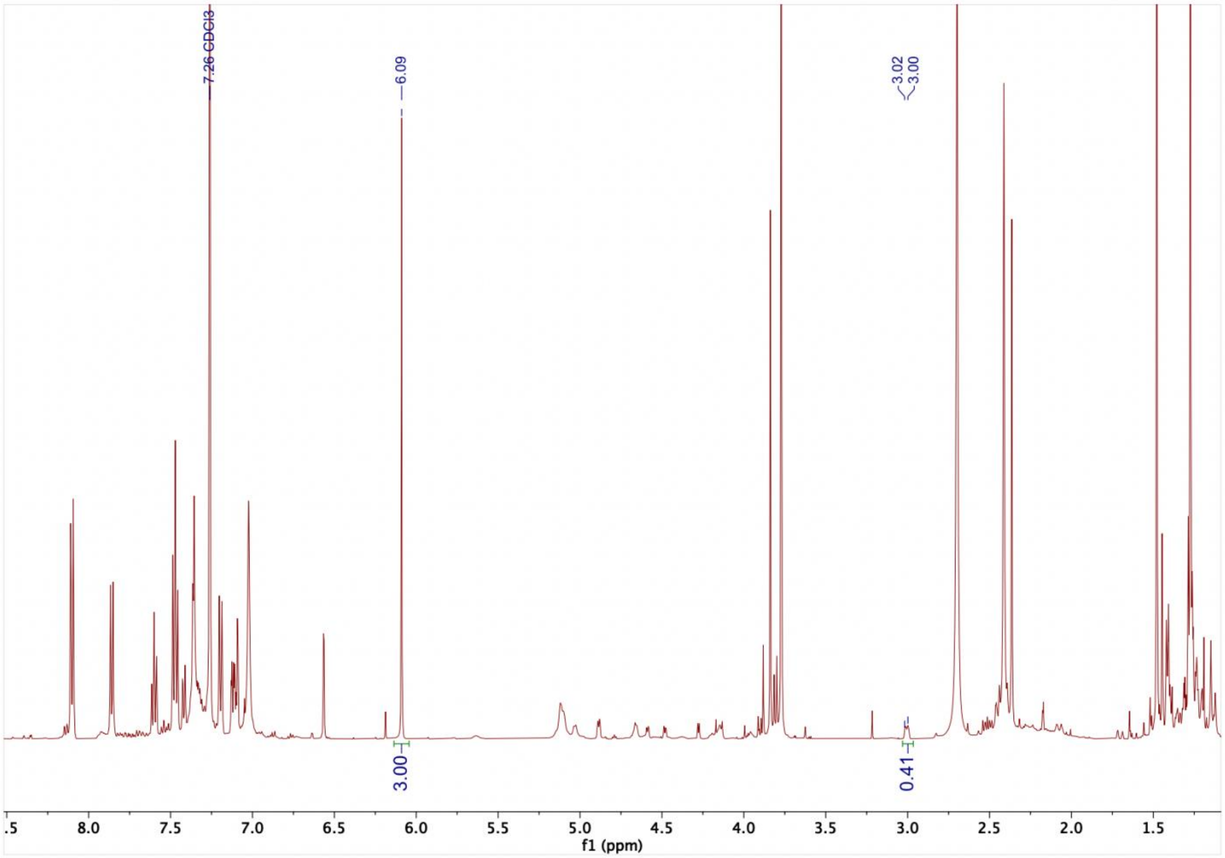


assay yield assignment of **45** via 1H NMR (500 MHz, CDCl3)

[Using the doublet at 3.01 ppm corresponding to 1H: 0.41*100% = 41% yield of **45**].

An analytical amount of pure product was obtained following the purification procedures below. The residue was purified by automated flash chromatography (40 g high performance silica column, 0-100% EtOAc in hexane). The product containing fractions were then combined and after a second purification via preparative HPLC (XBridge BEH C18 OBD column, 10-100%

MeCN in water (0.1% NH4OH modifier)). The product containing fractions were then combined and after a third purification via chiral HPLC (Enantiocel A6 (2 x 25 cm) column, 20% methanol(0.1% diethyl amine)/$CO_2$ (100 bar), 65 mL/min, 220 nm).

**$^{1}$H NMR (500 MHz, $CDCl_3$)** δ 7.35 (qq, *J* = 7.1, 2.7 Hz, 5H), 5.08 (s, 2H), 4.74 (t, *J* = 5.3 Hz, 1H), 4.57 (s, 1H), 3.94 (t, *J* = 5.6 Hz, 1H), 3.05 (dt, *J* = 12.2, 5.8 Hz, 1H), 2.36 (td, *J* = 13.2, 5.5 Hz, 1H), 2.17 – 1.97 (m, 1H), 1.51 (s, 3H), 1.31 (s, 3H).

**$^{13}$C NMR (126 MHz, $CDCl_3$)** δ 156.0, 136.1, 128.8, 128.5, 128.3, 118.2, 112.1, 85.0, 79.0, 67.3, 56.6, 33.5, 33.2, 26.1, 24.1.

**IR (film)** $\nu_{max}$ 3370, 2981, 2931, 2241, 1682, 1529, 1455, 1374, 1360, 1292, 1263, 1232, 1206, 1158, 1107, 1058, 1024, 969, 909, 864, 804, 778, 757, 738, 695, 589, 572, 513, 477.

**HRMS (ESI-TOF)** *m/z* calcd. for $C_{17}H_{21}N_2O_4$ ($[M+H]^+$) 317.1496, found 317.1495.

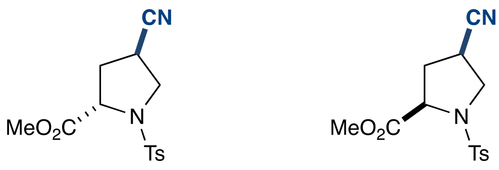


**major diastereomer** **minor diastereomer**

**(±)-Methyl-4-cyano-1-tosylpyrrolidine-2-carboxylate (46)**

The title compound was prepared according to **General procedure A**. 4-CzPN (7.9 mg, 0.01 mmol, 2 mol%), tosyl cyanide (135.9 mg, 0.75 mmol, 1.5 equiv.), benzoyl peroxide (121.1 mg, 0.5 mmol, 1.0 equiv.), 2,4,6-trimethylpyridine (198 µL, 1.5 mmol, 3.0 equiv.), acetone (15 mL) and $H_2O$ (1 mL) were added directly to the reaction vial. From activated alcohol solution: methyl 4-hydroxy-1-(p-tolylsulfonyl)pyrrolidine-2-carboxylate (149.7 mg, 0.5 mmol, 1.0 equiv.), NHC-J (255.2 mg, 0.6 mmol, 1.2 equiv.), MTBE (15 mL) and pyridine (60 µL, 0.75 mmol, 1.5 equiv.). The residue was purified by automated flash chromatography (40 g high performance silica column, 0-100% EtOAc in hexane). The product containing fractions were then combined and after a second purification via preparative HPLC (XBridge BEH C18 OBD column, 20-100% MeCN in water (0.1% NH4OH modifier)), the title compound (78.1 mg, 0.25 mmol, 50.7%) was obtained as a slightly yellow solid as a mixture of diastereomers (dr 2:1). The diastereomers were

separated via chiral HPLC (Enantiocel A6 (2×25 cm), 40% EtOH/$CO_2$ (100 bar), 65 ml/min, 220 nm).

**IR (film)** $\nu_{max}$ 3382, 2976, 2940, 2872, 2246, 1753, 1681, 1600, 1521, 1496, 1426, 1392, 1367, 1348, 1321, 1302, 1238, 1156, 1112, 1073, 1053, 1039, 997, 972, 947, 867, 854, 828, 812, 795, 769, 728, 724, 697, 636, 601, 573, 542, 486, 459.

**HRMS (ESI-TOF)** *m/z* calcd. for $C_{14}H_{17}N_2SO_4$ ($[M+H]^+$) 309.0904, found 309.0904.

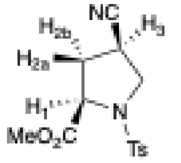


**Methyl (2*R*,4*R*)-4-cyano-1-tosylpyrrolidine-2-carboxylate (minor diastereomer)**

**$^1$H NMR (500 MHz, CDCl$_3$)** δ 7.76 (d, *J* = 8.0 Hz, 2H), 7.35 (d, *J* = 8.0 Hz, 2H), 4.50 (dd, *J* = 8.4, 6.1 Hz, 1H), 3.90 (dd, *J* = 10.9, 7.6 Hz, 1H), 3.72 (s, 3H), 3.54 (dd, *J* = 10.9, 8.0 Hz, 1H), 2.90 (p, *J* = 7.9 Hz, 1H), 2.60 (dt, *J* = 13.2, 8.2 Hz, 1H), 2.44 (s, 3H), 2.32 (ddd, *J* = 13.5, 7.9, 6.1 Hz, 1H).

**$^{13}$C NMR (126 MHz, CDCl$_3$)** δ 170.9, 144.7, 135.1, 130.11, 130.08, 127.7, 118.3, 59.8, 59.6, 52.9, 50.9, 34.5, 34.4, 27.6, 21.7.

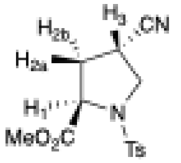


**Methyl (2*S*,4*R*)-4-cyano-1-tosylpyrrolidine-2-carboxylate (major diastereomer)**

**$^1$H NMR (500 MHz, CDCl$_3$)** δ 7.74 (d, *J* = 8.1 Hz, 2H), 7.35 (d, *J* = 8.1 Hz, 2H), 4.40 (dd, *J* = 8.7, 3.2 Hz, 1H), 3.85 (dd, *J* = 9.8, 7.6 Hz, 1H), 3.73 (s, 3H), 3.49 (dd, *J* = 9.8, 7.9 Hz, 1H), 3.31 (dq, *J* = 9.8, 7.6 Hz, 1H), 2.44 (s, 3H), 2.42 – 2.35 (m, 1H), 2.25 (dt, *J* = 13.2, 9.1 Hz, 1H).

**$^{13}$C NMR (126 MHz, CDCl$_3$)** δ 171.2, 144.7, 134.2, 130.13, 130.10, 129.7, 127.8, 127.6, 118.2, 118.2, 59.44, 59.37, 53.0, 50.6, 34.7, 34.6, 27.4, 21.7.

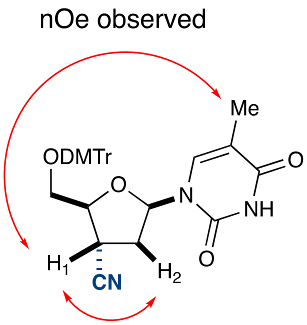


**(2*S*,3*R*,5*R*)-2-((bis(4-methoxyphenyl)(phenyl)methoxy)methyl)-5-(5-methyl-2,4-dioxo-3,4-dihydropyrimidin-1(2*H*)-yl)tetrahydrofuran-3-carbonitrile (47)**

Prepared following **General procedure A** with slight modification. 4-CzPN (7.9 mg, 0.01 mmol, 2 mol%), tosyl cyanide (135.9 mg, 0.75 mmol, 1.5 equiv.), benzoyl peroxide (121.1 mg, 0.5 mmol, 1.0 equiv.), 2,4,6-trimethylpyridine (198 µL, 1.5 mmol, 3.0 equiv.), acetone (15 mL) and $H_2O$ (1 mL) were added directly to the reaction vial. From activated alcohol solution, the following modifications were maded: 1-((2*R*,4*S*,5*R*)-5-((bis(4-methoxyphenyl)(phenyl)methoxy)methyl)-4-hydroxytetrahydrofuran-2-yl)-5-methylpyrimidine-2,4(1*H*,3*H*)-dione (272.3 mg, 0.5 mmol, 1.0 equiv.) was dissolved in **MTBE (10 ml)**. Pyridine (60 µL, 0.75 mmol, 1.5 equiv.) was then added to the solution dropwise. The mixture was allowed to stir for 5 minutes before NHC-J (155.2 mg, 0.6 mmol, 1.2 equiv.) was then added. The condensation was allowed to stir for 45 minutes before MTBE (5 ml) was added to wash the condensation vial and filtered into the reaction vial. The residue was concentrated, redissolved with EtOAc, washed with $NaHCO_3$, water, and brine. The organic layers were combined, dried over $Na_2SO_4$, concentrated, and purified by automated flash chromatography (40 g high performance silica column, 0-100% EtOAc/hexane gradient). The product containing fractions were then combined and concentrated. Title compound (149 mg, 0.27 mmol, 53.8%) was obtained as a yellow solid as a single diastereomer.

**$^{1}$H NMR (500 MHz, CDCl$_3$)** δ 9.26 (s, 1H), 7.47 (d, *J* = 1.4 Hz, 1H), 7.43 – 7.36 (m, 2H), 7.34 – 7.27 (m, 6H), 7.25 – 7.20 (m, 1H), 6.89 – 6.78 (m, 4H), 6.17 (dd, *J* = 7.1, 4.1 Hz, 1H), 4.29 (dt, *J* = 8.4, 2.9 Hz, 1H), 3.79 (s, 7H), 3.62 (dd, *J* = 11.1, 2.9 Hz, 1H), 3.49 (q, *J* = 8.8 Hz, 1H), 3.44 (dd, *J* = 11.1, 3.0 Hz, 1H), 2.76 (ddd, *J* = 13.8, 9.1, 7.1 Hz, 1H), 2.59 (ddd, *J* = 13.5, 8.8, 4.1 Hz, 1H), 1.56 (d, *J* = 1.3 Hz, 3H).

**$^{13}$C NMR (126 MHz, CDCl$_3$)** δ 163.9, 158.9, 150.2, 144.2, 135.2, 135.13, 135.11, 130.10, 130.08, 128.2, 128.1, 127.4, 118.2, 113.5, 111.5, 87.3, 85.5, 82.2, 61.9, 55.4, 36.9, 28.3, 12.2.

**IR (film)** $\nu_{max}$ 2930, 2835, 2338, 1684, 1606, 1507, 1463, 1445, 1247, 1174, 1113, 1067, 1029, 826, 755, 726, 700, 583.

**HRMS (ESI-TOF)** *m/z* calcd. for $C_{32}H_{31}N_3O_6Na^+$ ($[M+Na]^+$) 576.2105, found 576.2106.

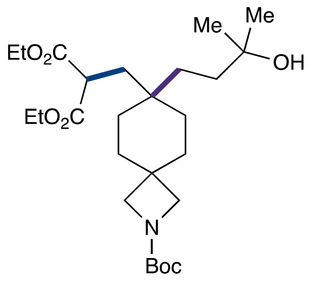


**Diethyl 2-((2-(*tert*-butoxycarbonyl)-7-(3-hydroxy-3-methylbutyl)-2-azaspiro[3.5]nonan-7-yl)methyl)malonate (48)**

This procedure was adapted from a known literature method.[5] To a 40 mL vial equipped with a cross- shaped stir bar was added, in order: *tert*-butyl 7-methylene-2-azaspiro[3.5]nonane-2-carboxylate (118.7 mg, 0.50 mmol, 1.00 equiv.), 4CzPN (3.9 mg, 0.005 mmol, 0.01 equiv.), Ni(TMHD)$_2$ (53.2 mg, 0.125 mmol, 0.25 equiv.), diethyl 2-chloromalonate (243.3 mg, 1.25 mmol, 2.50 equiv.), CsOAc (287.9 mg, 1.50 mmol, 3.00 equiv.), TBACl (83.4 mg, 0.300 mmol, 0.6 equiv.), and *tert*-amyl alcohol (5 mL). This vial was placed under nitrogen.

To a separate 40 mL vial equipped with a cross-shaped stir bar was added NHC-*p*CF3 (602.2 mg, 1.30 mmol, 2.60 equiv.) and anhydrous TBME (5 mL). 3-Methyl-1,3-butanediol was then added (133.4 µL, 130.2 mg, 1.25 mmol, 2.50 equiv.) and the suspension was sonicated

for 15 minutes. Pyridine (104.7 μL, 1.30 mmol, 2.60 equiv.) was then added, and the suspension was stirred at room temperature under nitrogen atmosphere. After 15 minutes, the resulting suspension was transferred through a syringe filter into the vial containing the other reaction components.

The reaction mixture was then sparged with nitrogen for 15 minutes. The vial was then sealed with parafilm and placed in a PennPhD m1 integrated photoreactor. The reaction was irradiated with 450 nm light for 16 hours (m1 450 nm LED plate, 5% intensity, 5200 rpm fans, 1000 rpm stirring), after which solvents were removed via rotary evaporation. The crude residue was then purified by automated flash chromatography (50 g high performance silica column, 0-80% EtOAc/hexanes gradient) followed by preparative HPLC (XBridge BEH C18 OBD column, 40-75% MeCN/$H_2O$ with 0.1% $NH_4OH$) to afford the title compound as a yellow oil (206.7 mg, 0.427 mmol, 85.5% yield).

**$^1$H NMR (500 MHz, $CDCl_3$)** δ 4.29 – 4.11 (m, 4H), 3.57 (d, *J* = 4.0 Hz, 4H), 3.36 (t, *J* = 5.9 Hz, 1H), 1.98 (d, *J* = 6.0 Hz, 2H), 1.73 – 1.56 (m, 5H), 1.46 (s, 9H), 1.41 – 1.32 (m, 4H), 1.29 (t, *J* = 7.2 Hz, 7H), 1.25 (q, *J* = 2.9 Hz, 3H), 1.22 (s, 6H).

**$^{13}$C NMR (126 MHz, $CDCl_3$)** δ 170.5, 156.7, 79.3, 70.7, 61.8, 47.5, 36.5, 34.9, 34.0, 32.1, 31.2 29.5, 28.6, 14.1.

**IR (film)** $\nu_{max}$ 3458, 2974, 2932, 2871, 1750, 1731, 1702, 1454, 1406, 1366, 1250, 1146, 1035, 913, 862, 772, 744, 647, 561 cm$^{-1}$.

**HRMS (ESI-TOF)** *m/z* calcd. for $C_{26}H_{45}NNaO_7^+$ ([M+Na]$^+$) 506.3088, found 506.3088.

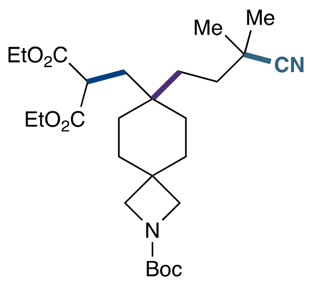


**Diethyl 2-((2-(*tert*-butoxycarbonyl)-7-(3-cyano-3-methylbutyl)-2-azaspiro[3.5]nonan-7-yl)methyl)malonate (49)**

The title compound was prepared according to a modified **General procedure B** on a 0.4 mmol scale. 4-CzPN (6.3 mg, 8 µmol, 2 mol%), tosyl cyanide (108.73 mg, 0.6 mmol, 1.5 equiv.), benzoyl peroxide (96.89 mg, 0.4 mmol, 1.0 equiv.), 2,4,6-trimethylpyridine (158.7 µL, 1.45 mmol, 3.0 equiv.), acetone (12 mL) and $H_2O$ (0.8 mL) were added directly to the reaction vial. From activated alcohol solution: (diethyl 2-((2-(*tert*-butoxycarbonyl)-7-(3-hydroxy-3-methylbutyl)-2-azaspiro[3.5]nonan-7-yl)methyl)malonate (193.5 mg, 0.4 mmol, 1.0 equiv.), NHC-5 (240.9 mg, 0.52 mmol, 1.3 equiv.), TFT (12 mL) and pyridine (48.3 µL, 0.6 mmol, 1.5 equiv.). The residue was purified by automated flash chromatography (40 g high performance silica column, 0-100% EtOAc in hexane). The product containing fractions were then combined and after a second purification via preparative HPLC (XBridge BEH C18 OBD column, 55-70% MeCN in water (0.1% NH4OH modifier)), the title compound (111.3 mg, 0.25 mmol, 63.7%) was obtained as a light yellow oil.

**$^1$H NMR (500 MHz, $CDCl_3$)** δ 4.17 (p, $J$ = 7.1 Hz, 4H), 3.53 (d, $J$ = 1.3 Hz, 4H), 3.27 (t, $J$ = 5.9 Hz, 1H), 1.94 (d, $J$ = 5.9 Hz, 2H), 1.62 (qt, $J$ = 13.5, 5.9 Hz, 4H), 1.41 (s, 13H), 1.31 (s, 6H), 1.24 (q, $J$ = 6.7 Hz, 10H).

**$^{13}$C NMR (126 MHz, $CDCl_3$)** δ 170.1, 156.6, 124.9, 79.3, 61.8, 60.1, 58.8, 47.3, 34.8, 34.3, 34.1, 32.4, 31.8, 31.1, 28.5, 26.8, 14.1.

**IR (film)** $\nu_{max}$ 2976, 2932, 2871, 2233, 1729, 1697, 1456, 1393, 1366, 1273, 1247, 1145, 1027, 731.

**HRMS (ESI-TOF)** *m*/*z* calcd. for $C_{27}H_{44}N_2O_6Na^+$ ($[M+Na]^+$) 515.3092, found 515.3097.

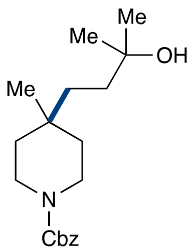


**Benzyl 4-(3-hydroxy-3-methylbutyl)-4-methylpiperidine-1-carboxylate (50)**

This procedure was adapted from a known literature method.[6]

**Alcohol solution (activated alcohol):** A 40-mL vial was charged with NHC-1 (628 mg, 1.59 mmol, 3.3 equiv.), and an X-shaped stir bar. The vial was evacuated and back-filled with $N_2$ (3 x 1 min). Then, 3-methylbutane-1,3-diol (150.5 mg, 1.44 mmol, 3 equiv) and MTBE (3 mL) were added via syringe under nitrogen. This mixture was stirred, and pyridine (122 µL, 1.52 mmol, 3.15 equiv) was added slowly via syringe. The nitrogen line was then removed, and the vial was sealed with parafilm. The heterogeneous mixture was vigorously stirred at room temperature for 30–45 minutes. During this time, a white precipitate formed and the mixture appeared either white or pink.

**Reaction vial**: A second 40-mL vial was charged with Mn(dpm)$_3$ (58.3 mg, 0.096 mmol, 20 mol%), potassium acetate (23.6 mg, 0.24 mmol, 0.5 equiv), benzoyl peroxide (350 mg, 1.44 mmol, 3 equiv), (Ir(dF(CF$_3$)ppy)$_2$(dtbbpy)PF$_6$ (1.35 mg, 1.2 µmol, 0.25 mol%), water (44 µL, 2.4 mmol, 5 equiv), Ni(acac)$_2$ (12.4 mg, 0.048 mmol, 10 mol%), and extra dry DMA (1.5 mL) under air.

Upon completion of the NHC condensation, filtered **Alcohol solution** and the **Reaction Vial** were sparged with $N_2$ at the headspace for 1-2 min. Then, the Reaction Vial was charged with filtered Alcohol solution (3 mL), benzyl 4-methylenepiperidine1-carboxylate (100 µL, 0.482 mmol, 1 equiv), and 1,1,3,3-tetramethyldisiloxane (0.43 mL, 2.4 mmol, 5 equiv). The reaction mixture was subjected to 450 nm IPR irradiation (50% LED intensity, 500 rpm stir rate, maximum fan speed) for 2 hours.

After this time, 1,4-dinitrobenzene was added as an internal standard, and an aliquot was removed for NMR analysis. Subsequently, the reaction mixture was poured into 15 mL of 10 wt% aqueous LiCl solution and transferred to a separatory funnel. The reaction vial was rinsed into the separatory funnel with $Et_2O$ (3 x 10 mL), and the layers separated. The aqueous layer was extracted with $Et_2O$ (5 x 40 mL). The combined extracts were washed with saturated $NaHCO_3$ solution and brine, dried over $Na_2SO_4$, filtered, and concentrated in vacuo. The crude reaction mixture was dissolved in minimal diethyl ether and hexane, loaded on a 50 g Biotage Sfär Silica normal phase column/40 g RediSepSilver normal column and purified with a slow gradient from 0-30% and a faster gradient from 30-100% EtOAc in hexanes. The resulting product fractions were directly concentrated and subjected to further purification by preparative reverse phase HPLC with 45-80%

MeCN in water (0.1% $NH_4OH$ modifier). Product containing fractions were concentrated, back extracted with $Et_2O$ five times, and washed with brine to provide the desired product as a colorless oil (91.3 mg, 0.286 mmol, 57% yield).

Spectral data are consistent with those reported in literature: Cai, Q.; McWhinnie, I. M.; Dow, N. W.; Chan, A. Y.; MacMillan, D. W. C. *J. Am. Chem. Soc.* **2024**, *146*, 12300-12309.

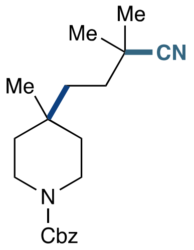


**Benzyl 4-(3-cyano-3-methylbutyl)-4-methylpiperidine-1-carboxylate (51)**

The title compound was prepared according to a modified **General procedure B** on a 0.28 mmol scale. 4-CzPN (4.4 mg, 5.6 µmol, 2 mol%), tosyl cyanide (76.1 mg, 0.42 mmol, 1.5 equiv.), benzoyl peroxide (67.8 mg, 0.28 mmol, 1.0 equiv.), 2,4,6-trimethylpyridine (111.1 µL, 0.84 mmol, 3.0 equiv.), acetone (8.4 mL) and $H_2O$ (0.56 mL) were added directly to the reaction vial. From activated alcohol solution: benzyl 4-(3-hydroxy-3-methyl-butyl)-4-methyl-piperidine-1-carboxylate (89.4 mg, 0.28 mmol, 1.0 equiv.), NHC-5 (168.6 mg, 0.36 mmol, 1.3 equiv.), TFT (8.4 mL) and pyridine (33.8 µL, 0.42 mmol, 1.5 equiv.). The residue was purified by automated flash chromatography (40 g high performance silica column, 0-50% EtOAc in hexane). The product containing fractions were then combined and after a second purification via preparative HPLC (XBridge BEH C18 OBD column, 45-65% MeCN in water (0.1% $NH_4OH$ modifier)), the title compound (51.0 mg, 0.155 mmol, 55.5%) was obtained as a light yellow oil.

**$^1$H NMR (500 MHz, $CDCl_3$)** δ 7.35 (d, $J$ = 4.4 Hz, 4H), 7.31 (h, $J$ = 4.2 Hz, 1H), 5.12 (s, 2H), 3.67 (dt, $J$ = 12.5, 5.2 Hz, 2H), 3.27 (ddd, $J$ = 13.5, 9.3, 3.7 Hz, 2H), 1.58 – 1.38 (m, 7H), 1.34 (s, 8H), 0.95 (s, 3H).

**$^{13}$C NMR (126 MHz, $CDCl_3$)** δ 155.5, 137.1, 128.6, 128.1, 128.0, 125.0, 67.1, 40.2, 37.0, 36.8, 34.8, 32.5, 31.2, 26.8, 23.1.

**IR (film)** $\nu_{max}$ 2929, 2872, 2233, 1694, 1429, 1276, 1242, 1171, 1090, 1023, 734, 697.

**HRMS (ESI-TOF)** *m*/*z* calcd. for $C_{20}H_{29}N_2O_2^+$ ($[M+H]^+$) 329.2224, found 329.2228.

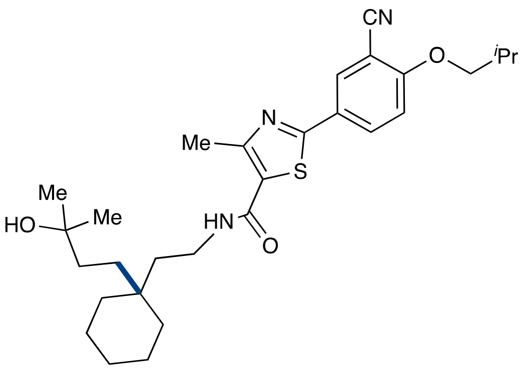


**2-(3-cyano-4-isopropoxyphenyl)-*N*-(2-(1-(3-hydroxy-3-methylbutyl)cyclohexyl)ethyl)-4-methylthiazole-5-carboxamide (52)**

This procedure was adapted from a known literature method.[6]

__Alcohol solution (activated alcohol):__ A 40-mL vial was charged with NHC-1 (652 mg, 1.65 mmol, 3.3 equiv.), and an X-shaped stir bar. The vial was evacuated and back-filled with $N_2$ (3 x 1 min). Then, 3-methylbutane-1,3-diol (156 mg, 1.5 mmol, 3 equiv) and MTBE (3 mL) were added via syringe under nitrogen. This mixture was stirred, and pyridine (127 µL, 1.58 mmol, 3.15 equiv) was added slowly via syringe. The nitrogen line was then removed, and the vial was sealed with parafilm. The heterogeneous mixture was vigorously stirred at room temperature for 30–45 minutes. During this time, a white precipitate formed and the mixture appeared either white or pink.

__Reaction vial__: A second 40-mL vial was charged with $Mn(dpm)_3$ (60.5 mg, 0.1 mmol, 20 mol%), potassium acetate (24.5 mg, 0.25 mmol, 0.5 equiv), benzoyl peroxide (363 mg, 1.5 mmol, 3 equiv), $(Ir(dF(CF_3)ppy)_2(dtbbpy)PF_6$ (1.4 mg, 1.25 µmol, 0.25 mol%), 2-(3cyano-4-isobutoxyphenyl)-N-(2-(cyclohex-1-en-1-yl)ethyl)-4-methylthiazole-5carboxamide (212 mg, 0.5 mmol, 1 equiv), water (45 µL, 2.5 mmol, 5 equiv), $Ni(acac)_2$ (12.9 mg, 0.05 mmol, 10 mol%), and extra dry DMA (1.5 mL) under air.

Upon completion of the NHC condensation, filtered **Alcohol solution** and the **Reaction Vial** were sparged with $N_2$ at the headspace for 1-2 min. Then, the Reaction Vial was charged with filtered Alcohol solution (3 mL) and 1,1,3,3-tetramethyldisiloxane (0.43 mL, 2.4 mmol, 5 equiv). The reaction mixture was subjected to 450 nm IPR irradiation (50% LED intensity, 500 rpm stir rate, maximum fan speed) for 6 hours.

After this time, 1,4-dinitrobenzene was added as an internal standard, and an aliquot was removed for NMR analysis. Subsequently, the reaction mixture was poured into 15 mL of 10 wt% aqueous LiCl solution and transferred to a separatory funnel. The reaction vial was rinsed into the separatory funnel with $Et_2O$ (3 x 10 mL), and the layers separated. The aqueous layer was extracted with $Et_2O$ (5 x 40 mL). The combined extracts were washed with saturated $NaHCO_3$ solution and brine, dried over $Na_2SO_4$, filtered, and concentrated in vacuo. The crude reaction mixture was dissolved in minimal diethyl ether and hexane, loaded on a 50 g Biotage Sfär Silica normal phase column/40 g RediSepSilver normal column and purified with a slow gradient from 0-60% and a faster gradient from 60-85% EtOAc in hexanes. The resulting product fractions were directly concentrated and subjected to further purification by preparative reverse phase HPLC with 45-80% MeCN in water (0.1% $NH_4OH$ modifier). Product containing fractions were concentrated, back extracted with $Et_2O$ five times, and washed with brine to provide the desired product as a colorless oil (143.5 mg, 0.280 mmol, 56.1% yield).

Spectral data are consistent with those reported in literature: Cai, Q.; McWhinnie, I. M.; Dow, N. W.; Chan, A. Y.; MacMillan, D. W. C. *J. Am. Chem. Soc.* **2024**, *146*, 12300-12309.

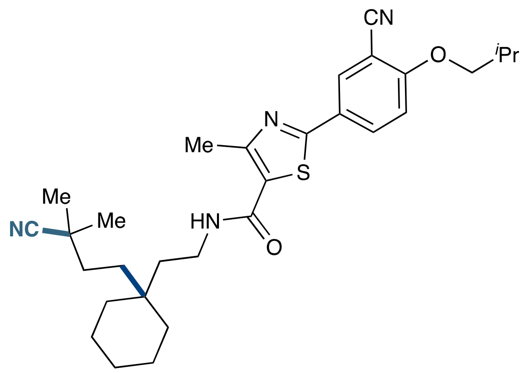


***N*-(2-(1-(3-cyano-3-methylbutyl)cyclohexyl)ethyl)-2-(3-cyano-4-isopropoxyphenyl)-4-methylthiazole-5-carboxamide (53)**

The title compound was prepared according to a modified **General procedure B** on a 0.25 mmol scale. 4-CzPN (3.9 mg, 5.0 μmol, 2 mol%), tosyl cyanide (67.95 mg, 0.375 mmol, 1.5 equiv.), benzoyl peroxide (60.56 mg, 0.25 mmol, 1.0 equiv.), 2,4,6-trimethylpyridine (99.22 μL, 0.75 mmol, 3.0 equiv.), acetone (7.5 mL) and $H_2O$ (0.5 mL) were added directly to the reaction vial. From activated alcohol solution: 2-(3-cyano-4-isopropoxyphenyl)-*N*-(2-(1-(3-hydroxy-3-methylbutyl)cyclohexyl)ethyl)-4-methylthiazole-5-carboxamide (127.9 mg, 0.25 mmol, 1.0 equiv.), NHC-5 (150.6 mg, 0.325 mmol, 1.3 equiv.), TFT (7.5 mL) and pyridine (30.2 μL, 0.98 mmol, 1.5 equiv.). The residue was purified by automated flash chromatography (40 g high performance silica column, 0-50% EtOAc in hexane). The product containing fractions were then combined and after a second purification via preparative HPLC (XBridge BEH C18 OBD column, 60-80% MeCN in water (0.1% $NH_4OH$ modifier)), the title compound (66.5 mg, 0.128 mmol, 51.1%) was obtained as a clear oil.

**$^1$H NMR (500 MHz, $CDCl_3$)** δ 8.10 (d, *J* = 2.3 Hz, 1H), 8.04 (dd, *J* = 8.9, 2.2 Hz, 1H), 6.99 (d, *J* = 8.9 Hz, 1H), 5.88 (t, *J* = 5.8 Hz, 1H), 3.88 (d, *J* = 6.4 Hz, 2H), 3.44 – 3.29 (m, 2H), 2.70 (d, *J* = 2.7 Hz, 3H), 2.19 (dh, *J* = 13.3, 6.6 Hz, 1H), 1.63 – 1.54 (m, 2H), 1.49 (d, *J* = 13.3 Hz, 8H), 1.41 (d, *J* = 6.1 Hz, 2H), 1.37 (s, 6H), 1.36 – 1.28 (m, 4H), 1.08 (d, *J* = 6.6 Hz, 6H).

**$^{13}$C NMR (126 MHz, $CDCl_3$)** δ 164.5, 162.4, 161.7, 155.9, 132.6, 132.0, 126.3, 126.1, 125.4, 115.6, 112.8, 103.0, 75.8, 36.6, 35.9, 35.7, 34.6, 34.5, 32.7, 32.2, 28.3, 26.8, 26.3, 21.6, 19.2, 17.5.

**IR (film)** $\nu_{max}$ 2928, 2868, 2231, 1640, 1607, 1508, 1434, 1368, 1607, 1508, 1434, 1368, 1278, 1169, 1128, 1063, 1010, 594, 553, 503.

**HRMS (ESI-TOF)** *m*/*z* calcd. for $C_{30}H_{40}N_4NaO_2S^+$ ($[M+Na]^+$) 543.2764, found 543.2767.

## 9) Reaction Scale-Up

To demonstrate the scalability of this reaction, compound **13** was prepared on a 1 mmol scale. To accommodate the use of a 40 mL vial in the integrated photoreactor, the reaction concentration was increased twofold.

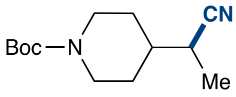


**(±)-*Tert*-butyl 4-(1-cyanoethyl)piperidine-1-carboxylate**

The title compound was prepared according to a modified General procedure A on 1 mmol scale, doubling the concentration. 4-CzPN (15.8 mg, 0.02 mmol, 2 mol%), tosyl cyanide (271.8 mg, 1.5 mmol, 1.5 equiv.), benzoyl peroxide (242.2 mg, 1.0 mmol, 1.0 equiv.), 2,4,6-trimethylpyridine (397 µL, 3.0 mmol, 3.0 equiv.), acetone (15 mL) and $H_2O$ (1 mL) were added directly to the reaction vial. From activated alcohol solution: tert-butyl 4-(1-hydroxyethyl)piperidine-1-carboxylate (229.3 mg, 1.0 mmol, 1.0 equiv.), NHC-J (510.3 mg, 1.2 mmol, 1.2 equiv.), MTBE (15 mL) and pyridine (121 µL, 1.5 mmol, 1.5 equiv.). The residue was purified by automated flash chromatography (40 g high performance silica column, 0-100% EtOAc in hexane). The product containing fractions were then combined and after a second purification via preparative HPLC (XBridge BEH C18 OBD column, 20-100% MeCN in water (0.1% NH4OH modifier), the title compound (126.7 mg, 0.53 mmol, 53.1 %) was obtained as a white solid.

## 10) References


1. Dong, Z.; MacMillan, D. W. C. *Nature* **2021**, *598*, 451–456.
2. Pangborn, A. B.; Giardello, M. A.; Grubbs, R. H.; Rosen, R. K.; Timmers, F. J. *Organometallics* **1996,** *15*, 1518-1520.
3. Le, C. C.; Wismer, M. K.; Shi, Z.-C.; Zhang, R.; Conway, D. V.; Li, G.; Vachal, P.; Davies, I. W.; MacMillan, D. W. C. *ACS Cent. Sci*. **2017**, *3,* 647–653.
4. Ripenko, V.; Vysochyn, D.; Klymov, I.; Zhersh, S.; Mykhailiuk, P. K., *J. Org. Chem.* **2021,** *86*, 14061-14068.
5. Wang, J. Z.; Lyon, W. L.; MacMillan, D. W. C. *Nature* **2024**, 628, 104-109.
6. Cai, Q.; McWhinnie, I. M.; Dow, N. W.; Chan, A. Y.; MacMillan, D. W. C. *J. Am. Chem. Soc*. **2024**, *146*, 12300-12309.

## 11) Spectral Data for Isolated Products

### ***Tert*-butyl 6-hydroxy-6-methyl-2-azaspiro[3.3]heptane-2-carboxylate (S1)**

$^1$H NMR (400 MHz, $CDCl_3$)

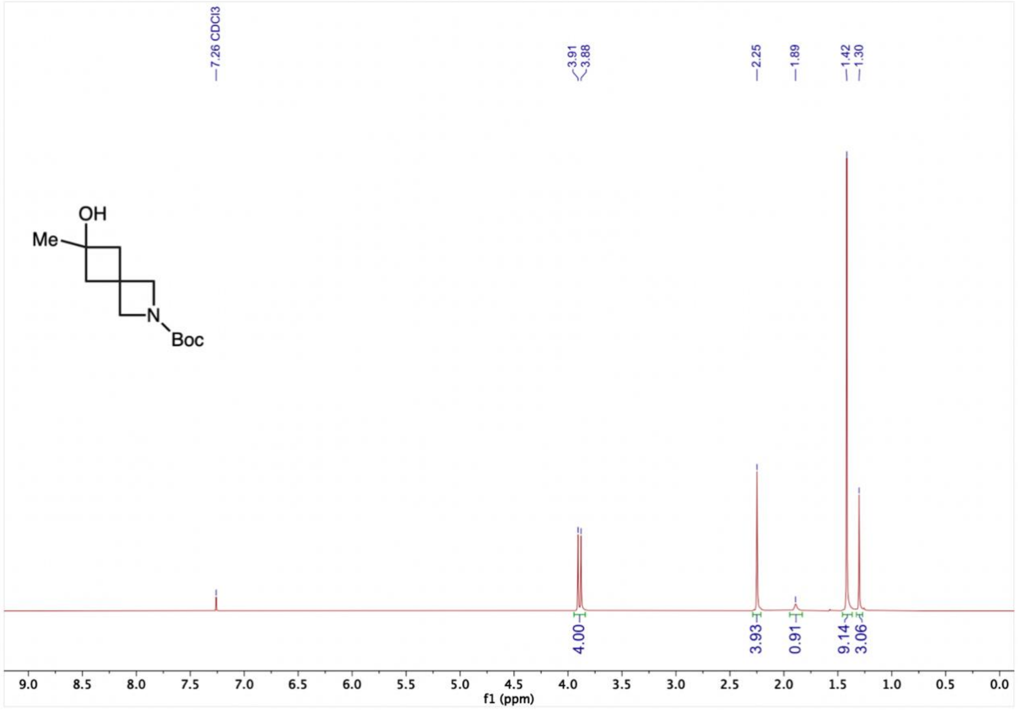


$^{13}$C NMR (126 MHz, $CDCl_3$)

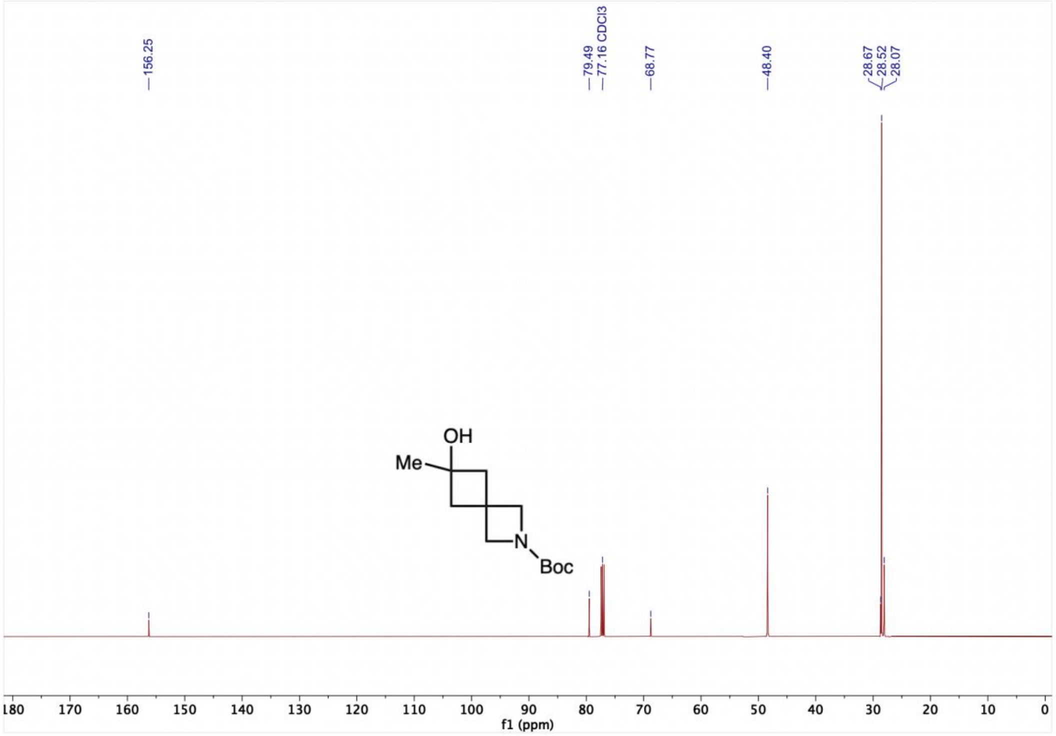

**(±)-*Tert*-butyl 7-hydroxy-7-methyl-5-oxa-2-azaspiro[3.4]octane-2-carboxylate (S2)**

$^{1}$H NMR (400 MHz, $CDCl_3$)

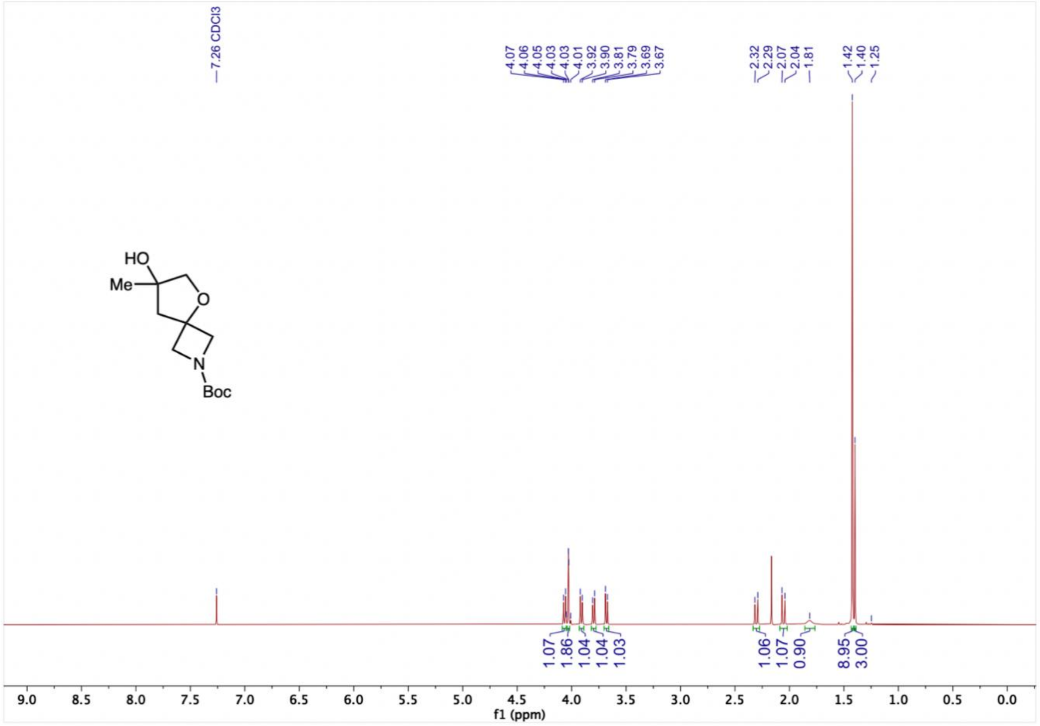


$^{13}$C NMR (126 MHz, $CDCl_3$)

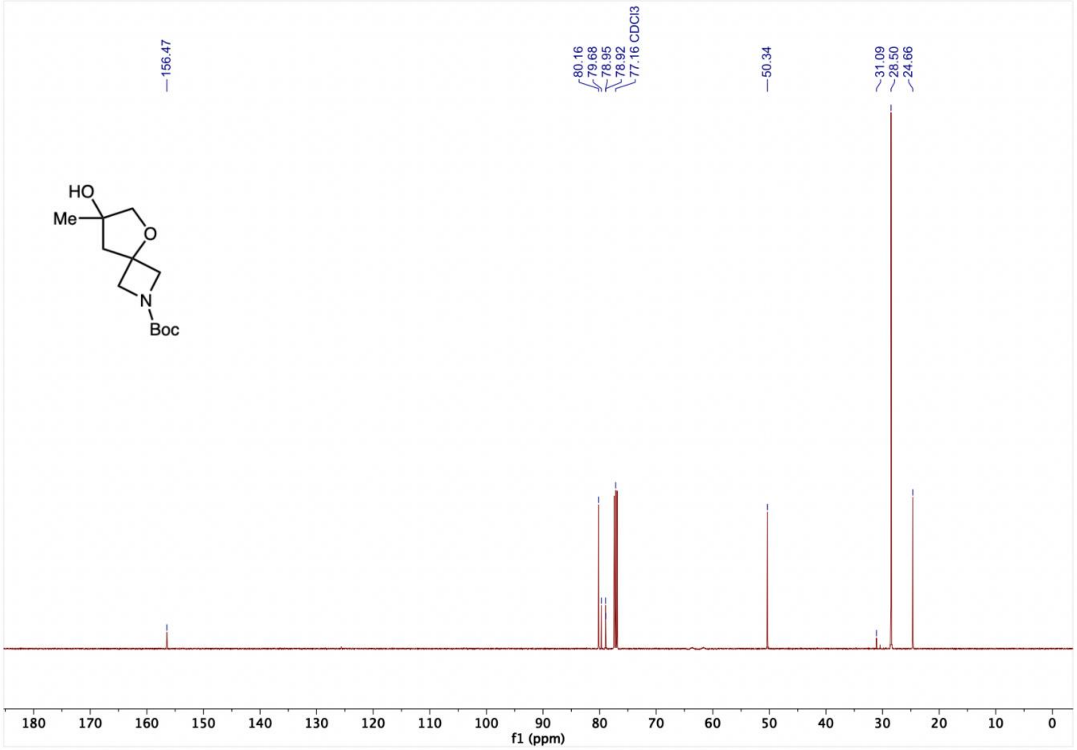

**Benzyl 5-hydroxy-5-methyl-2-azabicyclo[2.2.1]heptane-2-carboxylate (S4)**

$^{1}$H NMR (400 MHz, $CDCl_3$)

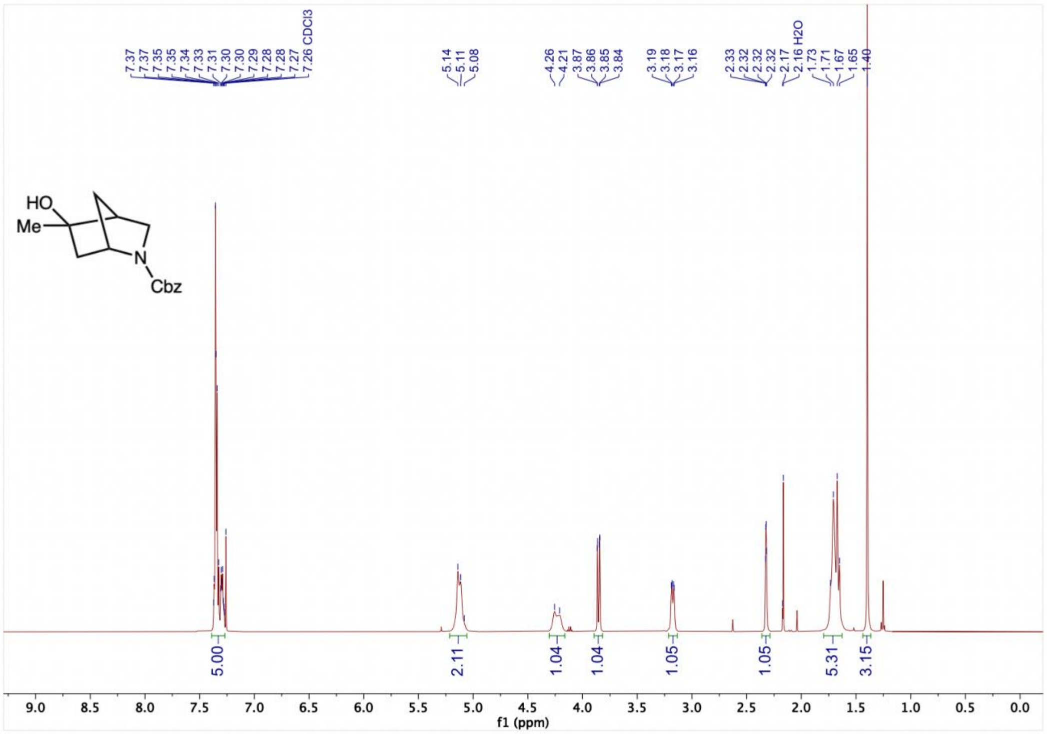


$^{13}$C NMR (126 MHz, $CDCl_3$)

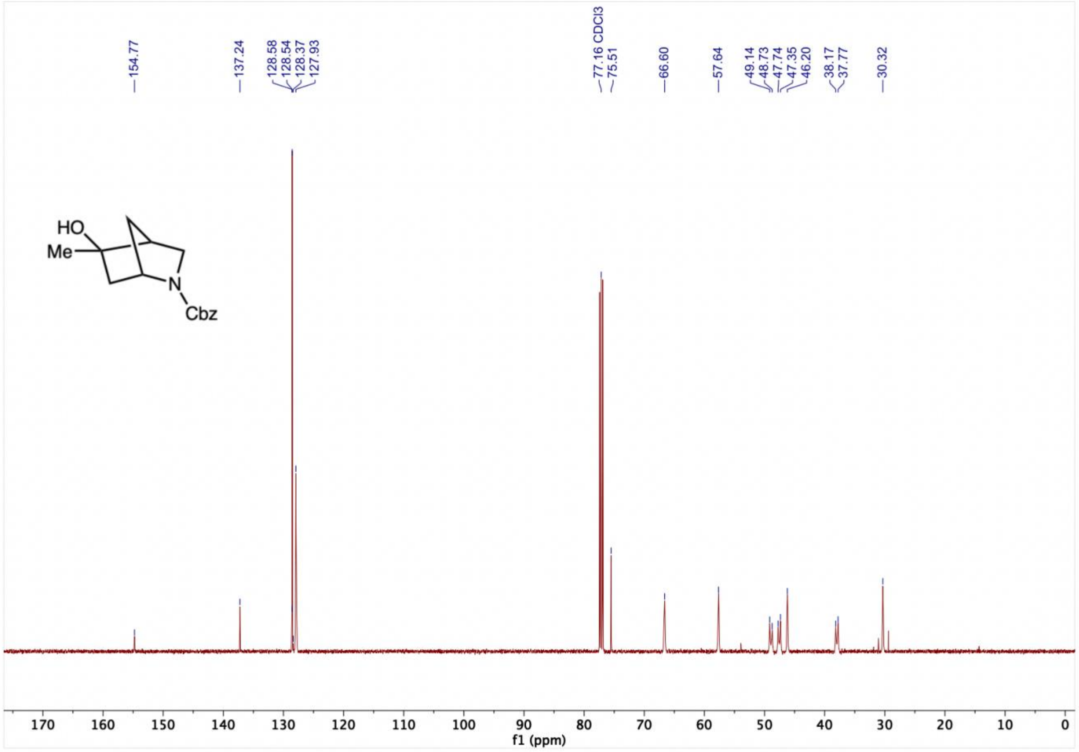

***Tert*-butyl ((*S*)-2-((1*S*,3*S*,5*S*)-3-cyano-2-azabicyclo[3.1.0]hexan-2-yl)-1-((1*r*,3*R*,5*R*,7*S*)-3-hydroxyadamantan-1-yl)-2-oxoethyl)carbamate (S5)**

$^1$H NMR (400 MHz, $CDCl_3$)

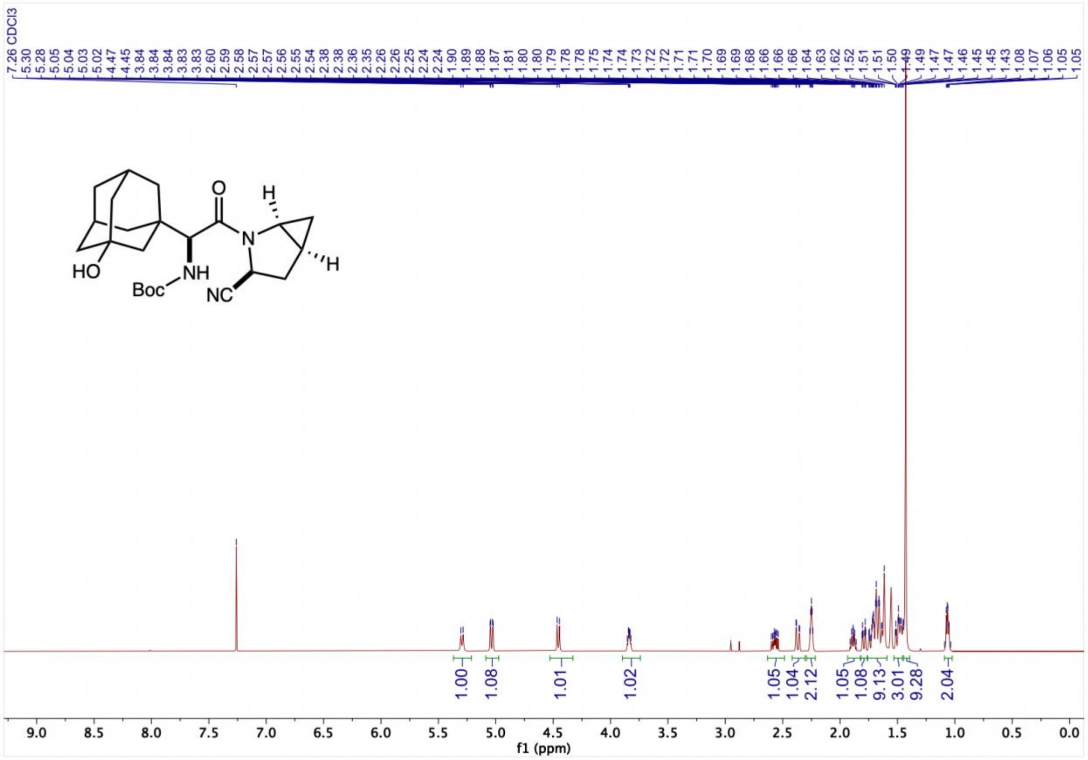


$^{13}$C NMR (126 MHz, $CDCl_3$)

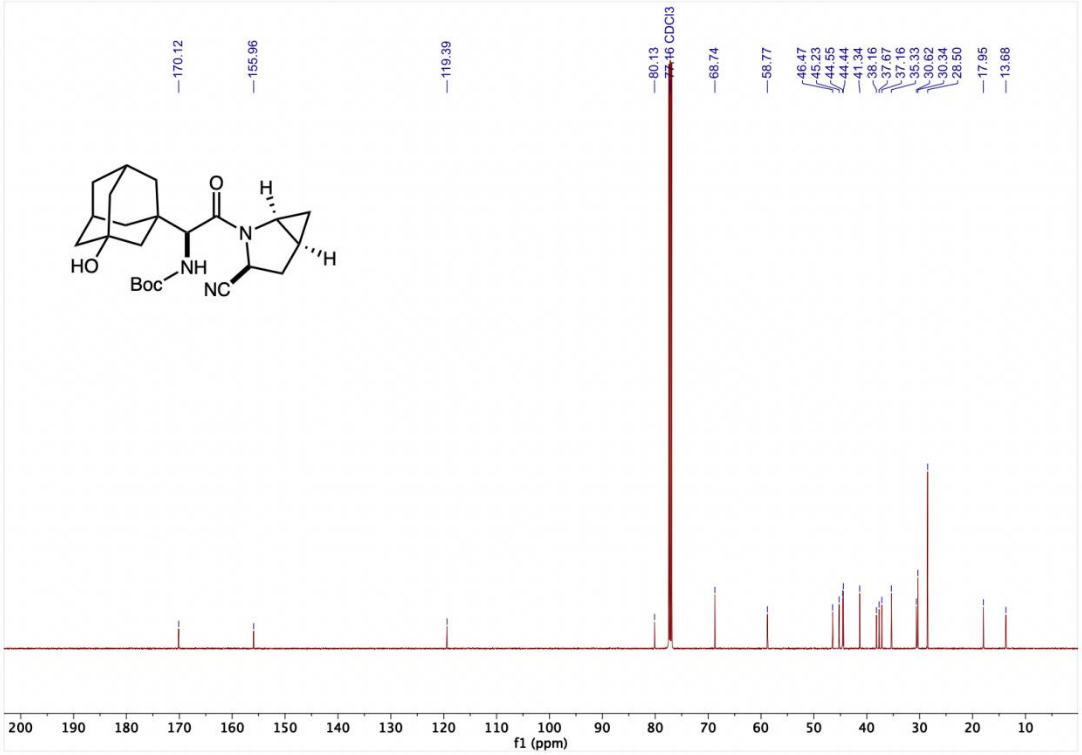

**1-(3-hydroxypyrrolidin-1-yl)-2-(4-isobutylphenyl)propan-1-one (S6)**

$^1H$ NMR (500 MHz, $CDCl_3$)

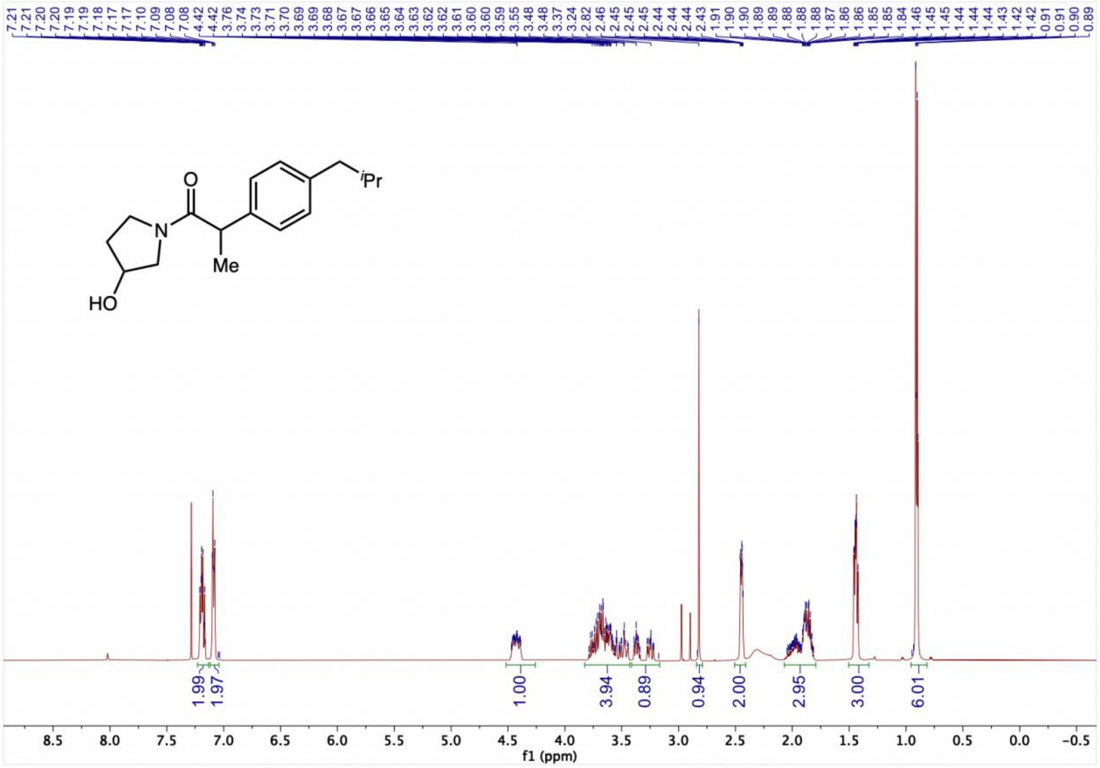

$^{13}C$ NMR (126 MHz, $CDCl_3$)

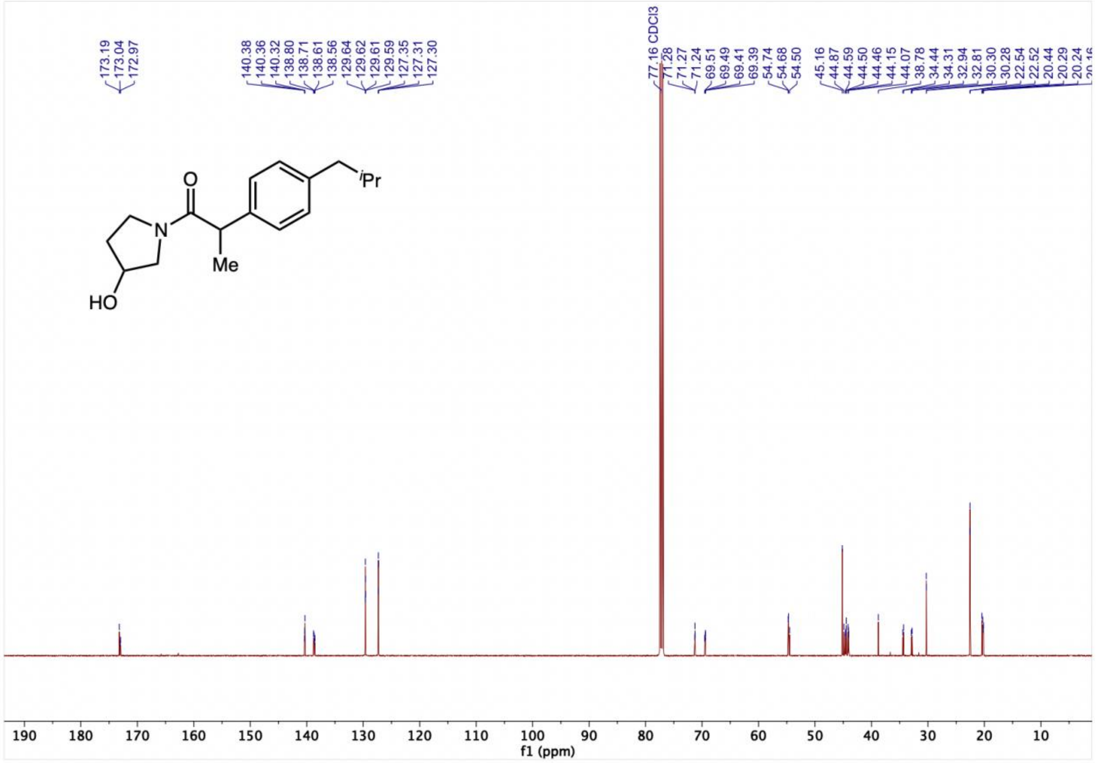

**(*S*)-2-((4,6-dimethylpyrimidin-2-yl)oxy)-*N*-((1*r*,3*R*,5*R*,7*S*)-3-hydroxyadamantan-1-yl)-3-methoxy-3,3-diphenylpropanamide (S7)**

$^{1}$H NMR (400 MHz, $CDCl_3$)

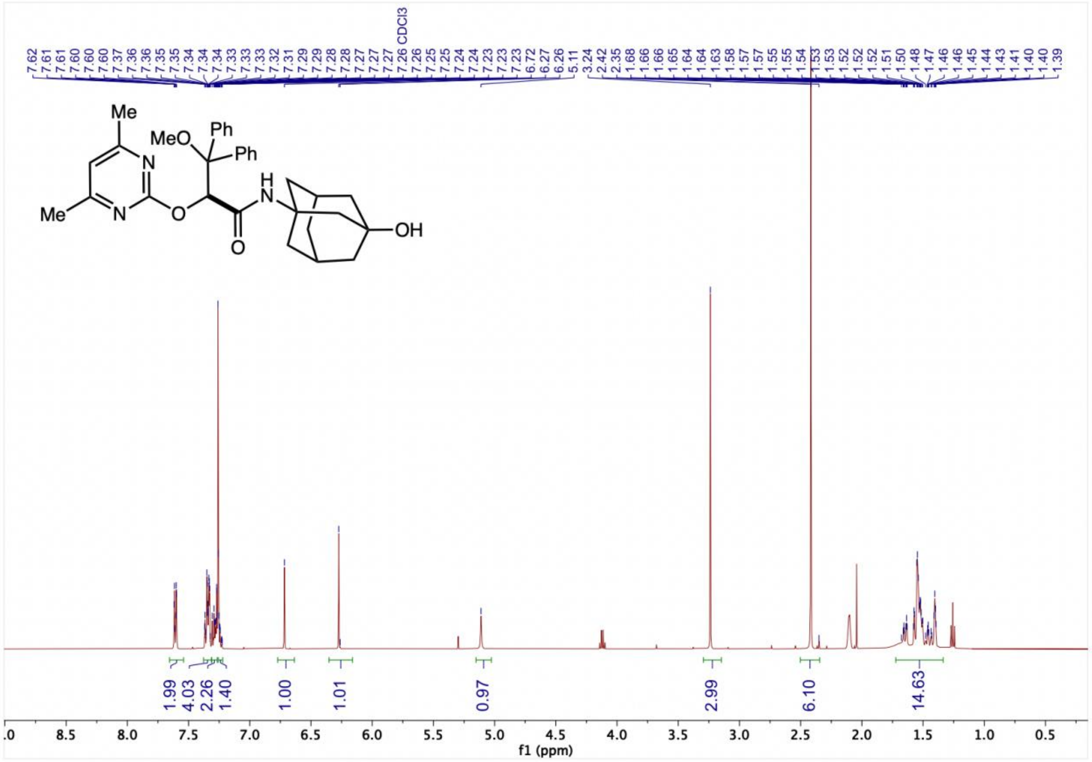


$^{13}$C NMR (126 MHz, $CDCl_3$)

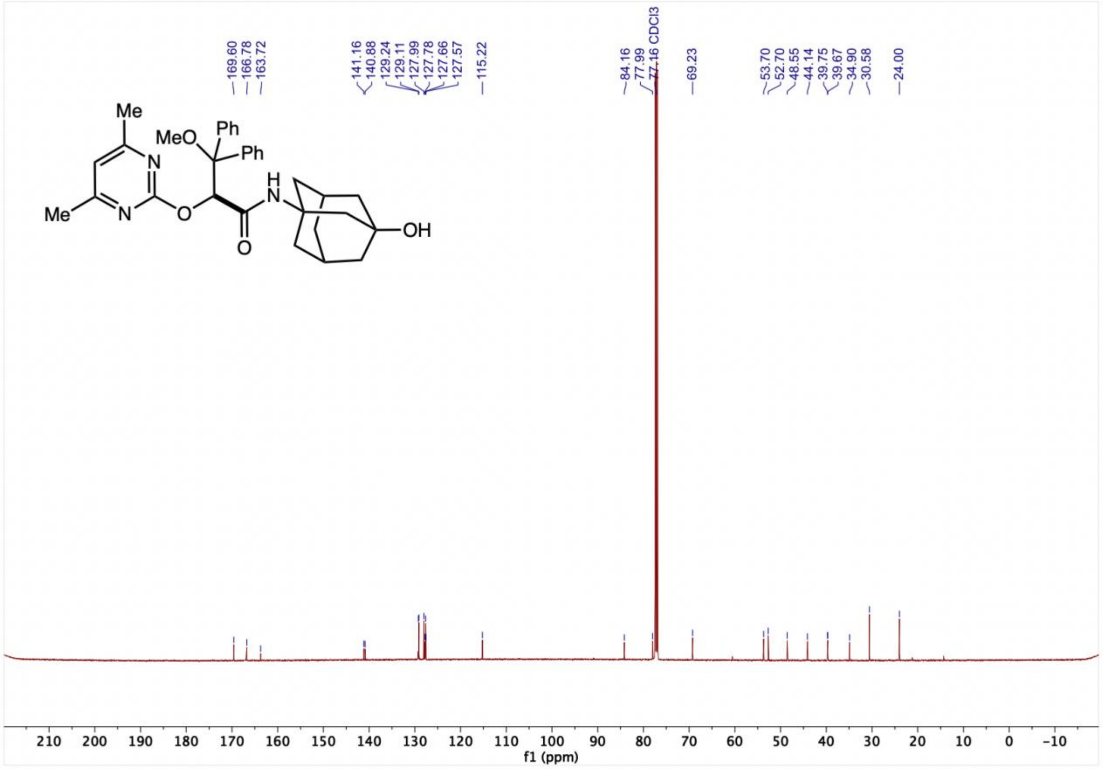

## ***Tert*-butyl 7-methylene-2-azaspiro[3.5]nonane-2-carboxylate (S8)**

$^{1}$H NMR (400 MHz, $CDCl_3$)

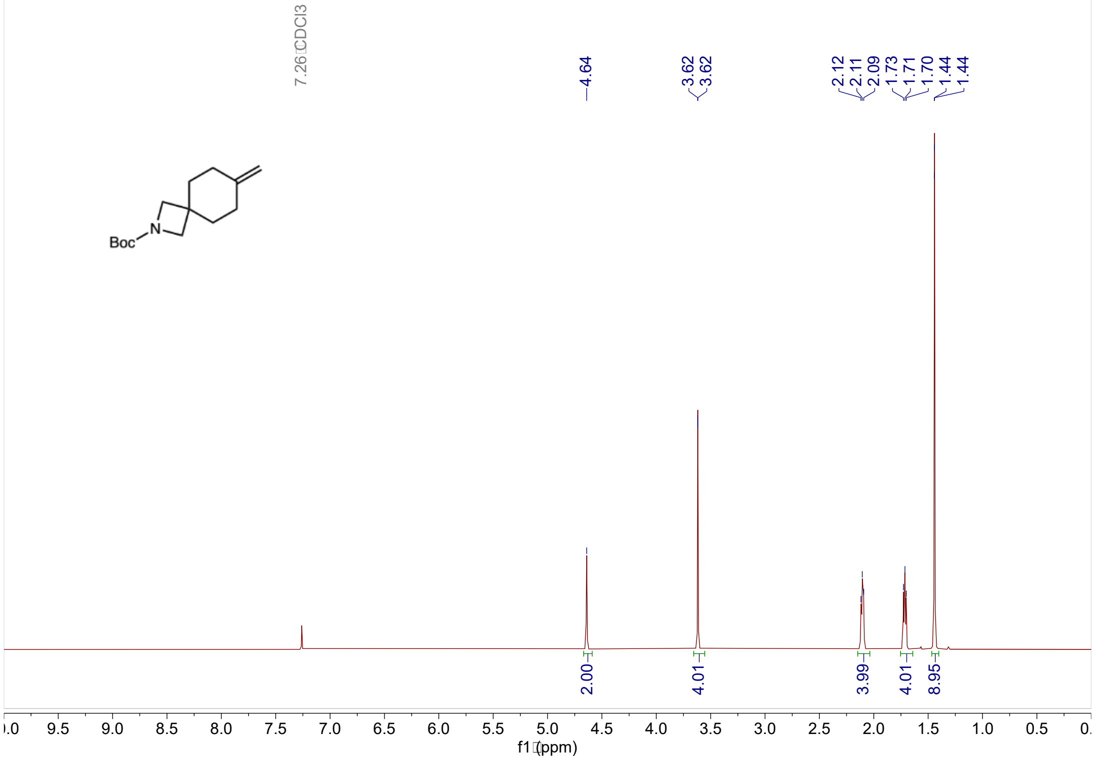


$^{13}$C NMR (126 MHz, $CDCl_3$)

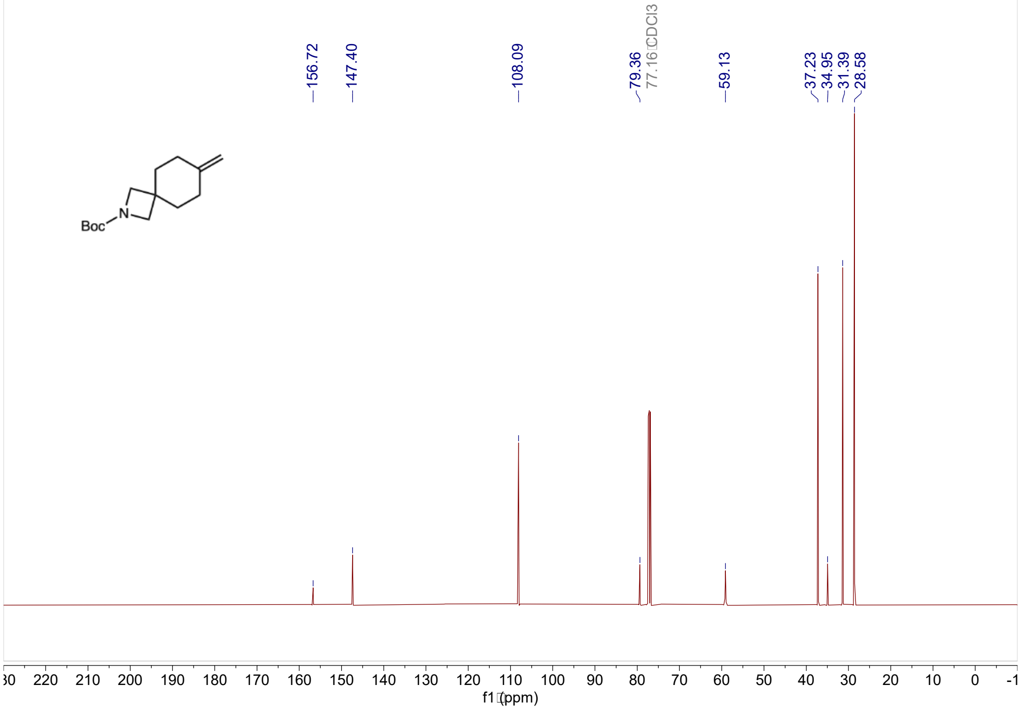

## (±)-Benzyl-3-cyanopyrrolidine-1-carboxylate (10)

$^{1}H$ NMR (400 MHz, $CDCl_3$)

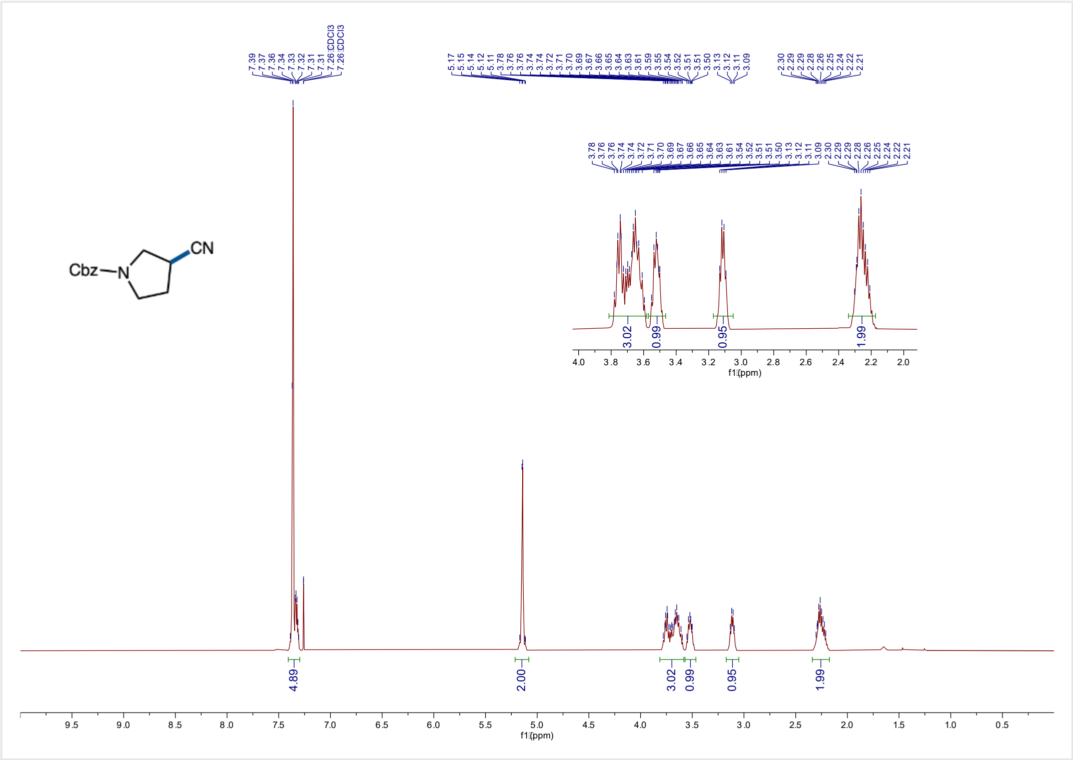

$^{13}C$ NMR (126 MHz, $CDCl_3$)

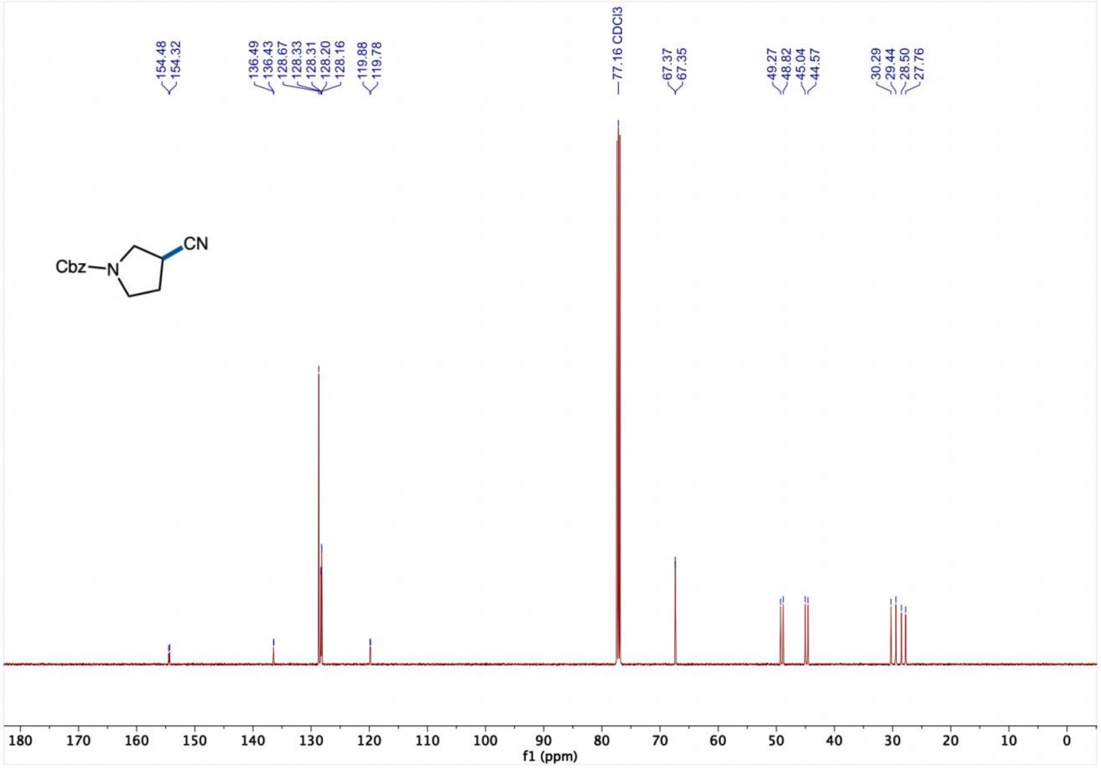

## 2-phenylcyclopentane-1-carbonitrile (11)

$^{1}$H NMR (400 MHz, $CDCl_3$)

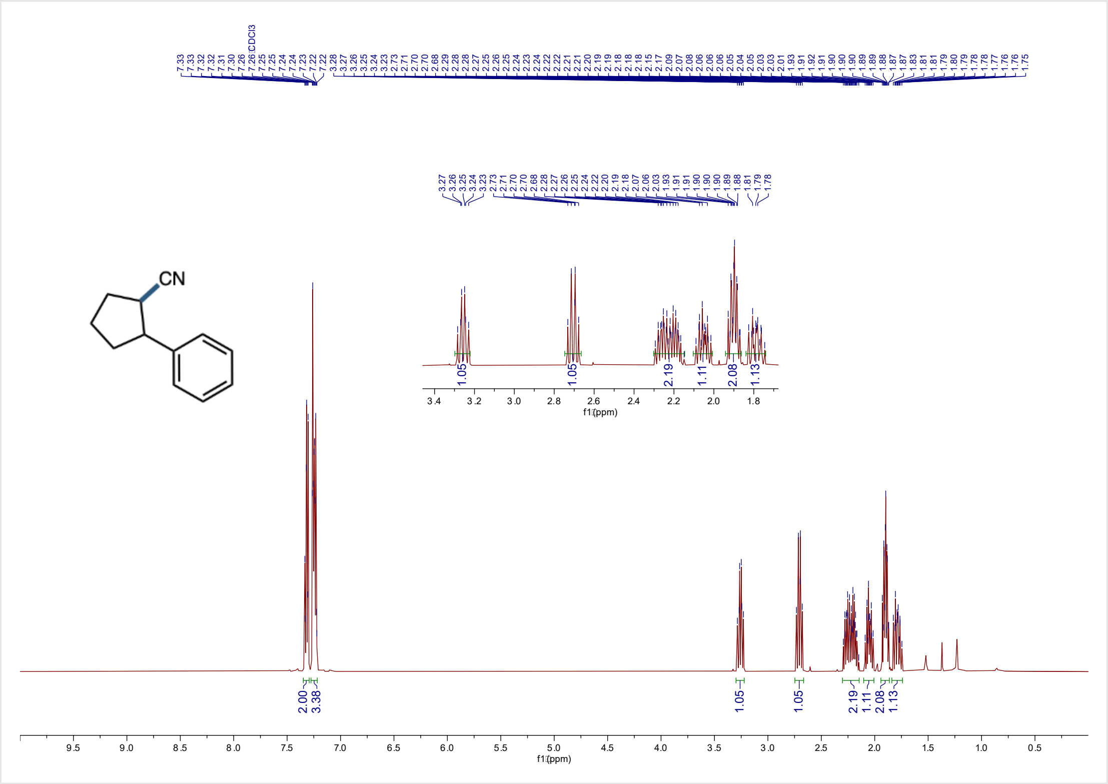

$^{13}$C NMR (126 MHz, $CDCl_3$)

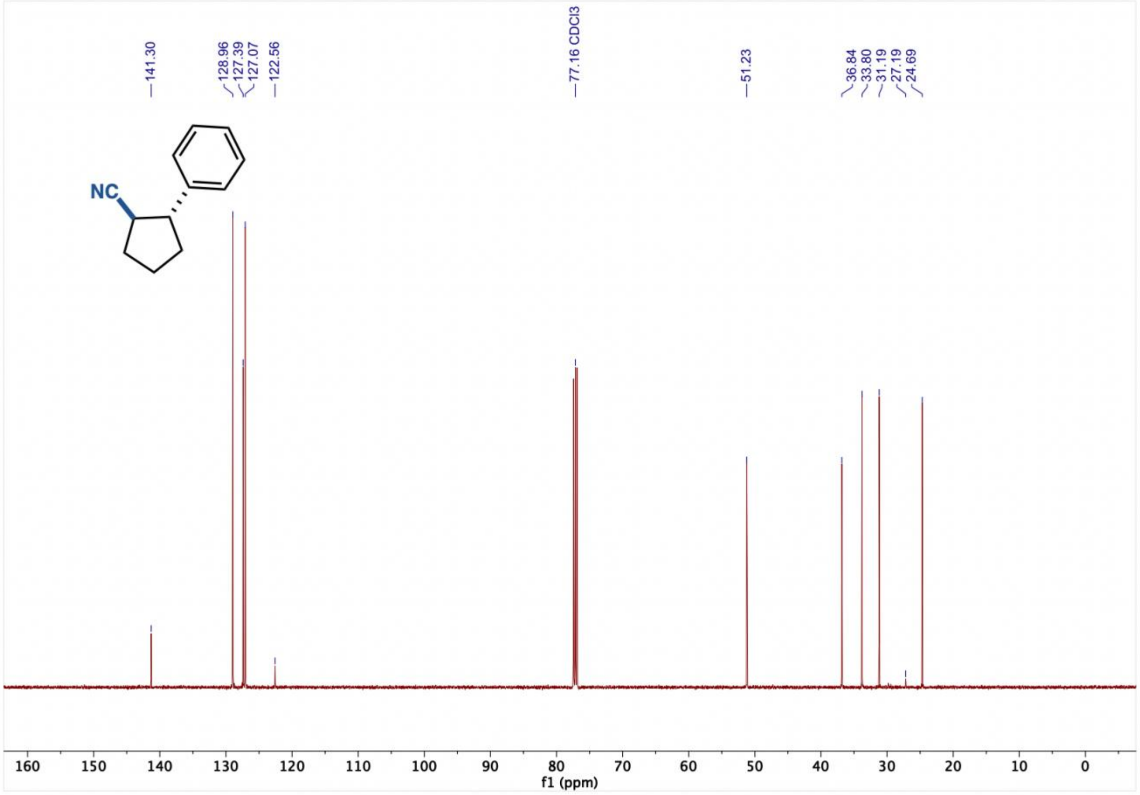

## (±)-*Tert*-butyl-4-cyanoazepane-1-carboxylate (12)

$^1$H NMR (400 MHz, $CDCl_3$)

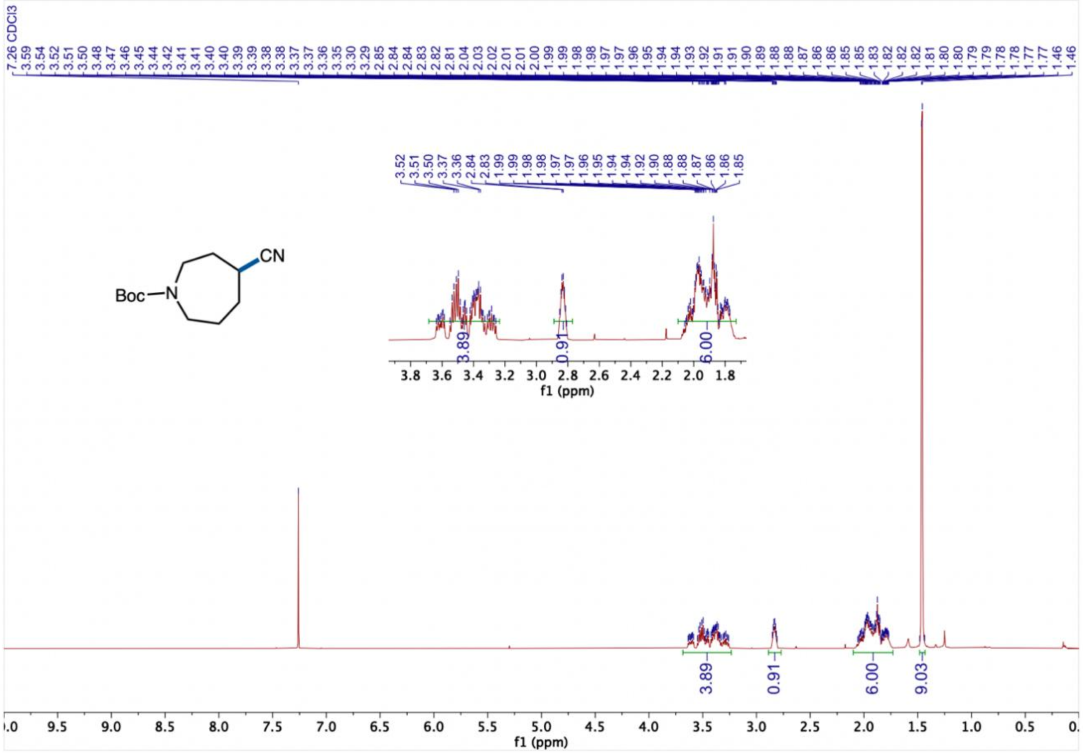


$^{13}$C NMR (126 MHz, $CDCl_3$)

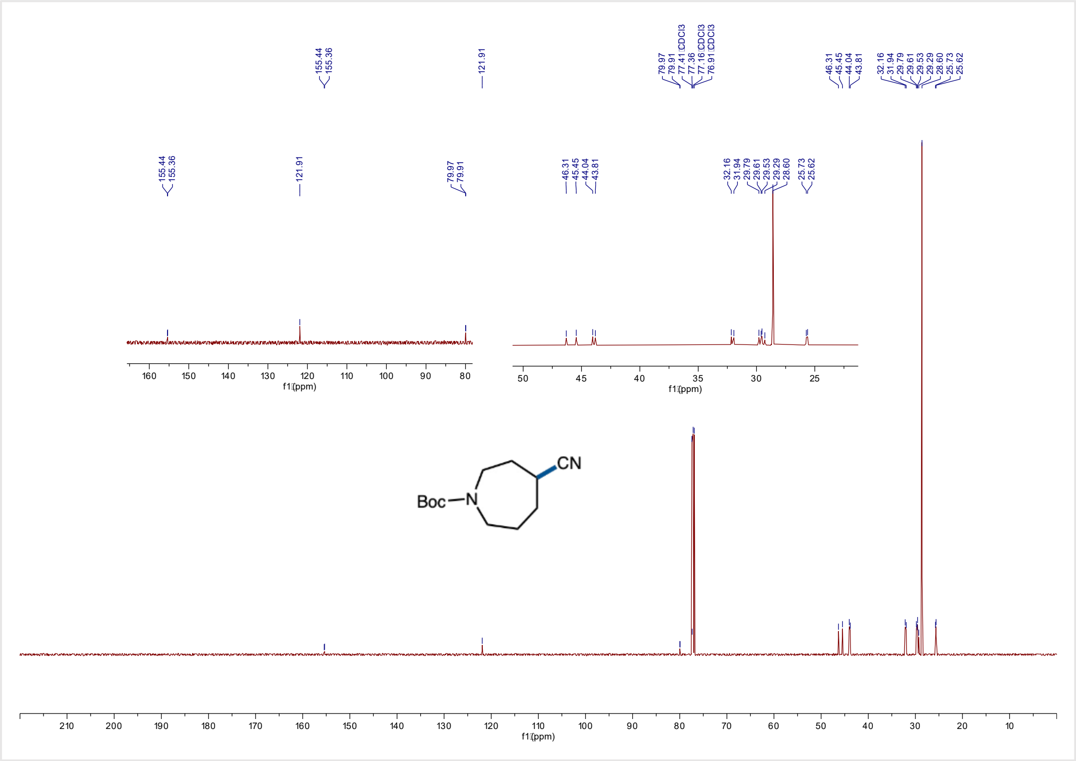

## (±)-*Tert*-butyl 4-(1-cyanoethyl)piperidine-1-carboxylate (13)

$^{1}H$ NMR (400 MHz, $CDCl_3$)

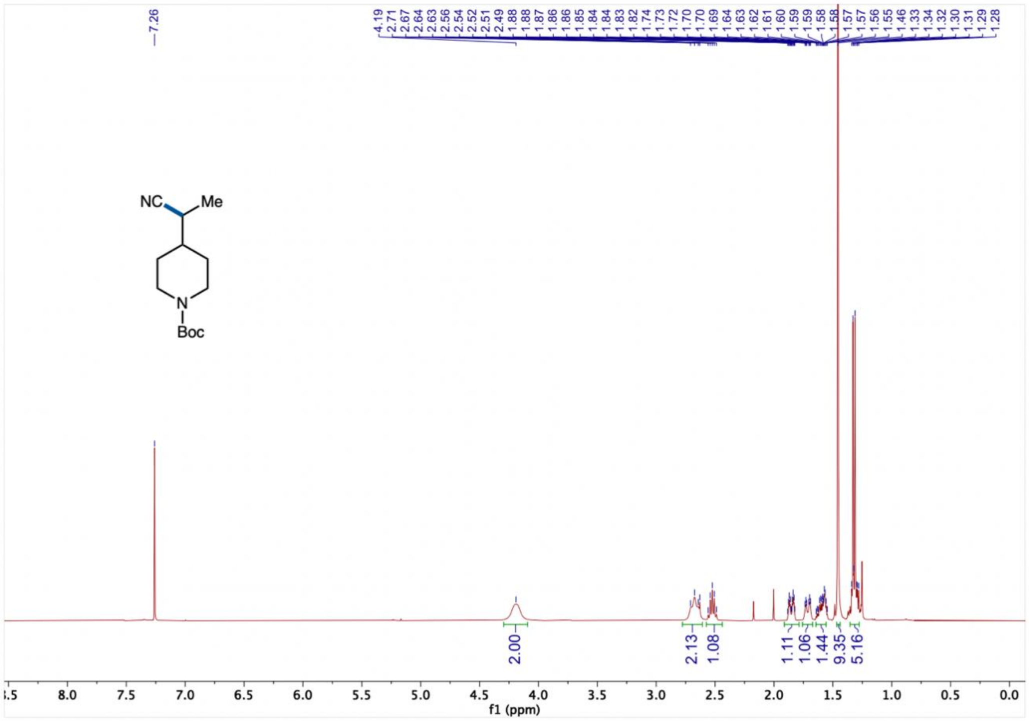


$^{13}C$ NMR (126 MHz, $CDCl_3$)

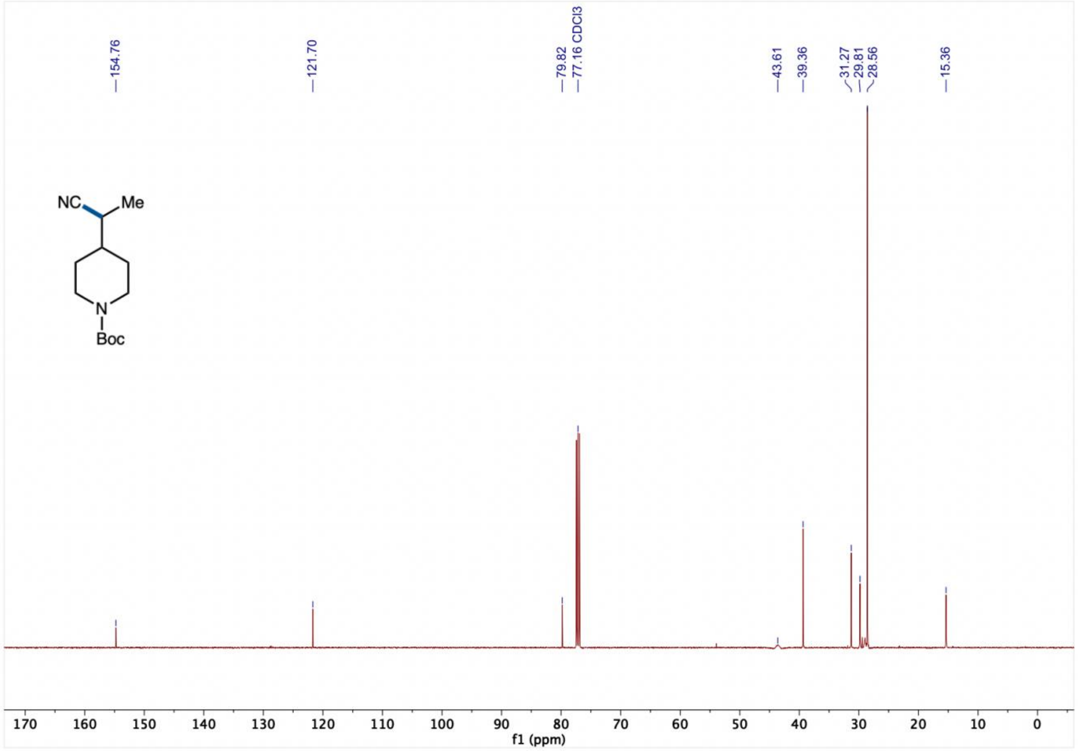

## (±)-2-methyl-4-phenyl-butanenitrile (14)

$^{1}$H NMR (400 MHz, $CDCl_3$)

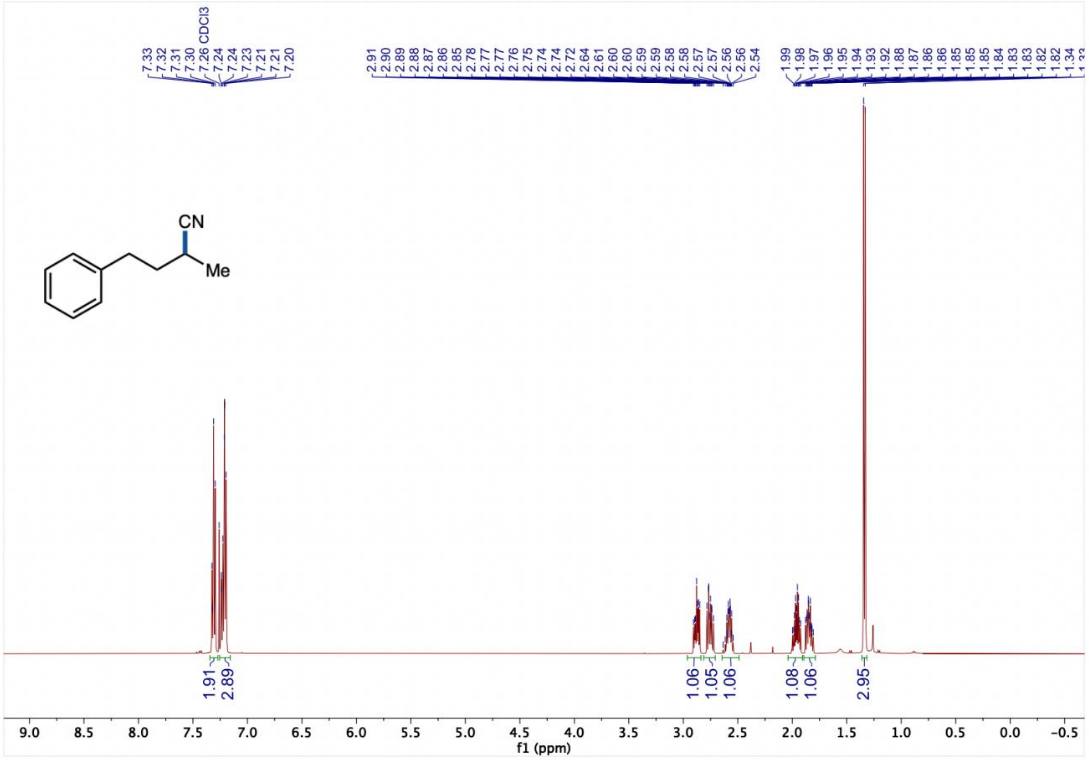

$^{13}$C NMR (126 MHz, $CDCl_3$)

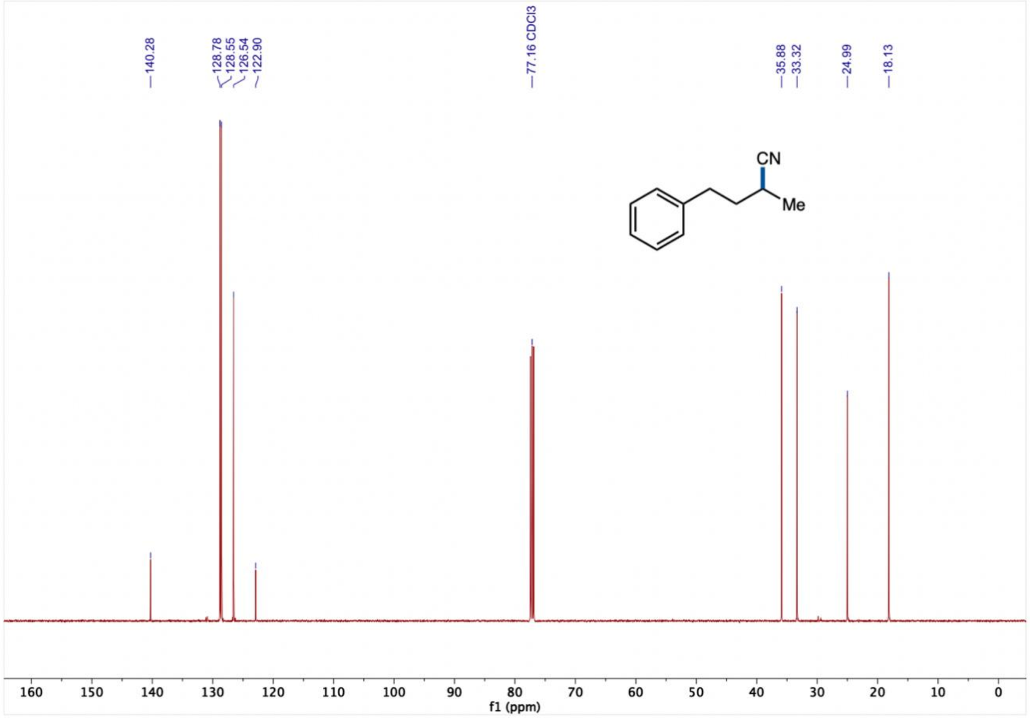

**(±)-*Tert*-butyl-3-cyano-1-oxa-8-azaspiro[4.5]decane-8-carboxylate (15)**

$^1$H NMR (400 MHz, $CDCl_3$)

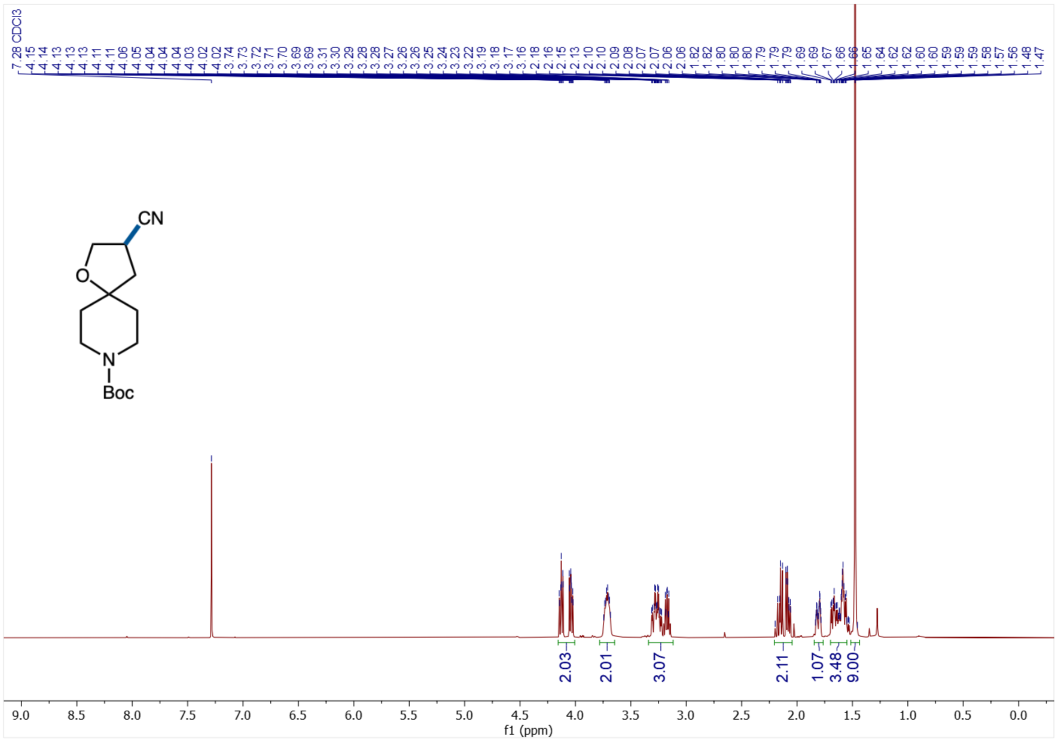


$^{13}$C NMR (126 MHz, $CDCl_3$)

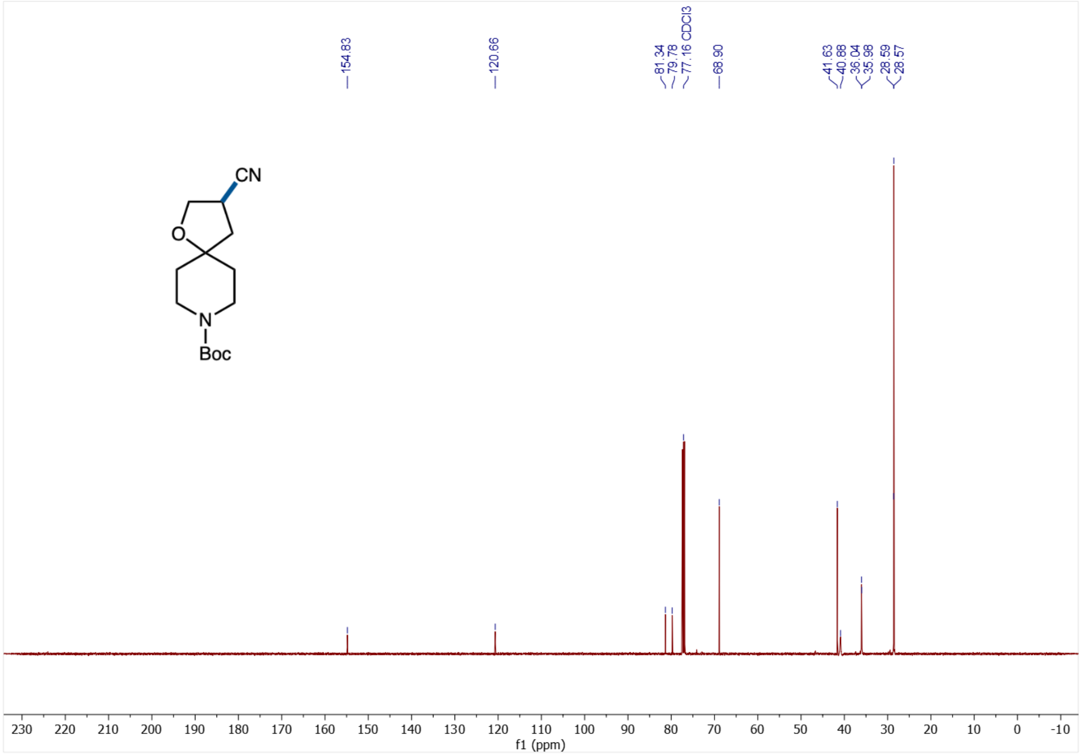

**(±)-*Tert*-butyl 6-cyano-2-azaspiro[3.4]octane-2-carboxylate (16)**

$^{1}H$ NMR (400 MHz, $CDCl_3$)

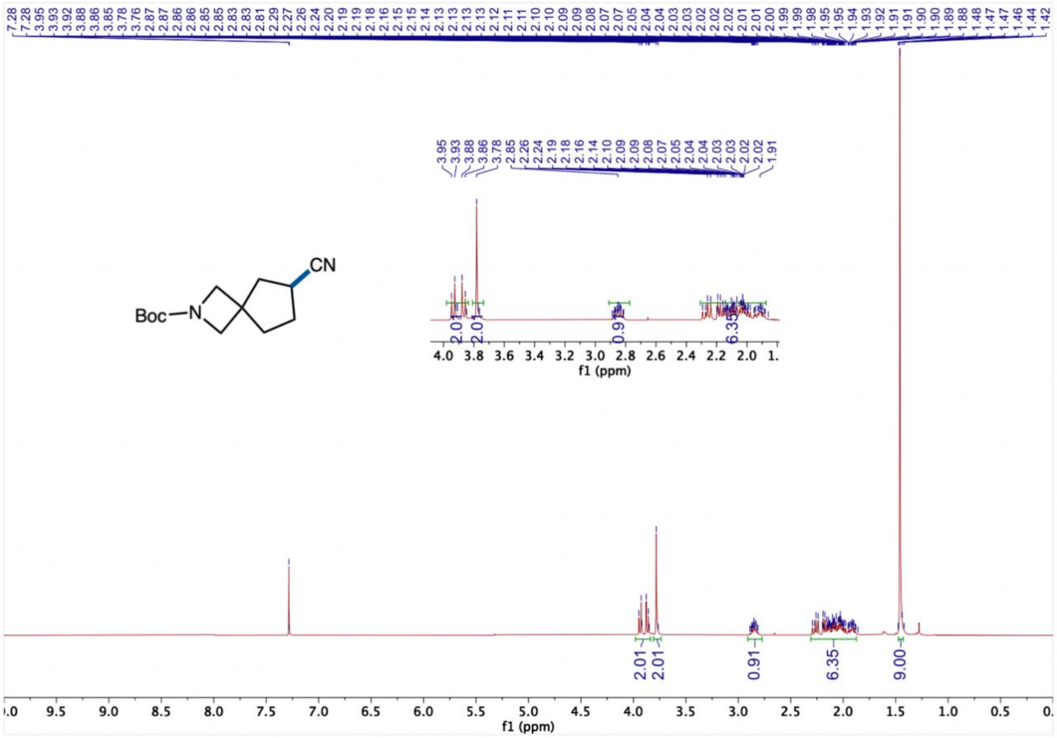


$^{13}C$ NMR (126 MHz, $CDCl_3$)

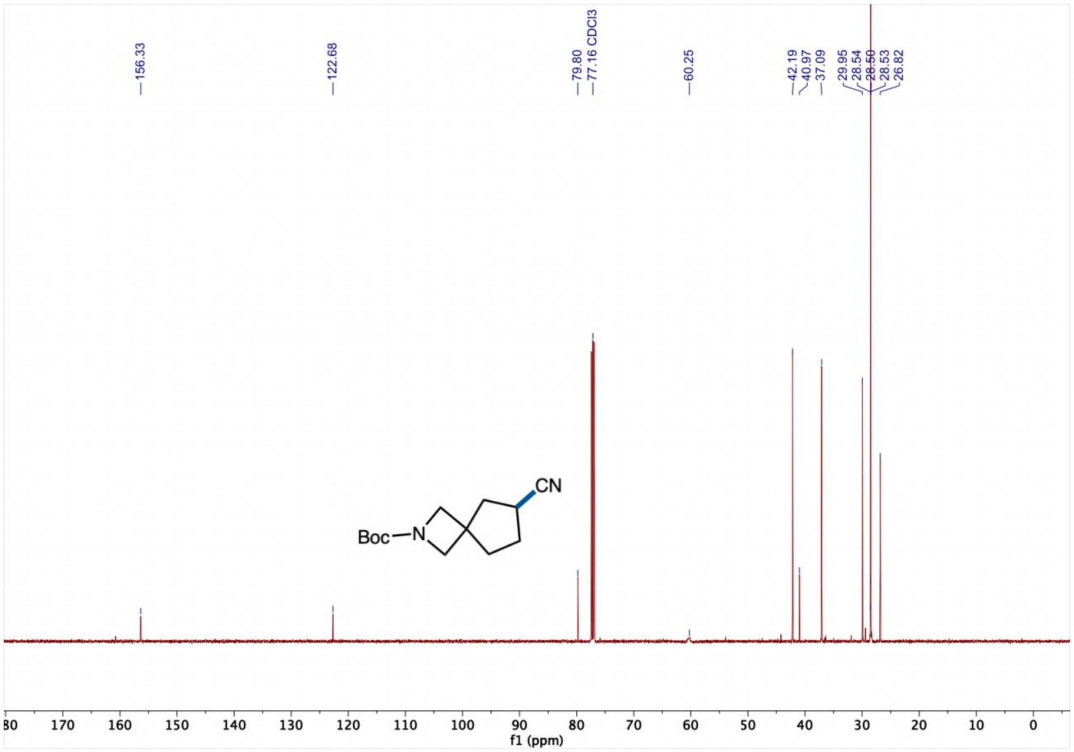

*Tert*-butyl-3-cyano-8-azabicyclo[3.2.1]octane-8-carboxylate (17)

$^{1}$H NMR (400 MHz, $CDCl_3$)

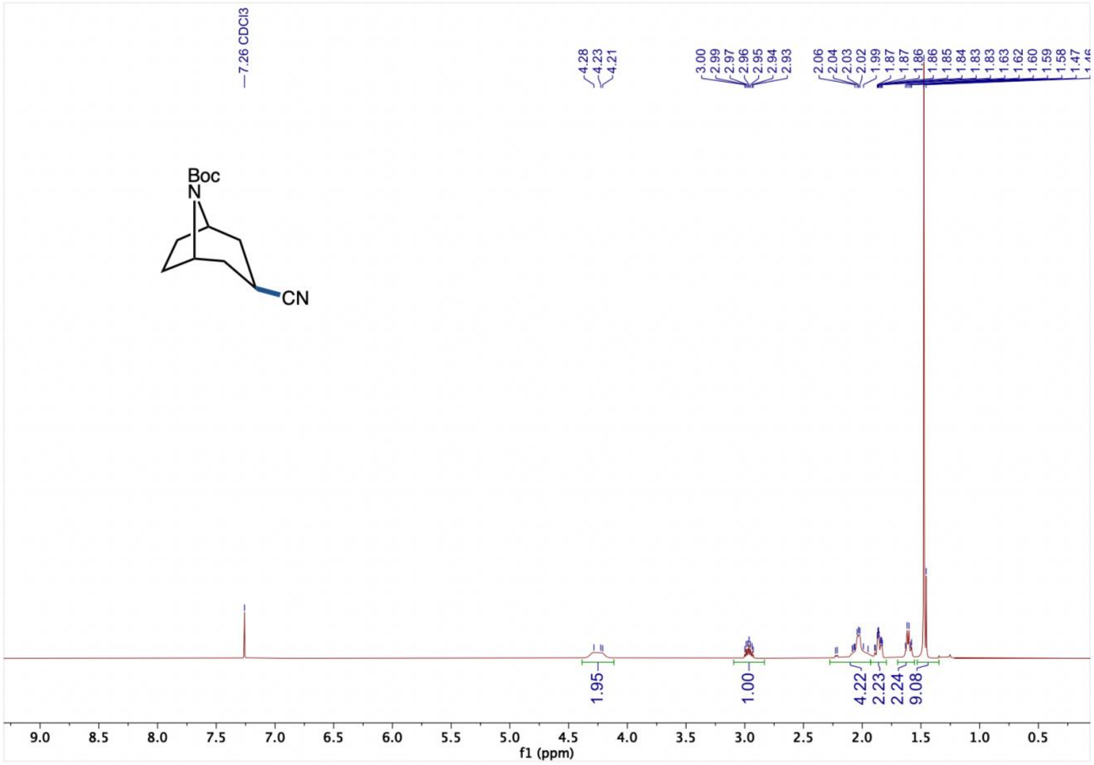


Quantitative $^{13}$C NMR (126 MHz, $CDCl_3$)

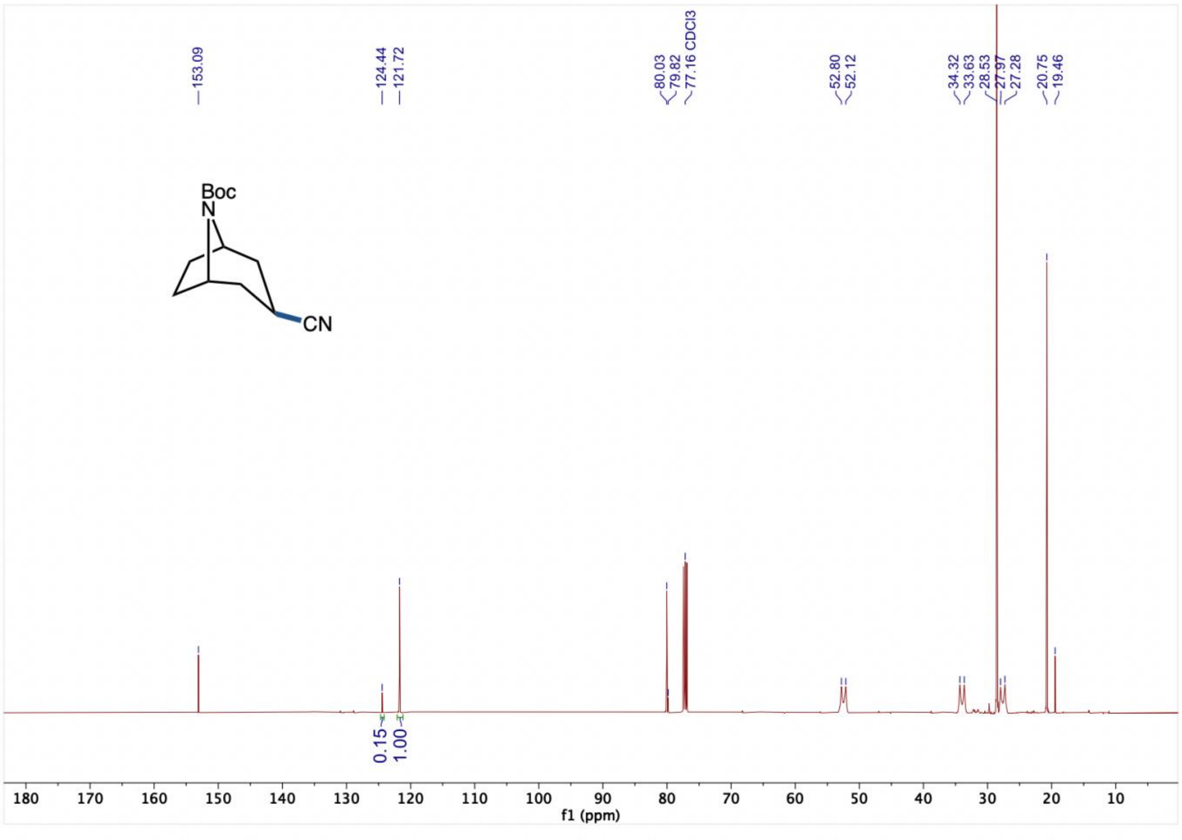

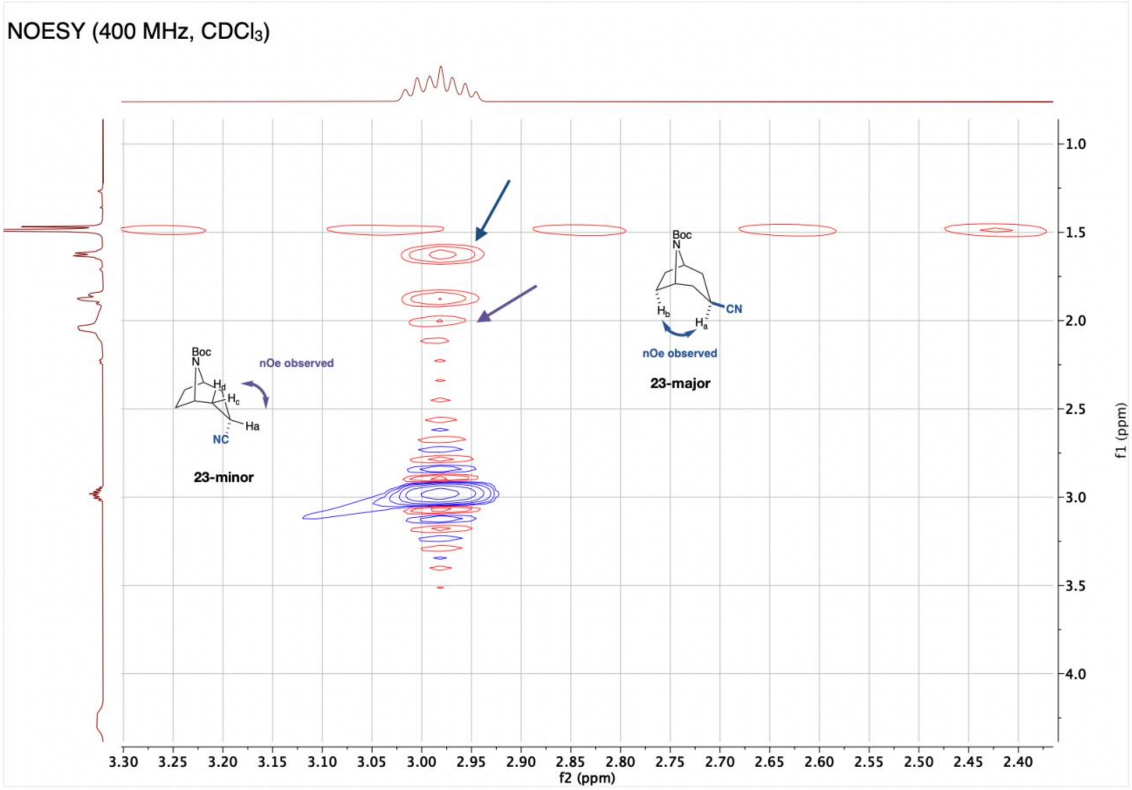
NOESY (400 MHz, CDCl3)
Boc
N
Hd
Hc
NC
Ha
nOe observed
23-minor
Boc
N
Hb
Ha
CN
nOe observed
23-major
f1 (ppm)
f2 (ppm)

***Tert*-butyl-5-cyano-2-(2,5-difluorophenyl)tetrahydro-2*H*-pyran-3-yl)carbamate (18)**

**Major diastereomer**

$^{1}H$ NMR (500 MHz, $CDCl_3$)

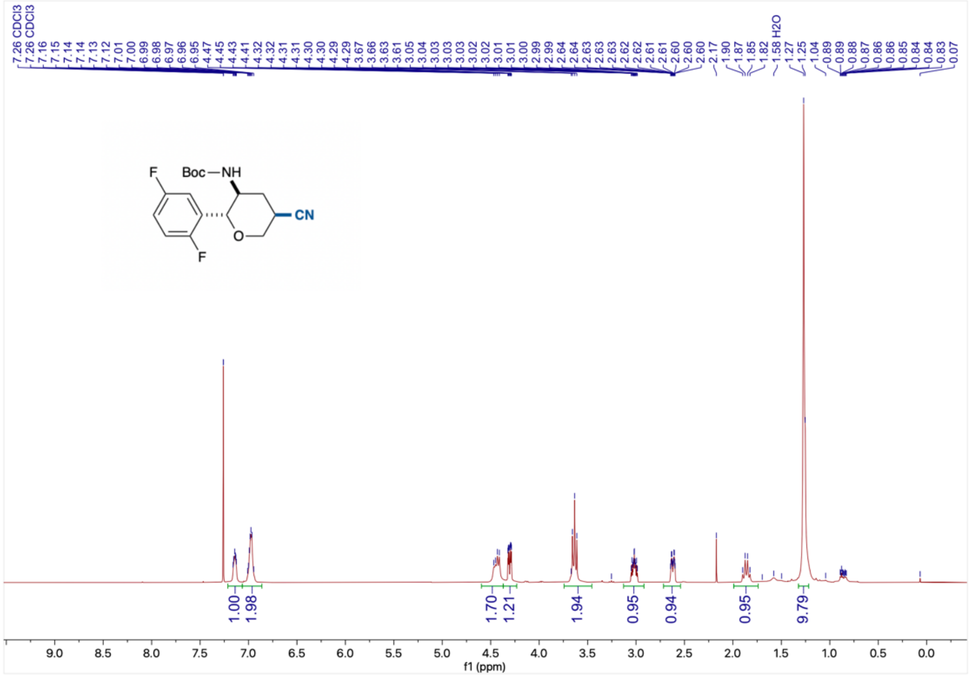

$^{13}C$ NMR (126 MHz, $CDCl_3$)

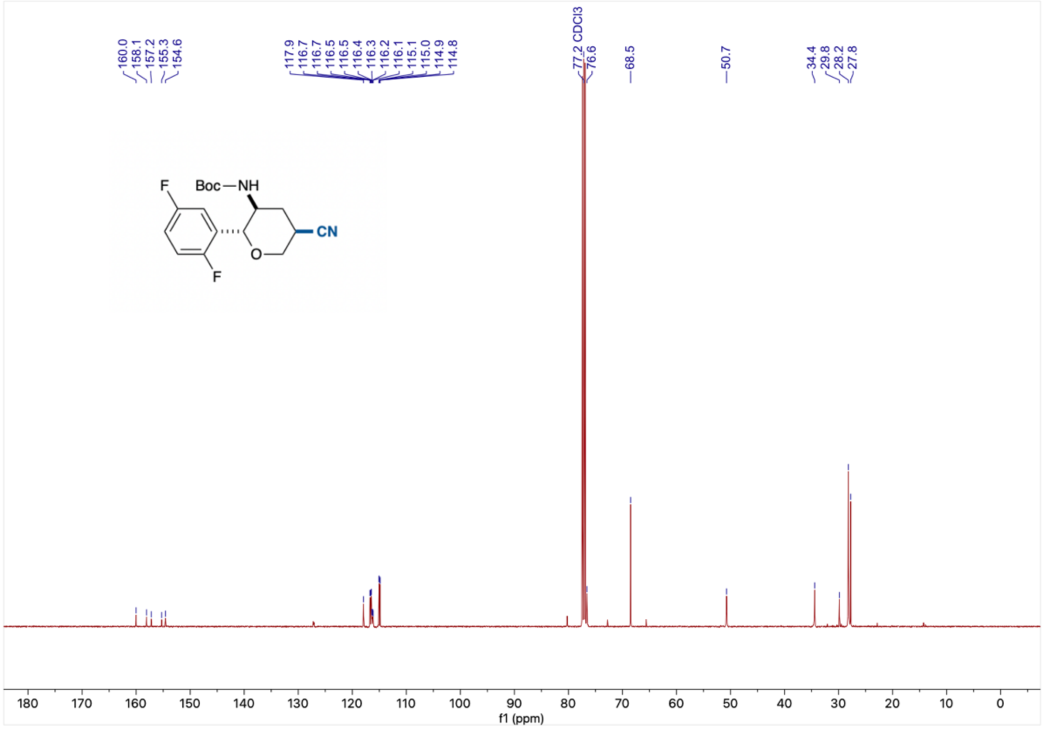

$^{19}F$ NMR (471 MHz, $CDCl_3$)

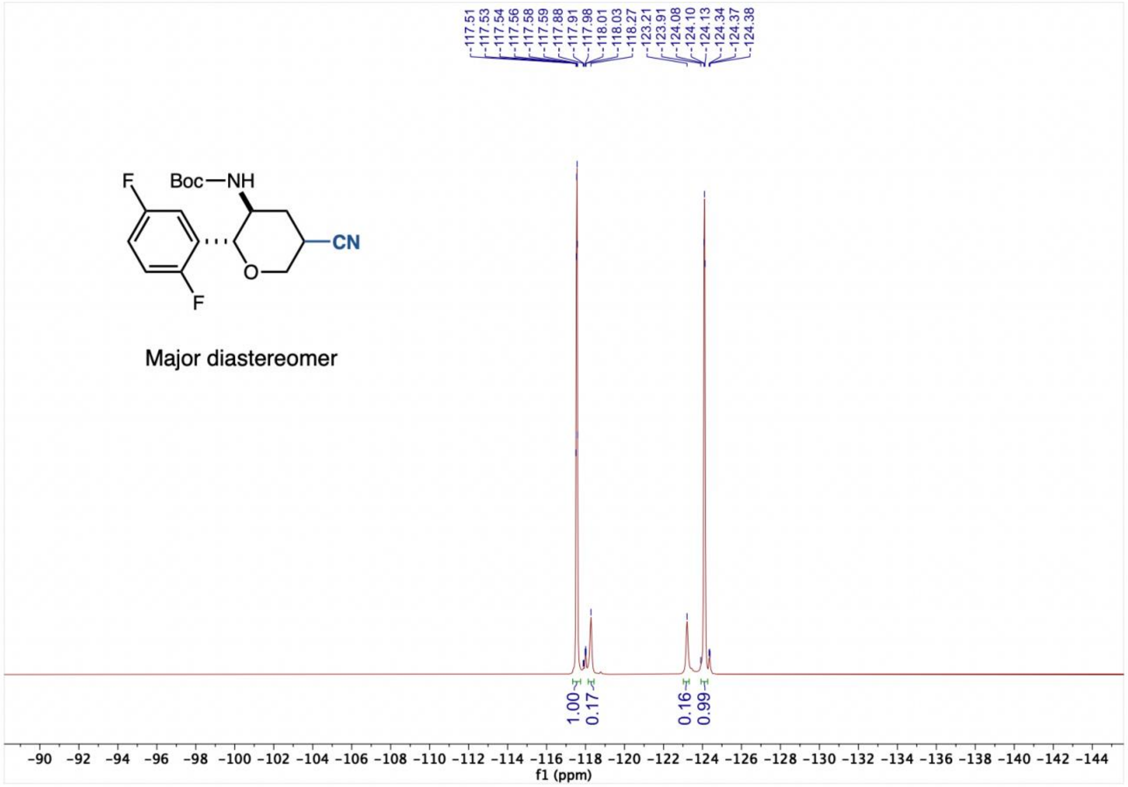


$^1H$-$^1H$ NOESY NMR (500 MHz, $CDCl_3$)

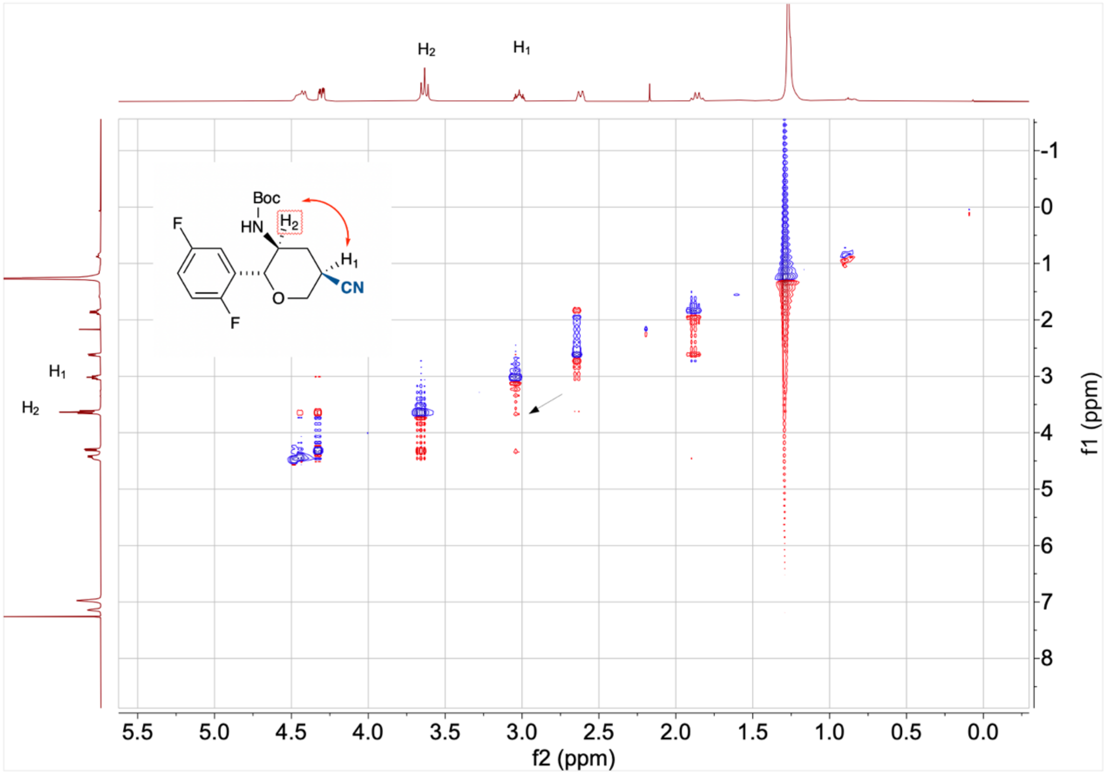

**Minor diastereomer**

$^{1}$H NMR (500 MHz, $CDCl_3$)

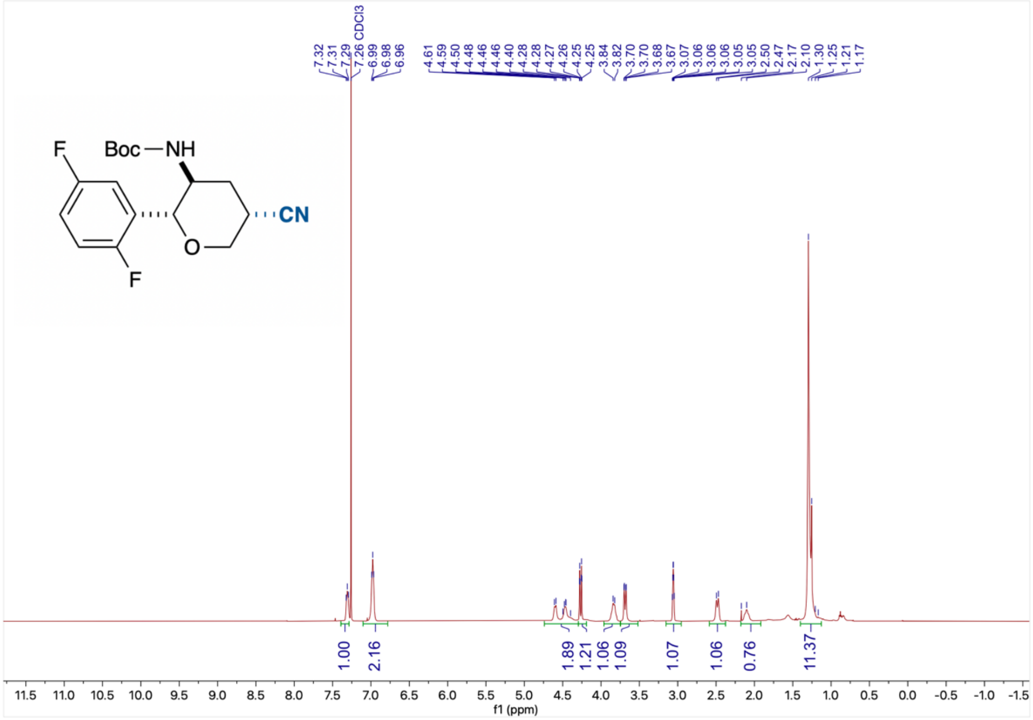


$^{13}$C NMR (126 MHz, $CDCl_3$)

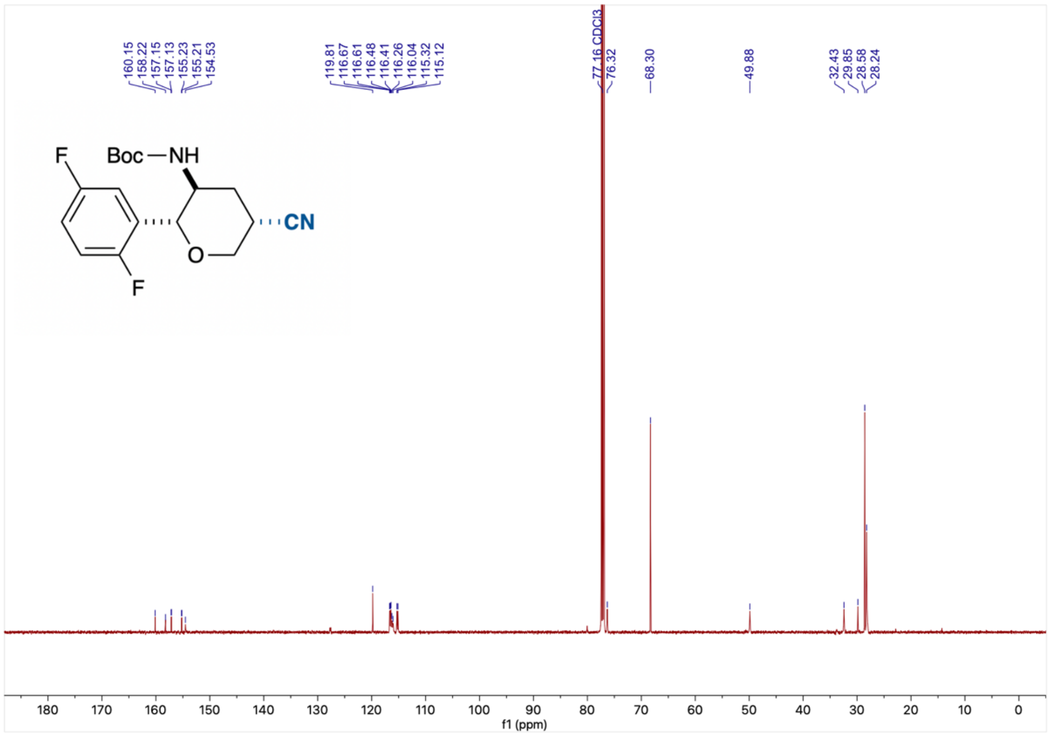

$^{19}$F NMR (471 MHz, CDCl$_3$)

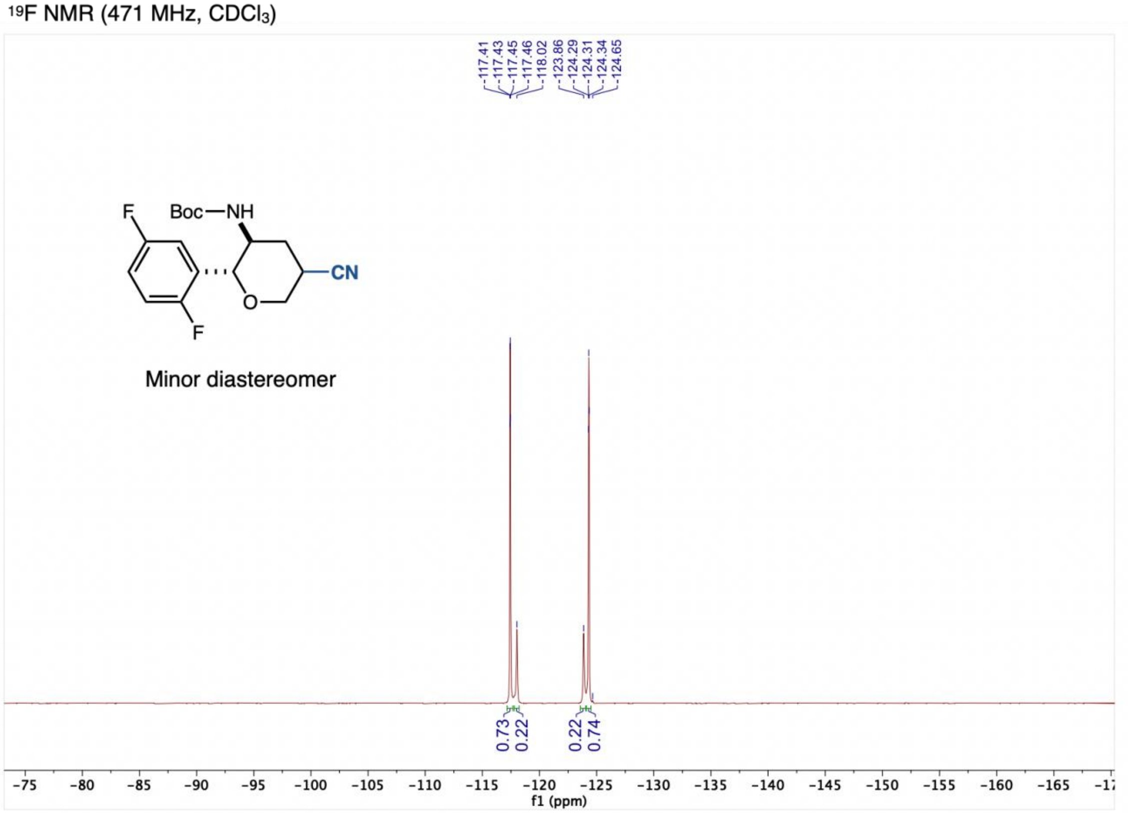


$^1$H-$^1$H NOESY NMR (500 MHz, CDCl$_3$)

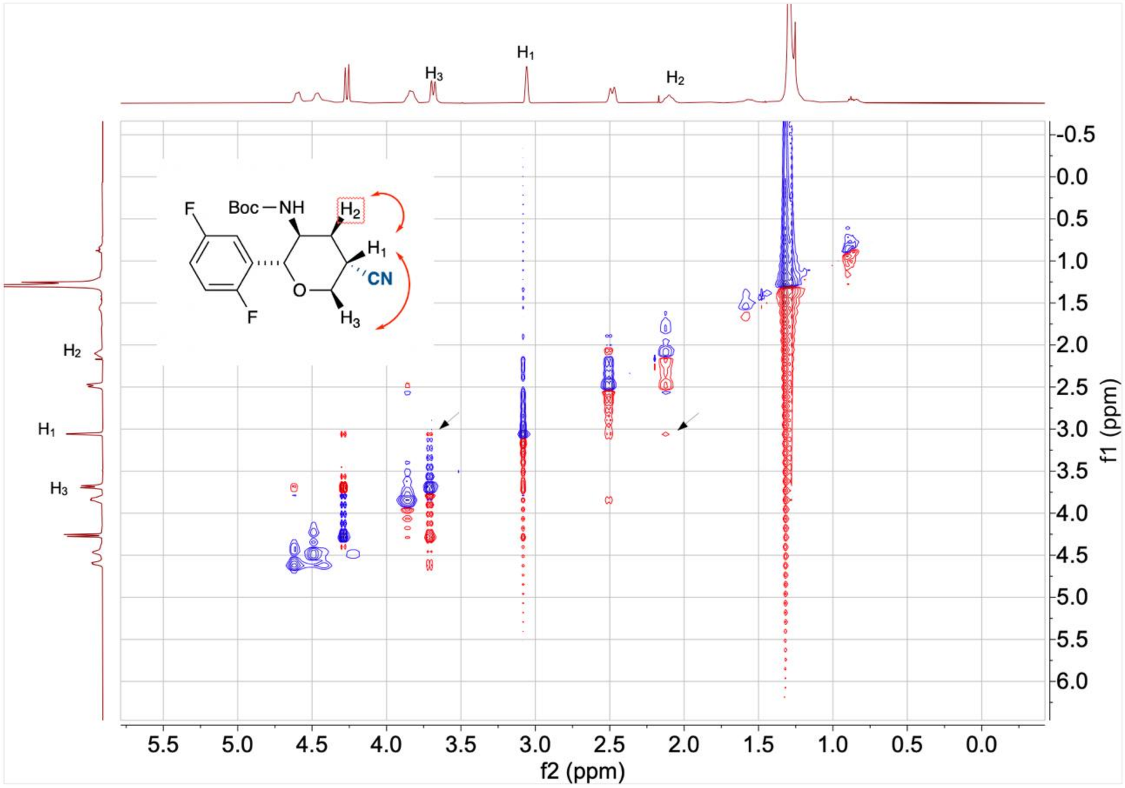

## *N*-(2-cyanoindan-1-yl)-2,2-dimethyl-propanamide (19)

$^{1}$H NMR (400 MHz, $CDCl_3$)

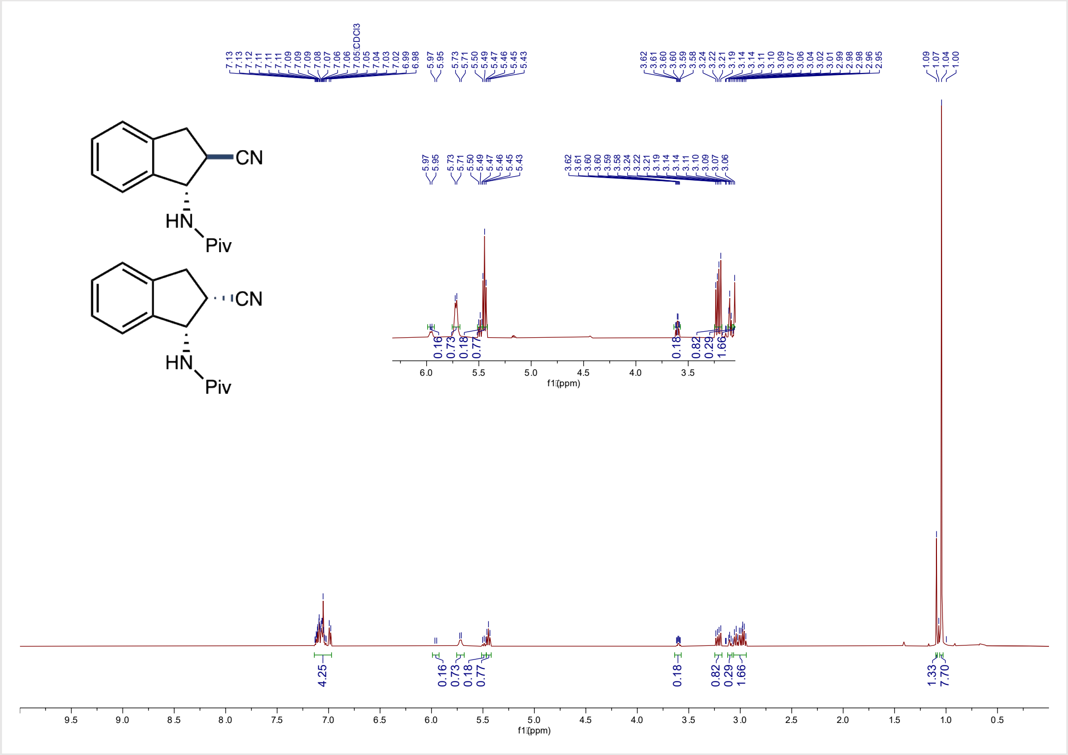

$^{13}$C NMR (126 MHz, $CDCl_3$)

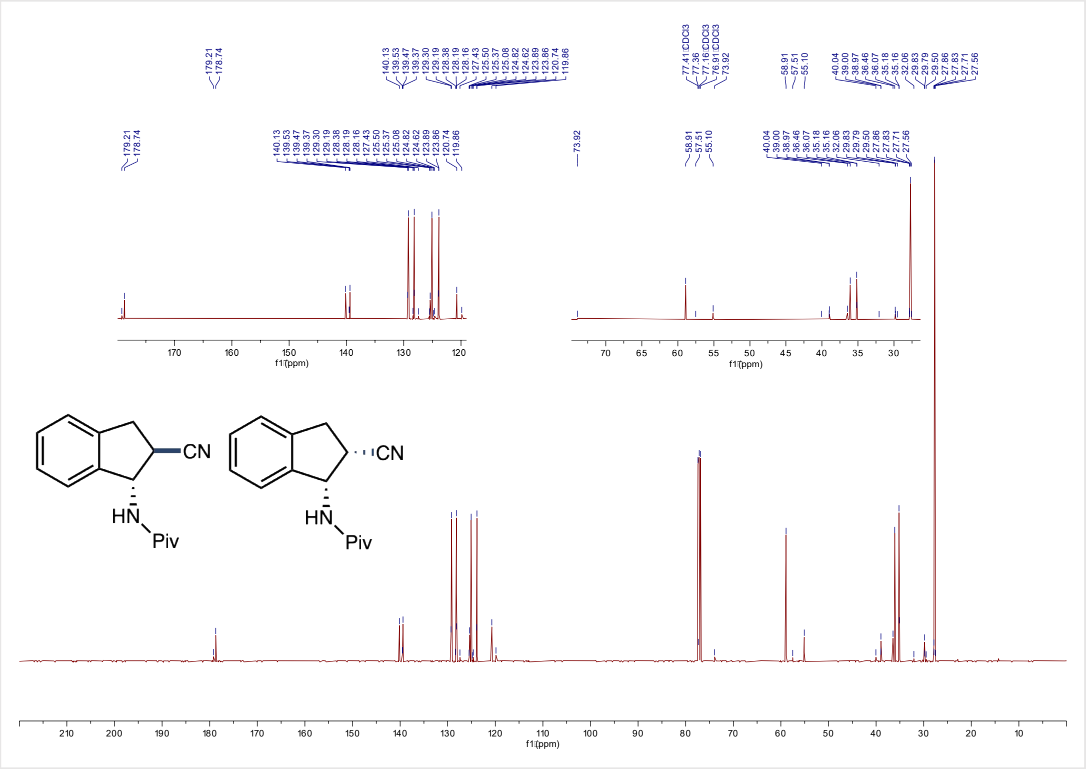

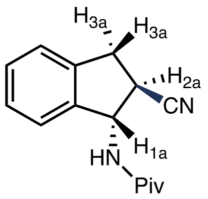


Major diastereomer (a)

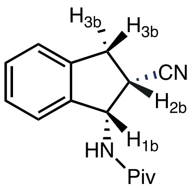


Minor diastereomer (b)

$^{1}$H-$^{1}$H COSY NMR (400 MHz, $CDCl_3$)

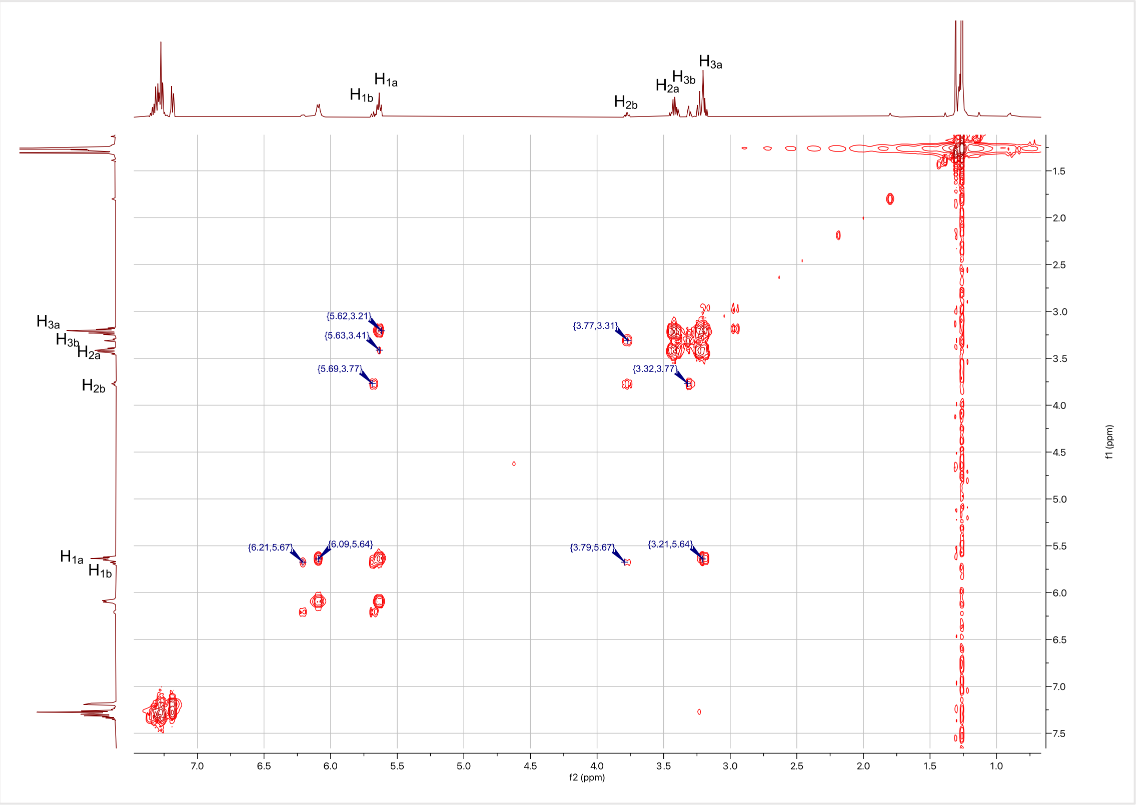

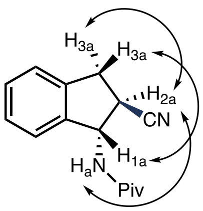


Major diastereomer (a)

$^{1}H$-$^{1}H$ NOESY NMR (400 MHz, $CDCl_3$)

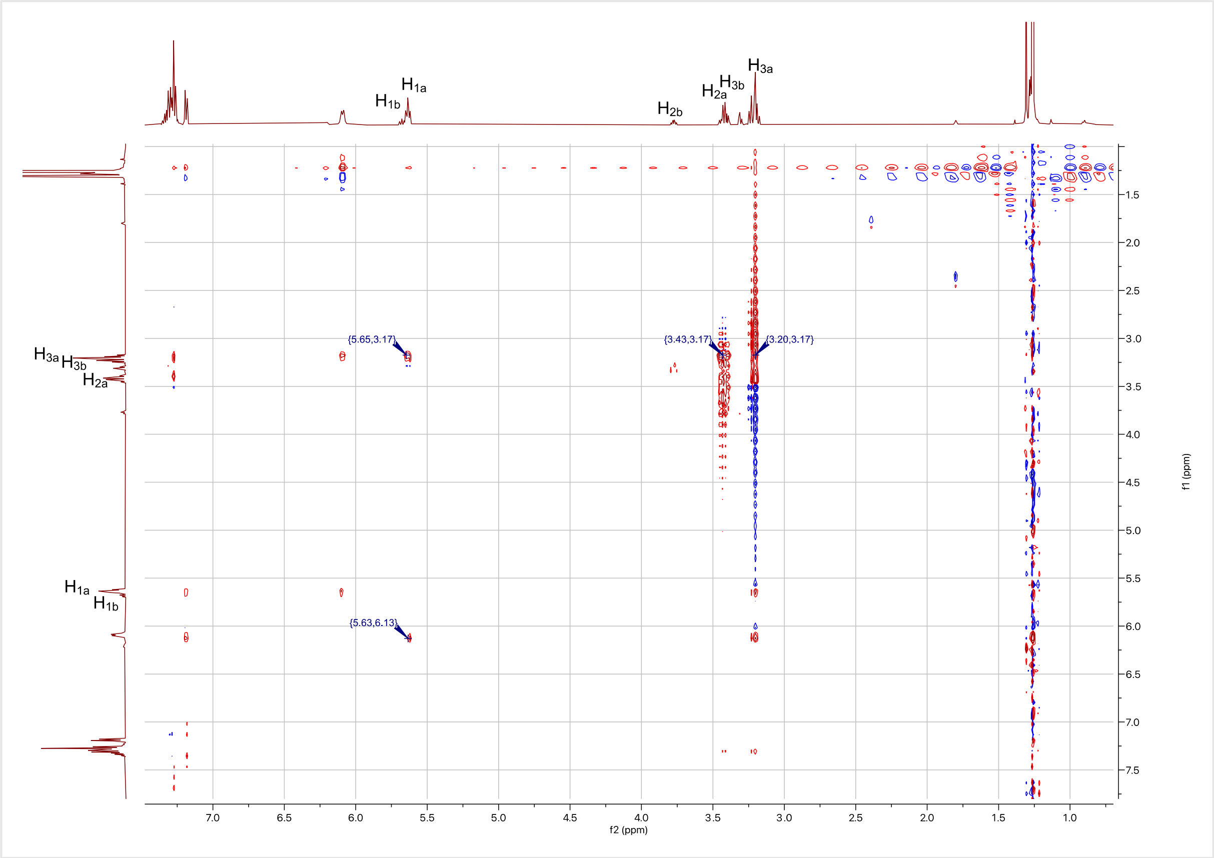

## *Tert*-butyl 3-cyano-3-methylazetidine-1-carboxylate (20)

$^{1}H$ NMR (400 MHz, $CDCl_3$)

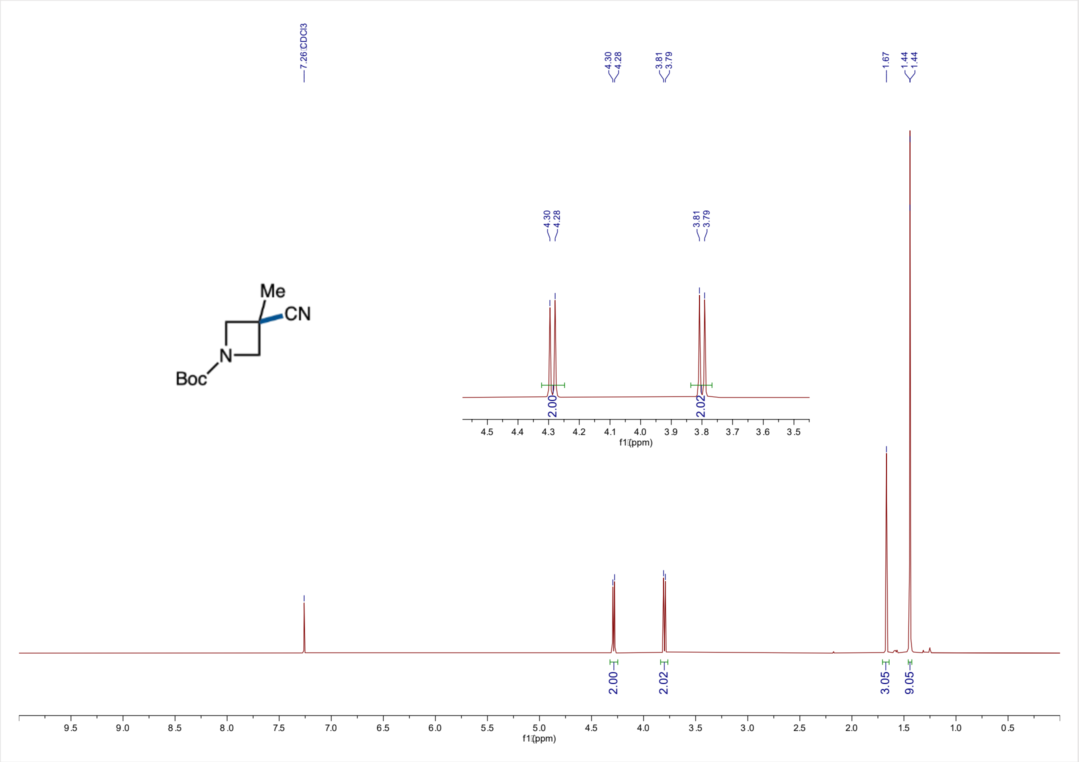


$^{13}C$ NMR (126 MHz, $CDCl_3$)

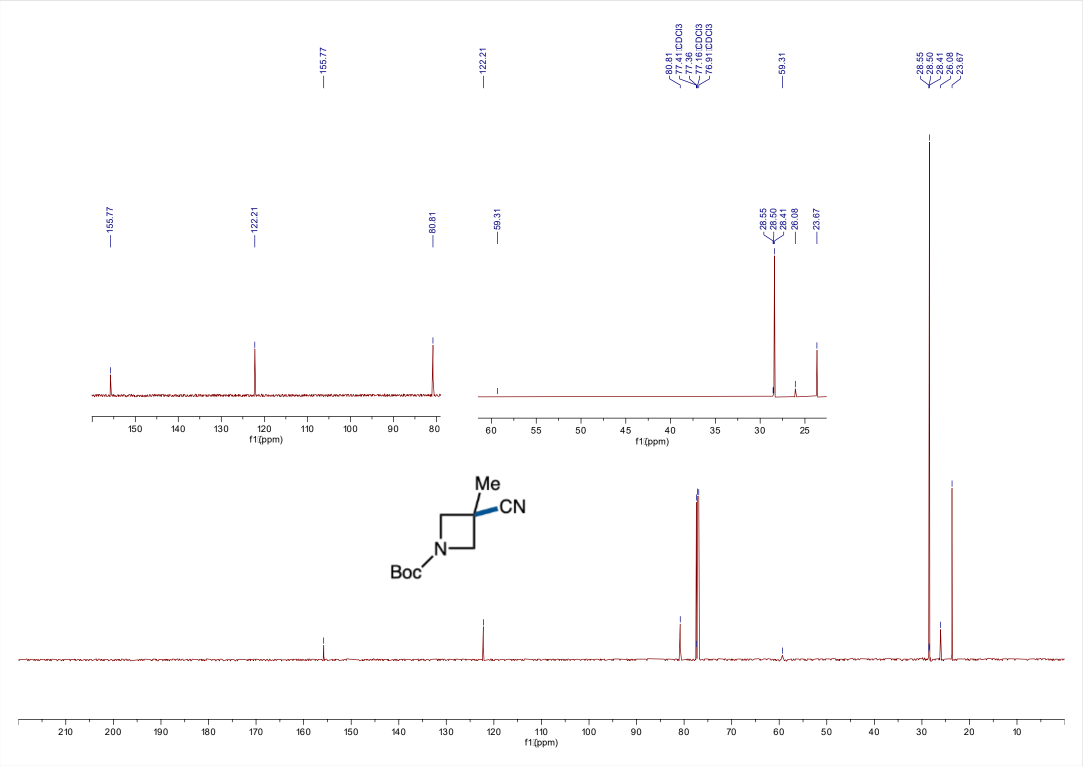

**(±)-Benzyl-3-cyano-3-methylpyrrolidine-1-carboxylate (21)**

$^{1}H$ NMR (400 MHz, $CDCl_3$)

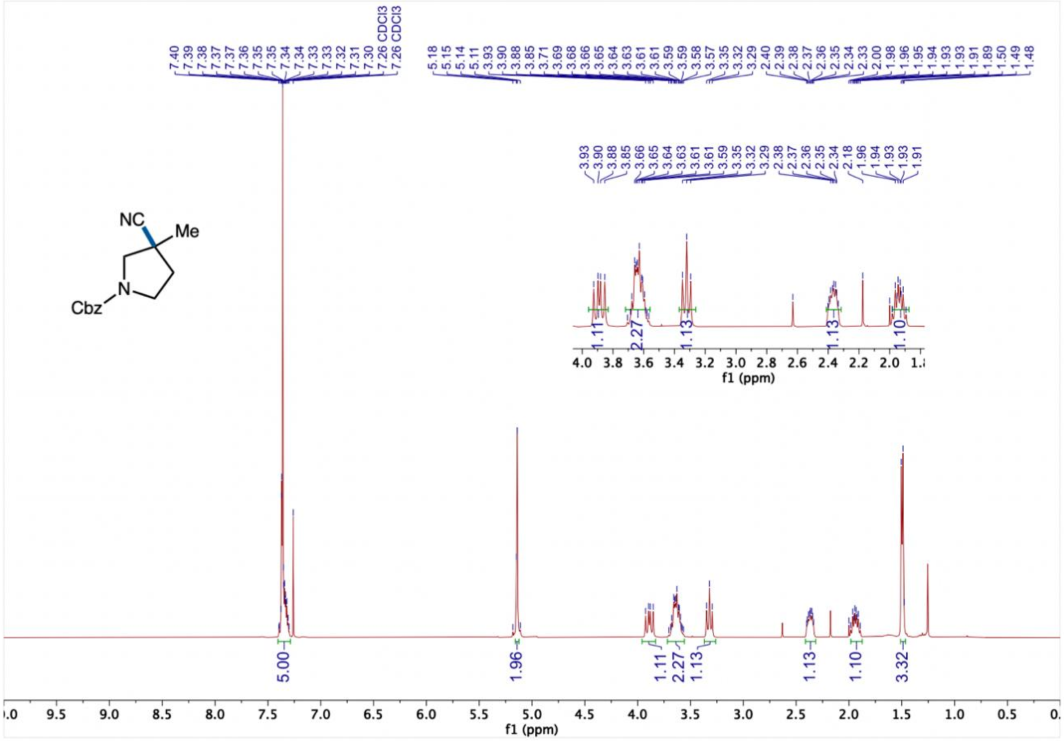


$^{13}C$ NMR (126 MHz, $CDCl_3$)

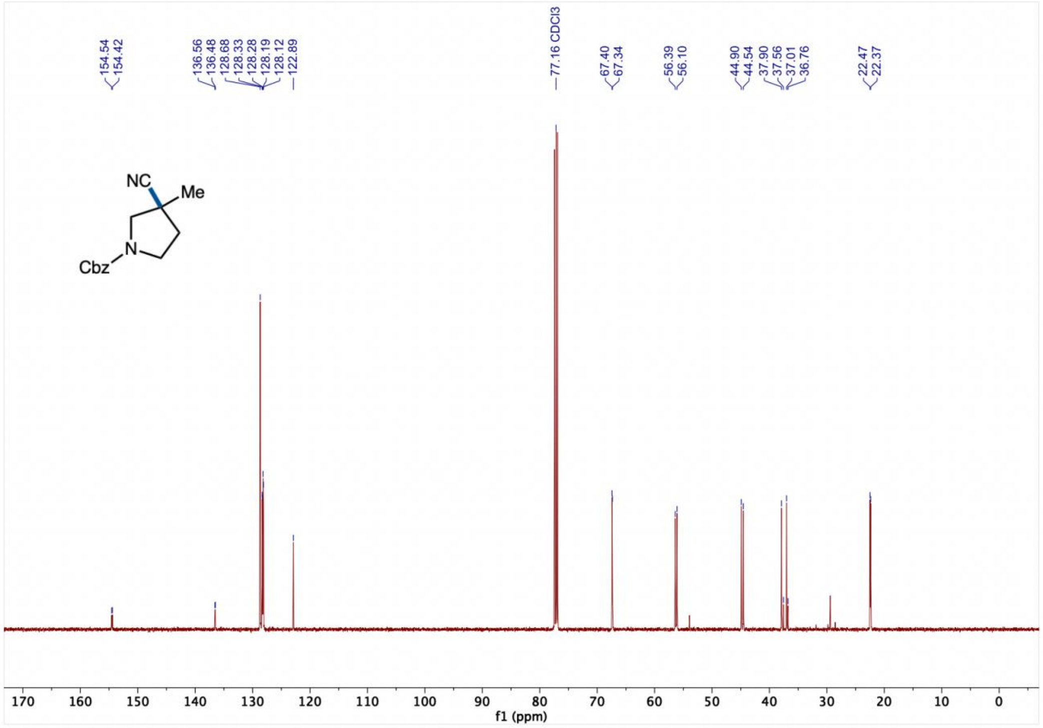

## Benzyl 4-cyano-4-methylpiperidine-1-carboxylate (22)

$^1H$ NMR (400 MHz, $CDCl_3$)

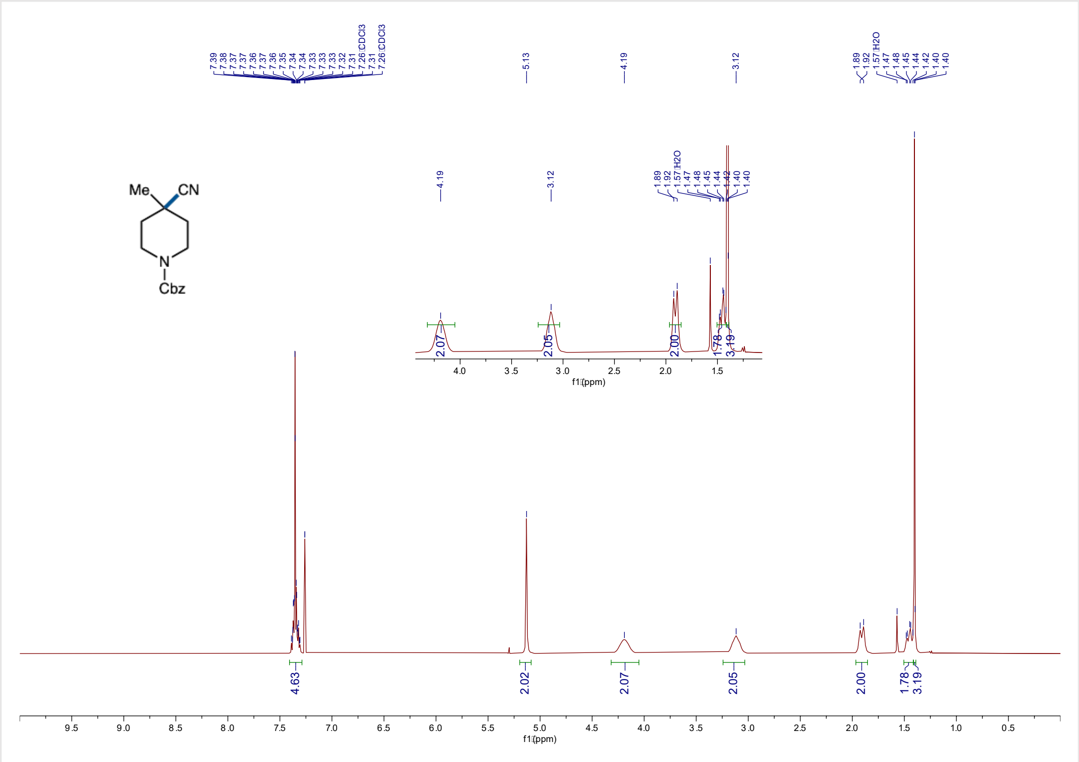

$^{13}C$ NMR (126 MHz, $CDCl_3$)

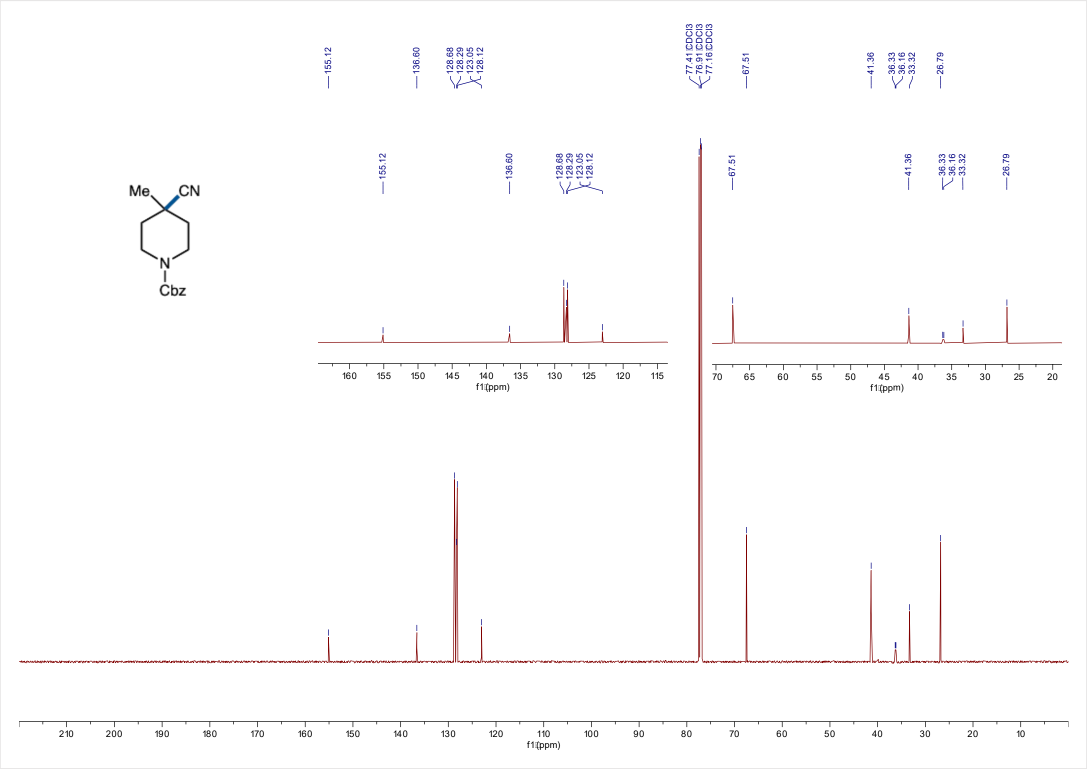

## (±)-Benzyl-3-cyano-3-methylpiperidine-1-carboxylate (23)

$^1H$ NMR (400 MHz, $CDCl_3$)

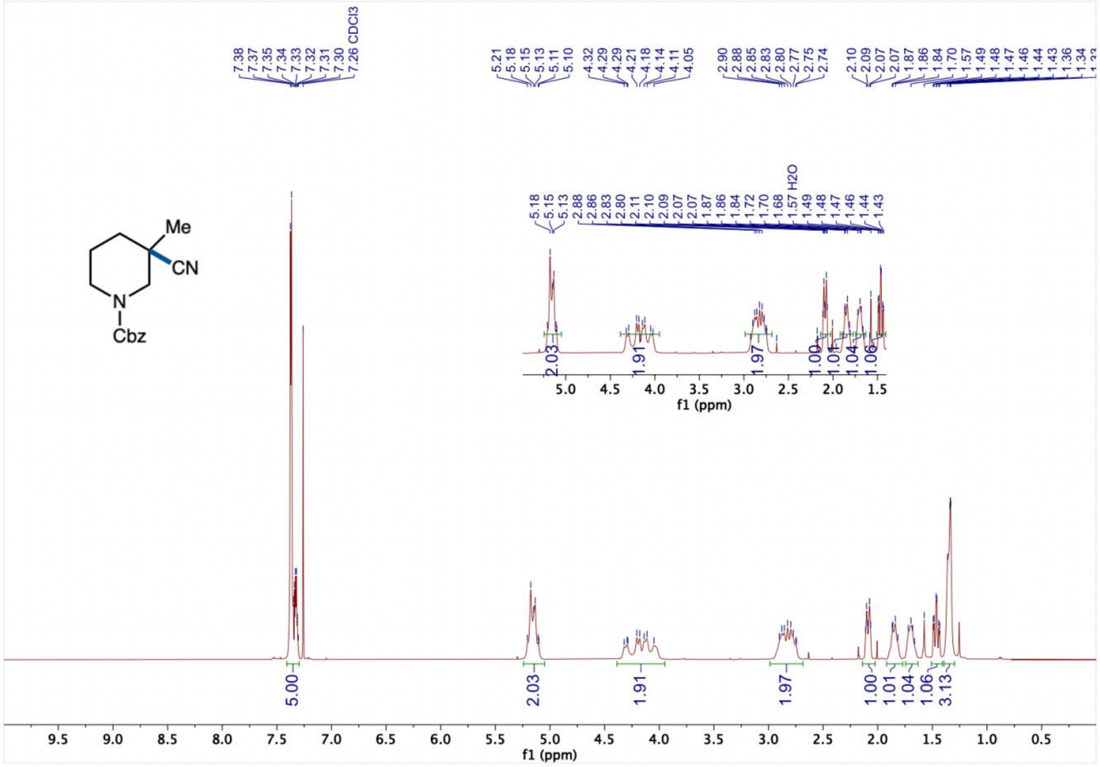

$^{13}C$ NMR (126 MHz, $CDCl_3$)

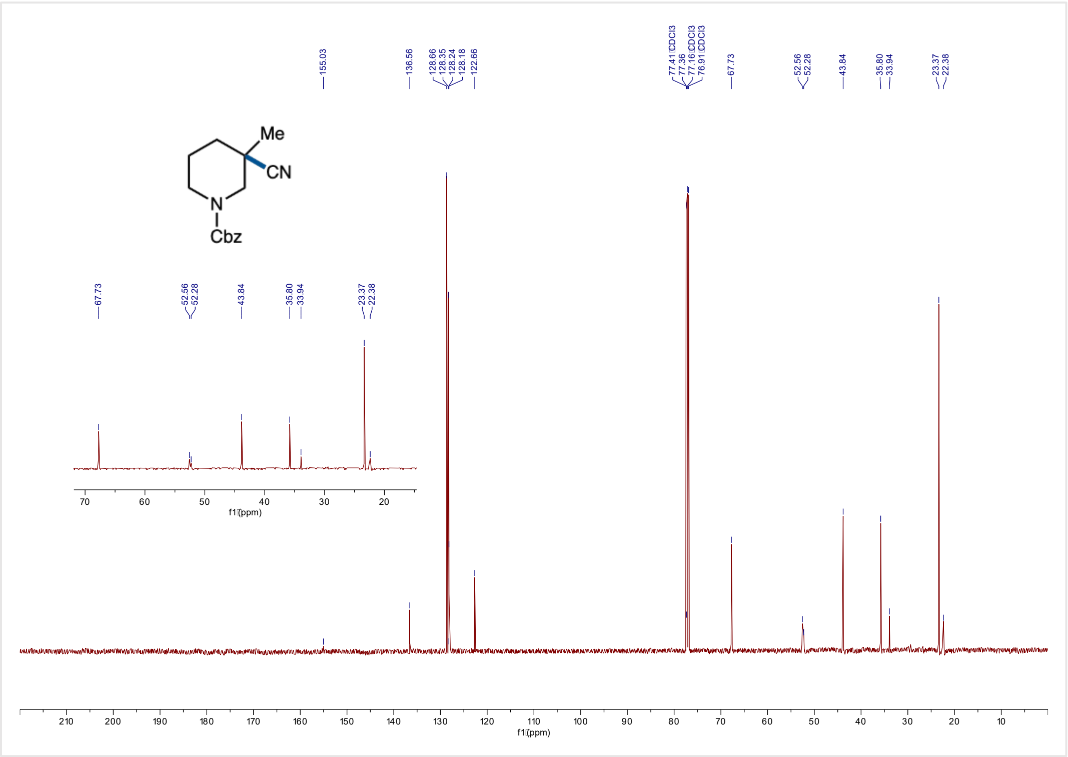

**1-(3,5-di-*tert*-butylbenzoyl)-4-methylpiperidine-4-carbonitrile (24)**

$^{1}$H NMR (400 MHz, $CDCl_3$)

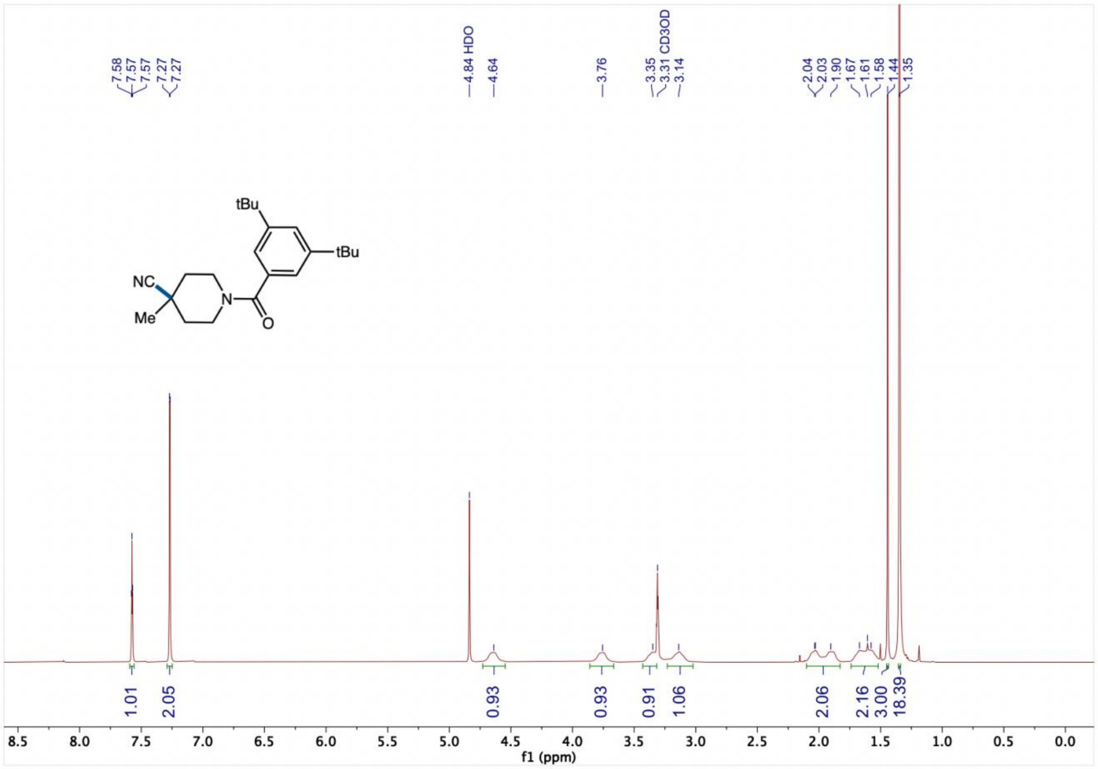


$^{13}$C NMR (126 MHz, $CDCl_3$)

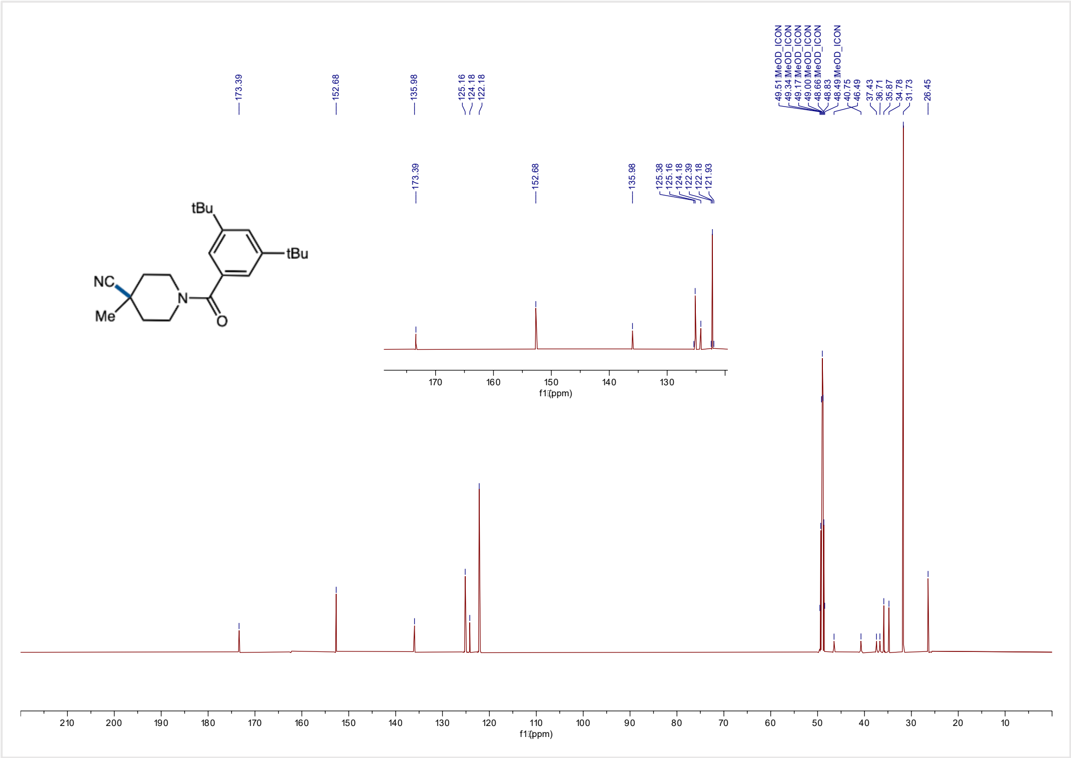

## (±)-*Tert*-butyl-4-cyano-4-methylazepane-1-carboxylate (25)

$^1$H NMR (400 MHz, $CDCl_3$)

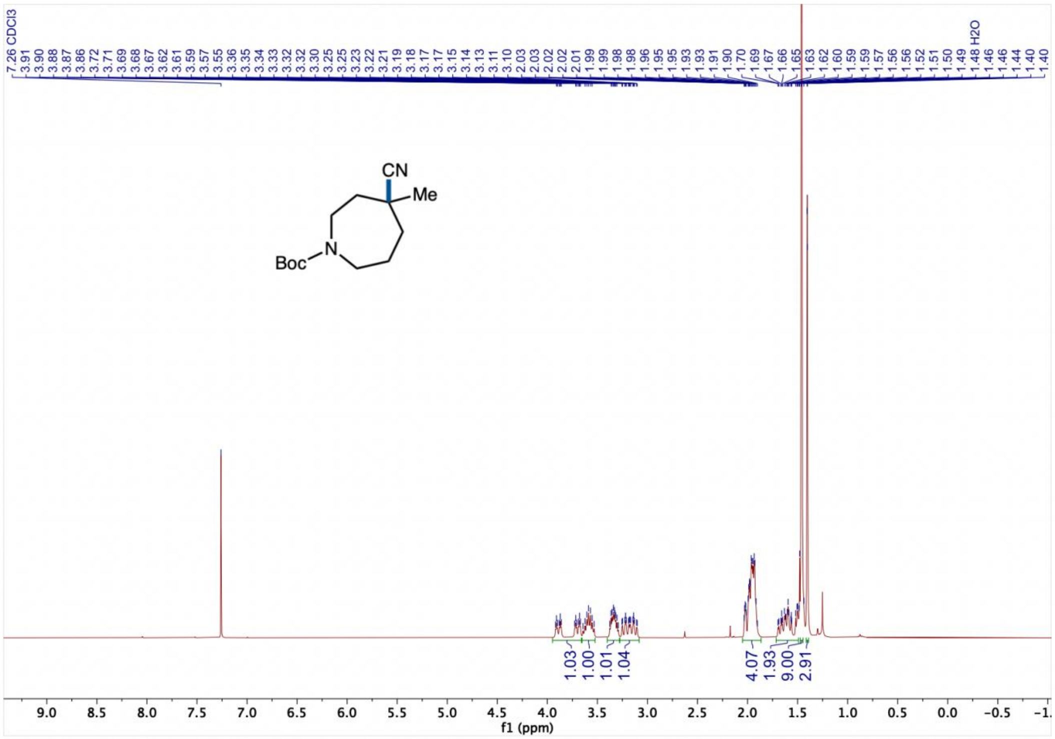


$^{13}$C NMR (126 MHz, $CDCl_3$)

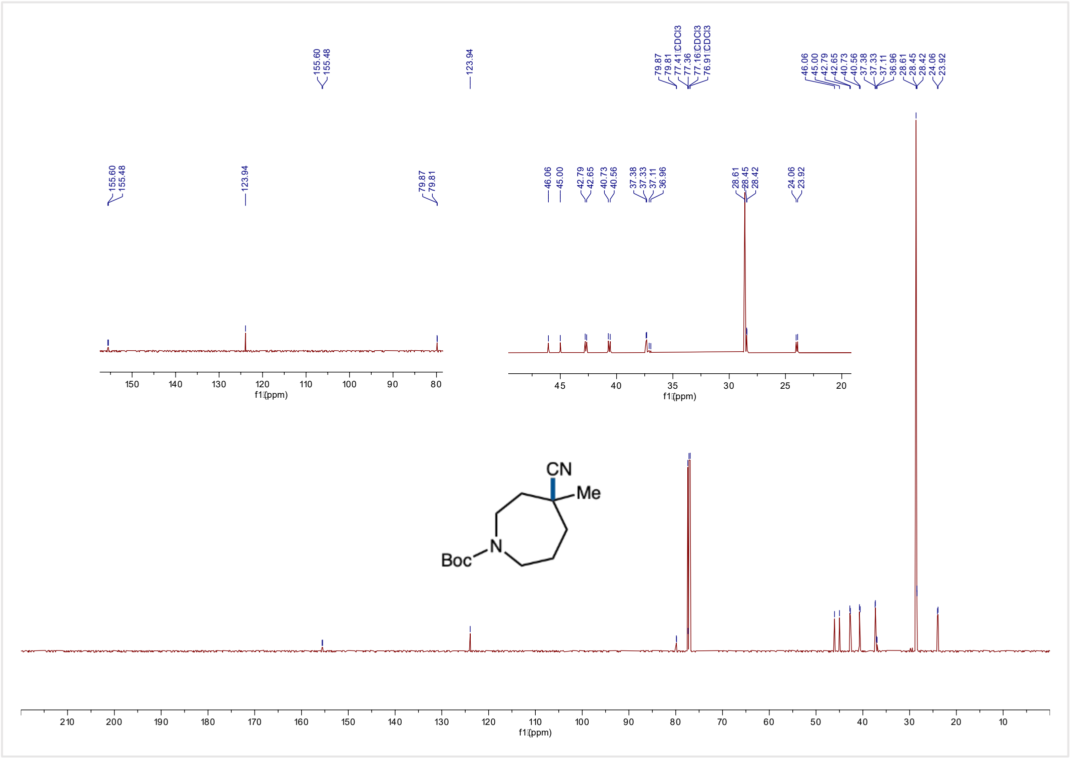

## 2,2-dimethyl-3-phenylpropanenitrile (26)

$^{1}$H NMR (400 MHz, $CDCl_3$)

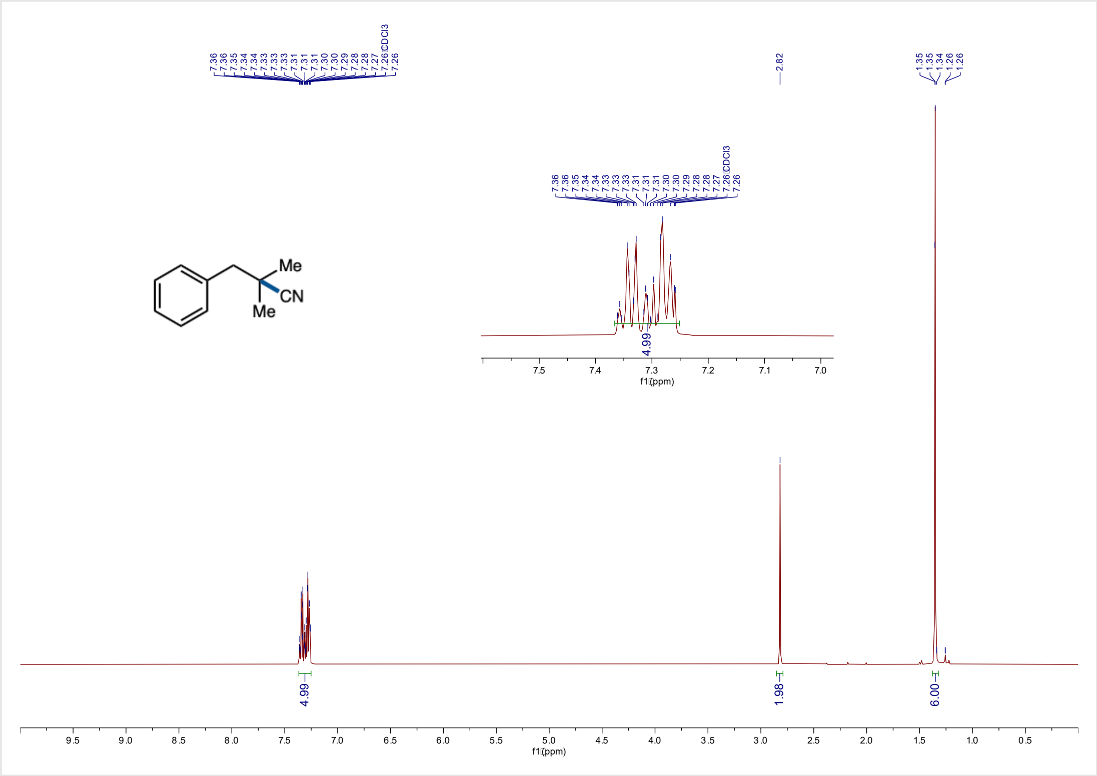

$^{13}$C NMR (126 MHz, $CDCl_3$)

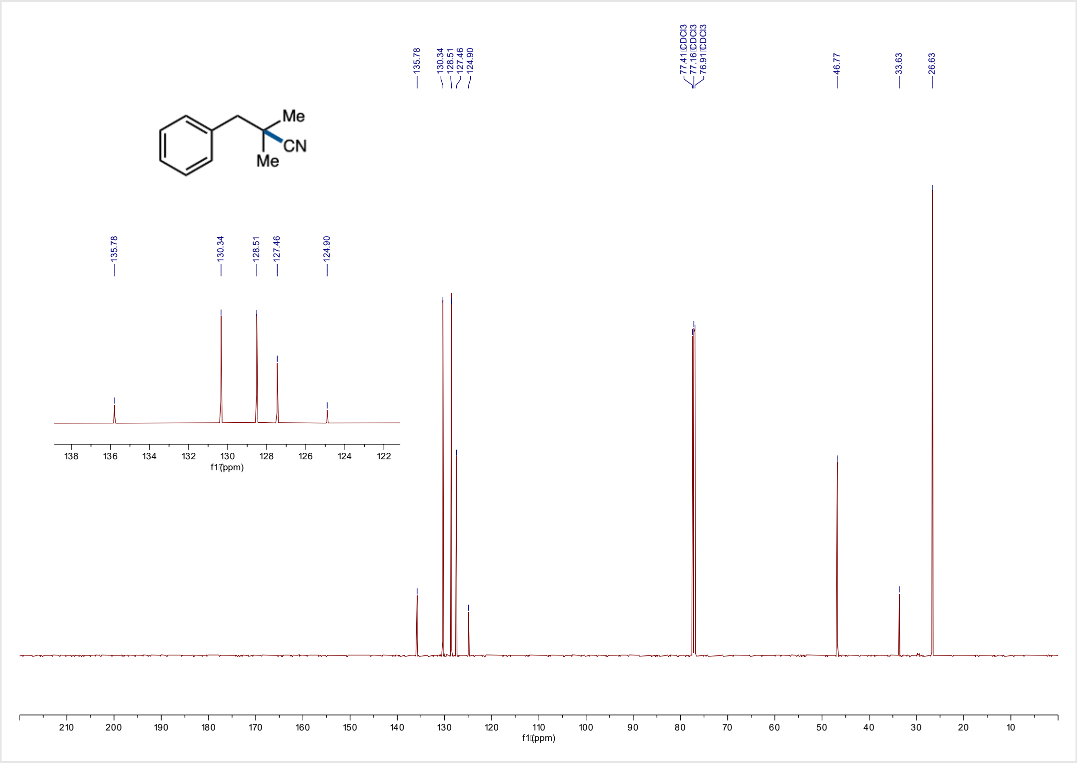

**Benzyl 4-(2-cyanopropan-2-yl)piperidine-1-carboxylate (27)**

$^{1}$H NMR (400 MHz, $CDCl_3$)

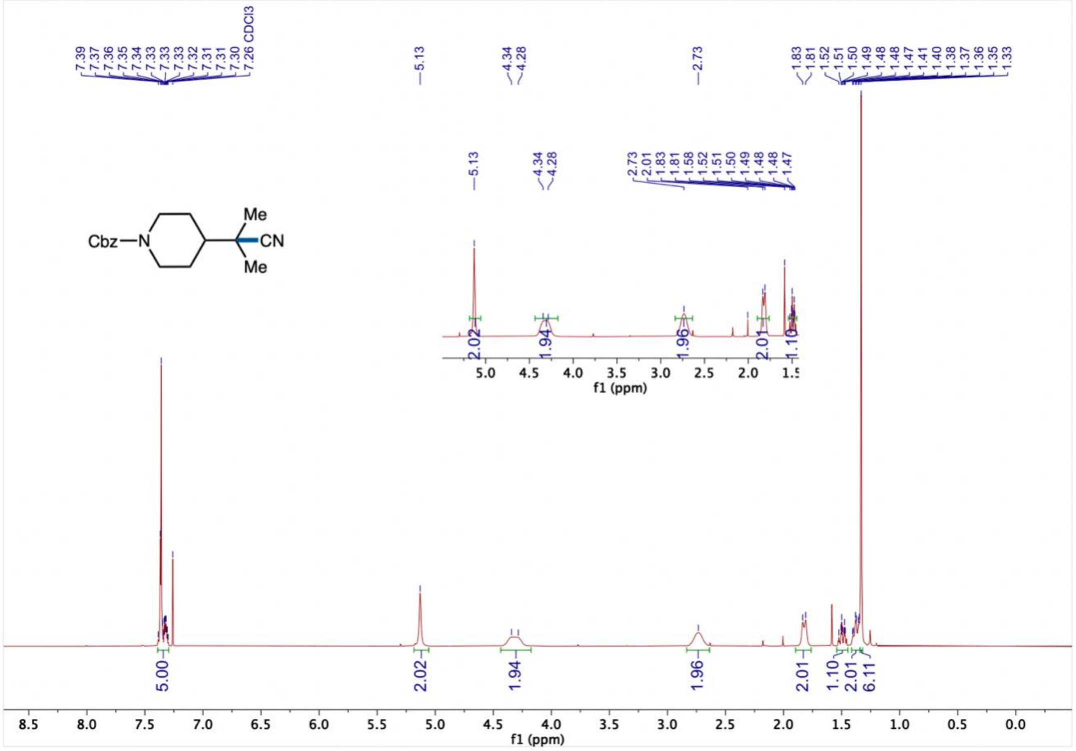

$^{13}$C NMR (126 MHz, $CDCl_3$)

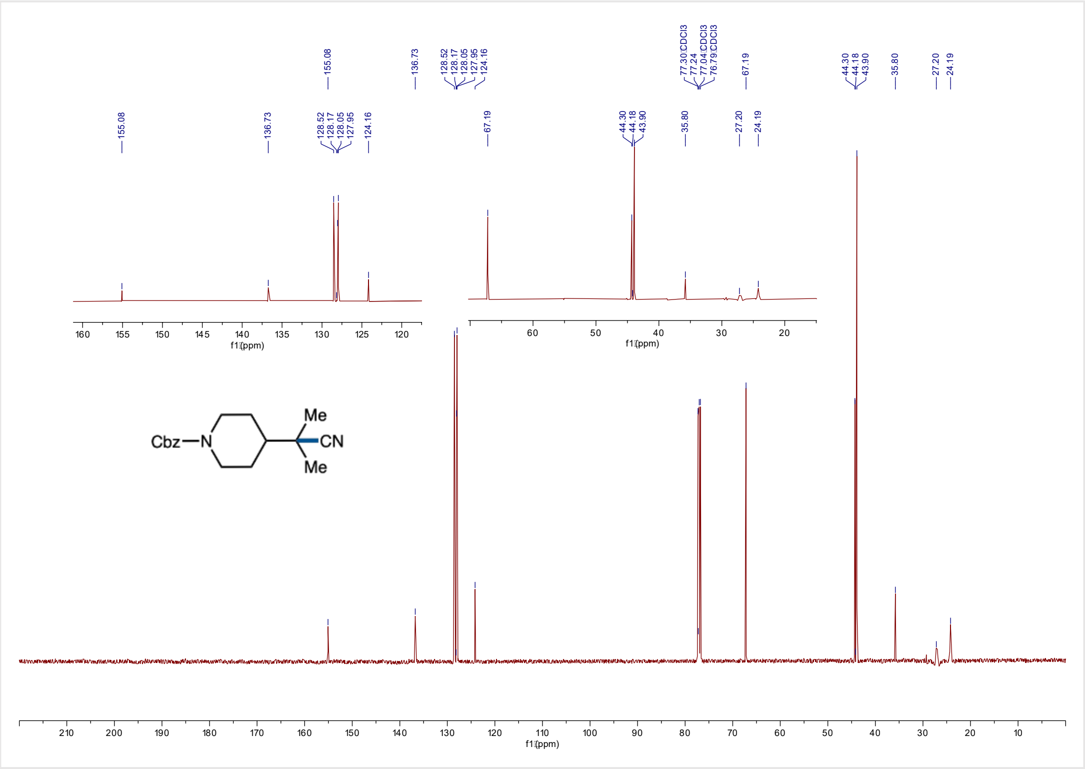

***Tert*-butyl (2-cyano-2-methylpropyl)carbamate (28)**

$^{1}H$ NMR (400 MHz, $CDCl_3$)

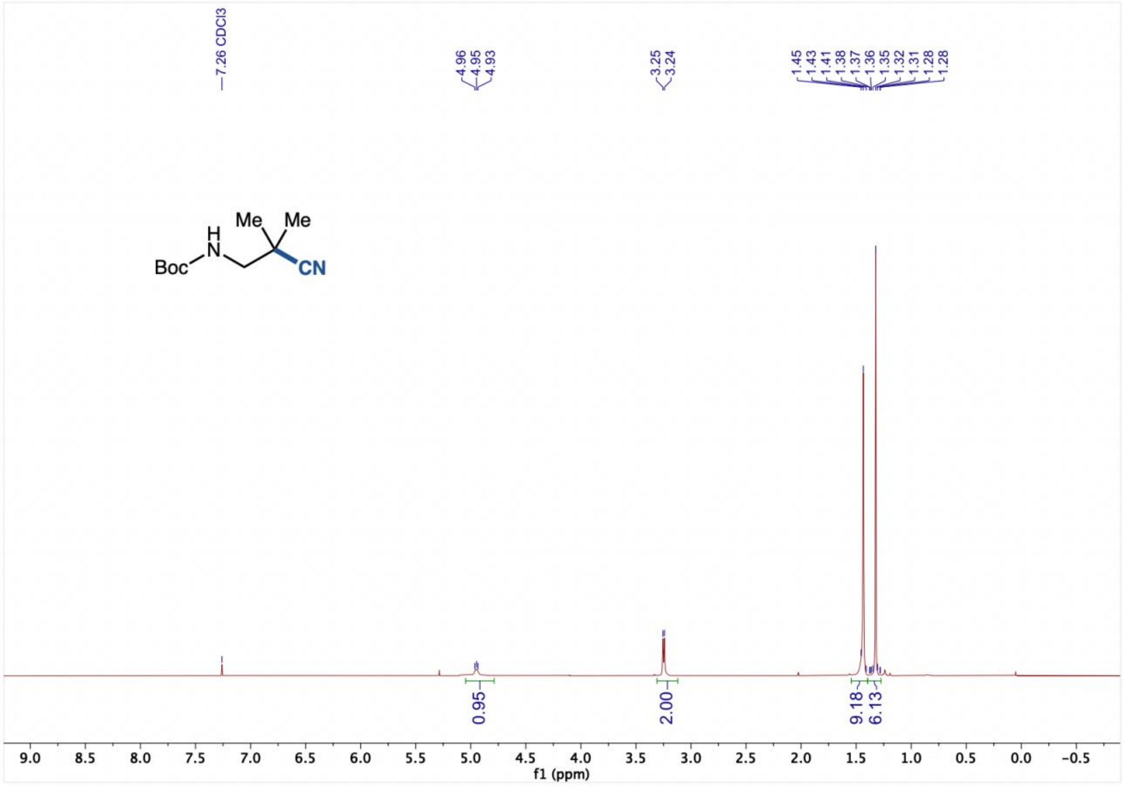


$^{13}C$ NMR (126 MHz, $CDCl_3$)

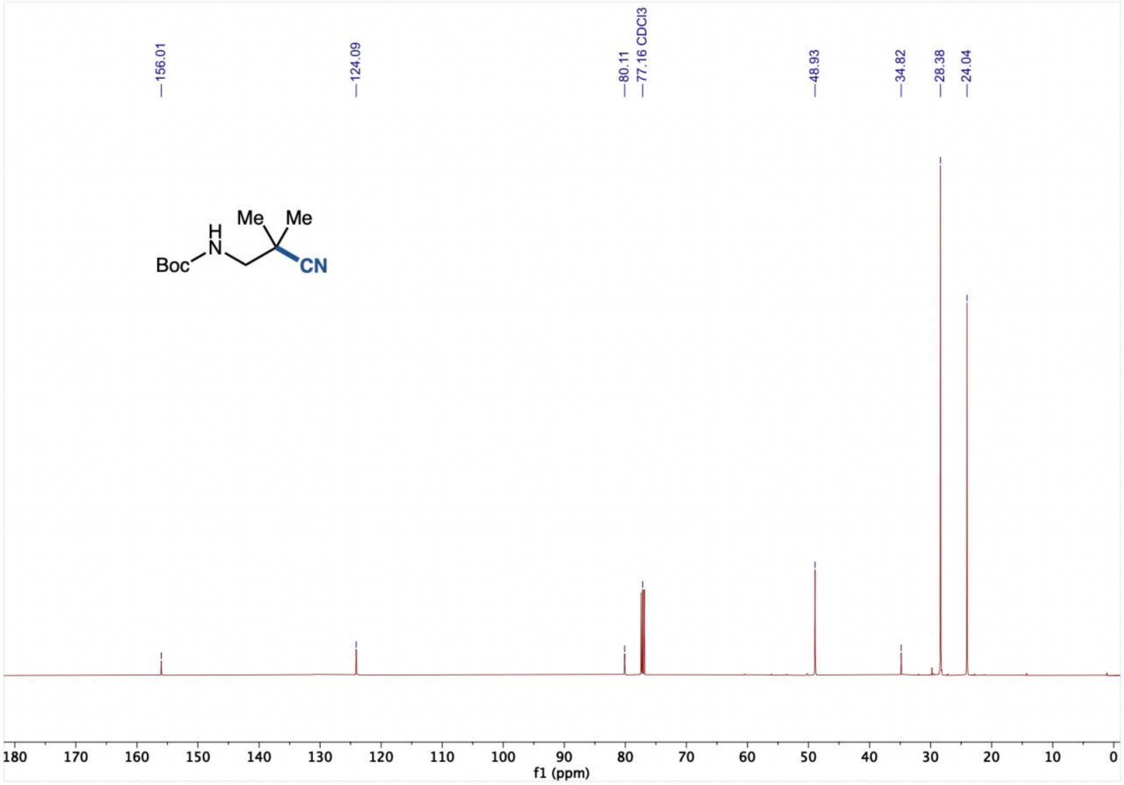

**Benzyl ((1*s*,3*r*,5*R*,7*S*)-3-cyanoadamantan-1-yl)carbamate (29)**

$^{1}H$ NMR (400 MHz, $CDCl_3$)

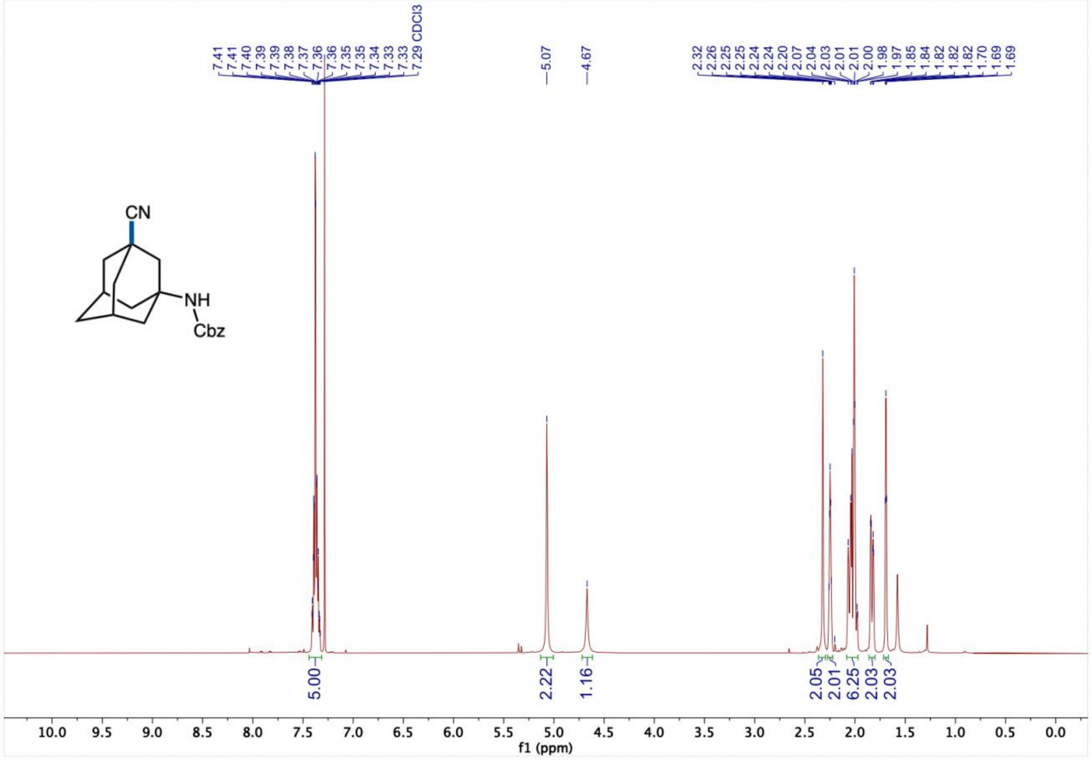


$^{13}C$ NMR (126 MHz, $CDCl_3$)

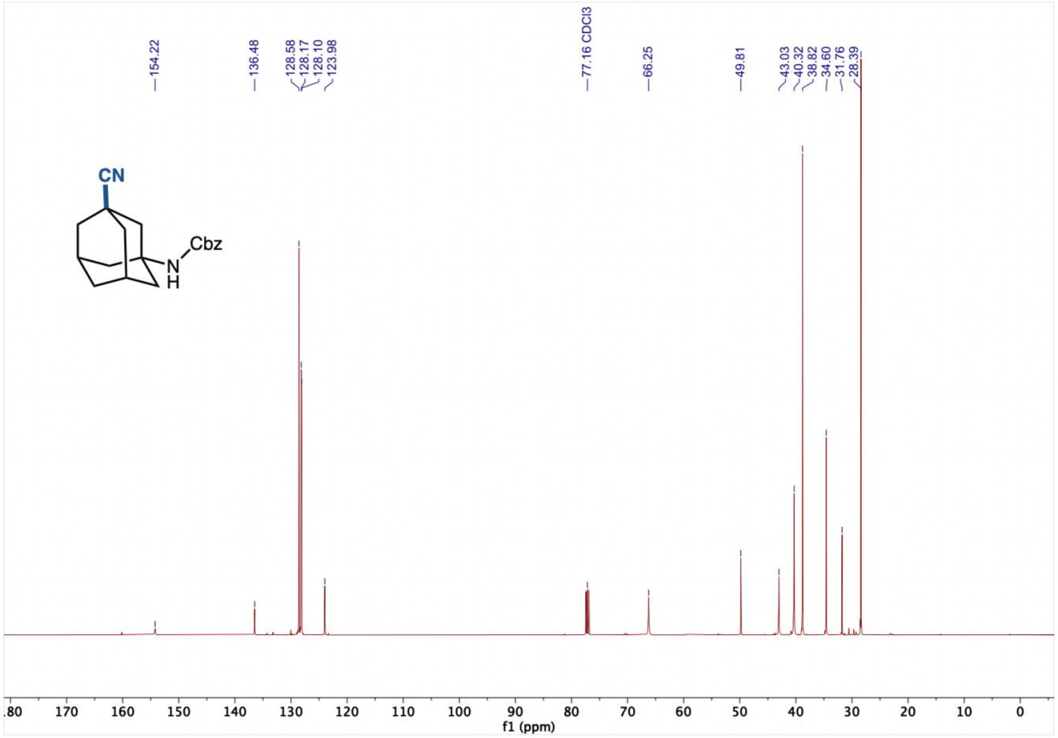

**Tert-butyl 6-hydroxy-6-methyl-2-azaspiro[3.3]heptane-2-carboxylate (30)**

$^{1}$H NMR (400 MHz, $CDCl_3$)

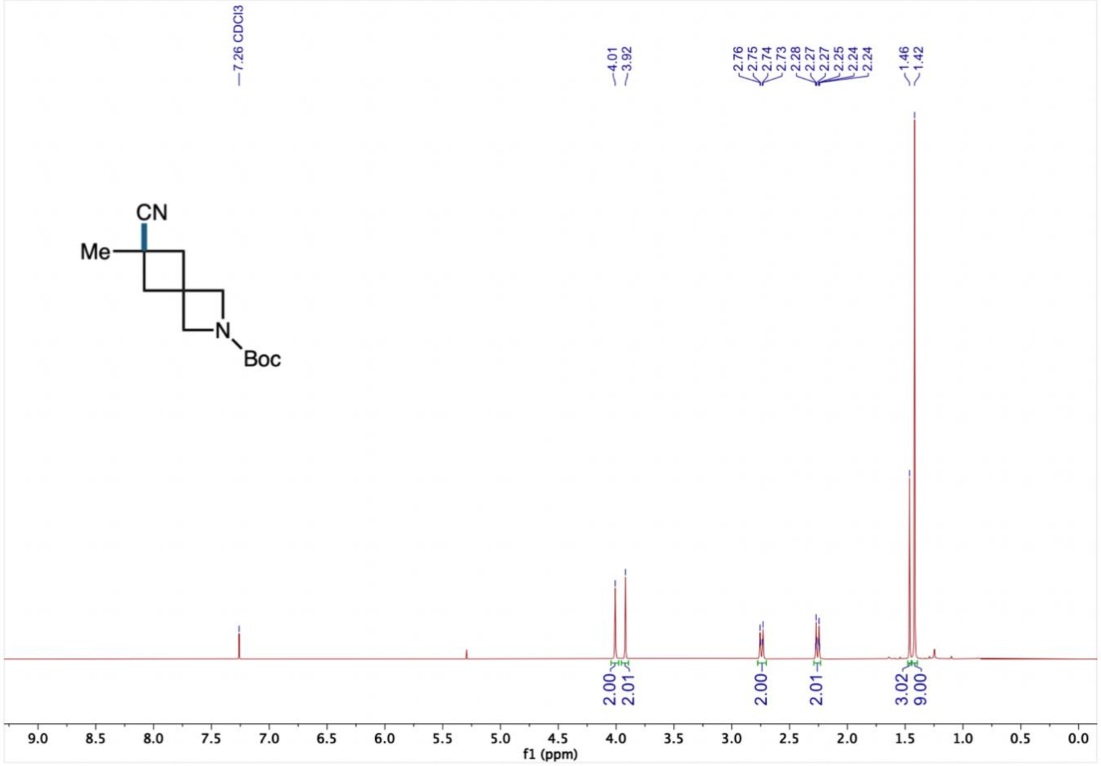


$^{13}$C NMR (126 MHz, $CDCl_3$)

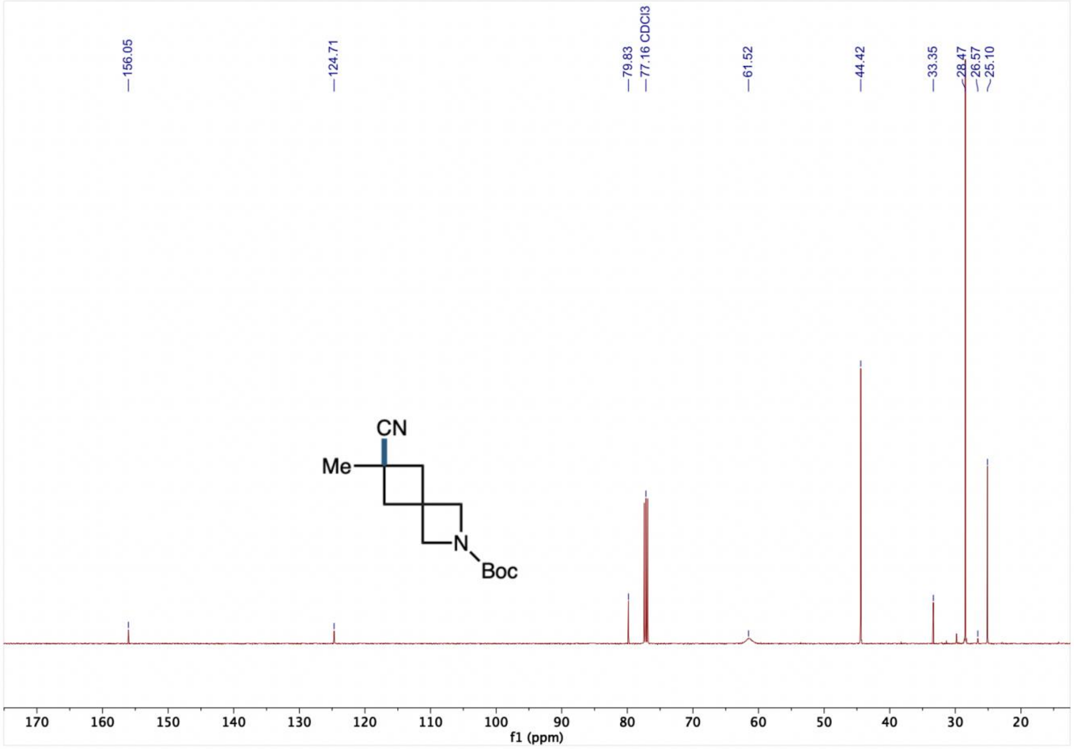

**(±)-*Tert*-butyl-7-cyano-7-methyl-5-oxa-2-azaspiro[3.4]octane-2-carboxylate (31)**

$^{1}H$ NMR (400 MHz, $CDCl_3$)

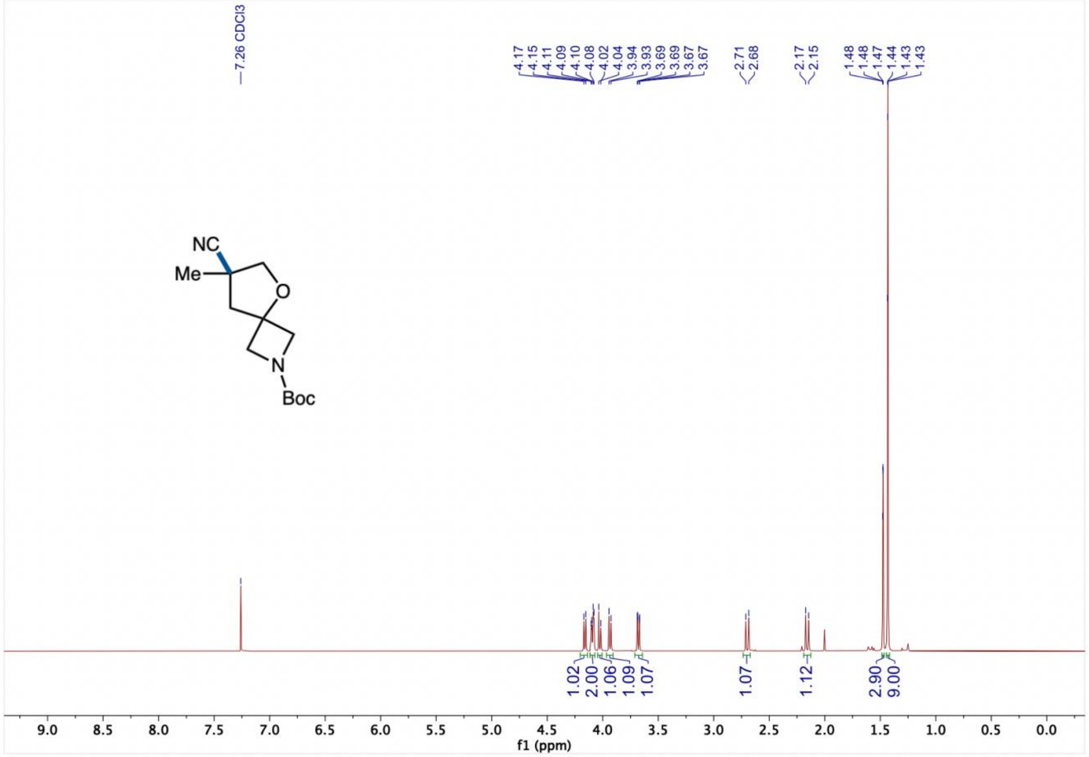


$^{13}C$ NMR (126 MHz, $CDCl_3$)

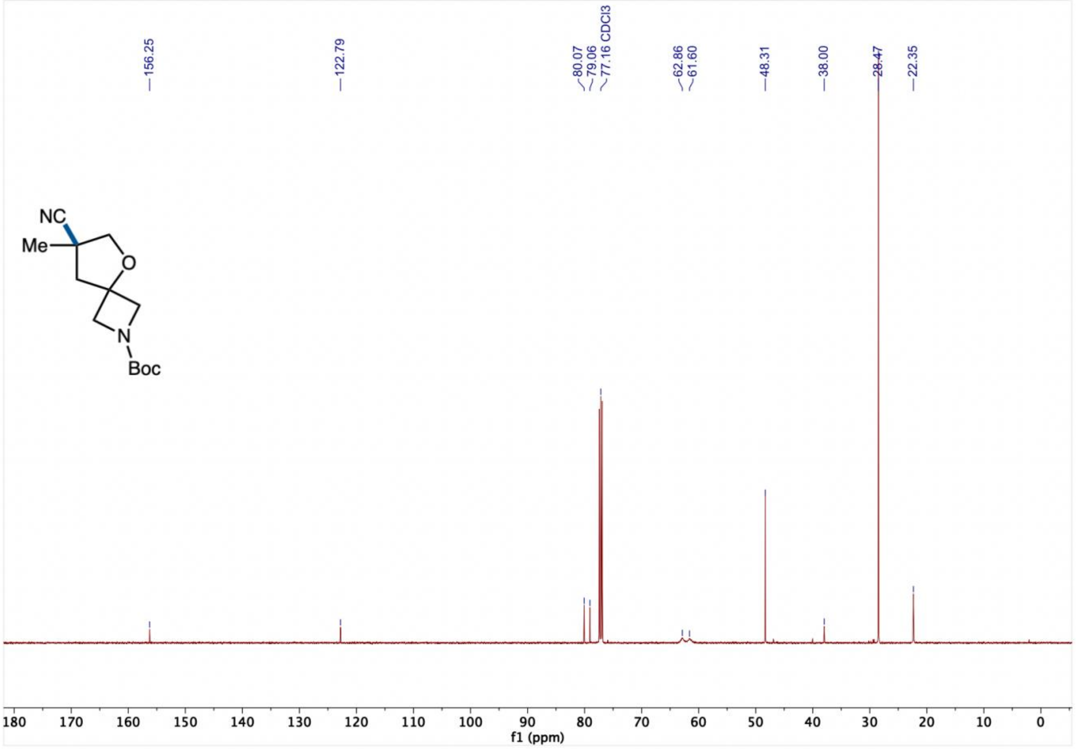

**(±)-*Tert*-butyl 6-cyano-2-azaspiro[3.4]octane-2-carboxylate (32)**

$^{1}H$ NMR (400 MHz, $CDCl_3$)

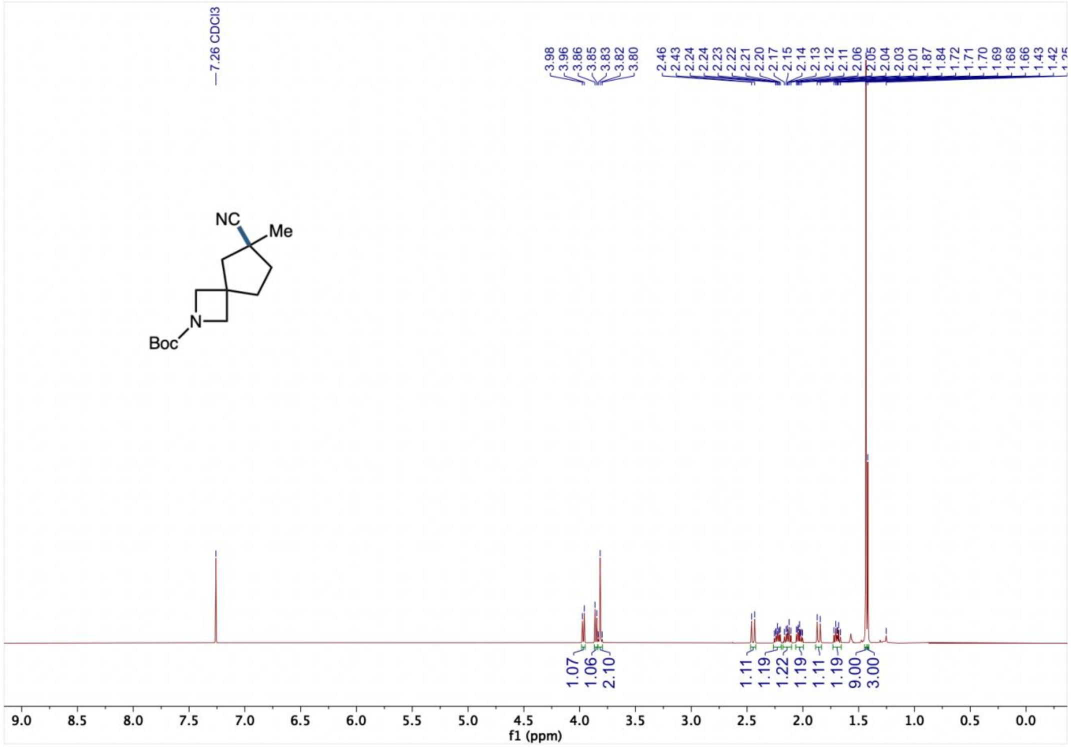

$^{13}C$ NMR (126 MHz, $CDCl_3$)

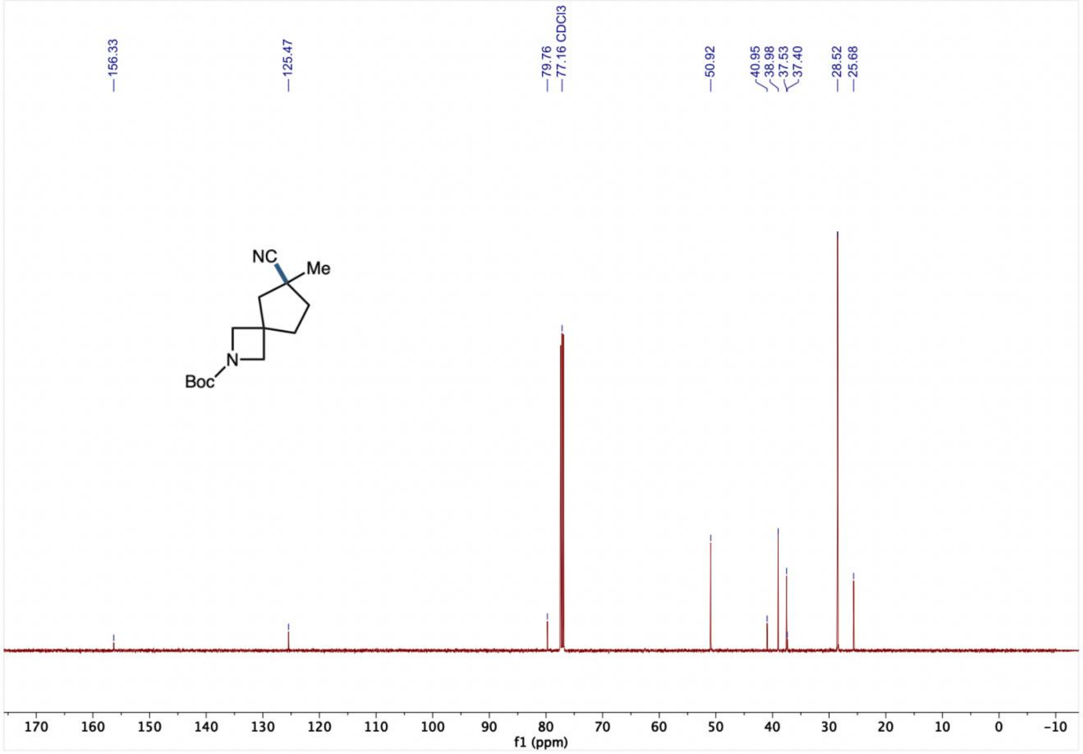

***Tert*-butyl 2-cyano-2-methyl-7-azaspiro[3.5]nonane-7-carboxylate (33)**

$^{1}$H NMR (400 MHz, $CDCl_3$)

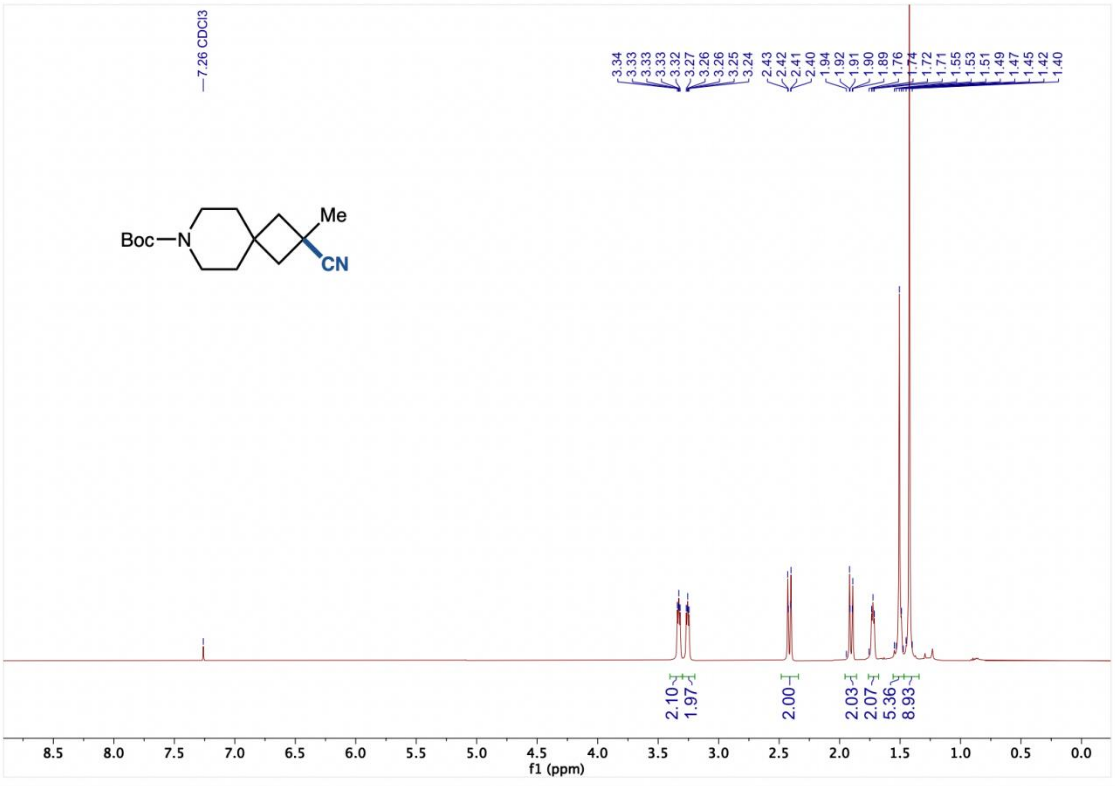

$^{13}$C NMR (126 MHz, $CDCl_3$)

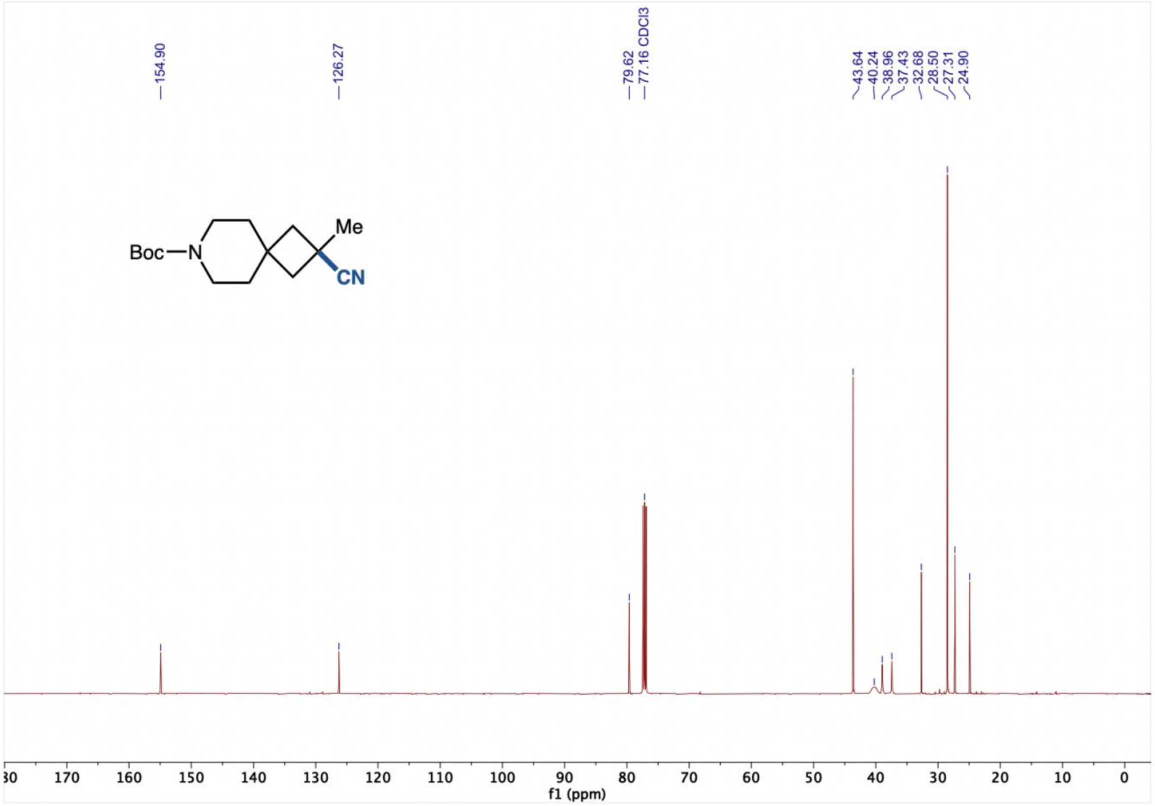

## (±)-Benzyl 5-cyano-5-methyl-2-azabicyclo[2.2.1]heptane-2-carboxylate (34)

$^{1}H$ NMR (400 MHz, $CDCl_3$)

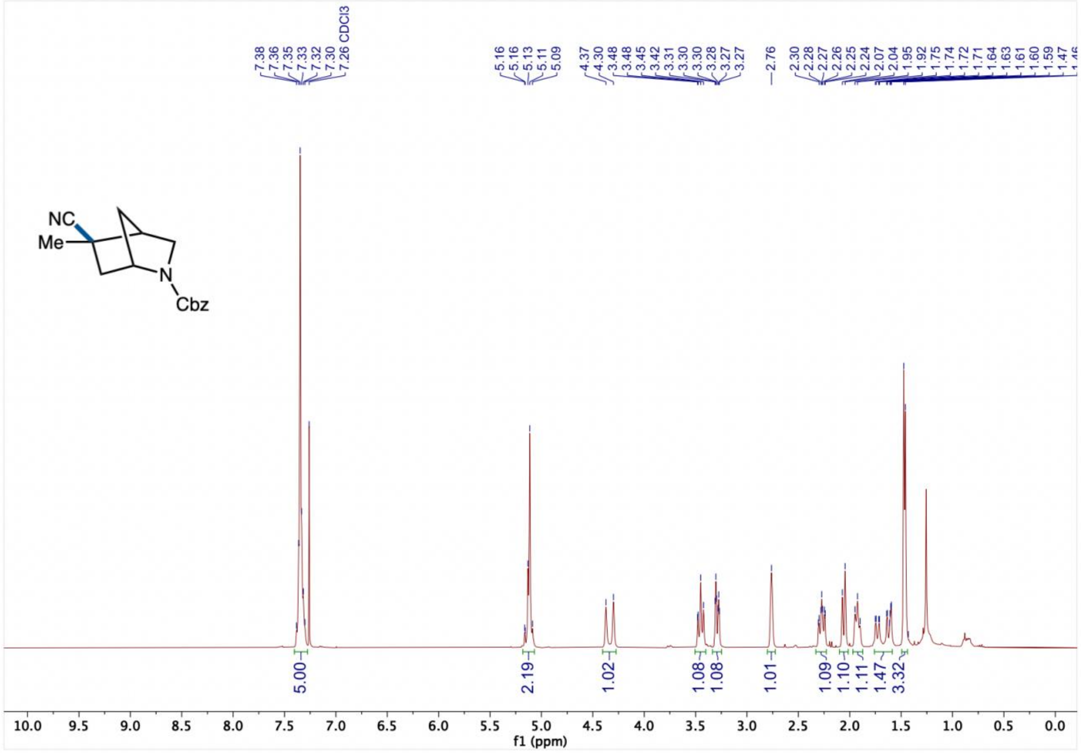


$^{13}C$ NMR (126 MHz, $CDCl_3$)

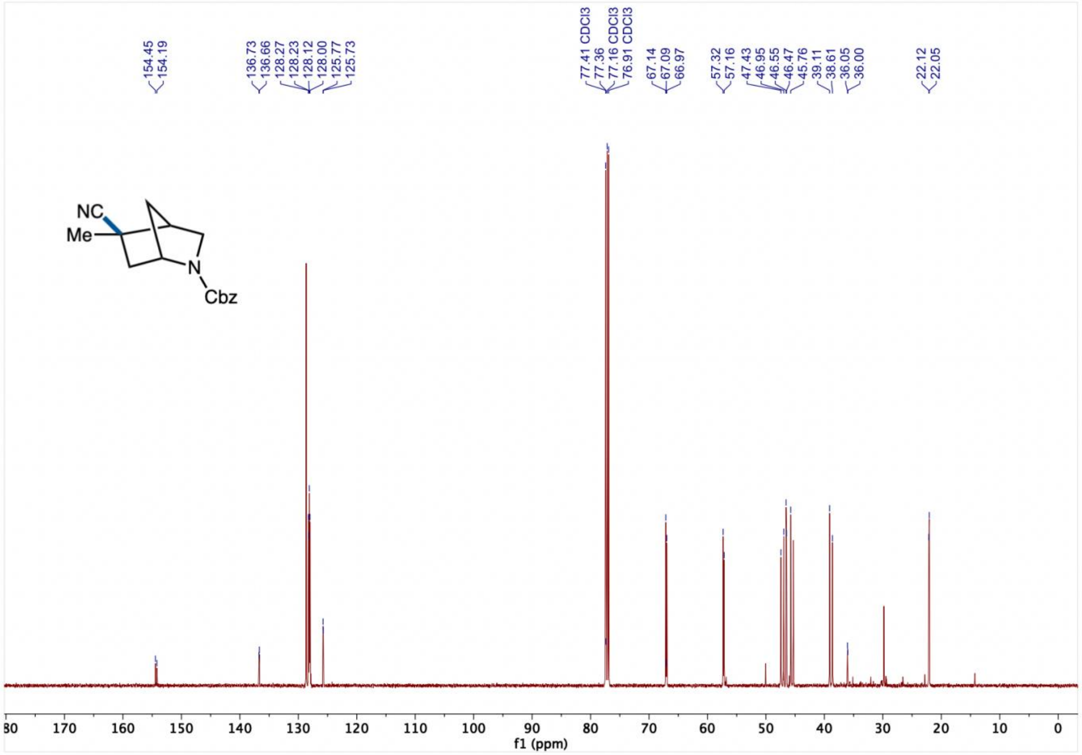

## 4,4-Diphenylbutanenitrile (35)

$^1$H NMR (400 MHz, $CDCl_3$)

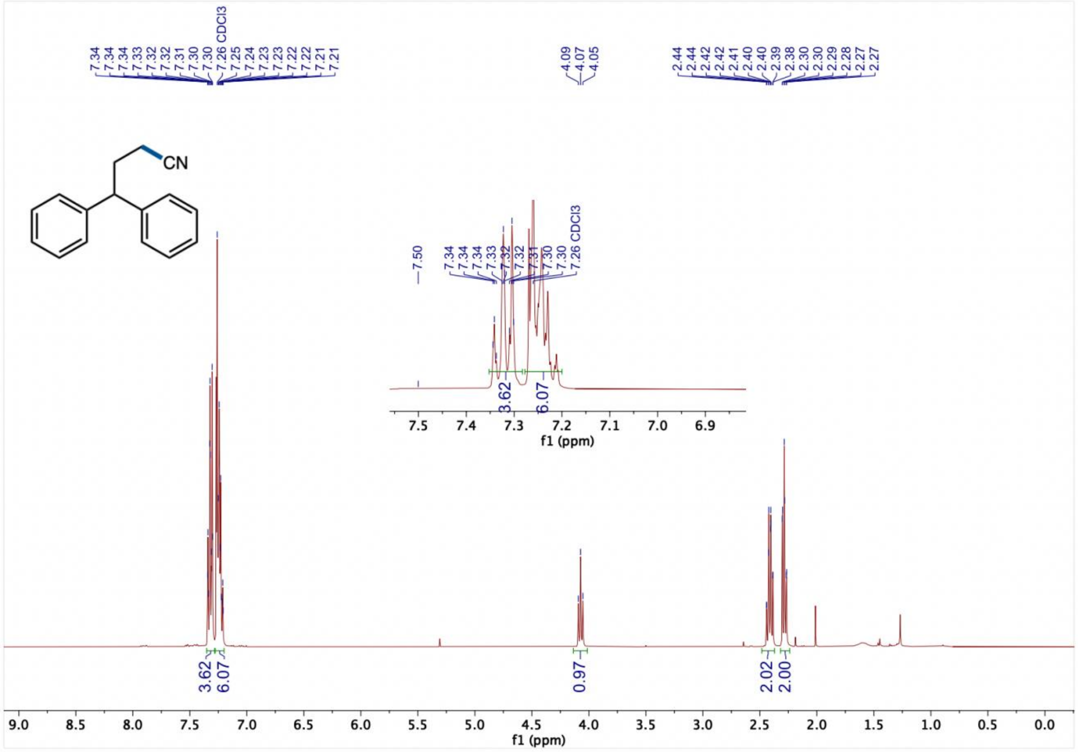

$^{13}$C NMR (126 MHz, $CDCl_3$)

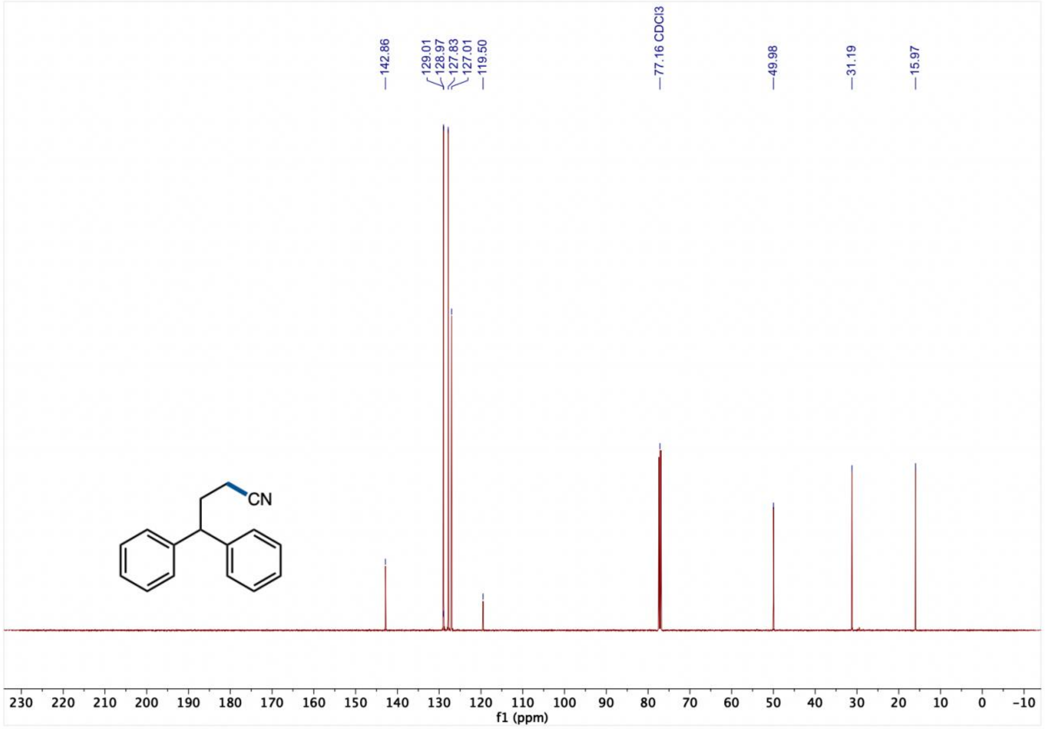

## *Tert*-butyl N-(1-benzyl-2-cyano-ethyl)carbamate (36)

$^1$H NMR (400 MHz, $CDCl_3$)

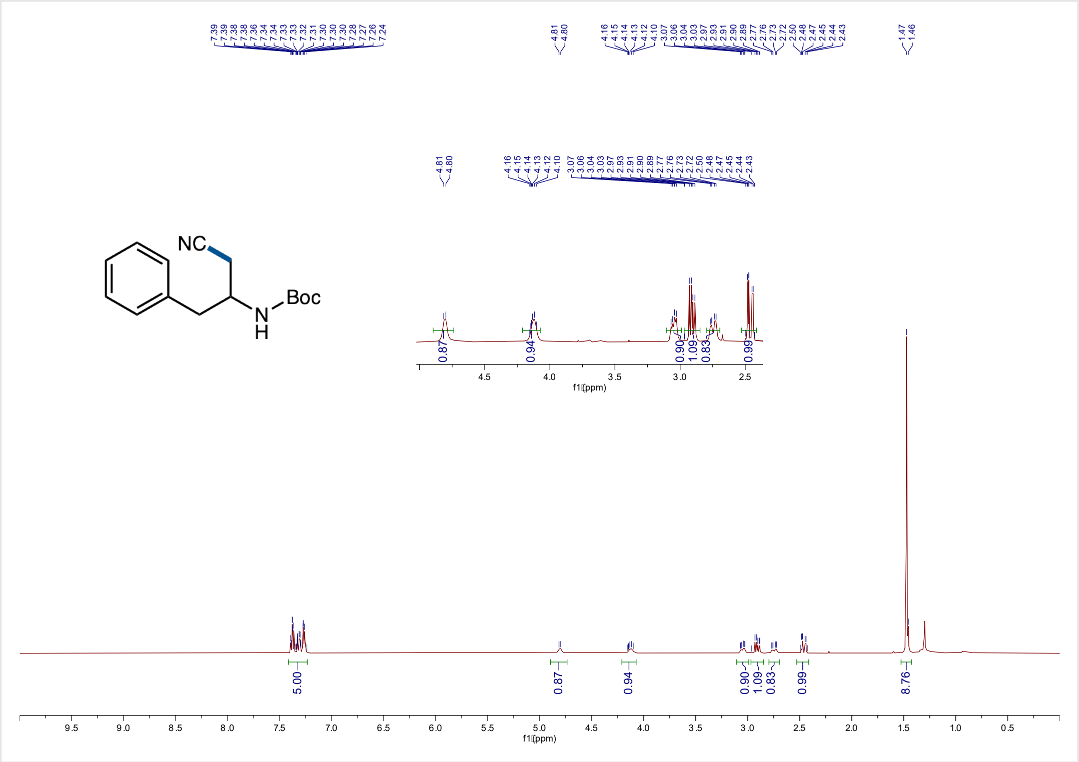

$^{13}$C NMR (126 MHz, $CDCl_3$)

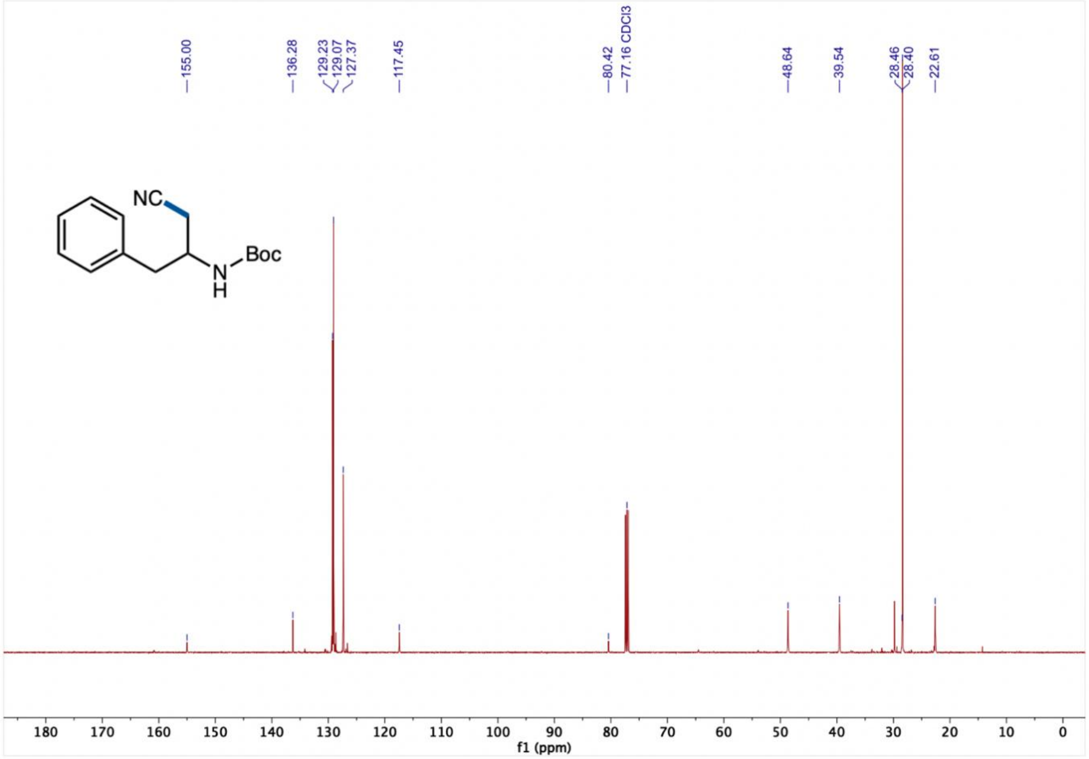

## *Tert*-butyl 2-(2-cyanoethyl)piperidine-1-carboxylate (37)

$^{1}H$ NMR (400 MHz, $CDCl_3$)

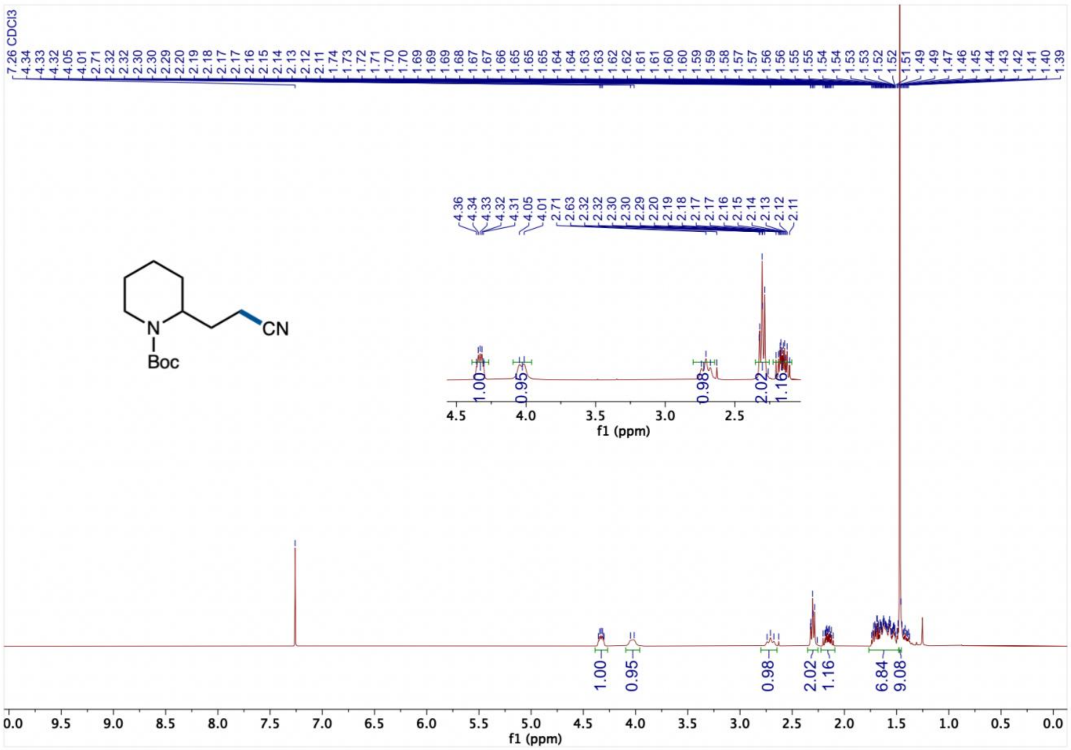


$^{13}C$ NMR (126 MHz, $CDCl_3$)

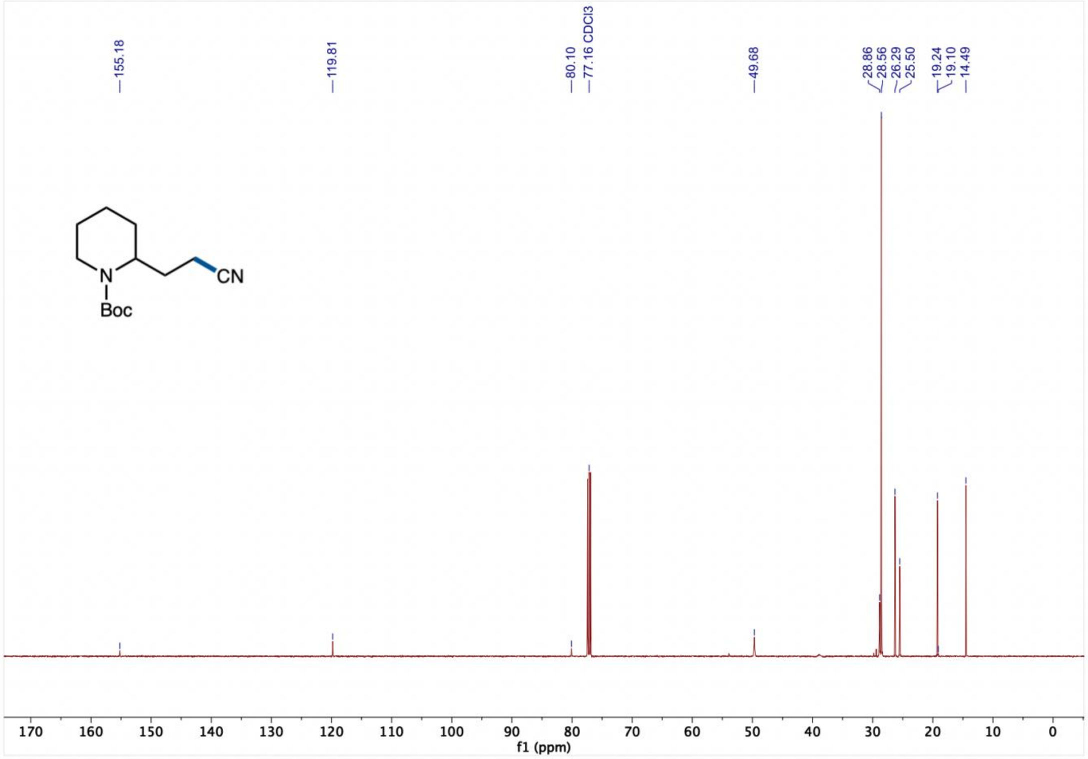

***Tert*-butyl 4-(2-cyanoethyl)piperidine-1-carboxylate (38)**

$^1$H NMR (400 MHz, $CDCl_3$)

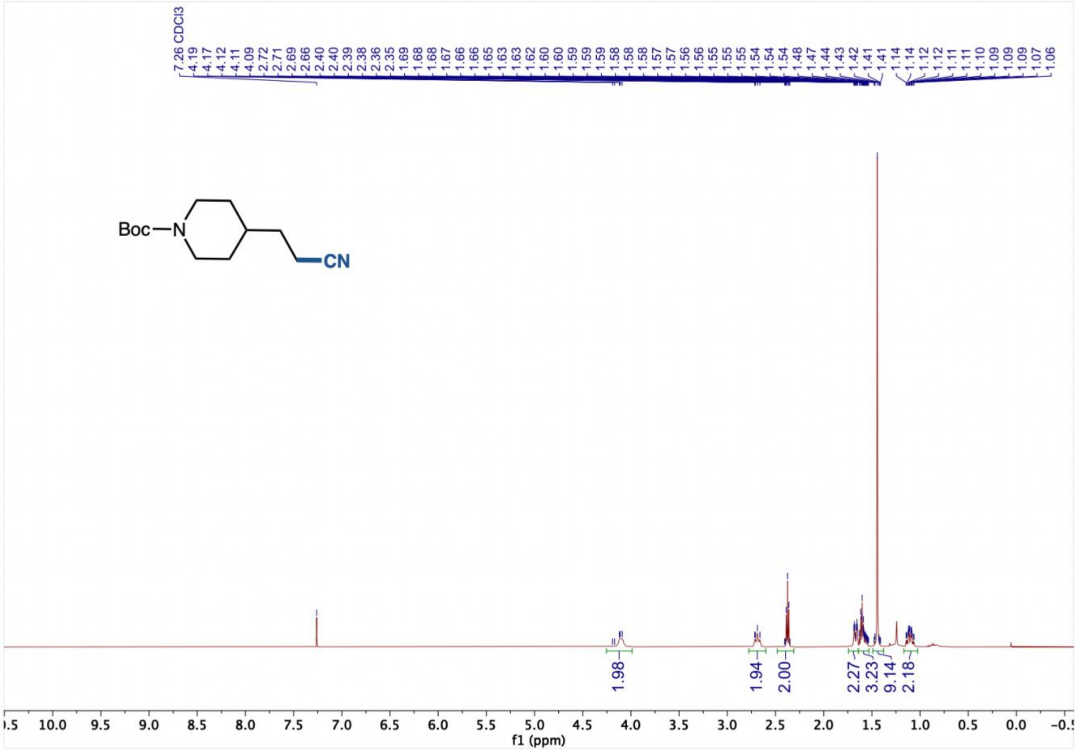

$^{13}$C NMR (126 MHz, $CDCl_3$)

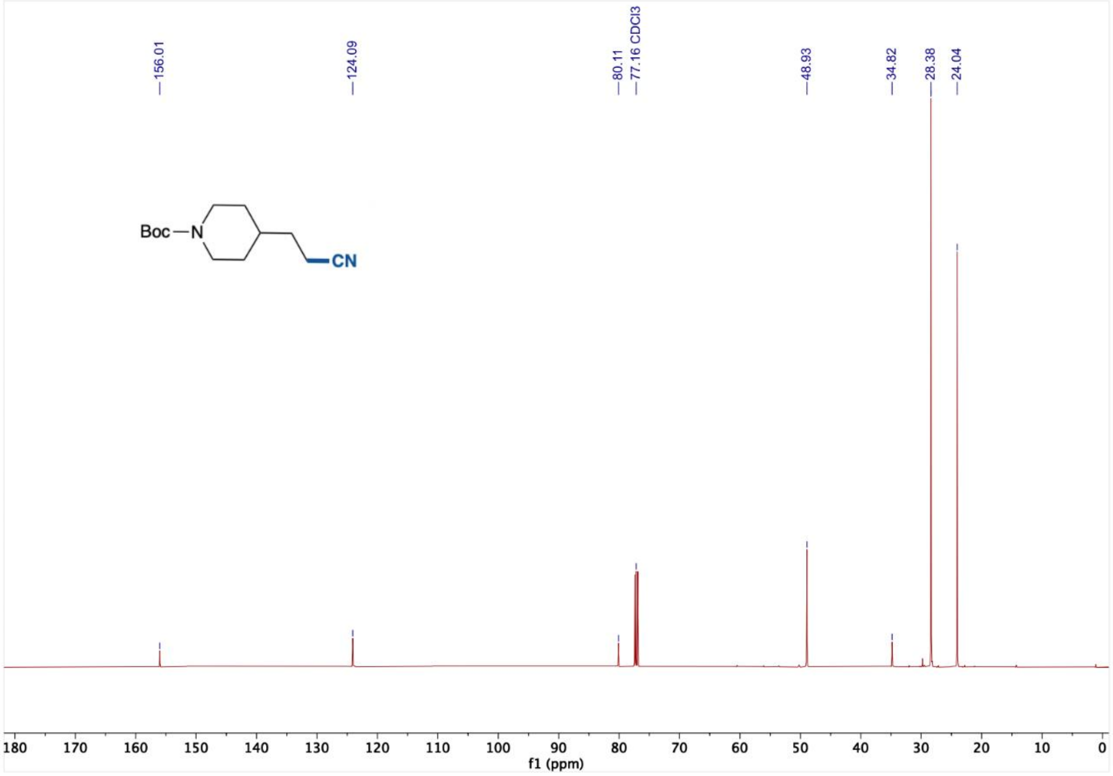

**4-(3-(4-cyanobutyl)-4,4-dimethyl-2,5-dioxoimidazolidin-1-yl)-2-(trifluoromethyl)benzonitrile (39)**

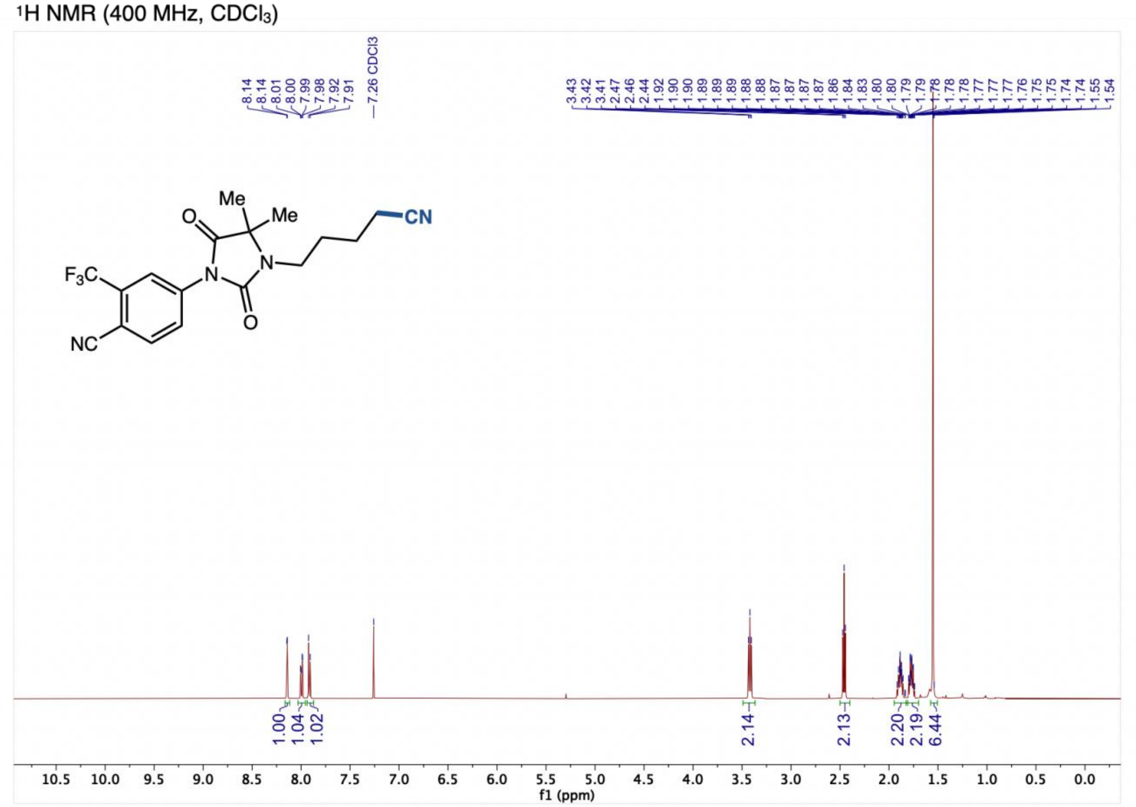


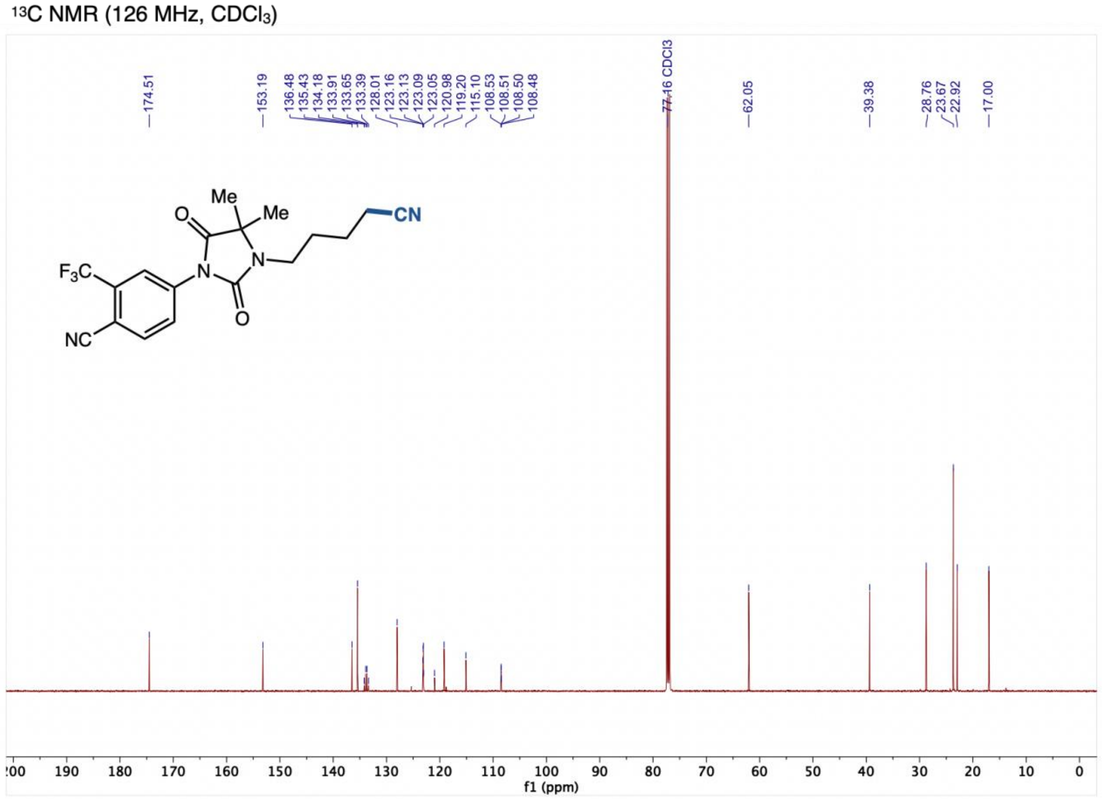

$^{19}$F NMR (471 MHz, CDCl3)

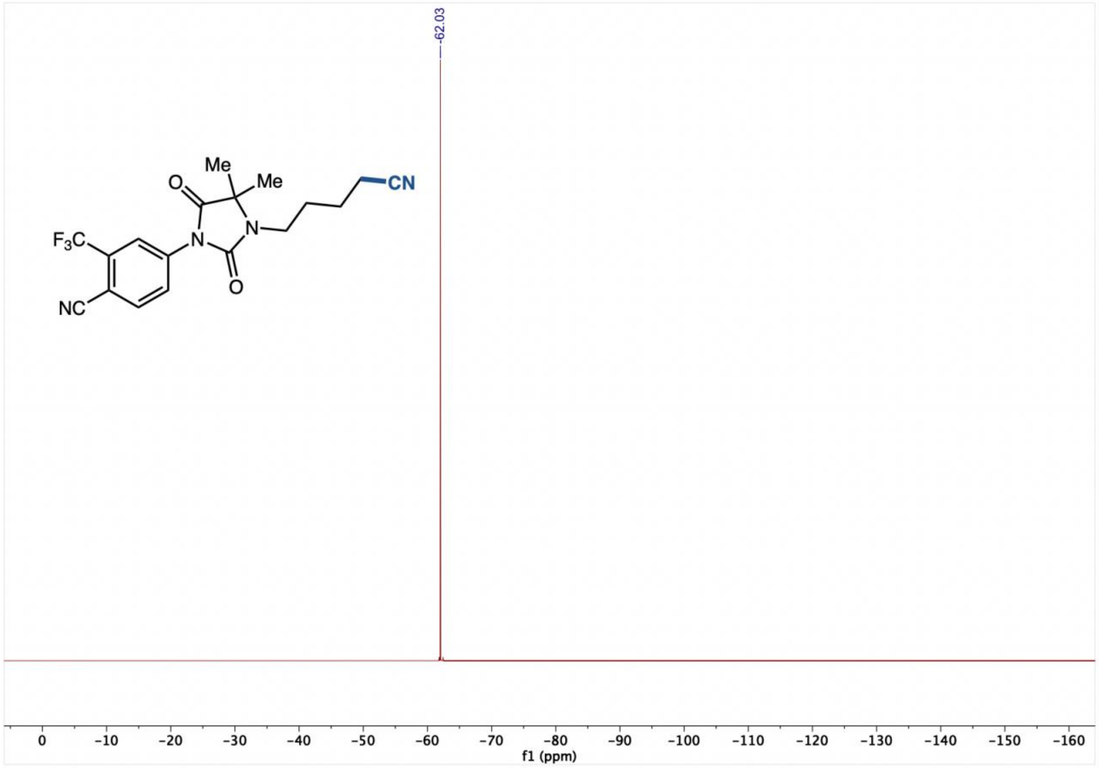


***Tert*-butyl ((*S*)-2-((1*S*,3*S*,5*S*)-3-cyano-2-azabicyclo[3.1.0]hexan-2-yl)-1-((1*r*,3*R*,5*R*,7*S*)-3-cyanoadamantan-1-yl)-2-oxoethyl)carbamate (40)**

$^{1}$H NMR (400 MHz, CDCl3)

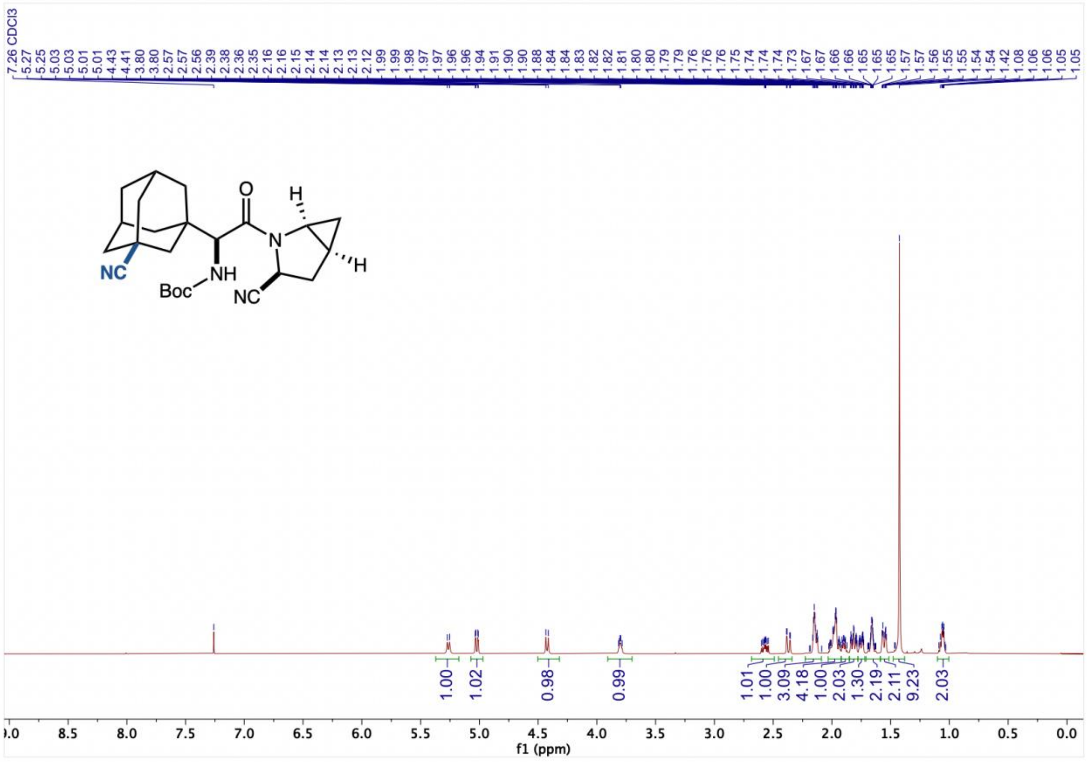

$^{13}C$ NMR (126 MHz, $CDCl_3$)

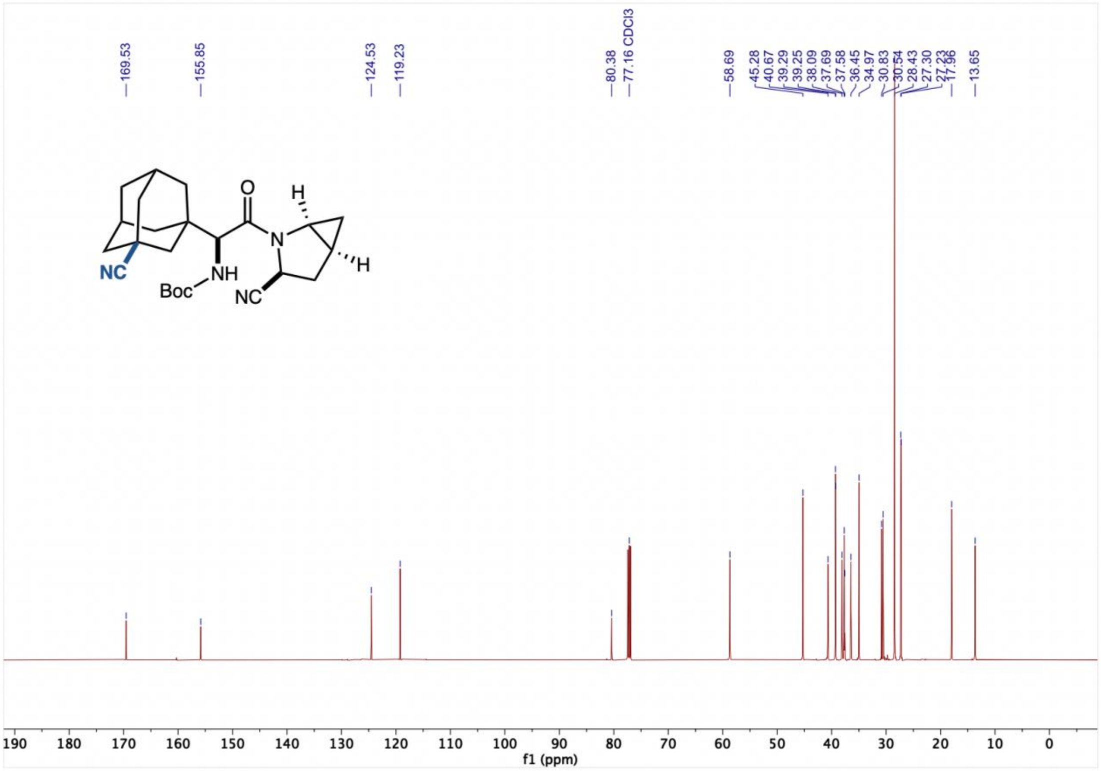


**(±)-1-(2-(4-isobutylphenyl)propanoyl)pyrrolidine-3-carbonitrile (41)**

$^{1}H$ NMR (500 MHz, $CDCl_3$)

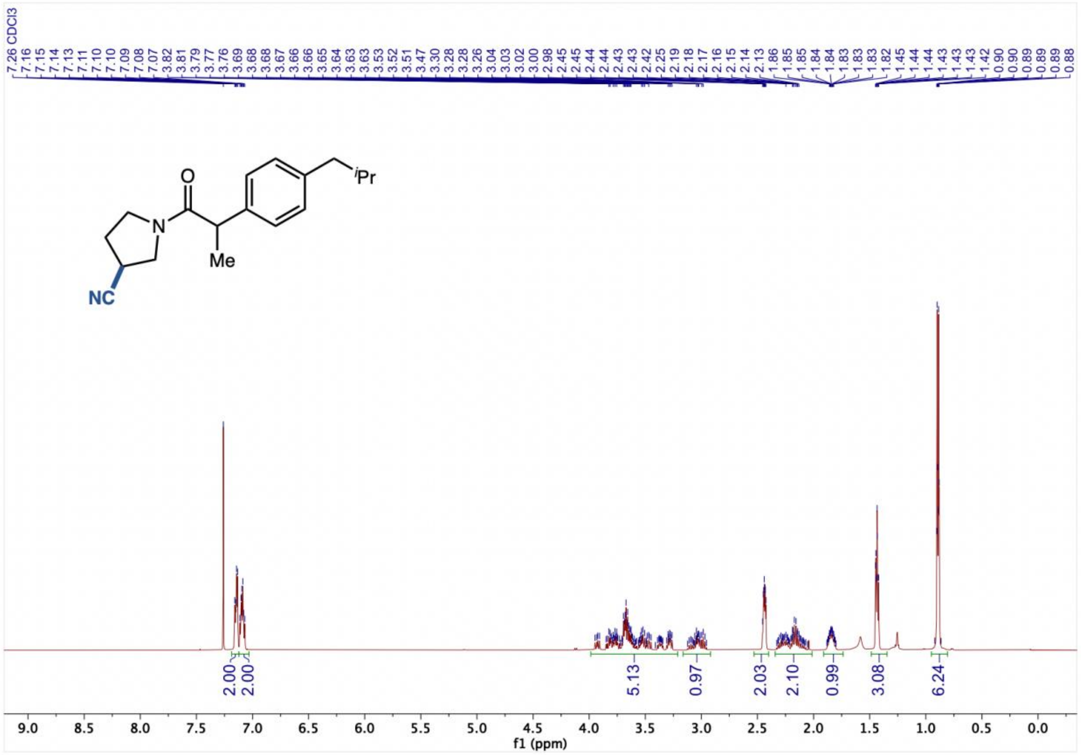

$^{13}C$ NMR (126 MHz, $CDCl_3$)

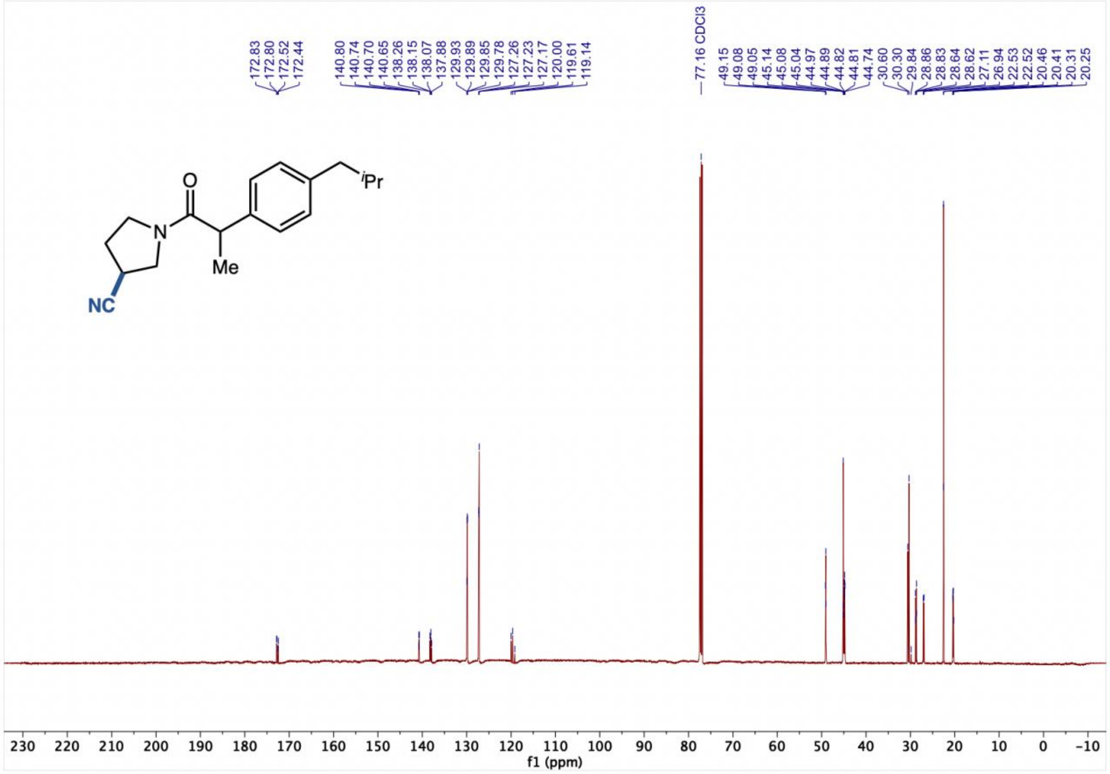


**(±)-1-(3-(2-cyanobenzyl)-1-methyl-2,6-dioxo-1,2,3,6-tetrahydropyridin-4-yl)piperidine-3-carbonitrile (42)**

$^{1}H$ NMR (500 MHz, $CDCl_3$)

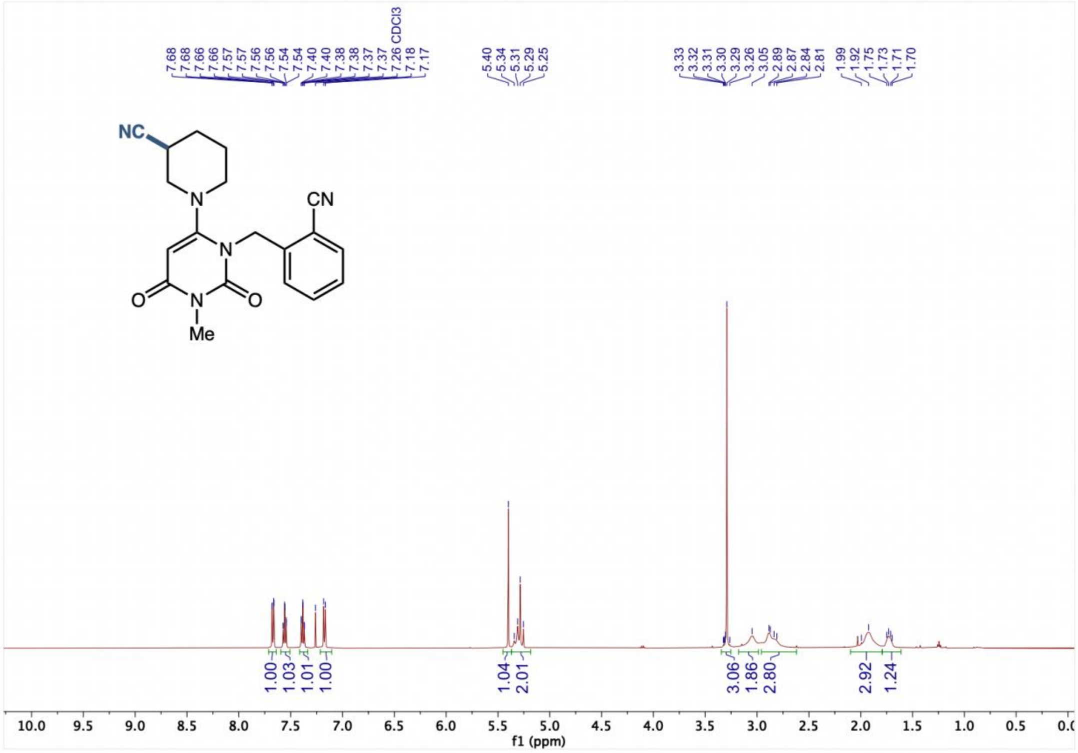

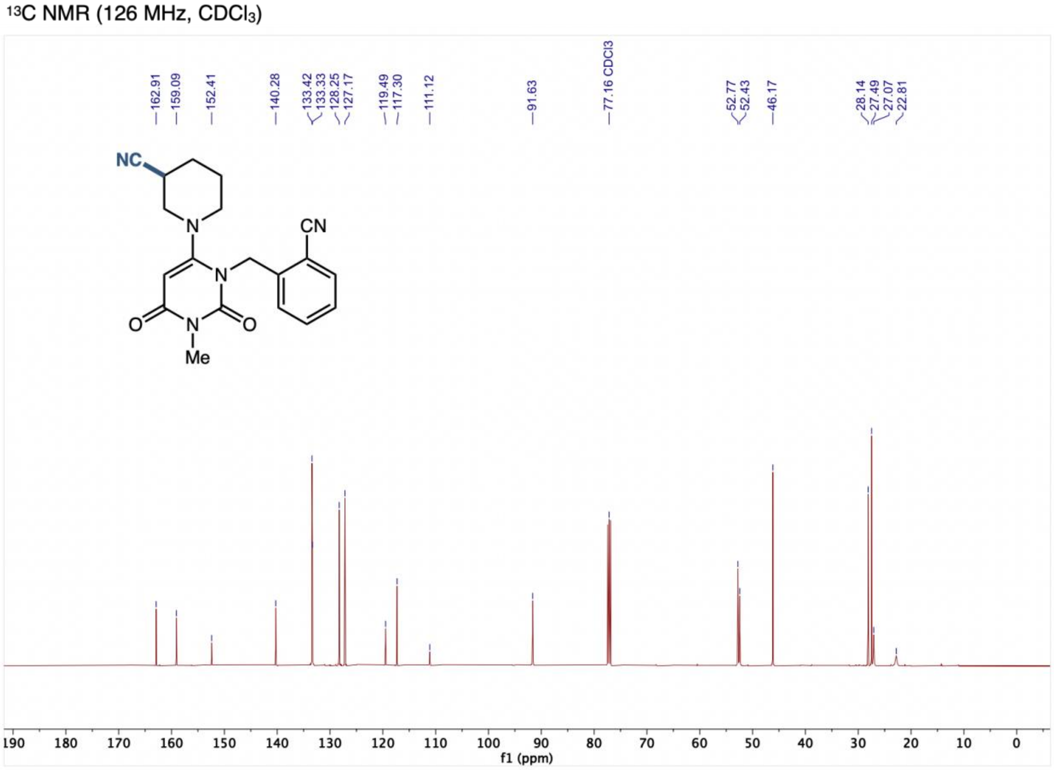


**(*S*)-*N*-((1*s*,3*S*,5*R*,7*S*)-3-cyanoadamantan-1-yl)-2-((4,6-dimethylpyrimidin-2-yl)oxy)-3-methoxy-3,3-diphenylpropanamide (43)**

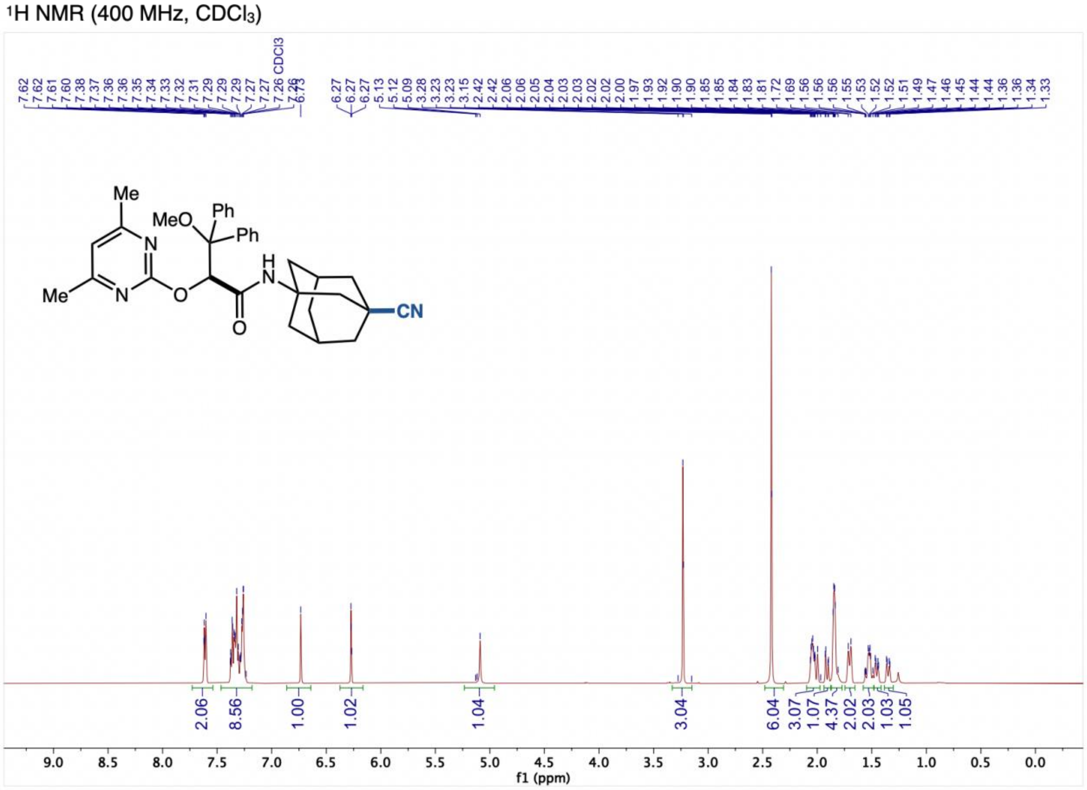

$^{13}C$ NMR (126 MHz, $CDCl_3$)

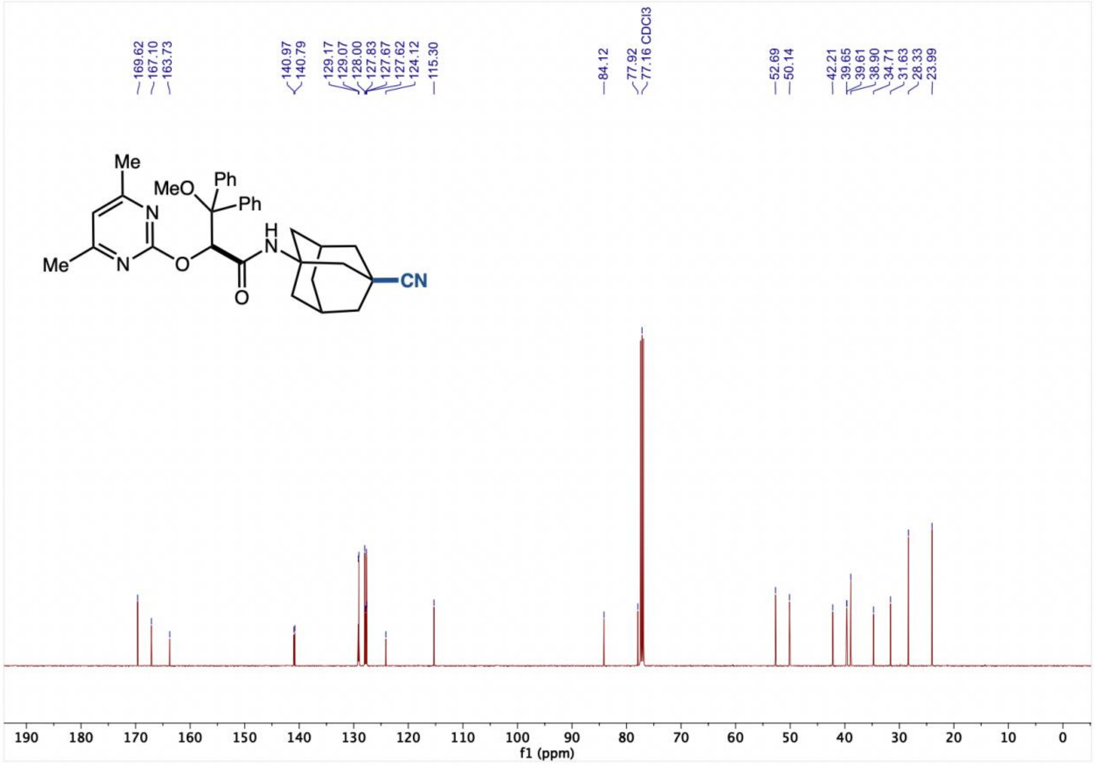


***Tert*-butyl N-[rac-(1S)-2,2-dimethyl-1-[rac-(2S,4R)-4-cyano-2-[[4-(4-methylthiazol-5-yl)phenyl]methylcarbamoyl]pyrrolidine-1-carbonyl]propyl]carbamate (44)**

$^{1}H$ NMR (500 MHz, $CDCl_3$)

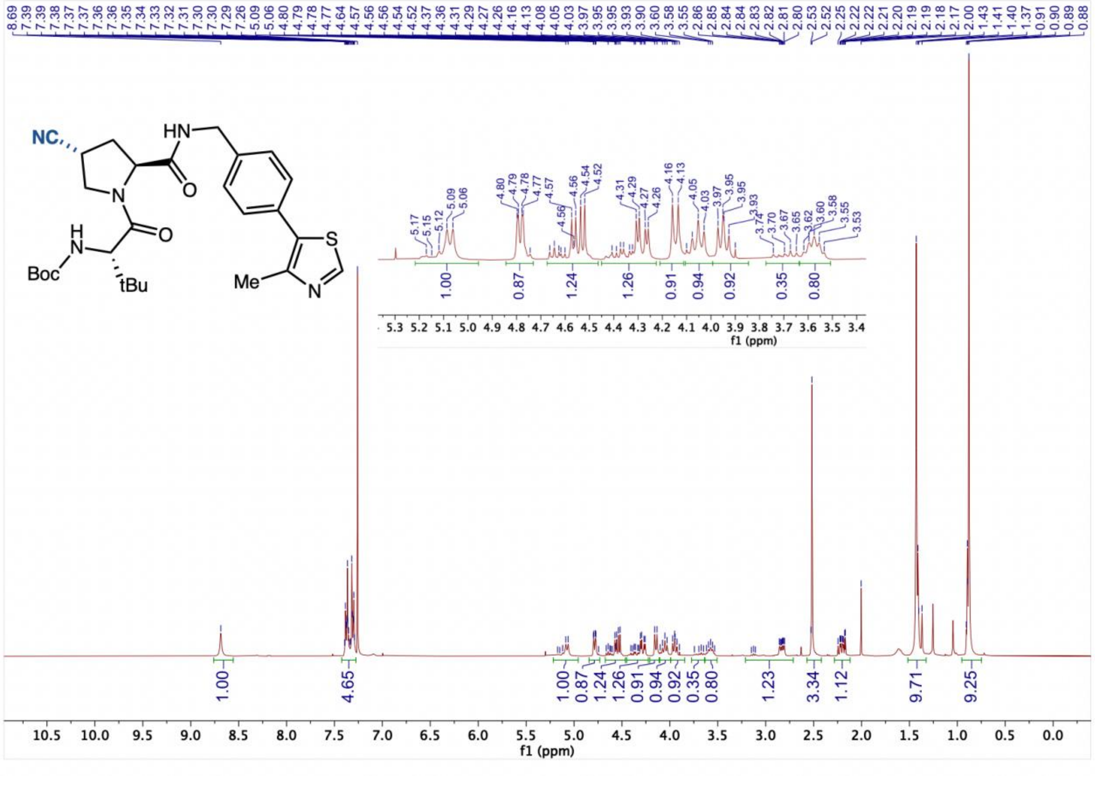

$^{13}C$ NMR (126 MHz, $CDCl_3$)

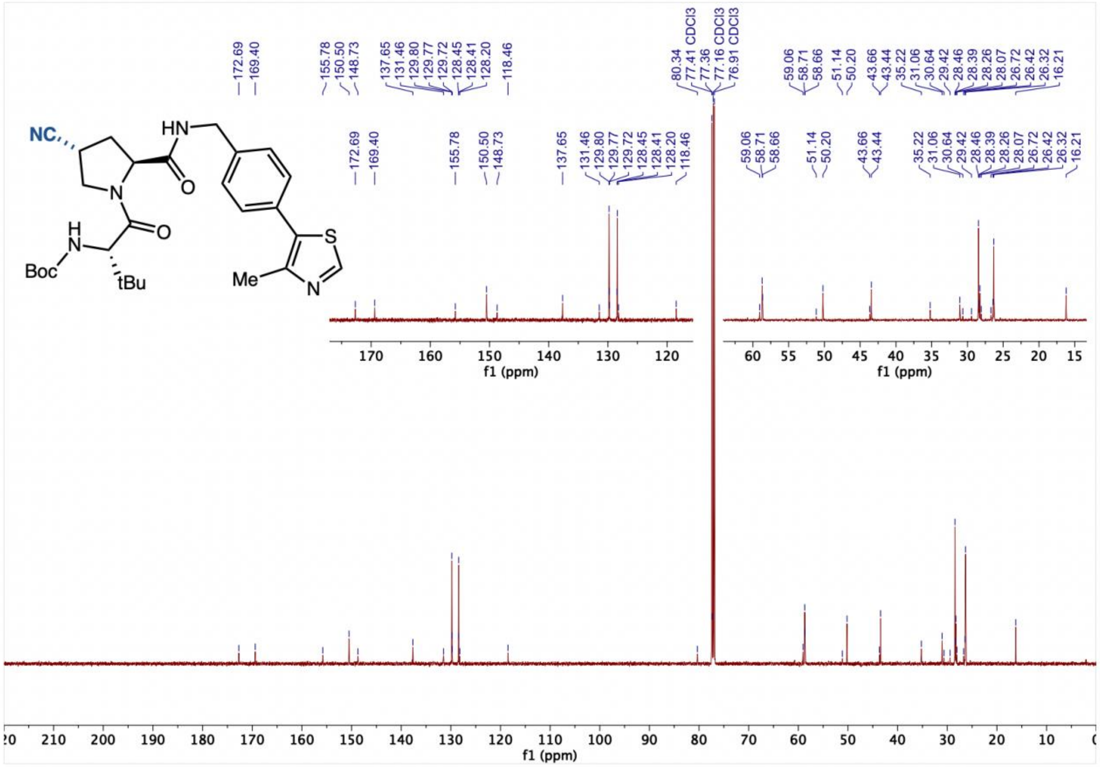


$^{1}H$ NMR (500 MHz, $CDCl_3$)

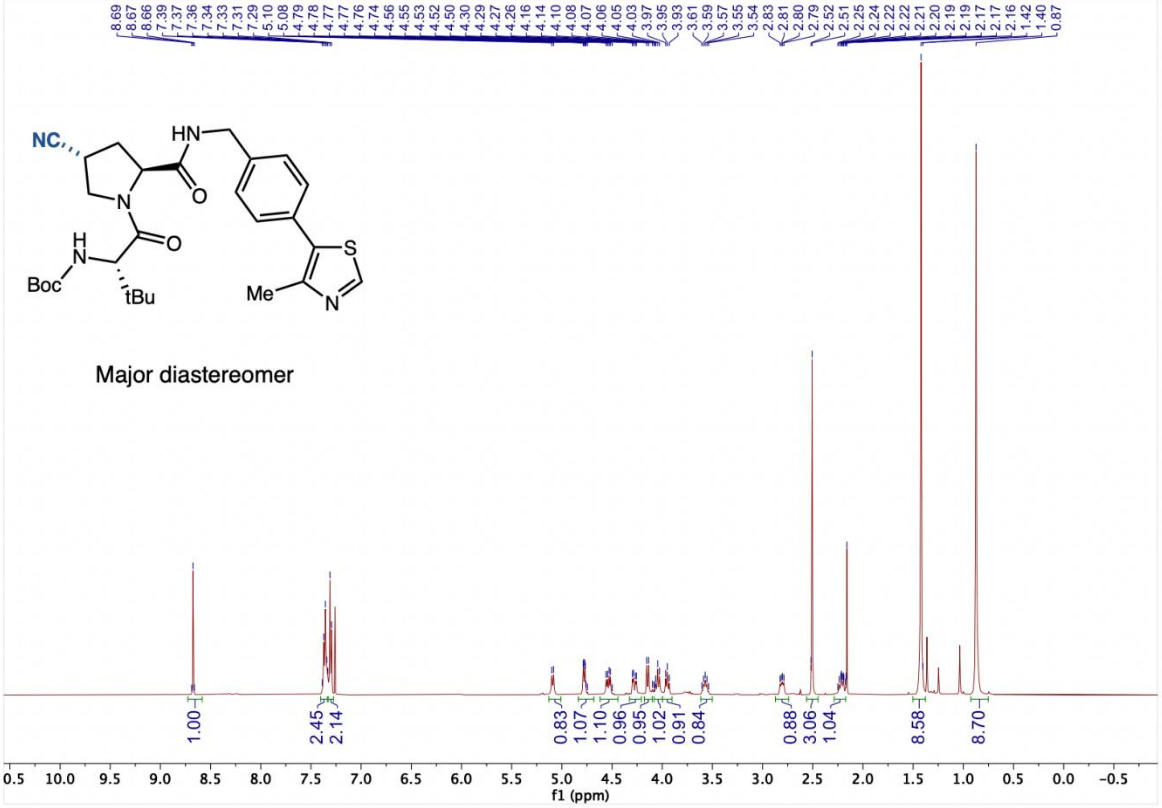

$^{13}C$ NMR (126 MHz, $CDCl_3$)

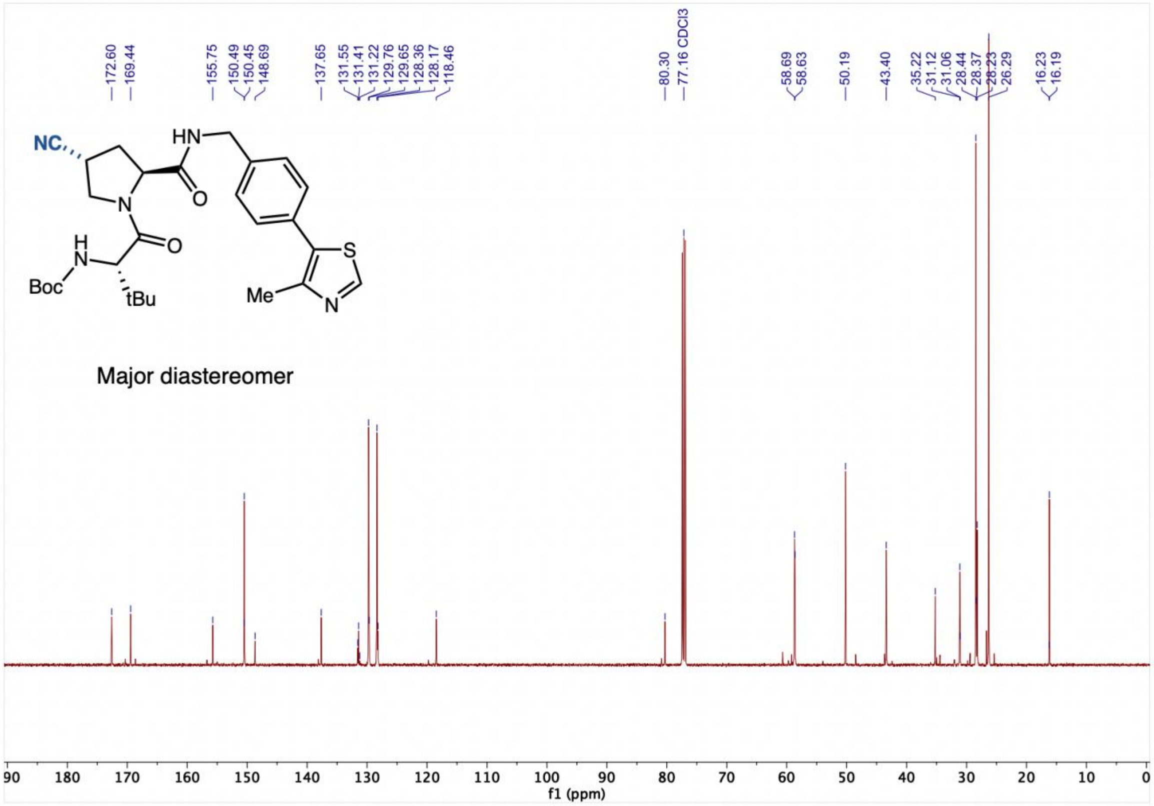


NOESY (500 MHz, $CDCl_3$)

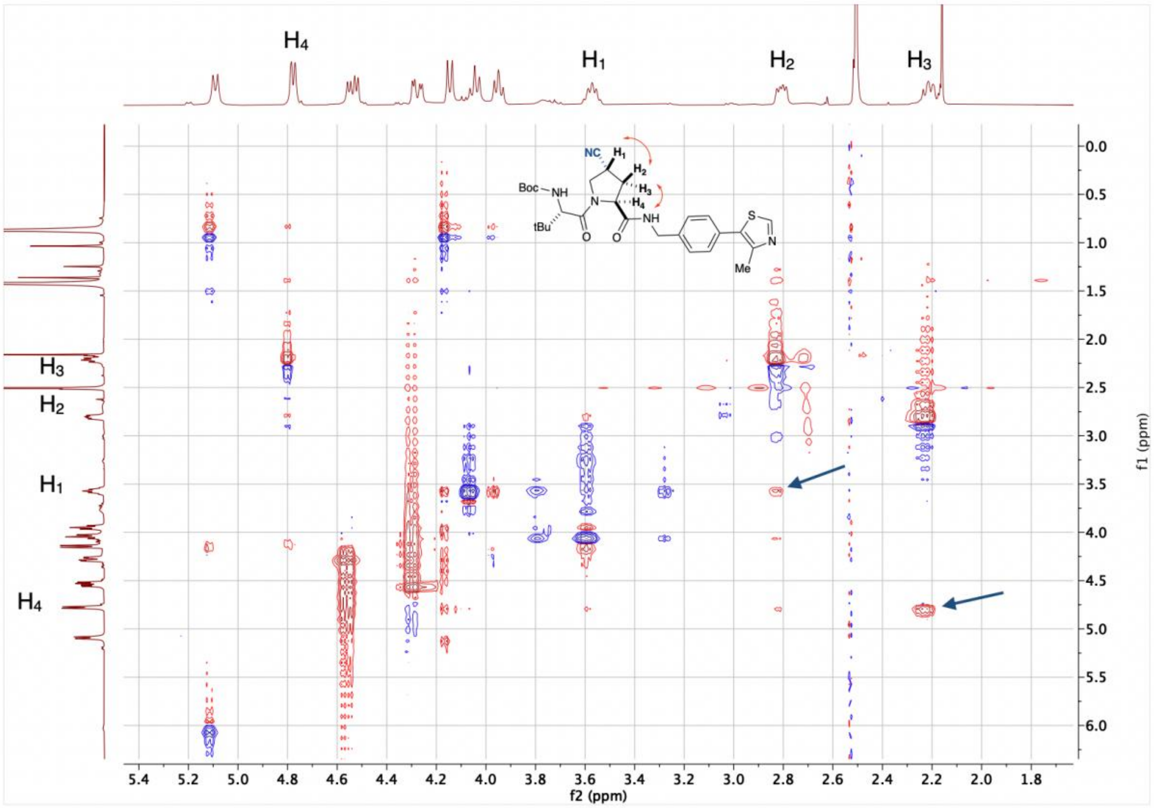

**Benzyl N-[rac-(3aS,4R,6aR)-6-cyano-2,2-dimethyl-4,5,6,6a-tetrahydro-3aH-cyclopenta[d][1,3]dioxol-4-yl]carbamate (45)**

$^{1}$H NMR (500 MHz, $CDCl_3$)

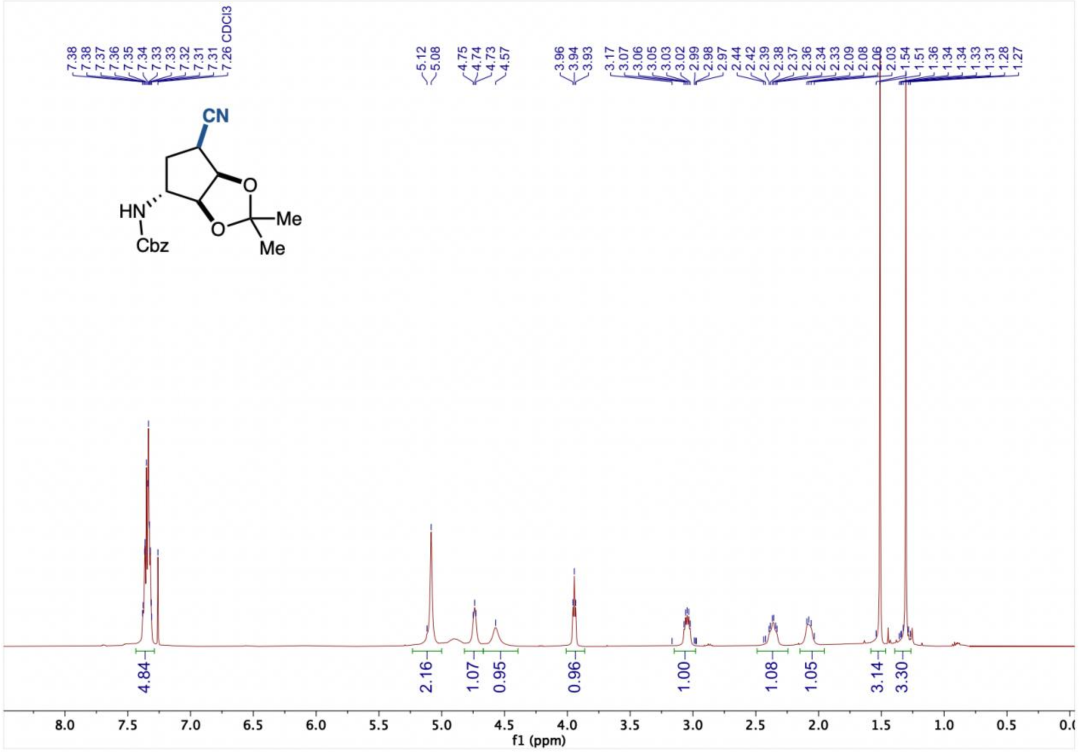

$^{13}$C NMR (126 MHz, $CDCl_3$)

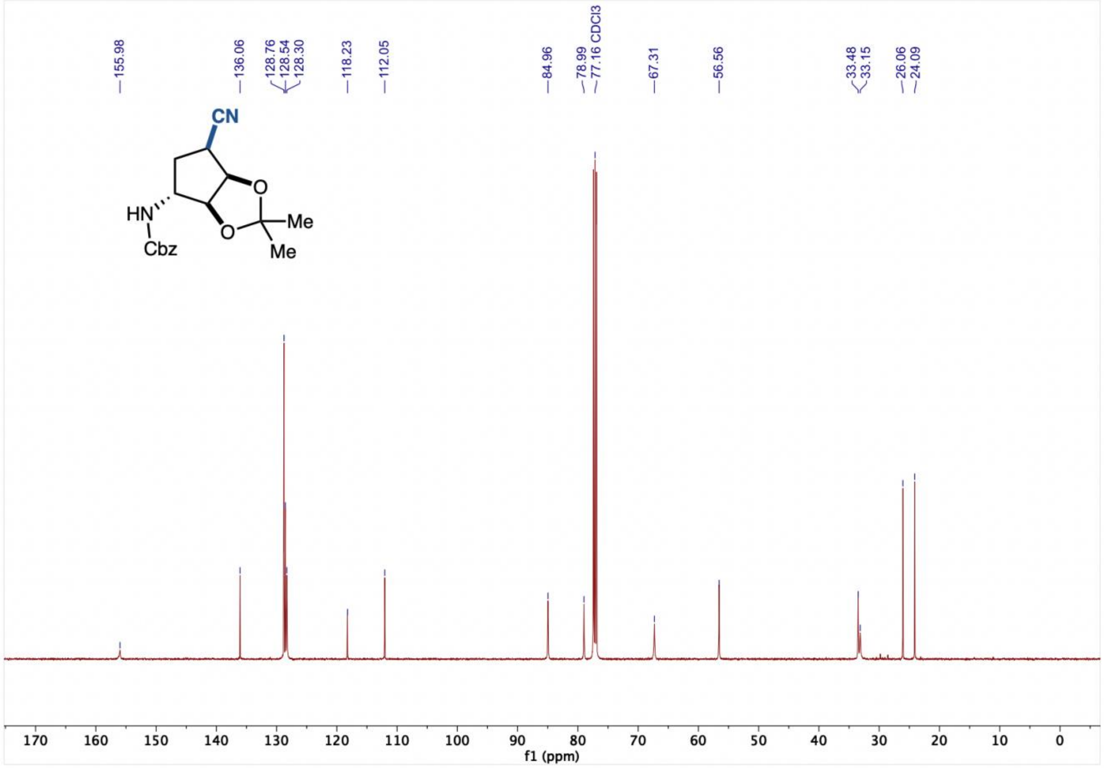

NOESY (500 MHz, $CDCl_3$)

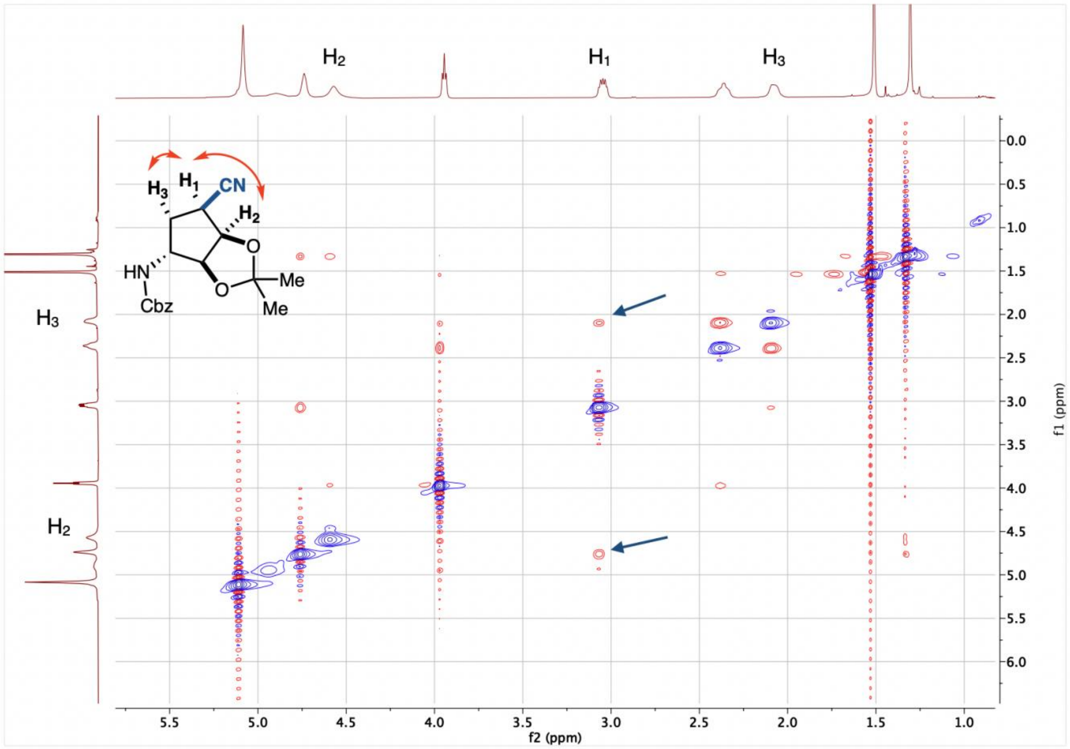

## (±)-Methyl-4-cyano-1-tosylpyrrolidine-2-carboxylate (46)

$^1H$ NMR (400 MHz, $CDCl_3$)

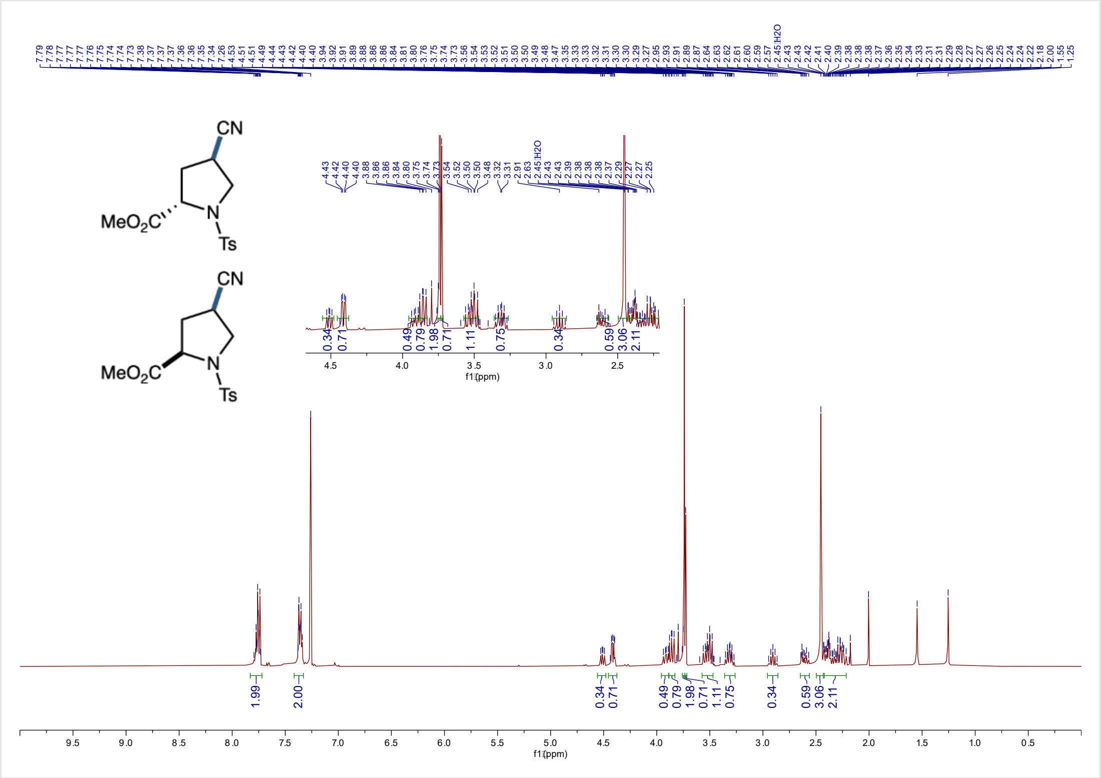

**Major diastereomer**

$^1$H NMR (400 MHz, $CDCl_3$)

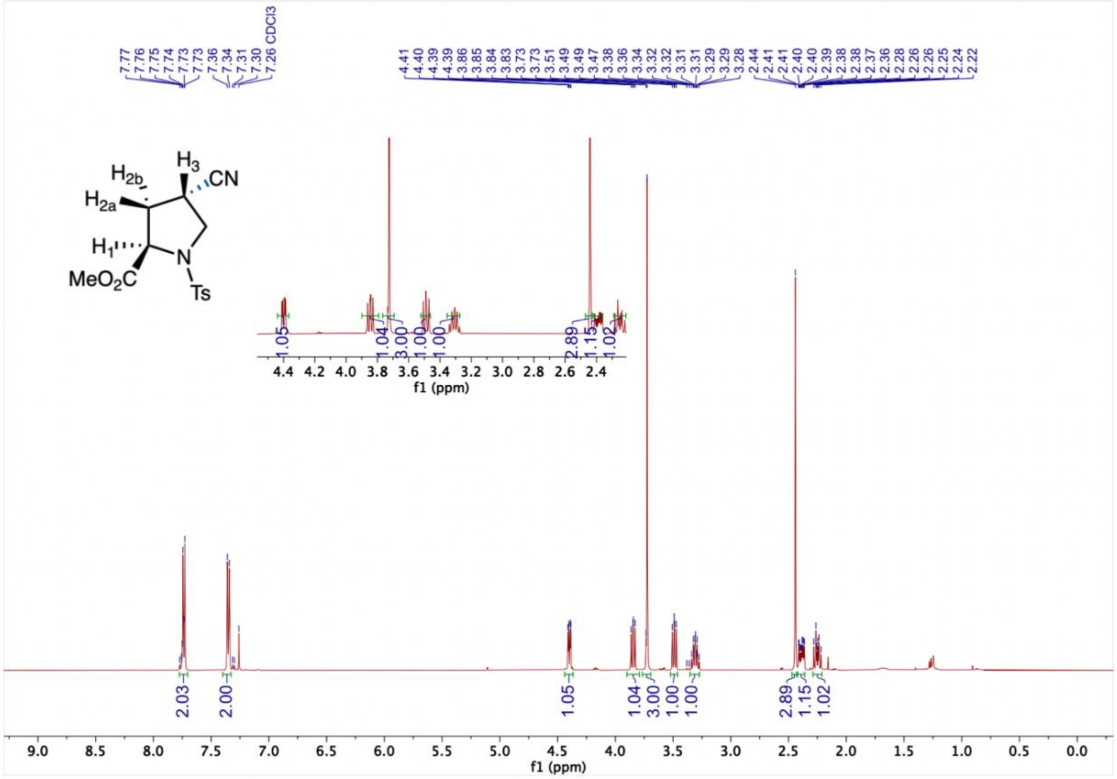

$^{13}$C NMR (126 MHz, $CDCl_3$)

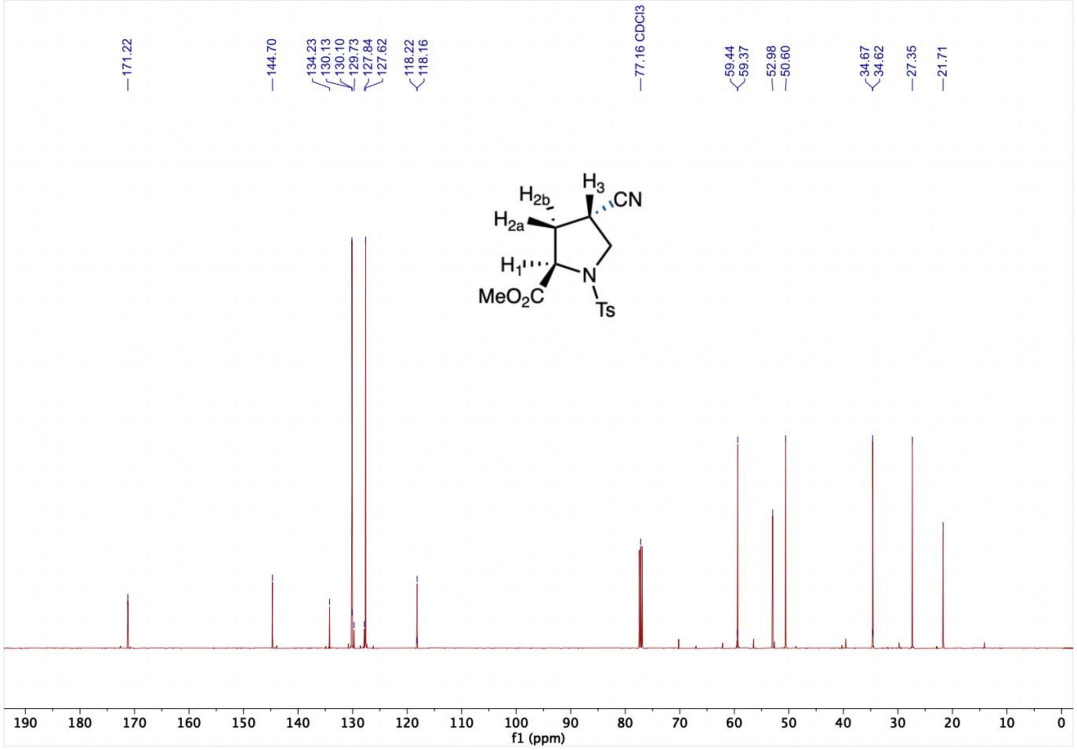

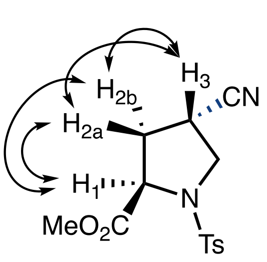


$^1$H-$^1$H COSY NMR (400 MHz, $CDCl_3$)

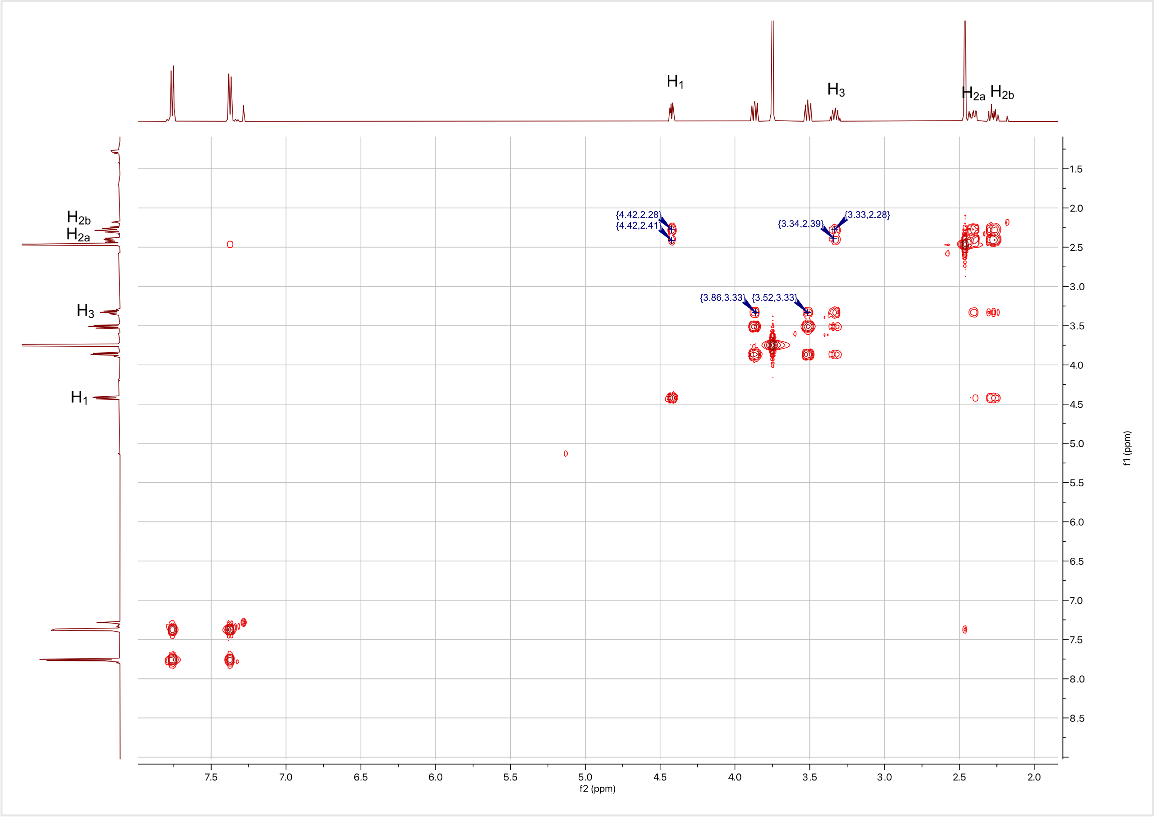

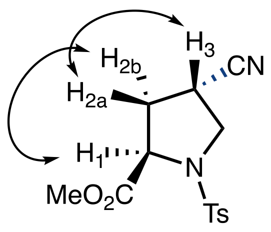


$^1$H-$^1$H NOESY NMR (400 MHz, $CDCl_3$)

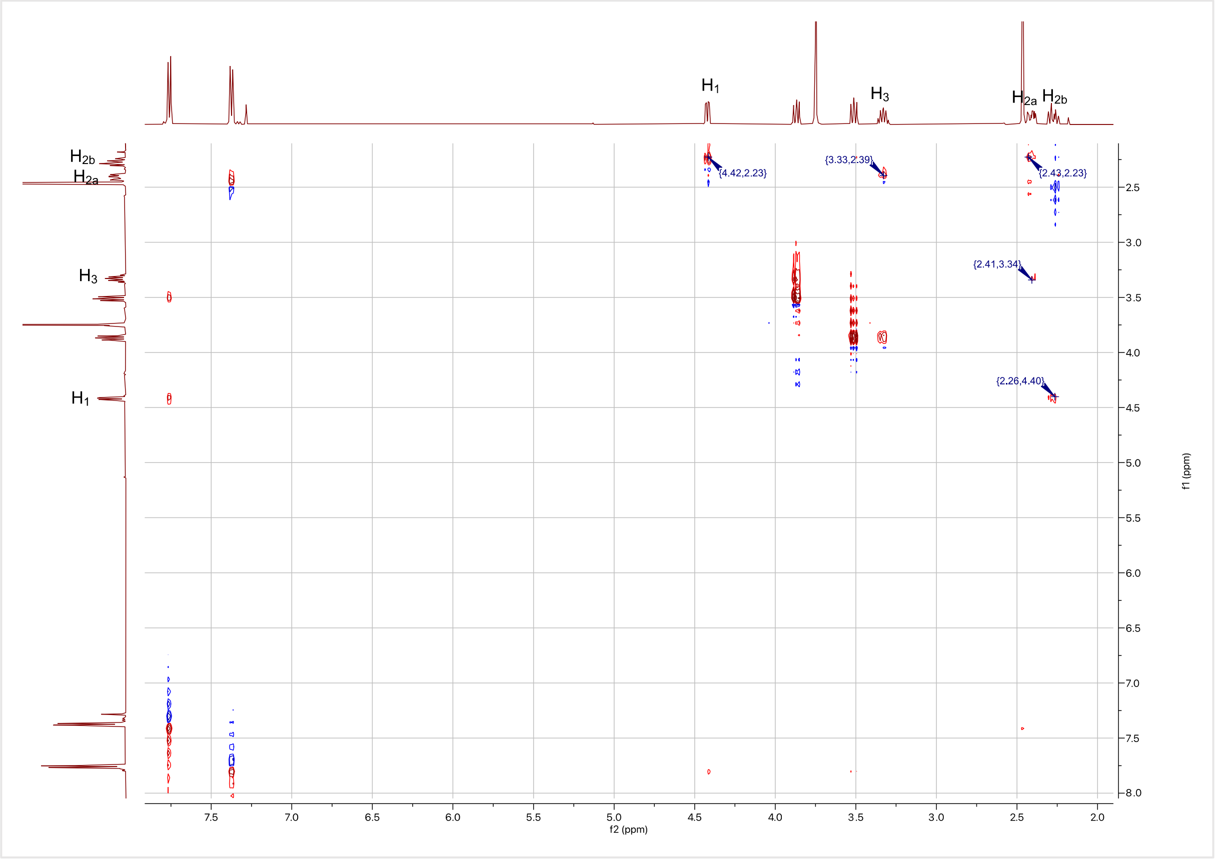

**Minor diastereomer**

$^{1}$H NMR (400 MHz, $CDCl_3$)

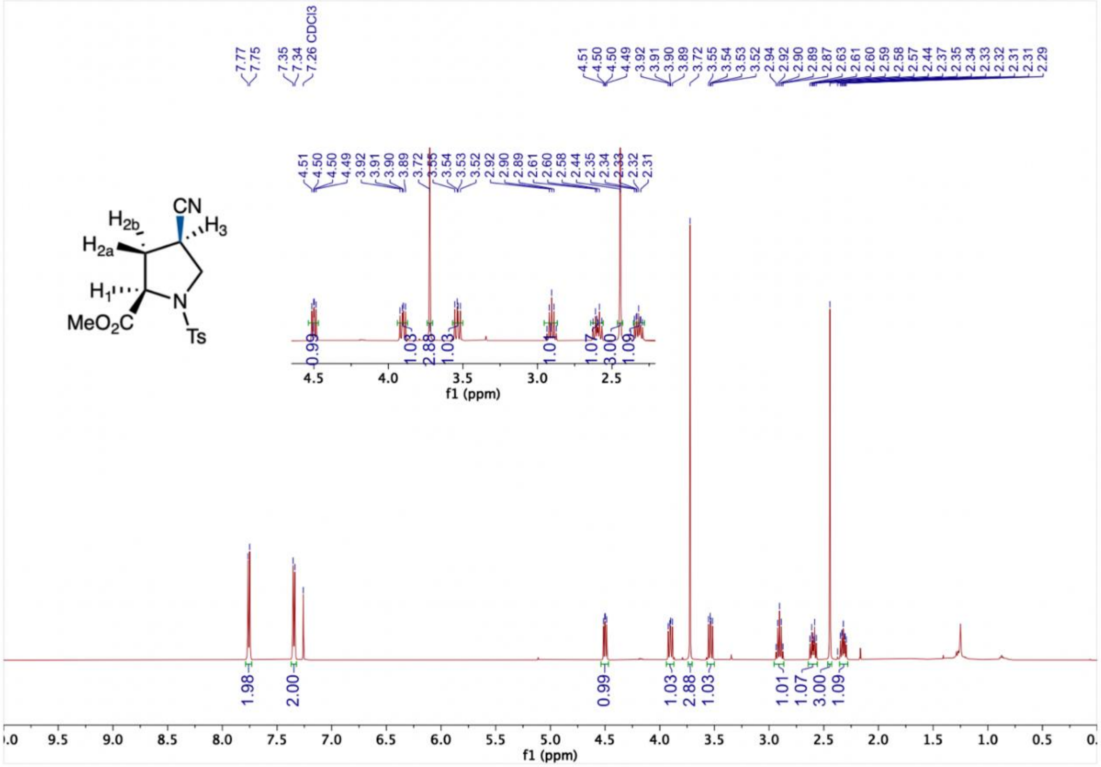

$^{13}$C NMR (126 MHz, $CDCl_3$)

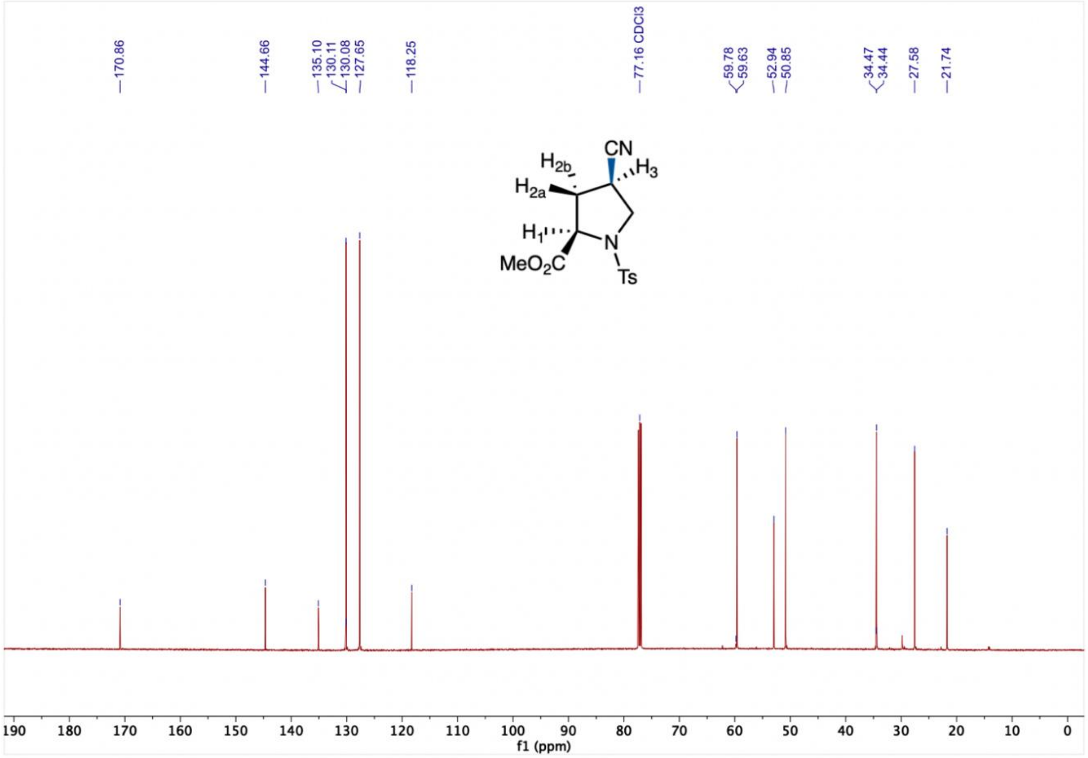

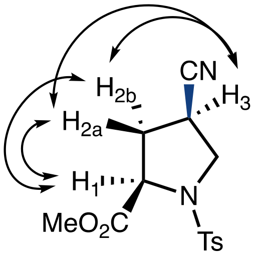


$^{1}$H-$^{1}$H COSY NMR (400 MHz, $CDCl_3$)

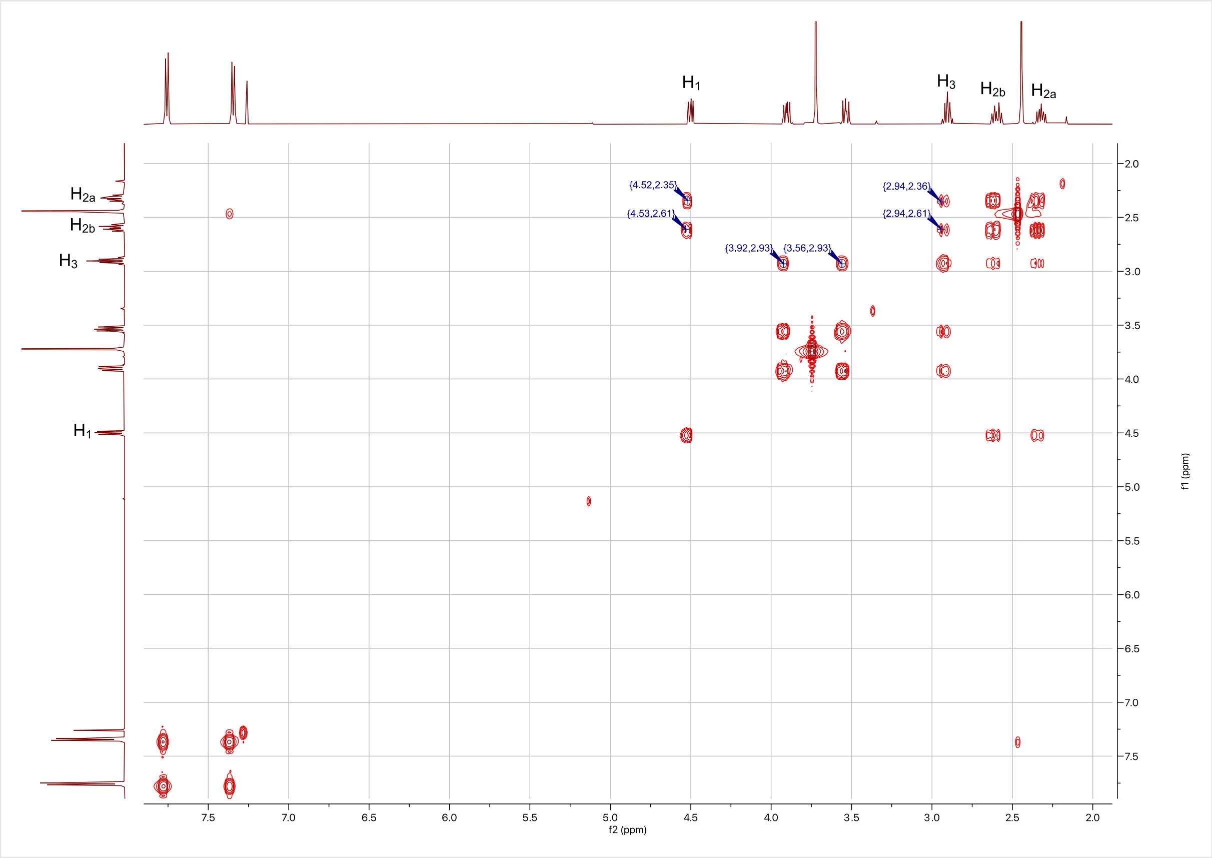

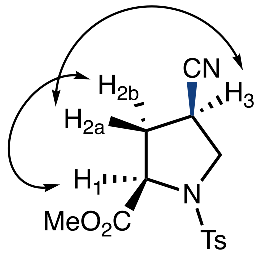


$^{1}H$-$^{1}H$ NOESY NMR (400 MHz, $CDCl_3$)

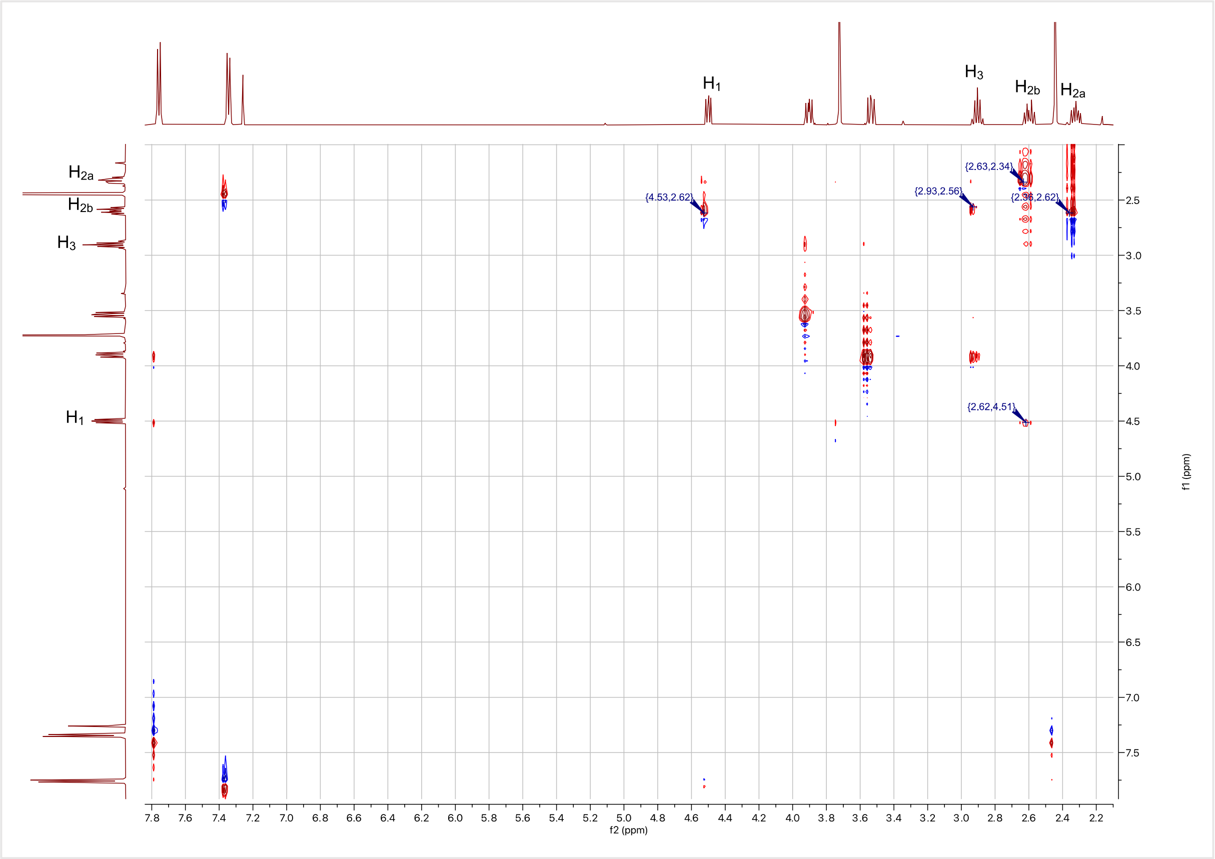

**(2*S*,3*R*,5*R*)-2-((bis(4-methoxyphenyl)(phenyl)methoxy)methyl)-5-(5-methyl-2,4-dioxo-3,4-dihydropyrimidin-1(2*H*)-yl)tetrahydrofuran-3-carbonitrile (47)**

$^{1}H$ NMR (500 MHz, $CDCl_3$)

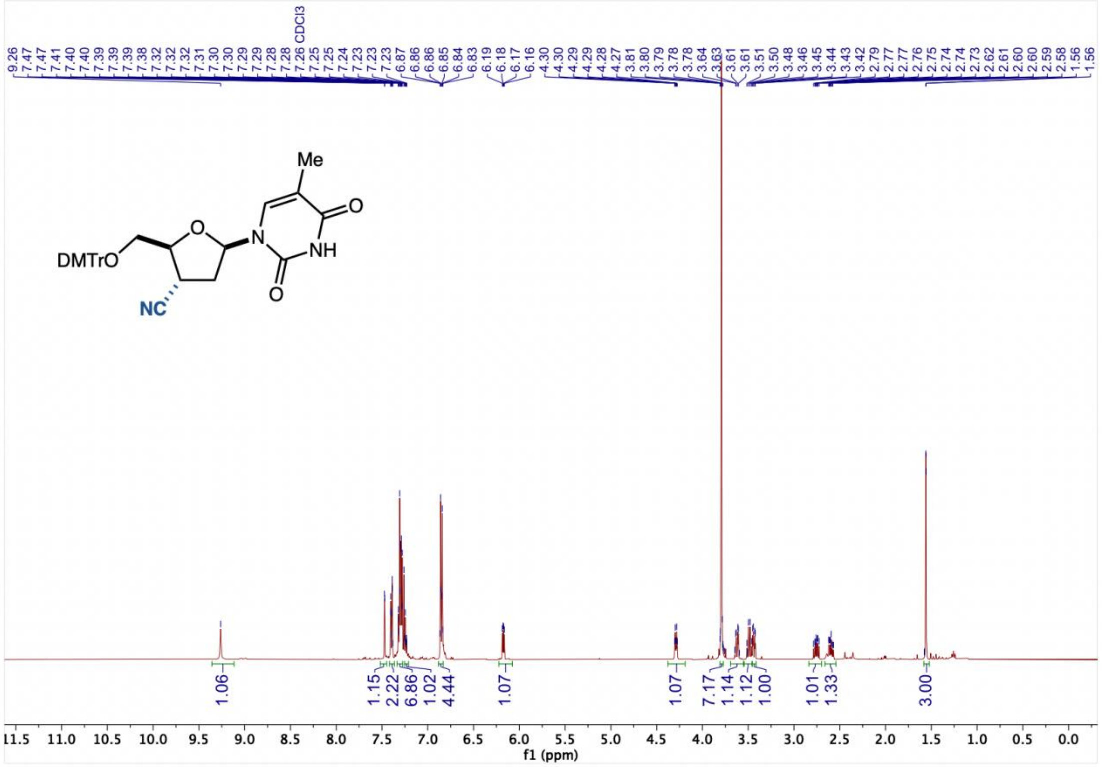


$^{13}C$ NMR (126 MHz, $CDCl_3$)

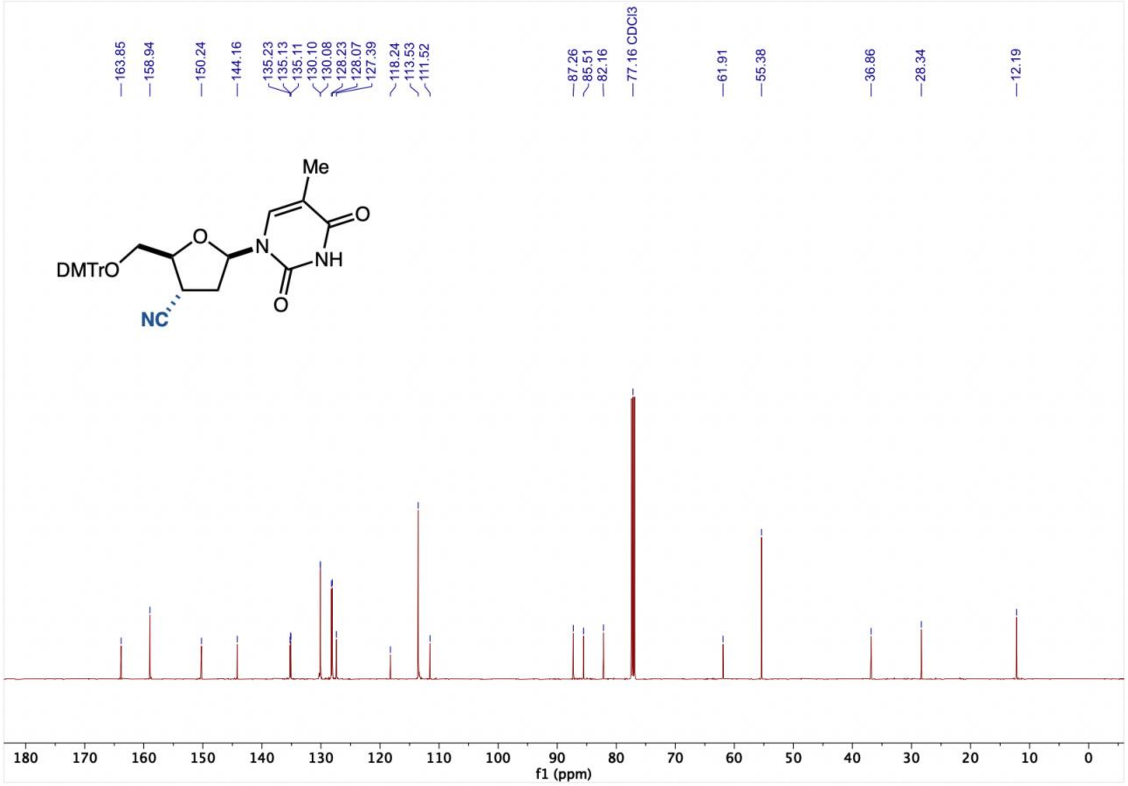

NOESY (500 MHz, $CDCl_3$)

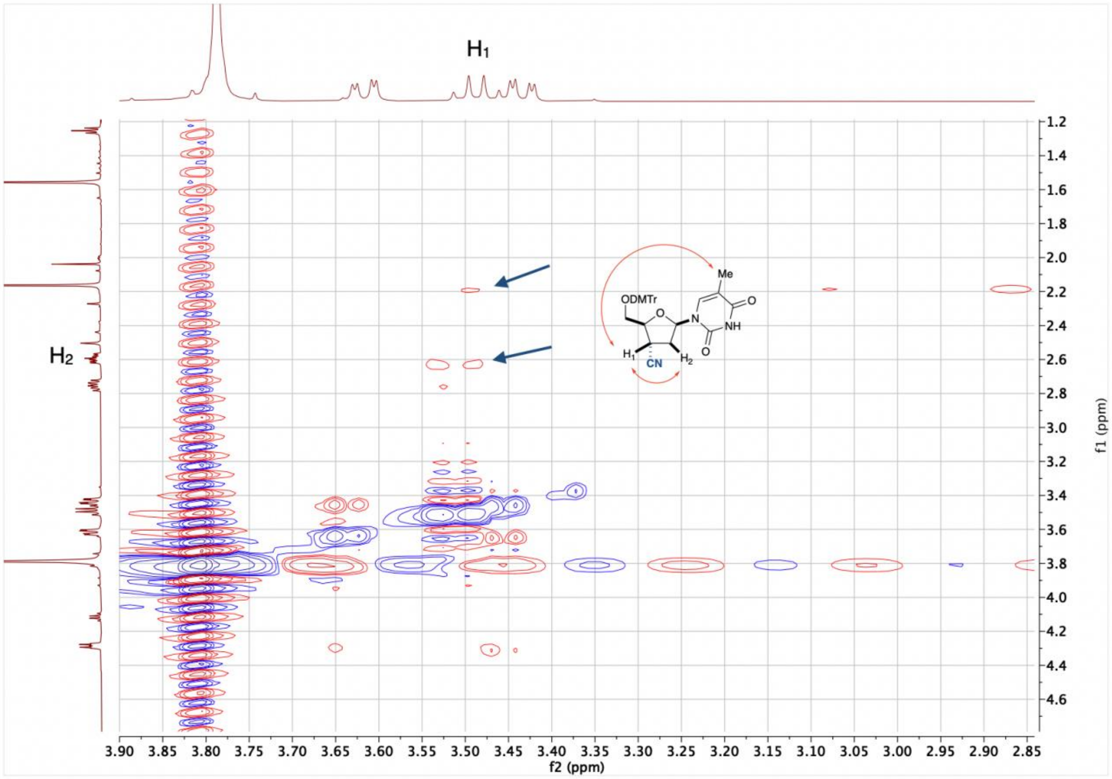


**Diethyl 2-((2-(*tert*-butoxycarbonyl)-7-(3-hydroxy-3-methylbutyl)-2-azaspiro[3.5]nonan-7-yl)methyl)malonate (48)**

$^1H$ NMR (500 MHz, $CDCl_3$)

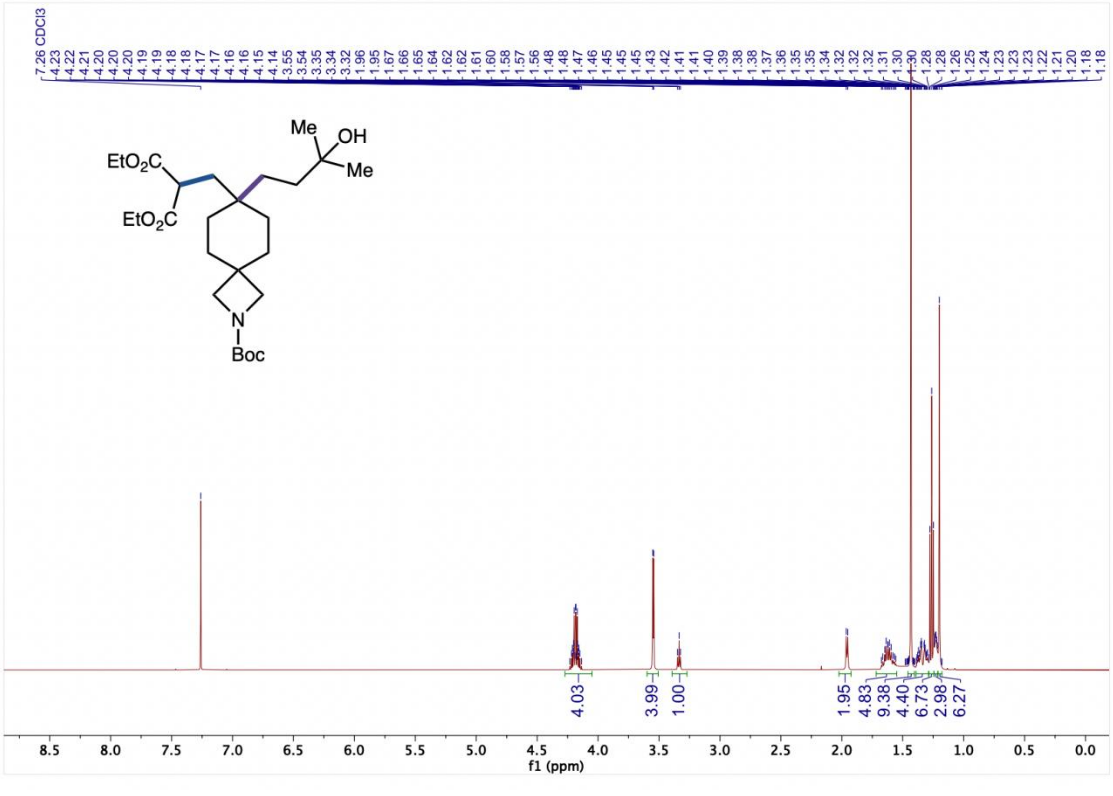

13C NMR (126 MHz, CDCl3)

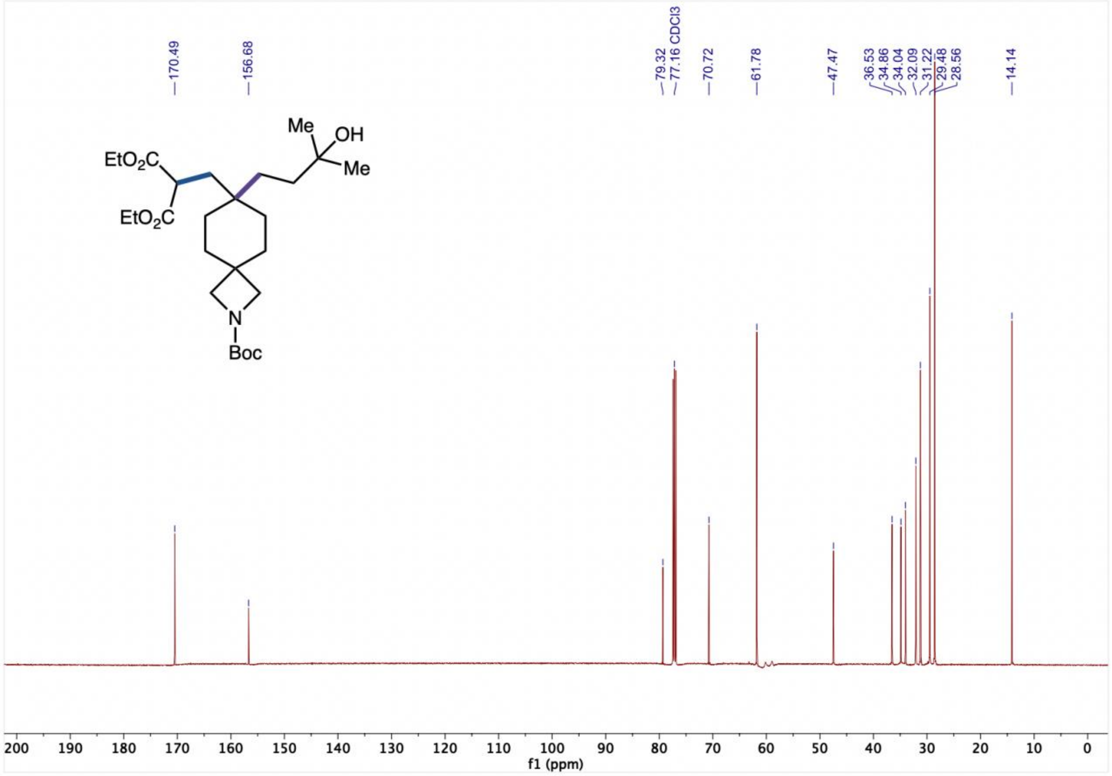


**Diethyl 2-((2-(*tert*-butoxycarbonyl)-7-(3-cyano-3-methylbutyl)-2-azaspiro[3.5]nonan-7-yl)methyl)malonate (49)**

1H NMR (500 MHz, CDCl3)

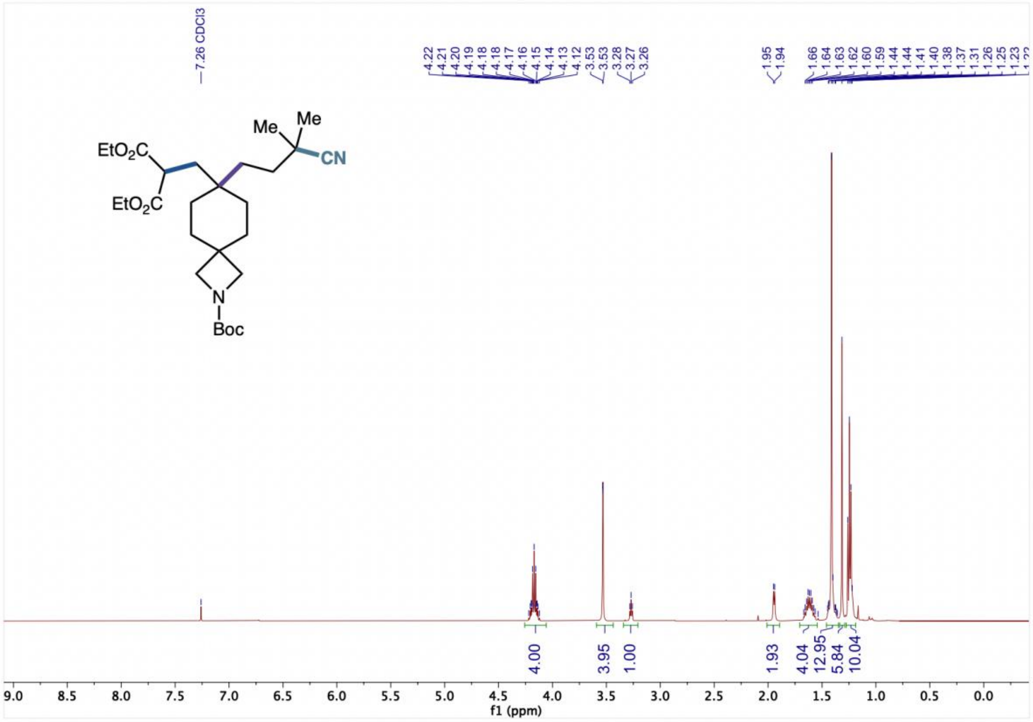

13C NMR (126 MHz, CDCl3)

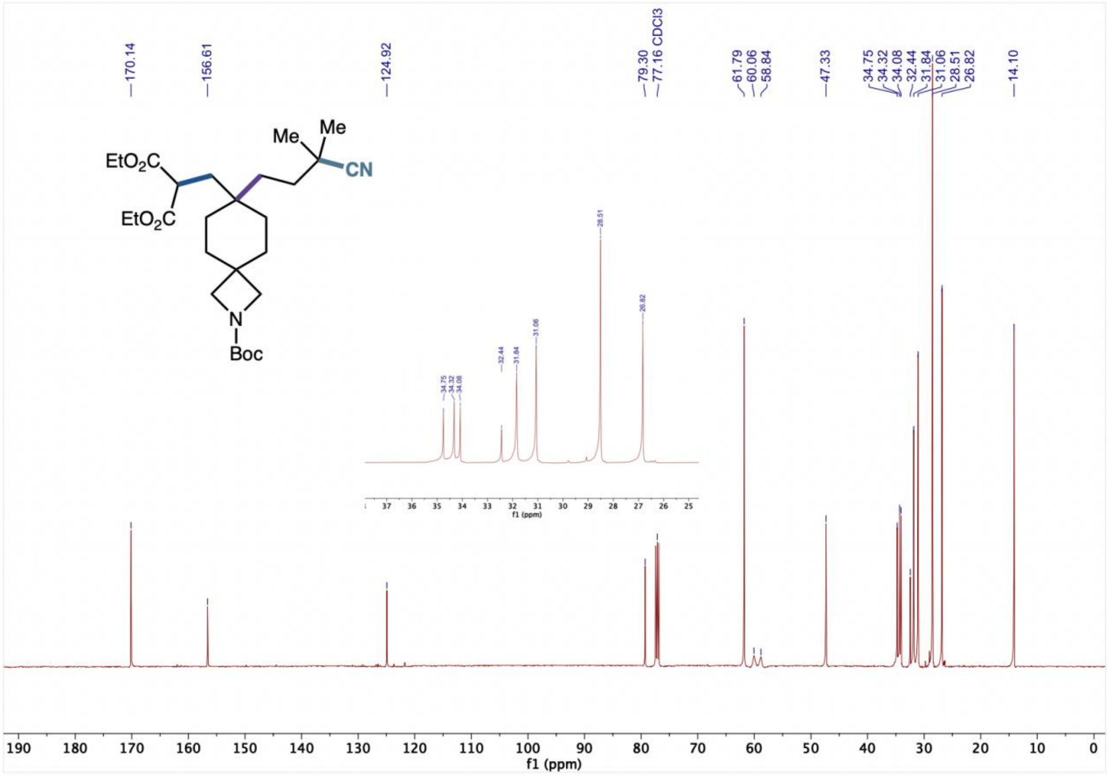


**Benzyl 4-(3-hydroxy-3-methylbutyl)-4-methylpiperidine-1-carboxylate (50)**

1H NMR (500 MHz, CDCl3)

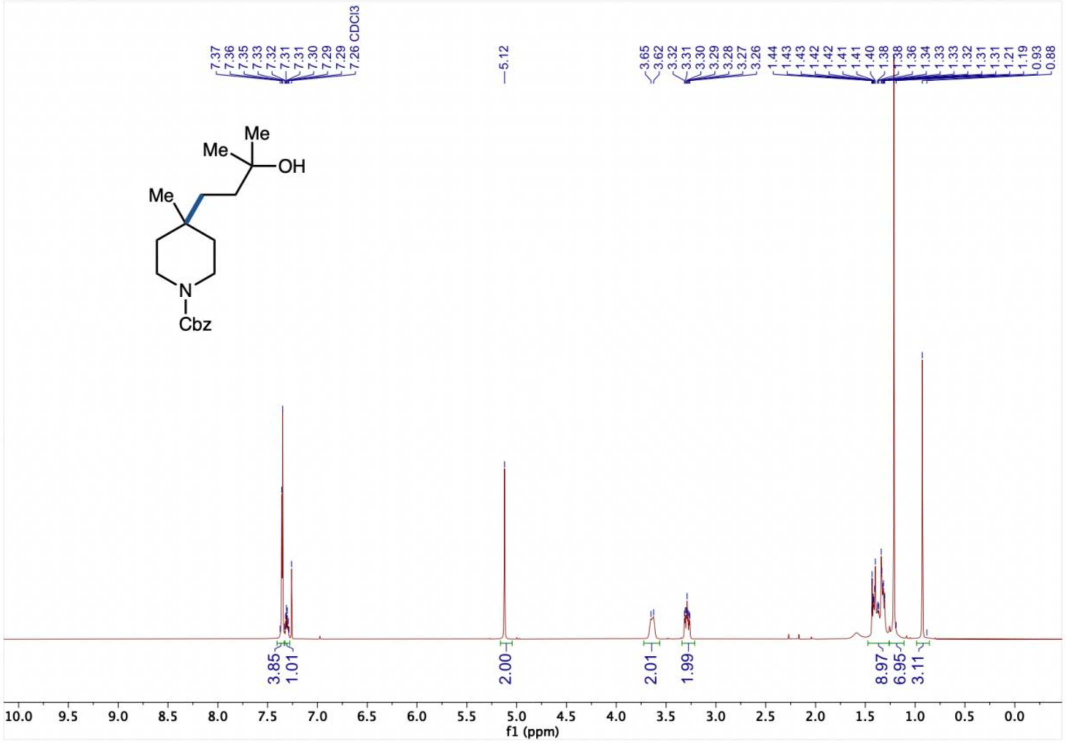

**Benzyl 4-(3-cyano-3-methylbutyl)-4-methylpiperidine-1-carboxylate (51)**

$^1H$ NMR (500 MHz, $CDCl_3$)

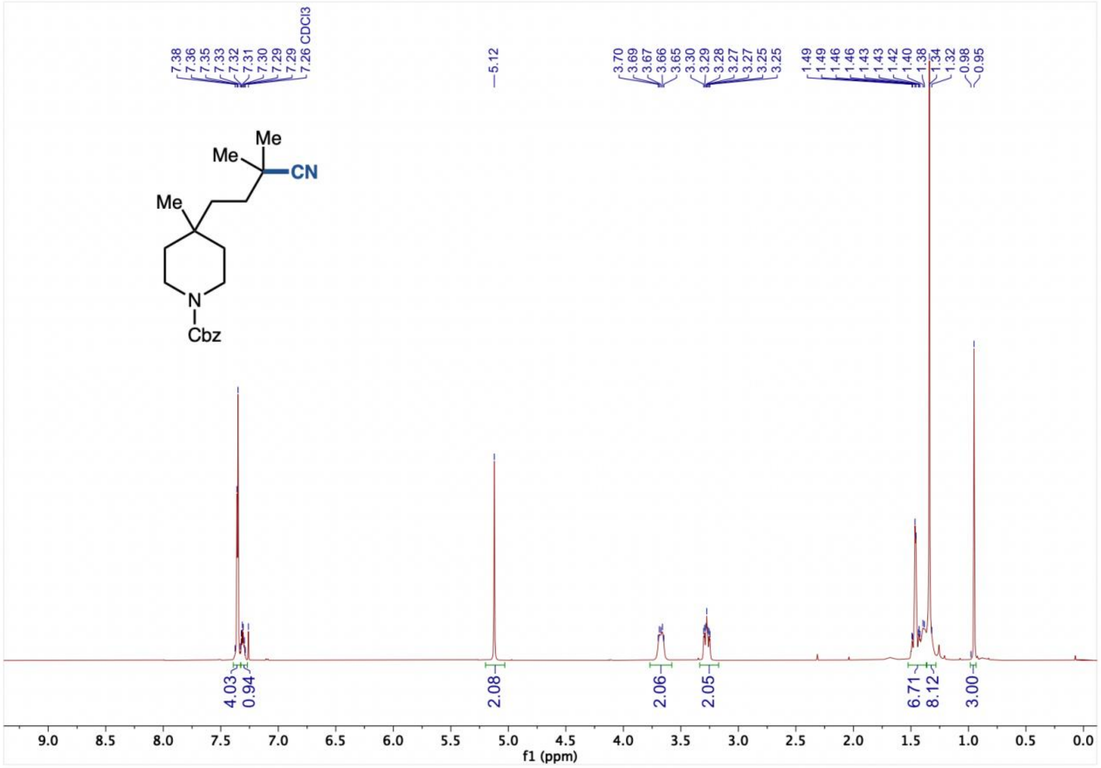


$^{13}C$ NMR (126 MHz, $CDCl_3$)

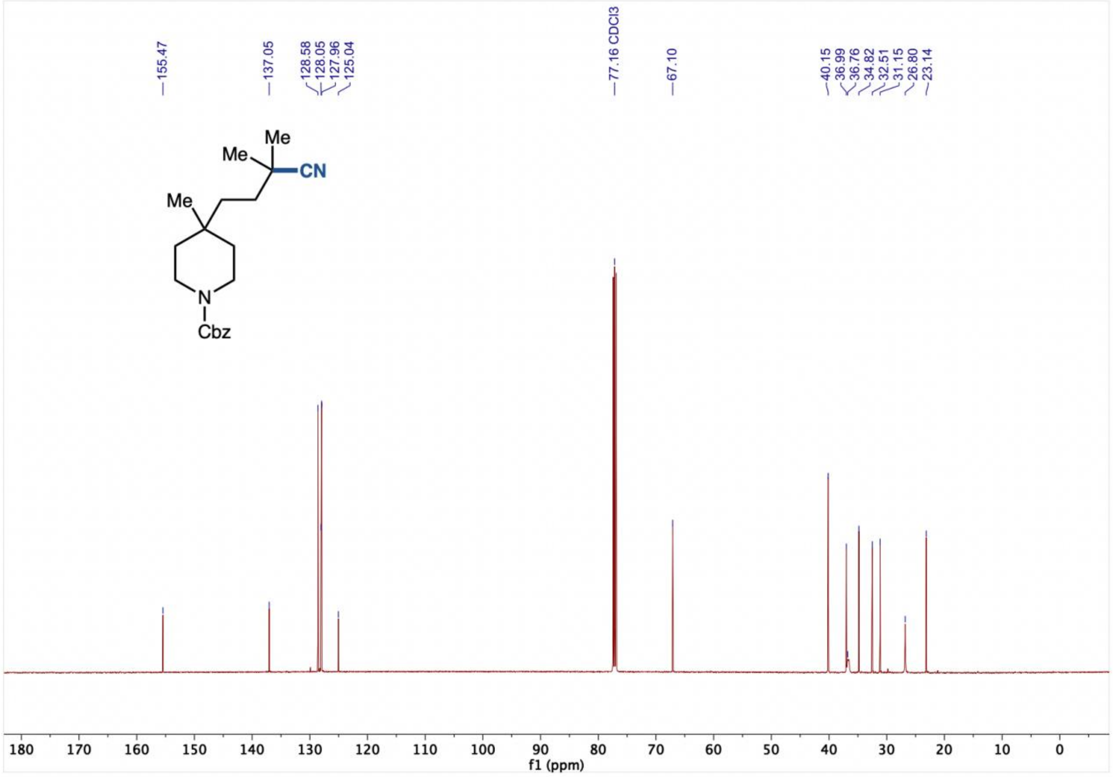

**2-(3-cyano-4-isopropoxyphenyl)-*N*-(2-(1-(3-hydroxy-3-methylbutyl)cyclohexyl)ethyl)-4-methylthiazole-5-carboxamide (52)**

$^1$H NMR (500 MHz, $CDCl_3$)

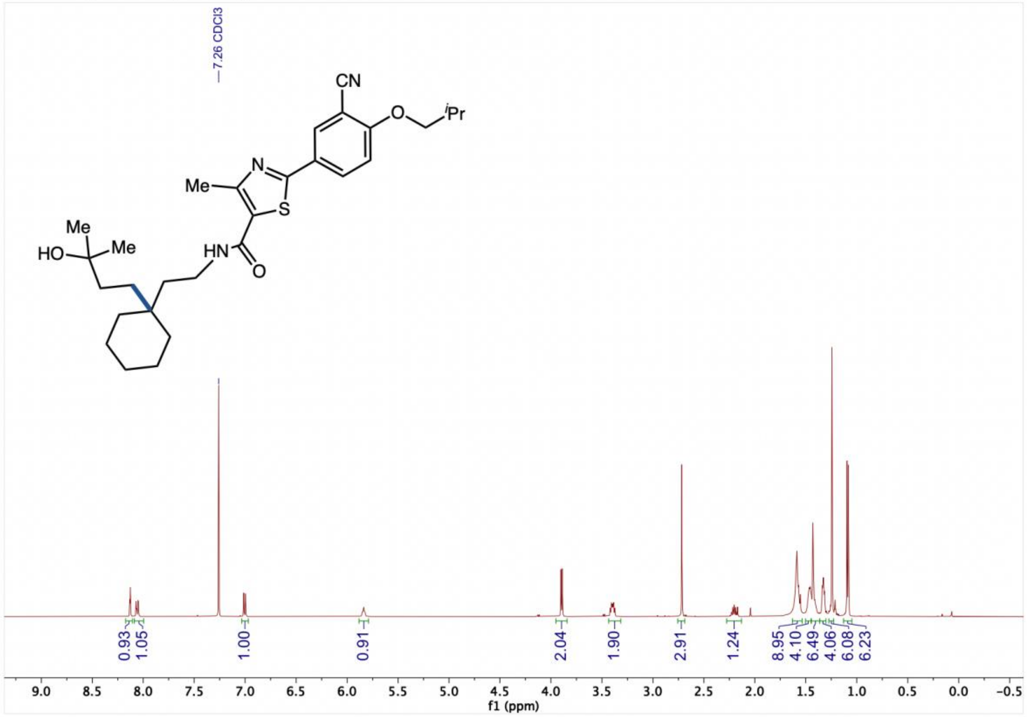


***N*-(2-(1-(3-cyano-3-methylbutyl)cyclohexyl)ethyl)-2-(3-cyano-4-isopropoxyphenyl)-4-methylthiazole-5-carboxamide (53)**

$^1$H NMR (500 MHz, $CDCl_3$)

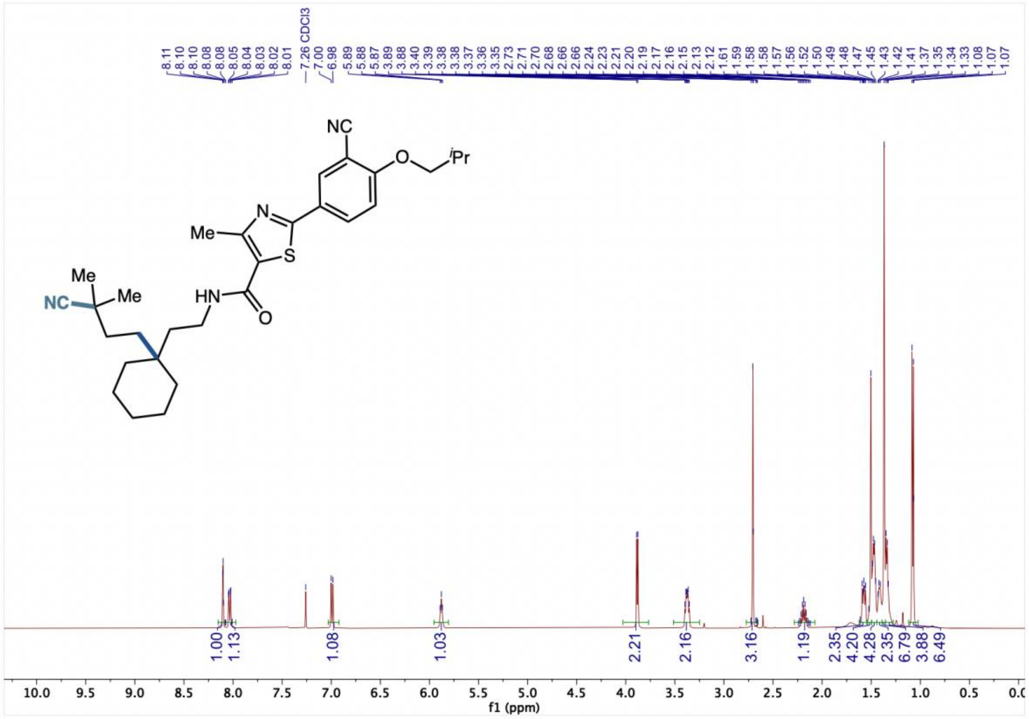

$^{13}C$ NMR (126 MHz, $CDCl_3$)

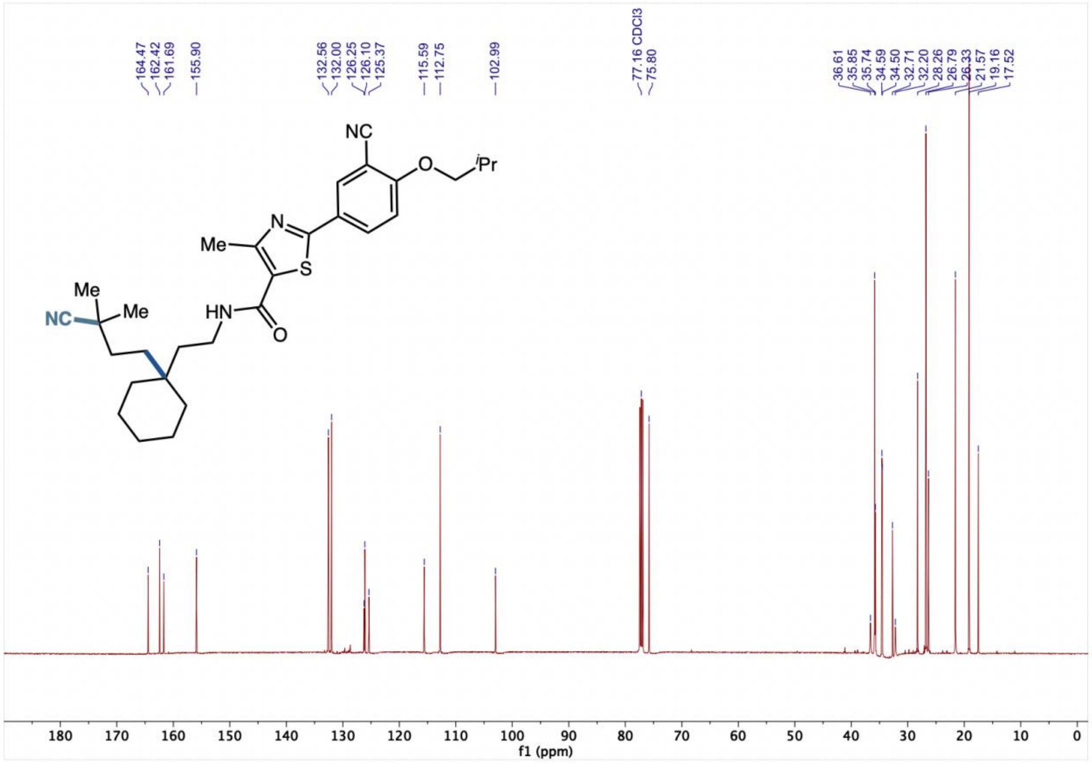

Supplement: Supplementary file 1 [file ol6c00711_si_001.pdf]
